# Cobalt-Catalyzed Hydrogenation Reactions Enabled by Ligand-Based Storage of Dihydrogen

**DOI:** 10.1021/acscatal.2c02467

**Published:** 2022-08-01

**Authors:** Sophie W. Anferov, Alexander S. Filatov, John S. Anderson

**Affiliations:** Department of Chemistry, The University of Chicago, Chicago, Illinois 60627, United States

**Keywords:** catalysis, redox-active, cobalt, hydrogenation, hydrogen

## Abstract

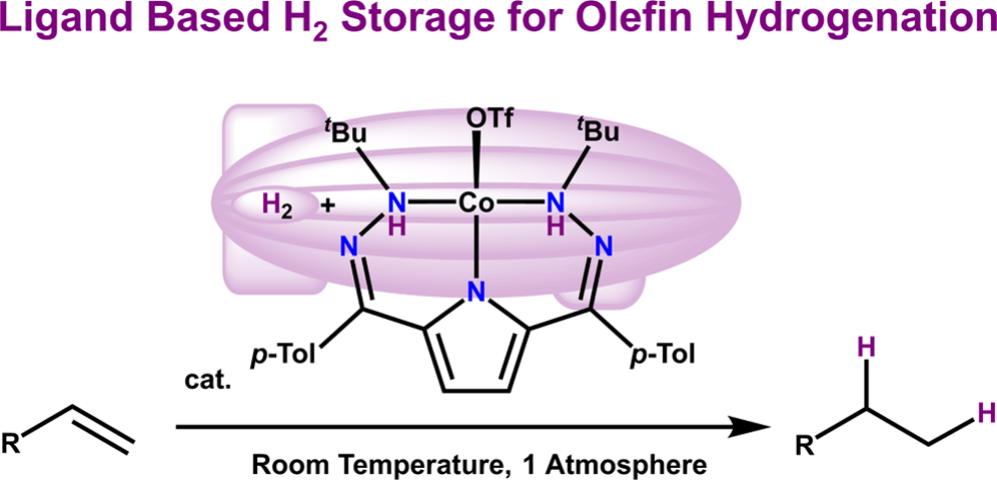

The use of supporting ligands that can store either protons or electrons has emerged as a powerful strategy in catalysis. While these strategies are potent individually, natural systems mediate remarkable transformations by combining the storage of both protons and electrons in the secondary coordination sphere. As such, there has been recent interest in using this strategy to enable fundamentally different transformations. Furthermore, outsourcing H-atom or hydrogen storage to ancillary ligands can also enable alternative mechanistic pathways and thereby selectivity. Here, we describe the application of this strategy to facilitate radical reactivity in Co-based hydrogenation catalysis. Metalation of previously reported dihydrazonopyrrole ligands with Co results in paramagnetic complexes, which are best described as having Co(II) oxidation states. These complexes catalytically hydrogenate olefins with low catalyst loadings under mild conditions (1 atm H_2_, 23 °C). Mechanistic, spectroscopic, and computational investigations indicate that this system goes through a radical hydrogen-atom transfer (HAT) type pathway that is distinct from classic organometallic mechanisms and is supported by the ability of the ligand to store H_2_. These results show how ancillary ligands can facilitate efficient catalysis, and furthermore how classic organometallic mechanisms for catalysis can be altered by the secondary coordination sphere.

## Introduction

The efficient shuttling of protons and electrons is key for many chemical transformations. Metal centers are often employed to facilitate this reactivity, but multi-proton/electron transformations remain challenging, particularly with abundant first-row transition metals with predominant one-electron reactivity.^1^ Natural systems have evolved to optimize the use of first-row metals by leveraging a secondary coordination sphere tailored to the needs of a given reaction.^2^ Given that the number of protons and electrons stored in the secondary coordination sphere is, in principle, only limited by ligand design, this strategy also opens the door for challenging reactions requiring the transfer of many proton and electron equivalents.

Common secondary sphere motifs utilized in synthetic systems include hydrogen bonding ligands,^3^ proton-shuttling functionalities,^4^ and redox-active sites that enable electron storage and transfer.^5^ Significant effort over the past few decades has illustrated the value of these strategies in facilitating or altering reactivity patterns, but examples where both protons and electrons can be stored on ancillary ligands are comparatively less common. There are several well-defined systems that store H-atom equivalents,^6^ and examples of the storage of a full equivalent of dihydrogen in a ligand backbone are even more rare.^7,8^ Despite their scarcity, such ligand scaffolds are promising candidates to efficiently facilitate challenging multi-proton/electron transfers in catalysis.

Our laboratory has been interested in first-row transition metal complexes ligated by dihydrazonopyrrole (DHP) ligands. These complexes can reversibly transfer dihydrogen stored on the ligand framework, which enables the catalytic hydrogenation of benzoquinone in a Ni-based system.^9,10^ Related Fe complexes are also able to transfer H-atoms to O_2_ to generate hydroperoxo intermediates and ultimately H_2_O_2_ using ligand-derived H-atom equivalents.^11^ We rationalized that striking a balance between redox and spin-state flexibility, as present with Fe complexes, and more classic organometallic metals, such as Ni, might be advantageous for new catalytic transformations.^10,11^

**Figure 1:**
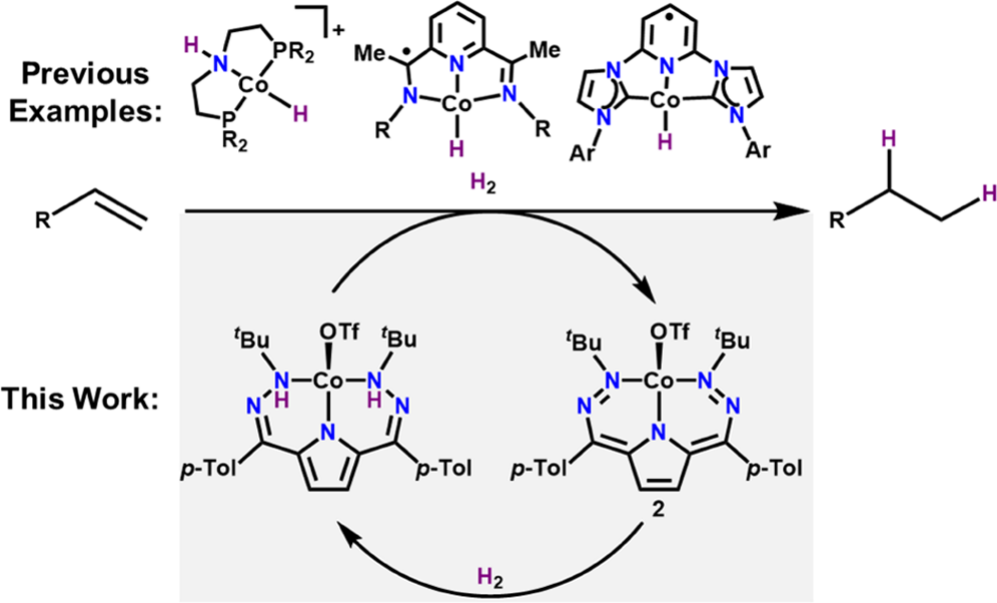
Existing Co-based hydrogenation catalysts or active species^12c,12e,14a,14b,14d,14e,15a^ and the current system highlighting ligand-based H_2_ storage.

In this context, Co features prominently among first-row transition metals in hydrogenation catalysis. Unlike Rh and Ir, Co does not necessarily proceed through classical two-electron transformations and, as with other first-row transition metals, exhibits a propensity for single-electron steps and varied spin states.^12−14^ These alternative trends can also be leveraged to obtain altered reactivity. For instance, recent reports have illustrated how Co complexes with ligands that can store protons or electrons can efficiently mediate catalytic hydrogenations, with some examples exhibiting alternative mechanistic pathways in the presence of light (Figure 1).^12,14^

Given this precedent for Co in hydrogenation chemistry and the opportunities that it presents as a first-row metal, we were interested in examining the interplay between Co centers and DHP ligands in H-transfer chemistry. Specifically, we sought to investigate whether Co DHP complexes were viable hydrogenation catalysts and, if so, whether the DHP ligand would enable alternative mechanisms to more canonical organometallic pathways.^12−14^ Here, we present a series of ^*t*Bu,Tol^DHP Co catalysts that mediate olefin hydrogenation via a ligand-assisted hydrogenation pathway. Catalysis occurs efficiently under mild conditions, comparable with the best Co hydrogenation catalysts currently known.^14e,15^ Spectroscopic, computational, and mechanistic investigations demonstrate that catalysis proceeds via a radical H-atom transfer mechanism supported by the ^*t*Bu,Tol^DHP ligand. These results illustrate how cooperativity between Co centers and ligands which can store H-atom or H_2_ equivalents enables efficient catalysis with novel mechanistic paradigms.

## Results and Discussion

### Synthesis and Characterization of **1**

**Figure 2:**
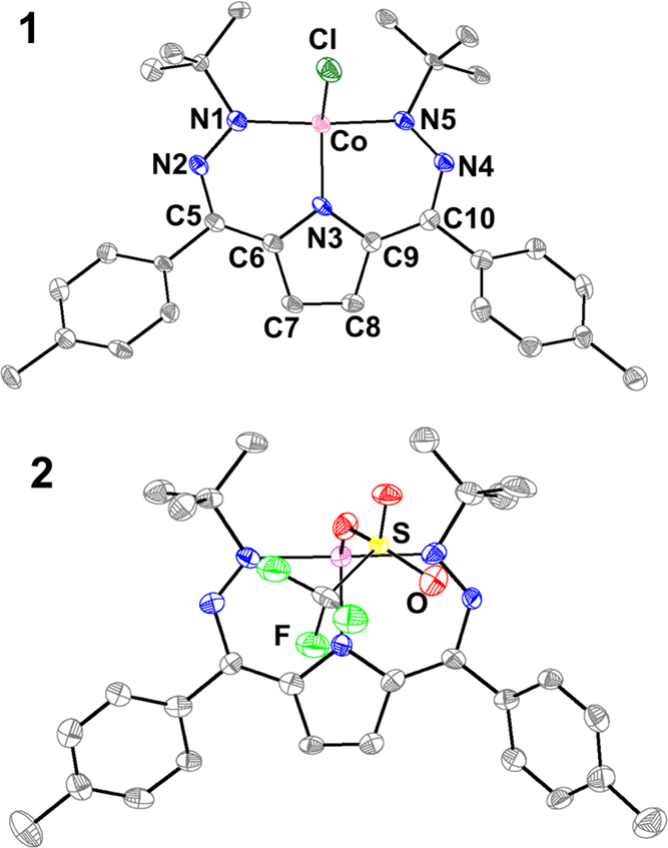
SXRD Structures (from left to right) of **1** and **2**. All displacement ellipsoids shown at 50%, and hydrogens omitted for clarity.

**Scheme 1:**
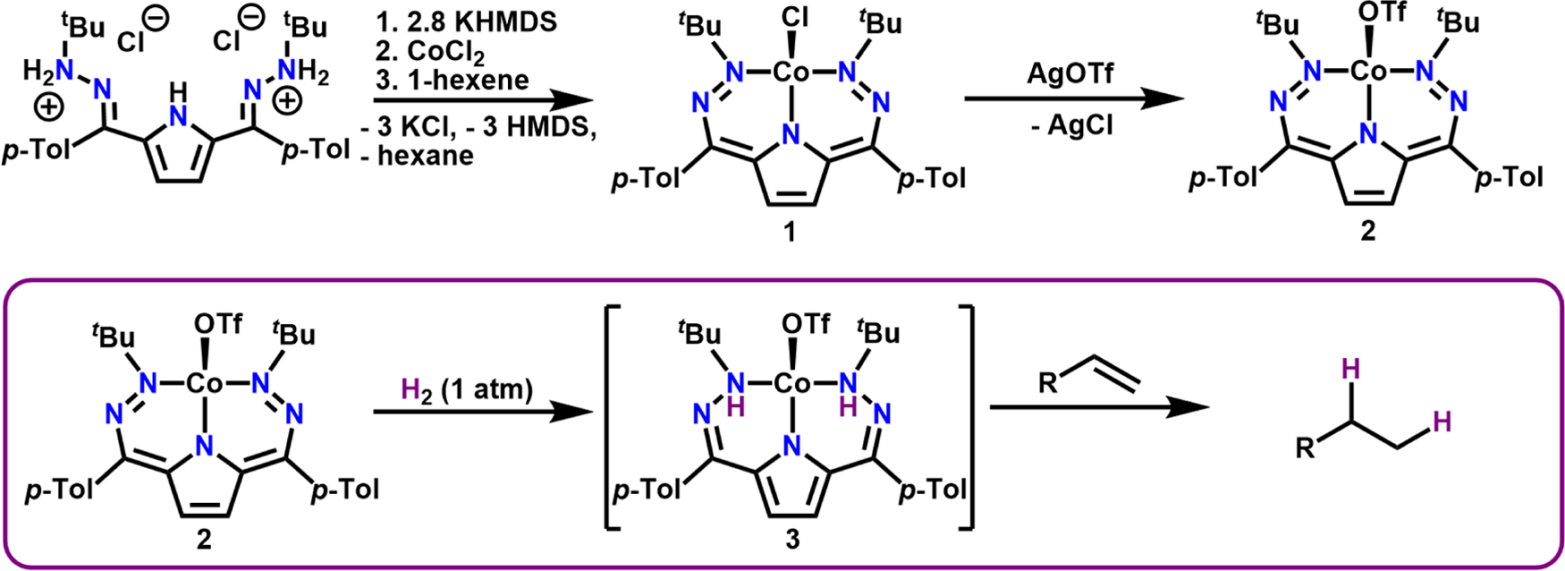
Synthesis of ^*t*Bu,Tol^DHP Complexes of Co and Hydrogenation Reactivity

Deprotonation of ^*t*Bu,Tol^DHP·2HCl^9c^ with 2.8 equiv of KHMDS followed by the rapid addition of a CoCl_2_ suspension in THF and a single equivalent of 1-hexene (acting as a H_2_ acceptor) provides [^*t*Bu,Tol^DHP]CoCl (**1**) as a magenta-purple solid in 75% yield (Scheme 1). We note this reaction still provides **1** as the major product in the absence of 1-hexene as a H_2_ acceptor, albeit in lower yields. Dark purple crystals suitable for single-crystal X-ray diffraction (SXRD) reveal a four-coordinate Co complex. The geometry of **1** can be quantified with τ_4_ and τ_4_′ values of 0.473 and 0.363, respectively, suggesting a see-saw complex with some tetrahedral character (Figure 2 and Table 1).^16^ Complex **1** is paramagnetic with an *S* = 1/2 solution spin state as determined by Evans’ method. This suggests either a low-spin Co(II) center with a DHP^–^ ligand or a Co(III) center with a DHP^2–•^ ligand radical. We note that there are several examples of low-spin Co(II) complexes in similar geometries, including a number of imino-pyridine ligated Co(II) complexes as well as Co porphyrin and corrin species.^17,18,21^

**Table 1:** Selected Bond Lengths (Å) and Angles (°) of 1, 2, and Related Ni Complexes

	1	2^a^	[DHP^2–•^]NiL^9a^	[DHP^–^]NiL^+^^9a^
M-N1/M-N5	1.891(5)	1.994(8)	1.866(2)	1.864(2)
	1.883(4)	2.000(8)	1.863(2)	1.860(2)
M-N3	1.861(4)	1.916(8)	1.872(2)	1.869(2)
N1-N2/N4-N5	1.303(6)	1.27 (1)	1.342(2)	1.302(2)
	1.288(6)	1.25(1)	1.337(3)	1.314(2)
M-X(Cl/O/P)	2.198(2)	2.168(7)	2.2319(7)	2.2630(7)
M-X(N/O)		2.126(7)		
N2-C5/N4-C10	1.343(7)	1.37(1)	1.319(3)	1.348(2)
	1.338(7)	1.39(1)	1.322(2)	1.342(3)
C5-C6/C9-C10	1.383(8)	1.38 (1)	1.417(3)	1.388(3)
	1.388(8)	1.38(1)	1.425(3)	1.393(3)
C6-C7/C8-C9	1.434(8)	1.43(1)	1.413(3)	1.444(3)
	1.433(8)	1.45(1)	1.421(3)	1.452(3)
C7-C8	1.351(8)	1.31(1)	1.371 (3)	1.343 (3)
N1-M-N5	164.5(2)	178.1(3)	163.59(8)	161.85(7)
N3-M-X	129.1(2)	113.2(3)	145.30(6)	142.64(5)
		105.1(3)		

a Crystallizes as a polymeric chain; monomer shown. L = PMe_3_ as described in the text.

The structure of **1** provides the opportunity to examine limiting electronic structures. We have found that the electronic structure of the DHP ligand results in changes to specific bonds in the scaffold in a diagnostic manner, namely, the N1-N2/N4-N5, C5-C6/C9-C10, and C7-C8 distances.^9,10^ Comparison of these distances between **1** and the previously reported four-coordinate Ni complexes [^Ph,Tol^DHP]NiPMe_3_^*n*+^ (*n* = 0 and 1) suggests that the DHP ligand in **1** is best considered as a closed-shell monoanionic DHP^–^ unit, thereby implying a Co(II) oxidation state.^9a^ However, this interpretation is convoluted by the differing metal centers and ligand sets in this comparison.

Complex **1** displays four distinct redox waves in its cyclic voltammogram (CV, Figure S19). This electrochemical data shows that the ^*t*Bu,Tol^DHP scaffold supports unusually rich redox flexibility in this system. Despite this, complex **1** does not exhibit discernible reactivity with H_2_. We hypothesized that the exchange of chloride for a less-coordinating anion could promote reactivity with H_2_ and potentially catalysis.

### Synthesis and Characterization of **2**

Complex **1** reacts with AgOTf in a mixture of benzene/acetonitrile to produce [^*t*Bu,Tol^DHP]CoOTf (**2**) as a maroon solid (Scheme 1). Similar to **1**, complex **2** is also paramagnetic with an *S* = 1/2 spin state as determined by Evans’ method. SXRD analysis on the very dark crystals of **2** reveals a Co center that is five-coordinate due to the formation of a polymeric chain from bridging OTf^–^ ligands in the solid state. The τ_5_ value for this complex is 0.605, putting it closer to trigonal bipyramidal than square pyramidal (at 1 and 0, respectively).^19^ Examination of the monomeric repeat unit of **2** reveals that the bond lengths within the DHP ligand undergo changes from those in **1**, which suggests a more distinctively oxidized DHP^–^ ligand, and hence also a Co(II) oxidation state assignment (Figure 1 and Table 1). The CV of **2** is qualitatively similar to that of **1**, albeit less reversible overall, likely due to enhanced lability of the OTf^–^ counteranion (Figure S20).

We then acquired electron paramagnetic resonance (EPR) spectroscopy to better understand the relative electronic structures of complexes **1** and **2**. The EPR of **1** is rhombic, with *g*-values of 2.02, 2.10, and 2.58 (Figures S21 and S22). EPR spectra of organic radicals are typically more isotropic and with all *g*-values near *g* = 2.0, suggesting that a Co(II) electronic structure may be appropriate.^20^ Indeed, literature examples where a five- or six-coordinate Co(III) center is bound to a ligand radical as well as previous examples with DHP radical ligands, namely, [^*t*Bu,Tol^DHP^2–•^]Ni and [^Ph,Tol^DHP^2–•^]Ni, all have smaller *g*-anisotropy than that of **1**, further supporting a [DHP^–^]Co(II) resonance structure.^9a,21,9c^ While the greater *g*-anisotropy of **1** is different from low spin, square-planar cobalt complexes,^22^ it is distinctly similar to related tetrahedral or see-saw complexes.^23^ The best simulation we have obtained uses hyperfine coupling (MHz) to both ^59^Co (*A_xx_* = 57.6, *A_yy_* = 62.4, *A_zz_* = 58.8) and ^14^N (*A_xx_* = 35.4, *A_yy_* = 44.7, *A_zz_* = 10.7), although we note that the complicated pattern means that alternative spin systems, for instance, those with coupling to more than one ^14^N nucleus, may also provide satisfactory fits. While the *g*-anisotropy and ^59^Co hyperfine constants support a Co(II) oxidation state, the large ^14^N hyperfine suggests that there is still significant spin on the DHP ligand. Density Functional Theory (DFT) calculations with the B3P functional support this notion. While the majority of the spin density is localized on Co, a significant fraction (30%) is present on the DHP ligand (Figure S35).

**Figure 3:**
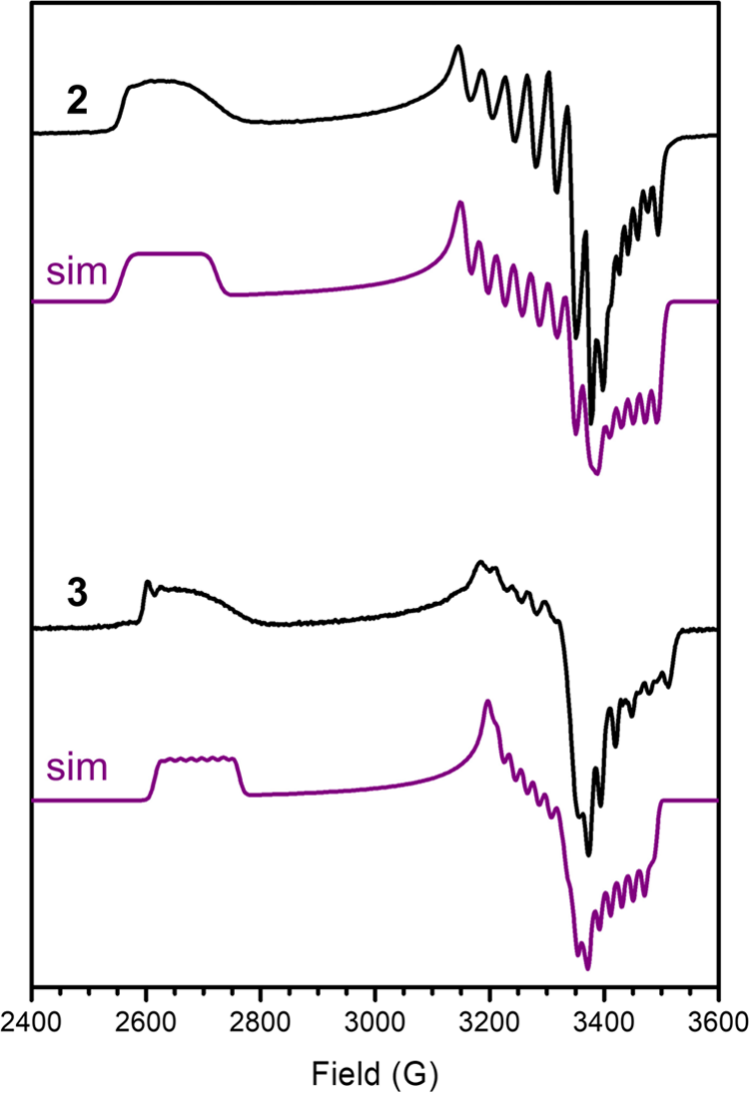
Perpendicular mode X-band EPR spectrum from top to bottom of 15 mM solutions of **2** and **3** at 15.9 K in toluene and 20 K in toluene, respectively. Simulation shown in black lines for each. Simulation parameters for **2**: *g* = 2.01, 2.11, 2.61; Co-A = +56.5, +89.0, +75.6 MHz; N-A = +12.1, +5.9, +11.2 MHz. **3**: *g* = 2.02, 2.10, 2.56; Co-A = +54.8, +61.0, +66.8 MHz, N-A = +23.1, +24.5, −14.1 MHz. Experimental conditions: microwave frequency 9.6304 GHz, microwave power 0.2 mW. The full and simulated spectra are shown in the SI (Figures S21–S27 and Table S1).

The EPR spectrum of **2** is similarly rhombic to that of **1** with *g*-values of 2.01, 2.11, and 2.61, and our best simulation similarly features coupling (MHz) to both ^59^Co (*A_xx_* = 56.5, *A_yy_* = 89.0, A_zz_ = 75.6) and ^14^N (*A_xx_* = 12.1, *A_yy_* = 5.9, *A_zz_* = 11.2) (Figure 3). As with **1**, this data suggests that the best description of **2** is as a low-spin Co(II) center with an oxidized monoanionic ^*t*Bu,Tol^DHP ligand. Consistent with the structural data above, the relative *g*-anisotropies and hyperfine constants between **1** and **2** both support that **2** is closer to a “pure” Co(II) resonance structure, although we note that DFT calculations still support some radical character on the DHP ligand (20%, Figure S37).

As a final probe of electronic structure, we obtained Co K-edge X-ray absorption spectroscopy (XAS) data on both **1** and **2**. The pre-edge feature for **1** is only slightly shifted to higher energy from **2** by 0.4 eV (from 7709.3 to 7709.7 eV), which is at the experimental resolution, and the K-edges for these two complexes are also quite similar (Figures S28–S29).^24^ This data again supports similar electronic structures between **1** and **2** and are consistent with other Co(II) complexes.^14b^^25^ Thus, while the electronic structures of these complexes, particularly **1**, are highly covalent with reasonably invoked DHP non-innocence, the best limiting resonance contributor is [DHP^–^]Co(II).

### Generation and Characterization of **3**

**Figure 4:**
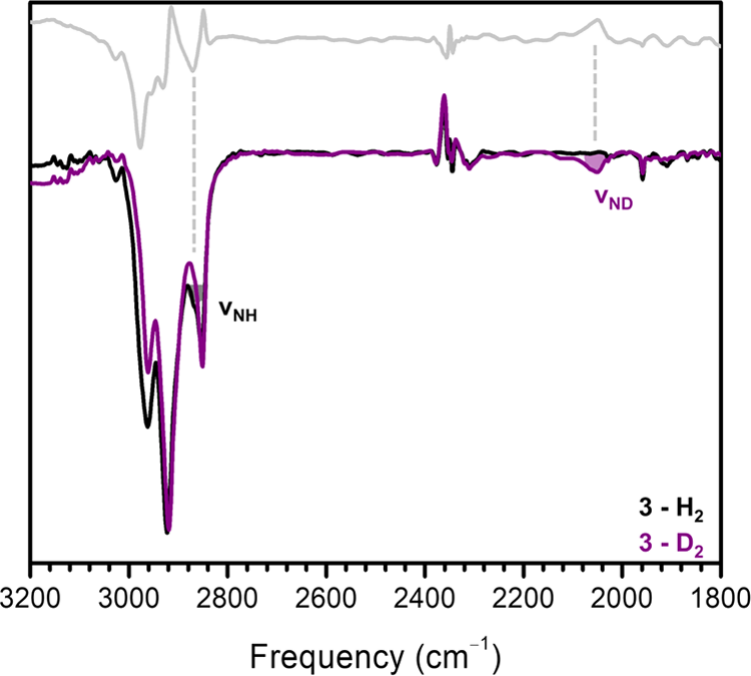
Thin-Film IR spectrum of **3** and **3-D**_**2**_ with difference spectrum in the inset.

While **1** shows no reactivity with H_2_, addition of H_2_ to cold solutions of **2** results in a new magenta product (**3**) (Scheme 1). Complex **3** forms very slowly, taking over 48 h for complete conversion at −25 **°**C, and is unstable at and above 0 **°**C. The use of D_2_ results in significantly slower conversion but still allows for the formation of enough **3-D**_**2**_ for IR characterization (Figure 4). Though **3** is unstable to higher temperatures, it is stable to vacuum once formed.

The ^1^H NMR spectrum of **3** is paramagnetic and broad (Figure S2). This is consistent with 2 e^–^ reactivity with H_2_, and we have tentatively assigned **3** as the hydrogenated product, [^*t*Bu,Tol^DHP-H_2_]CoOTf, analogously to the reactivity observed with a related Ni system.^10^ The EPR signal of **3** at 20 K further supports this assignment, with an observed major *S* = 1/2 signal along with a small amount (<10%) of unconverted **2** (Figure 3, bottom). Fitting of the major species reveals parameters that are quite similar to **1** and **2**, with *g*-values of 2.02, 2.10, and 2.56 and hyperfine coupling (MHz) to both ^59^Co (*A_xx_* = 54.8, *A_yy_* = 61.0, *A_zz_* = 66.8) and ^14^N (*A_xx_* = 23.1.645, *A_yy_* = 24.5, *A_zz_* = −14.1). Importantly, the EPR spectrum of **3** is less consistent with a Co–H assignment; inclusion of any significant hyperfine coupling to ^1^H notably worsens the fit. The Co K-edge XAS data for **3** has the same 0.4 eV shift to lower energy versus **1**, which again supports a very similar Co oxidation state across these three compounds (Figures S30 and S31).

We then turned to verifying the presence and investigating the localization of the added protons from hydrogenation. Hydrogenated and deuterated samples were prepared at −25 **°**C in minimal toluene over 48 or 72 h under ∼3 atm of H_2_ or D_2_, respectively. These samples were kept cold throughout drying and sample preparation. Both mineral oil and thin-film IR samples reproducibly show a clear N–D stretch in the deuterated samples around 2065 cm^–1^ (Figure 4, Table S63 and eq S1). The corresponding isotopically shifted feature is not immediately apparent in the spectrum of proteo-**3** due to convolution with C–H stretches, but a subtraction spectrum does indicate the presence of a feature underneath these other stretches at ∼2870 cm^–1^ that is consistent with a N–H functionality and which closely corresponds to the DFT-predicted N–H stretch of 2922 cm^–1^ (Table S63). This result confirms that the reactivity with H_2_ involves the formation of N–H’s on the ^*t*Bu,Tol^DHP ligand. As a final verification of the assigned structure of **3**, time-dependent DFT (TD-DFT) calculations were performed to compare the predicted and experimental UV–visible (UV–vis) spectra of **3**. The theoretical UV–vis spectrum of **3** using the PBE0 functional on the previously optimized geometry matches well with the experimental spectrum, further supporting our assignment (Figure S87).

### Hydrogenation Catalysis

Given the reactivity observed with dihydrogen to form **3**, we wanted to test if **2** could be used as a hydrogenation catalyst. Given its use in the synthesis of **1**, we initially chose 1-hexene as a test substrate under mild conditions (1 atm of H_2_, 23 **°**C). We observed 79(6)% conversion to hexane with 1% catalyst loading of **2** under these conditions, and thus we proceeded to investigate the scope of this reaction with other olefins (Table 2). Terminal mono-substituted olefins are all efficiently hydrogenated, even with comparatively large substrates; 3,3-dimethylbutene is hydrogenated in 71(2)% yield. Alkynes can also be reduced; hydrogenation of 1-hexyne with **2** provides hexane with 69(1)% yield and only 6(3)% of the singly hydrogenated product 1-hexene. A more moderate yield of 55(4)% is obtained with styrene.

**Table 2:**
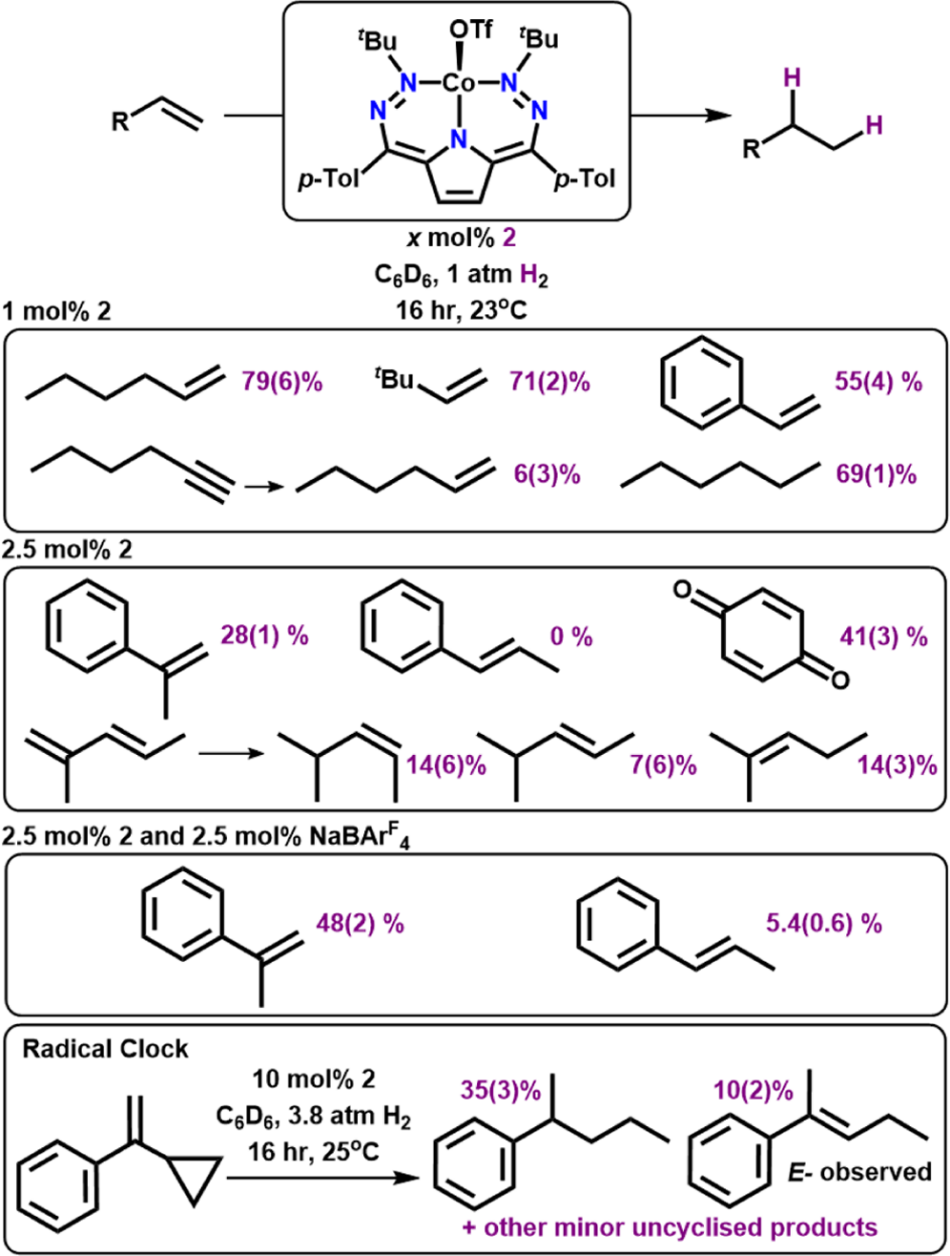
Hydrogenation Substrate Scope with **2**

**Scheme 2:**
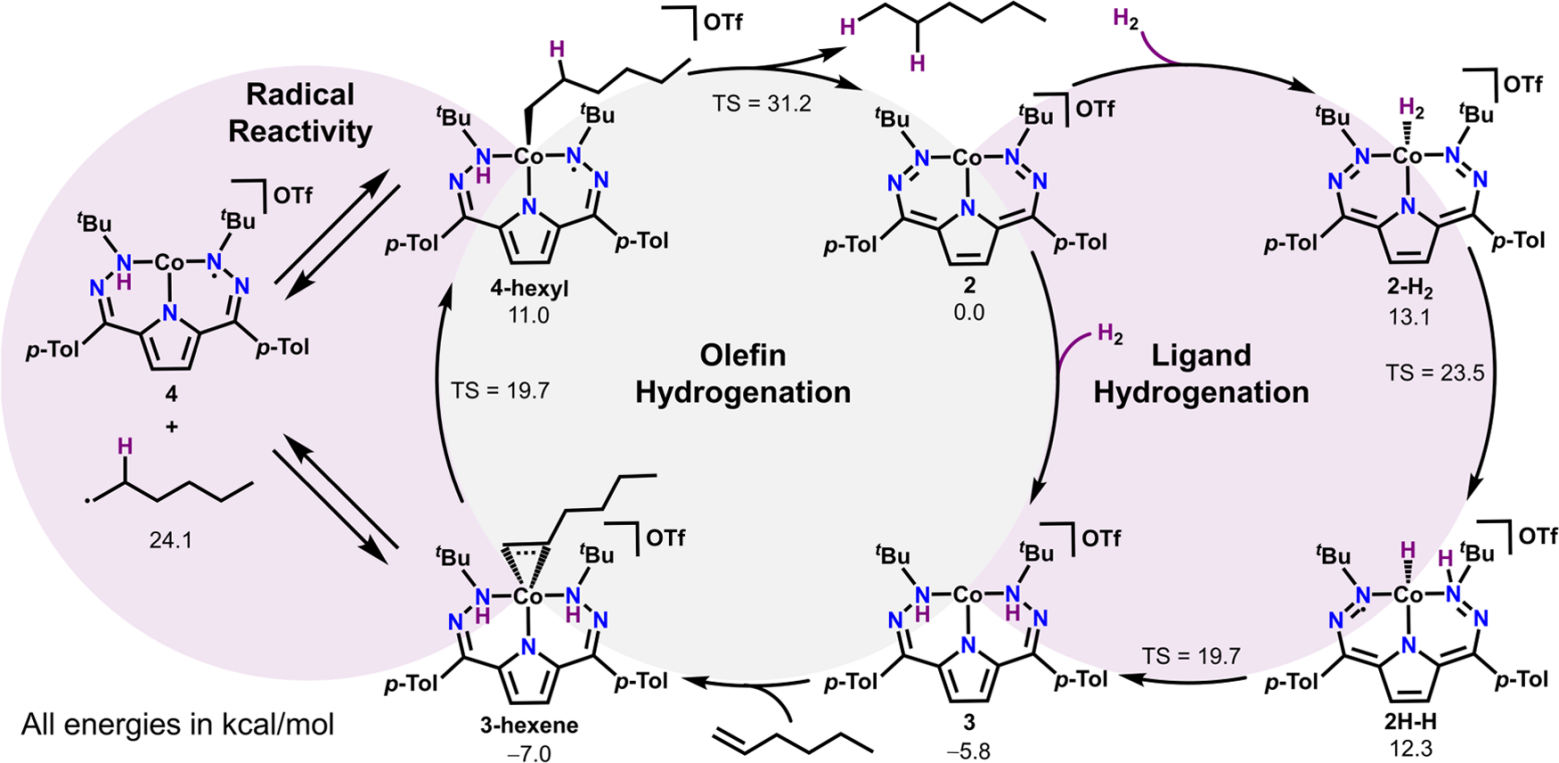
Mechanism for the Synthesis of **3** as well as for the Hydrogenations of Olefins (1-Hexene Used as Model Substrate)

Significantly attenuated yields are observed with more sterically encumbering substrates, such as α-methyl styrene, which is hydrogenated in only 28(1)% yield, even with a higher 2.5% catalyst loading. This suggests that steric limitations play a major role in catalysis by **2**, which is perhaps unsurprising given the large *t*-Bu substituents on the DHP ligand. Reactivity is shut down completely with β-methyl styrene even at 2.5% catalyst loadings. Based on mechanistic DFT calculations (see below), we decided to investigate whether OTf^–^-abstracting reagents might enable higher conversions with these sterically encumbering substrates. In situ addition of NaBAr_4_^F^ (BAr_4_^F^ = tetrakis[3,5-bis(trifluoromethyl)phenyl]borate) as an OTf^–^ abstractor to the catalytic mixture approximately doubles the yield of cumene from α-methyl styrene to 48(2)% and furthermore enables detectable hydrogenation yields (∼5%) for β-methyl styrene. Conversely, the addition of LiOTf to catalytic reactions with α-methyl styrene lowers the yield to 5.8%. These results suggest that the dissociation of triflate is likely important during catalysis and is consistent with DFT calculations that support lower energy pathways for the cationic fragment [(^*t*Bu,Tol^DHP)Co]^+^ (Scheme 2).

We also investigated the regioselectivity of hydrogenation with the substrate trans-2-methyl-1,3-pentene. Here, selectivity for the hydrogenation of the terminal, disubstituted olefin, is observed with ∼35% yield, consistent with the reactivity trends from mono-olefinic substrates. Interestingly, the hydrogenated products show cis/trans isomerization as well as migration of the internal double bond to the more thermodynamically favorable tri-substituted position. Given the limited hydrogenation reactivity with internal mono-olefins, we hypothesized that the isomerization of this substrate might be due to a radical pathway for hydrogenation and undertook mechanistic experiments to explore this possibility.

The hydrogenation of α-cyclopropyl styrene as a radical clock test substrate with 10% cat. loading of **2** showed exclusive cyclopropyl ring-opened products, as would be expected for a radical reaction.^26^ For this substrate, the major product is doubly hydrogenated *sec*-pentylbenzene in 35(3)% yield. We also observe the tri-substituted olefin product 2-phenylpent-2-ene in 10(2)% yield. This product is the expected intermediate olefin formed after ring-opening. Regardless of the exact product distribution, the absence of any hydrogenated products with an intact cyclopropyl ring strongly suggests a radical mechanism and also suggests related radical reactions to form the observed olefin migration products from trans-2-methyl-1,3-pentene.

The agency of radical reactivity in the hydrogenation catalysis of **2** is noteworthy, as Co-based hydrogenation catalysts frequently go through classical organometallic mechanisms featuring Co–H intermediates without radical reactivity.^14g^ Indeed, there has been recent interest in discovering Co catalysts with alternative mechanisms, in some cases switched with light.^14g^ The absence of any observable Co–H species upon hydrogenation of **2** and the observed radical reactivity in hydrogenation catalysis suggested to us the possibility of an unusual DHP ligand-promoted radical hydrogenation mechanism. While such a mechanism is supported by our experimental data, we also wanted to use DFT calculations to obtain a clearer picture of accessible pathways.

### Computational Analysis

All of the experimental data on **3** is consistent with the assignment of a hydrogenated ligand with a Co(II) center generated from the reaction of **2** with H_2_. This proposed assignment of **3** and other catalytically relevant intermediates was therefore investigated using DFT calculations (Scheme 2, Figures S56–S57, S64–S65, and S85–S86). Geometry optimizations and frequency calculations for postulated intermediates and transition states along two key cycles, addition of H_2_ to the DHP scaffold (Scheme 2 right) as well as 1-hexene hydrogenation (Scheme 2 middle), were calculated using the O3LYP functional and basis sets of def2-SVP on H, def2- TZVPP on Co, and def2-TZVP on N and C atoms (Scheme 2). Both doublet and quartet spin states were considered, and the results suggest that both spin states are relevant for catalysis.

The mechanism to form **3** was investigated both with OTf^–^ bound to Co and also with an outer sphere (unbound) OTf^–^. The transition state energies found for the outer sphere OTf^–^ pathway are generally lower in energy, sometimes significantly than for reactivity with OTf^–^ bound to Co. This observation is consistent with the enhanced yields we observe with added NaBAr_4_^F^ as a OTf^–^ abstractor. The initial hydrogenation of **2** is overall favorable with a free energy of −5.8 kcal/mol. The pathway to form this species goes first 13.1 kcal/mol uphill from **2** through an *S* = 1/2 Co-H_2_ adduct (**2-H**_**2**_). A high-energy transition state between **2-H**_**2**_ and an *S* = 1/2 Co hydride (**2H-H**) with a singly hydrogenated DHP ligand is 23.5 kcal/mol uphill from **2**. Intermediate **2H-H** can then proceed through a low-energy (7.4 kcal/mol versus **2H-H**) transition state to generate **3**.

The DFT-predicted pathway for olefin hydrogenation proceeds through roughly thermoneutral binding of 1-hexene to **3** (−1.2 kcal/mol) to form an *S* = 3/2 high-spin 1-hexene adduct (**3-hexene**). We then considered two branching pathways from this olefin adduct. Firstly, **3-hexene** could go through an ene-reaction-like transformation to generate a Co-alkyl product, **4-hexyl**. This reaction is unfavorable by 18.0 kcal/mol with a transition state 25.5 kcal/mol uphill in energy from **3-hexene**. We note that the depicted primary product is significantly lower in energy than the formal 2,1 insertion product, likely driven by the sterics of the *t*-Bu groups and thus possibly explaining the preference of the system for terminal olefins. Subsequent loss of alkane and regeneration of **2** proceeds through a high-energy transition state of 31.2 kcal/mol versus **2**. While this pathway is potentially feasible, the facile room-temperature activity of **2** and the observed radical reactivity also prompted us to evaluate separate radical intermediates.

Intermediate **3-hexene** may also transfer an H-atom to the bound 1-hexene to generate a hexyl radical and a singly hydrogenated Co complex **4**. This reaction to generate a terminal hexyl radical is 31.1 kcal/mol uphill in energy from **3-hexene** and is comparable in energy to the highest lying transition state for DHP hydrogenation (30.5 kcal/mol higher than **3-hexene**). The comparatively low energy of these free radical intermediates suggests a very weak Co–C bond in **4-hexyl**, and indeed, the loss of a primary hexyl radical from this intermediate is only 13.1 kcal/mol uphill in energy, and the formation of secondary radicals is even more accessible (Figures S85 and S86). As expected, subsequent H-atom abstraction from **4** by a hexyl radical is extremely favorable.

Without additional experimental details, which are difficult to obtain on this paramagnetic system, it is difficult to determine whether the hexene hydrogenation steps proceed exclusively through Co-bound species, such as **4-hexyl**, or if direct H-atom transfer to generate outer sphere carbon radicals dominates. We suspect that the mechanism features both alkyl radical and metal-alkyl species, which may explain the steric preferences for hydrogenation as well as the radical-derived products observed in mechanistic experiments. Furthermore, we note two additional mechanistic paradigms which deserve discussion. First, while we have limited our discussion to the above cationic pathway with an outer sphere OTf^–^, some of the putative intermediates with bound alkyl and OTf^–^ groups are slightly lower in energy, particularly for the later C–H bond forming steps, suggesting that additional ligation by counterions or solvent may facilitate cycles with bound alkyl substituents (Figures S56 and S57). Second, an additional pathway where intermediate **2H-H** directly binds and inserts olefin may be possible, although we think that the rate of olefin binding to **2H-H**, which would be a bimolecular process, is not likely to be competitive with the low barrier intramolecular reaction to proceed to **3**.

Nevertheless, the computed reaction pathways support the agency of the DHP ligand in hydrogenation and the absence of classic organometallic steps such as insertions from hydride intermediates. The calculations also suggest that improvements on yield or scope might be obtained by reducing the sterics on our catalyst and utilizing a more weakly coordinating anion than OTf^–^.

## Conclusions

In this study, we have synthesized a series of Co(II) complexes with the redox-active ^*t*Bu,Tol^DHP ligand scaffold. The CoOTf complex (**2**) forms a thermally unstable hydrogenated species (**3**) when exposed to dihydrogen, which can be characterized spectroscopically and computationally to confirm its assignment as a Co(II) complex with a hydrogenated DHP ligand. Complex **2** is also a competent catalyst for the hydrogenation of olefins at room temperature with 1 atmosphere of H_2_. Catalyst **2** selectively hydrogenates terminal alkenes, and it can also drive rearrangements of olefins into more thermodynamically favored products, likely through radical pathways. The agency of radical intermediates has been confirmed by the ring-opening of the radical clock α-cyclopropyl styrene in hydrogenation reactions. Our results demonstrate that the combination of ligand-based multi-proton and electron storage enables hydrogenation catalysis under mild conditions and, furthermore, that this ligand-centric paradigm enables alternative mechanistic pathways to more classic organometallic catalysts.

## Experimental Section

### General Methods

All chemicals were purchased from commercial suppliers and used without further purification. All manipulations were carried out under an atmosphere of N_2_ using standard Schlenk and glovebox techniques. Glassware was dried at 180 **°**C for a minimum of 2 h and cooled under vacuum prior to use. Solvents were dried on a solvent purification system from Pure Process Technology and stored over 4 Å molecular sieves under N_2_. Tetrahydrofuran (THF) was stirred over NaK alloy and run through an additional alumina column prior to use to ensure dryness. Solvents were tested for H_2_O and O_2_ using a standard solution of sodium-benzophenone ketyl radical anion. CD_3_CN, C_6_D_6_, and *d*_8_-toluene were dried over 4 Å molecular sieves under N_2_. α-cyclopropyl styrene was prepared following a previously reported procedure.^27^

^1^H and ^19^F NMR spectra were recorded on Bruker DRX 400 or 500 spectrometers. Chemical shifts are reported in ppm units referenced to residual solvent resonances for ^1^H spectra. UV–visible Spectra were recorded on a Bruker Evolution 300 spectrometer and analyzed using VisionPro software. A standard 1 cm quartz cuvette with an airtight screw cap with a puncturable Teflon seal was used for all measurements. A Unisoku CoolSpek cryostat was used for low-temperature measurements. ^1^H and ^19^F NMR spectra were recorded on either Bruker DRX 400 or AVANCE-500 spectrometers. IR spectra were obtained on a Bruker Tensor II spectrometer with the OPUS software suite. All IR samples were collected between KBr plates. EPR spectra were recorded on an Elexsys E500 Spectrometer with an Oxford ESR 900 X-band cryostat and a Bruker ColdEdge Stinger. EPR data was analyzed using the EasySpin MATLAB suite.^28^ Single-crystal X-ray diffraction data was collected in-house using Bruker D8 Venture diffractometer equipped with Mo microfocus X-ray tube (λ = 0.71073 Å).

X-ray near-edge absorption spectra (XANES) were employed to probe the local environment of Co. All sample preparation was performed under an inert atmosphere. Frozen solution samples were prepared by making a concentrated solution of the starting material in toluene (acetonitrile was added for solubility where indicated). This solution was then syringed into a precooled Teflon cuvette lined with Kapton tape in liquid nitrogen and then stored in liquid nitrogen until collection. Data was acquired at the Advanced Photon Source at Argonne National Labs with a bending magnet source with ring energy at 7.00 GeV. Co K-edge data was acquired at the MRCAT 10-BM beamline. The incident, transmitted, and reference X-ray intensities were monitored using gas ionization chambers. A metallic Co foil standard was used as a reference for energy calibration and was measured simultaneously with experimental samples. X-ray absorption spectra were collected at room temperature. Data collected was processed using the Demeter software suite, and Fityk was used for more precise pre-edge fitting.

### Co(^*t*Bu,Tol^DHP)Cl (1)

In a 20 mL vial in the glovebox, 1–2 mL of THF was added until the [^*t*Bu,Tol^DHP-H_4_][Cl]_2_ ligand sal^t^^9c^ (0.172 g, 1 equiv 0.333 mmol) dissolved completely as a yellow solution. A concentrated solution of KHMDS (0.186 g, 2.8 equiv, 0.932 mmol) in 1–2 mL THF was added dropwise with stirring. The solution turned from yellow to a bright red color, then darkened to a brownish green upon complete addition. After these color changes and additions were completed, CoCl_2_ (0.043 g, 1 equiv, 0.331 mmol), suspended in 1–2 mL of THF was added to the reaction mixture, which resulted in a color change to a brown-purple color. 1-hexene (41 μL, 1 equiv, 0.33 mmol) was added, with a resulting color change to a luminous magenta-purple. Shortly after the addition of 1-hexene, the reaction mixture was dried under vacuum to provide a purple solid. This solid was extracted with copious amounts of petroleum ether (50–60 mL). After drying this solution, **1** was obtained as a magenta-purple solid. Yield: 0.134 g, 75%. Single crystals for XRD were grown via cooling a petroleum ether solution at −35 **°**C. ^1^H NMR (400 MHz, C_6_D_6_, RT): δ = no signals. Magnetic susceptibility: Evans’ method (C_6_D_6_, RT, μ_B_): μ_eff_ = 1.74. UV–vis, nm in benzene, (ε, M^–1^ cm^–1^): 553 (3900). Anal. Calcd C, 62.86; H, 6.41; N, 13.09; found: C, 62.58; H, 5.97; N, 11.46. HRMS (EI) *m*/*z*: [M]^+^ calcd for **1**: C_28_H_34_N_5_ClCo 534.1835; found, 534.184.

### Co(^*t*Bu,Tol^DHP)OTf (2)

In a 20 mL vial in the glovebox, 2 mL of benzene was added to Co(^Tol,tBu^DHP)Cl **(1)** (0.060 g, 1 equiv, 0.11 mmol). A solution of silver triflate (0.028 g, 1 equiv, 0.11 mmol) in a mixture of 1:1 benzene/acetonitrile (2 mL) was added to the bright purple solution of **1**. The reaction was stirred for 1 h, over which time its color changed from emerald green immediately after addition to an olive color with concomitant formation of gray solids on the sides of the vial. This reaction mixture was dried under vacuum, after which the product was extracted with 10–20 mL of diethyl ether. Yield: 0.065 g, 89%. Single crystals suitable for XRD of **2** were grown out of a concentrated petroleum ether solution at −35 **°**C. ^1^H NMR (400 MHz, C_6_D_6_, RT): δ = 24.20 (bs), 10.17 (s), 9.07 (bs), 7.36 (s), 6.96(s), 6.87(bs), 4.06 (s). Magnetic susceptibility: Evans’ method for **2** (C_6_D_6_, RT, μ_B_): μ_eff_ = 1.71, UV–vis, nm in toluene, (ε, M^–1^cm^–1^): 516 (3700). Anal. Calcd C, 53.70; H, 5.28; N, 10.80; found, 54.32, 5.55, 10.31. HRMS (EI) *m*/*z*: [M]^+^ cald for **2**: C_29_H_34_N_5_O_3_F_3_S Co 648.1666; found, 648.1665.

### Reactivity with H_2_

A 100 mL Schlenk flask with 8 mg of **2** with 50–100 μL of toluene was prepared in the glovebox. This solution was frozen in liquid nitrogen, and the headspace was evacuated under vacuum. The flask was then backfilled with 1 atm of H_2_, which is equivalent to ∼3.8 atm of H_2_ at room temperature. The flask was then relocated into a freezer at −25 **°**C, where it was allowed to react for 30–36 h without stirring. Upon completion of the reaction with H_2_, the reddish-purple color of **2** converts to a pinker purple, indicating the formation of **3**. Complex **3** is stable to vacuum and is relatively stable as a solid to air but decomposes rapidly if exposed to air in the solution state. This complex is relatively stable below 0 **°**C, but slow decomposition occurs at this temperature and above. To characterize this product, the reaction vessel was pumped back into the nitrogen-filled glovebox and placed into a −35 **°**C freezer. The cold solution was then dried rapidly under vacuum and then analyzed by various techniques, as described below. IR (Nujol mull between KBr plates, cm^–1^): 3180 (N–H, w), 3170 (N–H, w), 1641 (s).

### Preparation of IR samples of 3

#### Nujol Mull

Complex **3** (8 mg), prepared in the method described above, was mixed in a cold mortar and pestle with minimal nujol to form a mustard-like suspension. This mixture was dolloped on a cooled KBr plate, and a second plate was placed on top. The sample was then transferred in an air-free temporary container to the spectrometer, and a spectrum was collected.

#### Thin Film on KBr Plate

Complex **3** (8 mg), prepared in the method described above, was dissolved in cold, dry diethyl ether to form a concentrated solution. This was dropped on a cooled KBr plate, and a second plate was placed on top. The sample was then transferred in an air-free temporary container to the spectrometer, and a spectrum was collected.

### General Catalytic Hydrogenation Procedures and Products

#### Procedure for 1% Loading

In a nitrogen-filled glovebox, a 250 mL Schlenk flask with a magnetic stir bar was charged with unsaturated substrate (0.077 mmol, 100 equiv), **2** (0.0005 g, 0.0008 mmol), mesitylene (0.002 mL, 0.014 mmol) (internal standard), and benzene-*d*_6_ (0.1 mL). On a Schlenk line, the solution was freeze–pump–thaw-degassed and warmed to room temperature with the contents under static vacuum. At room temperature, this vessel was backfilled with 1 atm H_2_ gas. The vessel was then sealed and left to stir for 18 h. The dark red-purple **1** could be observed to pinken within the hour, turn greenish-red, and then begin to turn orange/yellow after 6 h. After 18 h, the vessel was shipped back into the nitrogen-filled glovebox and diluted to 0.7 mL total volume. This was then analyzed via ^1^H and ^19^F NMR, and checked by GC-MS as needed.

#### Procedure for 2.5% Loading

The general method described above was implemented with 0.031 mmol, 40 equiv of unsaturated solvent used.

#### Procedure for 2.5% Loading with NaBAr_4_^F^

The general method described above was implemented with 0.031 mmol, 40 equiv of unsaturated solvent used and with the addition of 0.0008 mmol, 1 equiv of NaBAr_4_^F^ pre-added to the reaction vessel with 0.07 mL of THF.

#### Procedure for 10% Loading

In a nitrogen-filled glovebox, a 250 mL Schlenk flask with a magnetic stir bar was charged with unsaturated substrate (0.0077 mmol, 10 equiv), **2** (0.0005 g, 0.0008 mmol), mesitylene (0.002 mL, 0.014 mmol) (internal standard), and benzene-*d*_6_ (0.1 mL). On a Schlenk line, the solution was freeze–pump–thaw-degassed and backfilled at 77 K with 3.8 atm H_2_ gas. The vessel was then sealed and left to stir for 18 h. After 18 h, the vessel was shipped back into the nitrogen-filled glovebox and diluted to 0.7 mL total volume. This was then analyzed via ^1^H and ^19^F NMR and checked by GC-MS as needed.

## Supporting Information Available

Experimental procedures, NMR, IR, GC-MS UV–vis, EPR, XAS, SXRD data, and DFT (PDF)
Crystallographic Data 1 (CIF)
Crystallographic Data 2 (CIF)

(PDF)
(CIF)
(CIF)

## Supplementary Material

cs2c02467_si_001.pdf

cs2c02467_si_002.cif

cs2c02467_si_003.cif

## Accession Codes

CCDC 2173743 and 2173744 contain the supplementary crystallographic data for this paper. This data can be obtained free of charge via www.ccdc.cam.ac.uk/data_request/cif, or by emailing to data_request@ccdc.cam.ac.uk, or by contacting The Cambridge Crystallographic Data Centre, 12 Union Road, Cambridge CB2 1EZ, UK; fax: +44 1223 336033
