# Supplementary material for: Cobalt-Catalyzed Hydrogenation Reactions Enabled by Ligand-Based Storage of Dihydrogen
Source: ACS Catal. 2022 Aug 1;12(16):9933–43. doi: 10.1021/acscatal.2c02467 (PMC9396622; doi:10.1021/acscatal.2c02467)
Supplement: Supplementary file 1 — cs2c02467_si_001.pdf [file cs2c02467_si_001.pdf]

Supplementary Information for:

Cobalt-Catalyzed Hydrogenation Reactions Enabled by Ligand-Based Storage of Dihydrogen

Sophie W. Anferov, Alexander S. Filatov, John S. Anderson*

Department of Chemistry

The University of Chicago, Chicago, Illinois 60637, United States.

*corresponding author: jsanderson@uchicago.edu

Contents

General Methods 10

Co($^{tBu, Tol}$DHP)Cl (**1**) 10

Co($^{tBu, Tol}$DHP)OTf (**2**) 11

Reactivity with H_2 11

Preparation of IR samples of **3** 11

Nujol Mull 12

Thin film on KBr plate 12

NMR spectroscopy 12

Figure S1. ^1H NMR of **2** in C_6D_6. Residual solvent or grease marked with stars. 12

Figure S2. ^1H NMR of **3** in C_6D_6 13

UV-vis spectroscopy 13

Figure S3. UV-vis of **1** from a 0.31 mM solution of **1** in toluene. 14

Figure S4. UV-vis of **2** from a 0.87 mM solution of **2** in benzene. 14

Figure S5. UV-vis of **3** from a solution in toluene at -35°C 14

Figure S6. UV-vis of **3** in toluene at 0°C over first 4.5 hours scans every 4.5 minutes 15

Figure S7. UV-vis of **3** in toluene at 0 °C for second 10.5 hours (starting at 4.5 hours), scans every 31.5 minutes 15

Figure S8. UV-vis of **2** + H_2 from a 0.24 mM solution of **2** in toluene (RT, scans every 13 minutes, 21 hours). 16

Vibrational Spectroscopy 16

Figure S9. IR of nujol. 16

Figure S10. IR of **1** in nujol. 17

Figure S11. IR of **2** in nujol. 17

Figure S12. IR of **3** (black) and **3-D$_2$** (blue) in nujol. Star indicates the N–D stretch. 18

Figure S13. IR Difference Spectrum of **3** (black) and **3-D$_2$** (blue) in nujol. Star indicates the N–D stretch. 18

Figure S14. Zoomed IR with Difference Spectrum inset of **3** (black) and **3-D$_2$** (blue) in nujol. Star indicates the N–D stretch. 19

Figure S15. IR of nujol (orange) and **2** (purple), **3** (black) and **3-D$_2$** (blue) in nujol. Star indicates the N–D stretch. 19

Figure S16. IR of **3** (black) and **3-D$_2$** (blue) as a thin film on KBr. Blue star indicates the N–D stretch. Gray star indicates the proposed position of the N–H stretch. 20

Figure S17. IR Difference Spectrum of **3** (black) and **3-D$_2$** (blue) as a thin film on KBr. Blue star indicates the N–D stretch. Gray star indicates the proposed position of the N–H stretch. 20

Figure S18. Zoomed IR with Difference Spectrum inset of **3** (black) and **3-D$_2$** (blue) as a thin film on KBr. Blue star indicates the N–D stretch. Gray star indicates the proposed position of the N–H stretch. 21

Cyclic Voltammetry 21

Figure S19. Cyclic Voltammogram of **1**. 21

Figure S20. Cyclic Voltammogram of **2**. 22

EPR Spectroscopy 22

Figure S21. Full perpendicular-mode EPR spectrum of a 15 mM solution of **1** in toluene at 17 K. Some small unknown impurity peaks are marked. Conditions: MW frequency, 9.631 GHz; MW power, 2.0 mW 22

Figure S22. Small Window perpendicular-mode EPR spectrum (left) and simulated spectrum (right) of a 15 mM solution of **1** in toluene at 15 K. Conditions: MW frequency, 9.631 GHz; MW power, 2.0 mW 23

Figure S23. Full perpendicular-mode EPR spectrum of a 15 mM solution of **2** in toluene at 15 K. Conditions: MW frequency, 9.631 GHz; MW power, 2.0 mW. 23

Figure S24. Small Window perpendicular-mode EPR spectrum (left) and simulated spectrum (right) of a 15 mM solution of **2** in toluene at 15 K. Conditions: MW frequency, 9.631 GHz; MW power, 2.0 mW 24

Figure S25. Full perpendicular-mode EPR spectrum of a 15 mM solution of **3** in toluene at 20 K. Conditions: MW frequency, 9.631 GHz; MW power, 2.0 mW. 24

Figure S26. Small Window perpendicular-mode EPR spectrum (left) and simulated spectrum (right) of a 15 mM solution of **3** in toluene at 20 K. Conditions: MW frequency, 9.631 GHz; MW power, 2.0 mW 25

Figure S27. Small Window perpendicular-mode EPR spectrum and simulated spectrum overlay of a 15 mM solution of **3** in toluene at 20 K with 9.0% 2 impurity included. Conditions: MW frequency, 9.631 GHz; MW power, 2.0 mW. 25

Table S1. *g*-values for EPR 25

Code to generate simulations of EPR Spectra 26

1: 26

2: 26

3: 26

X-ray Absorption Spectroscopy 26

Figure S28. X-ray absorption spectra of **1** (red) and **2** (blue) 27

Figure S29. Derivative of X-ray absorption spectra of **1** (red) and **2** (blue) 27

Figure S30. X-ray absorption spectra of **1** (blue), **2** (red) and **3-MeCN** (green) 28

Figure S31. Derivative of X-ray absorption spectra of **1** (blue), **2** (red) and **3-MeCN** (green) 28
Single Crystal X-ray Diffraction 29
X-Ray Structure Determination. 29
Figure S32. SXRD of **1**. Co (pink), N (blue), C (gray), Cl (green), H-atoms omitted. 29
Table S2. SXRD of **1**. 29
Figure S33. SXRD of **2** (polymeric structure bridged by triflates). Co (pink), N (blue), C (gray), F (lime green), O (red), S (yellow), H-atoms omitted. 30
Table S3. SXRD of **2**. 31
Density Functional Theory (DFT) 31
Geometry Optimizations 31
Figure 34. Calculated structure of **1**. All C–H hydrogen atoms have been removed for clarity. 32
Table S4. Calculated coordinates of **1**. 32
Figure S35. Spin density plot of **1** at an iso value of 0.003. 35
Figure S36. Calculated structure of **2**. All C–H hydrogen atoms have been removed for clarity 36
Table S5. Calculated coordinates of **2**. 36
Figure S37. Spin density plot of **2** at an iso value of 0.005. 39
Figure S38. Calculated structure of [$^{tBu,\ Tol}$DHPCo]$^+$ (**2**$^+$). All C–H hydrogen atoms have been removed for clarity. 40
Table S6. Calculated coordinates of [$^{tBu,\ Tol}$DHPCo]$^+$ (**2**$^+$). 40
Figure S39. Calculated structure of **3**-HS. 43
Table S7. Calculated coordinates of **3**-HS 43
Figure S40. Calculated structure of **3**-LS. 46
Table S8. Calculated coordinates of **3**-LS 46
Figure S41. Spin density plot of **3**-HS at an iso value of 0.005. 49
Figure S42. Spin density plot of **3**-LS at an iso value of 0.005. 50
Figure S43. Calculated structure of [$^{tBu,\ Tol}$DHP-H_2Co]$^+$-HS (**2-H$_2^+$**). 50
Figure S44. Calculated structure of [$^{tBu,\ Tol}$DHP-H_2Co]$^+$-LS (**2-H$_2^+$**). 51
Table S9. Calculated coordinates of [$^{tBu,\ Tol}$DHP-H_2Co]$^+$ -HS (**2-H$_2^+$**) 51
Table S10. Calculated coordinates of [$^{tBu,\ Tol}$DHP-H_2Co]$^+$-LS (**2-H$_2^+$**) 53
Figure S45. Calculated structure of a high spin **3-hexene** 56
Figure S46. Calculated structure of a low spin **3-hexene**. 57
Figure S47. Calculated structure of a high spin **3-hexene**$^+$ 57
Figure S48. Calculated structure of a low spin **3-hexene**$^+$ 58

Figure S49. Calculated structure of a high spin **4-hexyl-α**. 58
Figure S50. Calculated structure of a low spin **4-hexyl-α**. 59
Figure S51. Calculated structure of a high spin **4-hexyl-β**. 59
Figure S52. Calculated structure of a low spin **4-hexyl-β**. 60
Figure S53. Calculated structure of a high spin **4-hexyl-β⁺** 60
Figure S54. Calculated structure of a low spin **4-hexyl-β⁺** 61
Table S11. Calculated coordinates of **3-hexene**-LS 61
Table S12. Calculated coordinates of **3-hexene**-HS 64
Table S13. Calculated coordinates of **3-hexene⁺**-HS 68
Table S14. Calculated coordinates of **3-hexene⁺**-LS 71
Table S15. Calculated coordinates of **4-hexyl-α**-LS 74
Table S16. Calculated coordinates of **4-hexyl-α**-HS 77
Table S17. Calculated coordinates of **4-hexyl-β**-LS 81
Table S18. Calculated coordinates of **4-hexyl-β**-HS 84
Table S19. Calculated coordinates of **4-hexyl-β⁺**-LS 88
Table S20. Calculated coordinates of **4-hexyl-β⁺**- HS 91
Table S21. Compared single point energies of intermediates along catalytic cycle for 1-hexene hydrogenation without accounting for entropic contributions. 94
Figure S55. Simplified catalytic steps towards 1-hexene hydrogenation with the beta-carbon being the first to be hydrogenated (from single point energies, without accounting for entropic contributions). 95
Table S22. Compared Gibbs free energies of intermediates along catalytic cycle for 1-hexene hydrogenation with accounting for entropic contributions. 95
Figure S56. Simplified catalytic steps towards 1-hexene hydrogenation with the beta-carbon being the first to be hydrogenated (accounting for entropic contributions). 96
Figure S57. Simplified catalytic steps towards 1-hexene hydrogenation with the beta-carbon being the first to be hydrogenated without triflate (accounting for entropic contributions). 96
Table S23. Compared Gibbs free energies of intermediates along catalytic cycle for 1-hexene hydrogenation without triflate bound with accounting for entropic contributions. 96
Figure S58. Spin density plot of **3-hexene** at an iso value of 0.005. 97
Figure S59. Spin density plot of **4-hexyl-β** at an iso value of 0.005 97
Figure S60. Calculated Structures of Hexanes, 1-hexene and H_2. 98
Table S24. Calculated coordinates of hexanes 98
Table S25. Calculated Coordinates of 1-hexene 98
Table S26. Calculated Coordinates of H_2 99

Figure S61. Energetically Compared 2-methyl-pent-1,3-ene Radical Isomer Structures. 100

Table S27. Compared Gibbs free energies of 2-methyl-pent-1,3-ene radical isomers 100

Figure S62. Energetically compared 2-methyl-pent-1,3-ene non-radical isomer structures 100

Table S28. Compared Gibbs free energies of 2-methyl-pent-1,3-ene non-radical isomers 100

Table S29. Calculated coordinates of 2-methyl-pent-1,3-ene radical byproduct isomers: primary radical structure 100

Table S30. Calculated coordinates of 2-methyl-pent-1,3-ene radical byproduct isomers: secondary radical structure 101

Table S31. Calculated coordinates of 2-methyl-pent-1,3-ene radical byproduct isomers: tertiary radical structure 102

Table S32. Calculated coordinates of 2-methyl-pent-1,3-ene starting material and product isomers: 2-methyl-pent-1,3-ene 102

Table S33. Calculated coordinates of 2-methyl-pent-1,3-ene starting material and product isomers: 2-methyl-pentane 103

Table S34. Calculated coordinates of 2-methyl-pent-1,3-ene starting material and product isomers: 2-methyl-pent-2-ene 104

Table S35. Calculated coordinates of 2-methyl-pent-1,3-ene starting material and product isomers: (*E*)-4-methylpent-2-ene 105

Table S36. Calculated coordinates of 2-methyl-pent-1,3-ene starting material and product isomers: (*Z*)-4- methylpent-2-ene 105

Table S37. Calculated coordinates of 2-methyl-pent-1,3-ene starting material and product isomers: 2-methylpent-1-ene 106

Table S38. Compared single point energies of intermediates towards ligand hydrogenation without accounting for entropic contributions. 107

Figure S63. Simplified catalytic steps towards ligand hydrogenation (without accounting for entropic contributions). 107

Table S39. Compared Gibbs free energies of intermediates along catalytic cycle for ligand hydrogenation with accounting for entropic contributions. 107

Figure S64. Simplified catalytic steps towards ligand-centered dihydrogen reactivity with triflate bound (accounting for entropic contributions)... 108

Figure S65. Simplified catalytic steps towards ligand-centered dihydrogen reactivity with the triflate anion outer-sphere (accounting for entropic contributions). 108

Figure S66. Calculated structure of high spin [($^{tBu, Tol}$DHP-H)CoH]OTf (**2H-H**). All C–H hydrogen atoms have been removed for clarity. 109

Figure S67. Calculated structure of low spin [($^{tBu, Tol}$DHP-H)CoH]OTf (**2H-H**). All C–H hydrogen atoms have been removed for clarity. 109

Figure S68. Calculated structure of high spin [($^{t\text{Bu, Tol}}$DHP -H)CoH]$^+$ (**2H-H$^+$**). All C–H hydrogen atoms have been removed for clarity. 110

Figure S69. Calculated structure of low spin [($^{t\text{Bu, Tol}}$DHP -H)CoH]$^+$ (**2H-H$^+$**). All C–H hydrogen atoms have been removed for clarity. 110

Table S40. Calculated coordinates of high spin [($^{t\text{Bu, Tol}}$DHP -H)CoH]OTf (**2H-H**) 110

Table S41. Calculated coordinates of low spin [($^{t\text{Bu, Tol}}$DHP -H)CoH]OTf (**2H-H**) 113

Table S42. Calculated coordinates of high spin [($^{t\text{Bu, Tol}}$DHP -H)CoH]$^+$ (**2H-H$^+$**). 116

Table S43. Calculated coordinates of low spin [($^{t\text{Bu, Tol}}$DHP -H)CoH]$^+$ (**2H-H$^+$**). 119

Figure 70. Calculated structure of low spin [($^{t\text{Bu, Tol}}$DHP)CoH$_2$]OTf (**2-H$_2$**). All C–H hydrogen atoms have been removed for clarity. 122

Figure S71. Calculated structure of high spin [($^{t\text{Bu, Tol}}$DHP)CoH$_2$]$^+$ (**2-H$_2$$^+$**). All C–H hydrogen atoms have been removed for clarity. 122

Figure S72. Calculated structure of low spin [($^{t\text{Bu, Tol}}$DHP)CoH$_2$]$^+$ (**2-H$_2$$^+$**). All C–H hydrogen atoms have been removed for clarity. 123

Table S44. Calculated coordinates of low spin [($^{t\text{Bu, Tol}}$DHP)CoH$_2$]OTf (**2-H$_2$**) (no optimized geometry for a high spin isomer could be found). 123

Table S45. Calculated coordinates of high spin [($^{t\text{Bu, Tol}}$DHP)CoH$_2$]$^+$ (**2-H$_2$$^+$**). 126

Table S46. Calculated coordinates of low spin [($^{t\text{Bu, Tol}}$DHP)CoH$_2$]$^+$ (**2-H$_2$$^+$**). 128

Figure S73. Spin density plot of **2-H$_2$** at an iso value of 0.005. 131

Figure S74. Spin density plot of **2H-H** at an iso value of 0.005. 132

Figure S75. Transition state structure between low spin [($^{t\text{Bu, Tol}}$DHP)CoH$_2$]OTf (**2-H$_2$**) and [($^{t\text{Bu, Tol}}$DHP -H)CoH]OTf (**2H-H**) and the triflate-free transition state. 132

Table S47. Calculated coordinates of transition state structures between low spin [($^{t\text{Bu, Tol}}$DHP)CoH$_2$]OTf (**2-H$_2$**) and [($^{t\text{Bu, Tol}}$DHP -H)CoH]OTf (**2H-H**). 132

Table S48. Calculated coordinates of transition state structures between low spin [($^{t\text{Bu, Tol}}$DHP)CoH$_2$]$^+$ (**2-H$_2$$^+$**) and [($^{t\text{Bu, Tol}}$DHP -H)CoH]$^+$ (**2H-H$^+$**) 135

Figure S76. Transition state structure between low spin [($^{t\text{Bu, Tol}}$DHP -H)CoH]OTf (**2H-H**) and **3** and triflate-free version. 138

Table S49. Calculated coordinates of transition state structure between low spin [($^{t\text{Bu, Tol}}$DHP -H)CoH]OTf (**2H-H**) and **3**. 138

Table S50. Calculated coordinates of transition state structure between low spin [($^{t\text{Bu, Tol}}$DHP -H)CoH]$^+$ (**2H-H$^+$**) and **3$^+$**. 141

Figure S77. Transition state structure between high spin [($^{t\text{Bu, Tol}}$DHP -H2)Co](1-hexene)OTf (**3-hexene**) and low spin [($^{t\text{Bu, Tol}}$DHP -H)Co](hex)OTf (**4-hexyl-β**) (and triflate free version). 144

Table S51. Calculated coordinates of transition state structure between high spin [($^{t\text{Bu, Tol}}$DHP -H$_2$)Co](1-hexene)OTf (**3-hexene**) and [($^{t\text{Bu, Tol}}$DHP -H)Co](hex)OTf (**4-hexyl-β**). 144

Table S52. Calculated coordinates of transition state structure between high spin [($^{tBu, Tol}$DHP -H$_2$)Co](1-hexene)$^+$ (**3-hexene$^+$**) and [($^{tBu, Tol}$DHP -H)Co](hex)$^+$ (**4-hexyl-β$^+$**). 148

Figure S78. Transition state structure between low spin [($^{tBu, Tol}$DHP -H)Co](hex)OTf (**4-hexyl-β**) and [($^{tBu, Tol}$DHP)Co]OTf/hexanes (**2**) and triflate-free version. 151

Table S53. Calculated coordinates of transition state structure between low spin [($^{tBu, Tol}$DHP -H)Co](hex) OTf (**4-hexyl-β**) and [($^{tBu, Tol}$DHP)Co]OTf/hexanes (**2**) 151

Table S54. Calculated coordinates of transition state structure between low spin [($^{tBu, Tol}$DHP -H)Co](hex)$^+$ (**4-hexyl-β$^+$**) and [($^{tBu, Tol}$DHP)Co]$^+$ /hexanes (**2$^+$**) 155

Figure S79. Calculated structure of high spin [($^{tBu, Tol}$DHP -H)Co]OTf (**4**-HS) 158

Figure S80. Spin density plot of **4**-HS at an iso value of 0.005. 159

Figure S81. Calculated structure of low spin [($^{tBu, Tol}$DHP -H)Co]OTf (**4**-LS) 159

Table S55. Calculated coordinates of high spin [($^{tBu, Tol}$DHP -H)Co]OTf (**4**-HS). 159

Table S56. Calculated coordinates of low spin [($^{tBu, Tol}$DHP -H)Co]OTf (**4**-LS). 162

Figure S82. Calculated Structure of high spin [($^{tBu, Tol}$DHP -H)Co] (**4$^+$**-LS) 165

Figure S83. Calculated structure of low spin [($^{tBu, Tol}$DHP -H)Co] (**4$^+$**-LS) 166

Table S57. Calculated coordinates of high spin [($^{tBu, Tol}$DHP -H)Co] (**4$^+$**-HS) 166

Table S58. Calculated coordinates of low spin [($^{tBu, Tol}$DHP -H)Co] (**4$^+$**-LS). 168

Figure S84. Calculated structures of 1-hexene primary and secondary radicals 171

Table S59. Calculated Coordinates of 1-hexene primary radical 171

Table S60. Calculated coordinates of 1-hexene secondary radical. 172

Table S61. Compared energies of radical-derived intermediates along catalytic cycle for 1-hexene hydrogenation without accounting for entropic contributions. 173

Table S62. Compared Gibbs free energies of radical-derived intermediates along catalytic cycle for 1-hexene hydrogenation with accounting for entropic contributions. 173

Figure S85. Simplified catalytic steps with radical pathways towards 1-hexene hydrogenation (accounting for entropic contributions) 173

Figure S86. Simplified catalytic steps with radical pathways towards 1-hexene hydrogenation without triflate (accounting for entropic contributions). 174

Figure S87. TDDFT of **3**. 174

Table S63. Calculated vs. experimental values. 174

Equation S1. IR Verification Calculations 175

Mass Spectrometry 176

Figure S88. HRMS of **3**. 176

General Catalytic Hydrogenation Procedures and Products 176

Procedure for 1% loading: 176

Procedure for 2.5% loading: 176
Procedure for 2.5% loading with $NaBAr^F_4$: 176
Procedure for 10% loading: 176
1-Hexene: 177
Hexane: 177
1-Hexyne: 177
Hexane and 1-Hexene: 177
Styrene: 177
Ethyl benzene: 177
3,3-Dimethylbutene: 177
2,2-Dimethylbutane: 177
α-Methyl styrene: 177
Isopropyl benzene (cumene): 178
β-Methyl styrene: 178
n-Propyl benzene: 178
Benzoquinone: 178
Hydroquinone: 178
2-methyl-pent-1,3-ene: 178
4-methyl-2-pentene (cis- and trans-) and 2-methyl-pent-2-ene: 178
α-cyclopropyl styrene: 179
Pentan-2-ylbenzene and (E)-pent-2-en-2-ylbenzene: 179
Controls 179
Figure S89. Controls with alternative cobalt catalyst 179
NMR data from Hydrogenations: Figures S90-100 179

General Methods

All chemicals were purchased from commercial suppliers and used without further purification. All manipulations were carried out under an atmosphere of N_2 using standard Schlenk and glovebox techniques. Glassware was dried at 180 °C for a minimum of two hours and cooled under vacuum prior to use. Solvents were dried on a solvent purification system from Pure Process Technologies and stored over 4 Å molecular sieves under N_2. Tetrahydrofuran (THF) was stirred over NaK alloy and run through an additional alumina column prior to use to ensure dryness. Solvents were tested for H_2O and O_2 using a standard solution of sodium-benzophenone ketyl radical anion. CD_3CN, C_6D_6, and d_8-toluene were dried over 4 Å molecular sieves under N_2. α−cyclopropyl styrene was prepared following a previously reported procedure.[1]

^{1}H and ^{19}F NMR spectra were recorded on Bruker DRX 400 or 500 spectrometers. Chemical shifts are reported in ppm units referenced to residual solvent resonances for ^{1}H spectra. UV-Visible Spectra were recorded on a Bruker Evolution 300 spectrometer and analyzed using VisionPro software. A standard 1 cm quartz cuvette with an airtight screw cap with a puncturable Teflon seal was used for all measurements. A Unisoku CoolSpek cryostat was used for low-temperature measurements. ^{1}H and ^{19}F NMR spectra were recorded on either Bruker DRX-400 or AVANCE-500 spectrometers. IR spectra were obtained on a Bruker Tensor II spectrometer with the OPUS software suite. All IR samples were collected between KBr plates. EPR spectra were recorded on an Elexsys E500 Spectrometer with an Oxford ESR 900 X-band cryostat and a Bruker Cold-Edge Stinger. EPR data was analyzed using the EasySpin Matlab suite.[2] Single crystal X-ray diffraction data were collected in-house using Bruker D8 Venture diffractometer equipped with Mo microfocus X-ray tube (λ = 0.71073 Å).

X-ray near-edge absorption spectra (XANES) were employed to probe the local environment of Co. Frozen solution samples were prepared by making a concentrated solution of the starting material in toluene (acetonitrile was added for solubility where indicated). This solution was then syringed into a pre-cooled Teflon window lined with Kapton tape in liquid nitrogen, then stored in liquid nitrogen until collection. Data were acquired at the Advanced Photon Source at Argonne National Labs with a bending magnet source with ring energy at 7.00 GeV. Co K-edge data were acquired at the MRCAT 10-BM beam line. The incident, transmitted and reference X-ray intensities were monitored using gas ionization chambers. A metallic Co foil standard was used as a reference for energy calibration and was measured simultaneously with experimental samples. X-ray absorption spectra were collected at room temperature. Data collected was processed using the Demeter software suite, and Fityk was used for more precise pre-edge fitting.

Co($^{tBu,\,Tol}$DHP)Cl (1)

In a 20 mL vial in the glovebox, 1-2 mL of THF was added until the [$^{tBu,\,Tol}$DHP-H_4][Cl]$_2$ ligand salt[3] (0.172 g, 1 eq. 0.333 mmol) dissolved completely as a yellow solution. A concentrated solution of KHMDS (0.186 g, 2.8 equiv., 0.932 mmol) in 1-2 mL THF was added dropwise with stirring. The solution turned from yellow to a bright red color, then darkened to a brownish green

upon complete addition. After these color changes and additions were completed, $CoCl_2$ (0.043 g, 1 eq., 0.331 mmol), suspended in 1-2 mL of THF was added to the reaction mixture which resulted in a color change to a brown-purple color. 1-hexene (41 μL, 1 eq., 0.33 mmol) was added with a resulting color change to a luminous magenta-purple. Shortly after the addition of 1-hexene, the reaction mixture was dried under vacuum to provide a purple solid. This solid was extracted with copious amounts of petroleum ether (50-60 mL). After drying this solution, **1** was obtained as a magenta-purple solid. Yield: 0.134 g, 75%. Single crystals for XRD were grown via cooling a petroleum ether solution at -35 °C. ^{1}H NMR (400 MHz, C_6D_6, RT): δ = no signals. Magnetic Susceptibility: Evans' Method (C_6D_6, RT, μ_B): μ_{eff} = 1.74; UV-vis, nm in benzene, (ε, $M^{-1}cm^{-1}$): 553 (3900). Anal. Calc. C, 62.86; H, 6.41; N, 13.09; Found: C, 62.58; H, 5.97; N, 11.46. HRMS (EI) m/z: $[M]^+$ calculated for **1**: $C_{28}H_{34}N_5ClCo$ 534.1835 found 534.184.

Co($^{tBu,\ Tol}$DHP)OTf (2)

In a 20 mL vial in the glovebox, 2 mL of benzene was added to Co($^{tBu,\ Tol}$DHP)Cl **(1)** (0.060 g, 1 eq., 0.11 mmol). A solution of silver triflate (0.028 g, 1 eq., 0.11 mmol) in a mixture of 1:1 benzene/acetonitrile (2 mL) was added to the bright purple solution of **1**. The reaction was stirred for 1 hour, over which time its color changed from emerald green immediately after addition to an olive color with concomitant formation of gray solids on the sides of the vial. This reaction mixture was dried under vacuum, after which the product was extracted with 10-20 mL of diethyl ether. Yield: 0.065 g, 89%. Single crystals suitable for XRD of **2** were grown out of a concentrated petroleum ether solution at −35 °C. ^{1}H NMR (400 MHz, C_6D_6, RT): δ = 24.20 (bs), 10.17 (s), 9.07 (bs), 7.36 (s), 6.96(s), 6.87(bs), 4.06 (s). ^{19}F NMR (400 MHz, C_6D_6, RT): δ = no signals. Magnetic Susceptibility: Evans' Method for **2** (C_6D_6, RT, μ_B): μ_{eff} = 1.71; UV-vis, nm in toluene, (ε, $M^{-1}cm^{-1}$): 516 (3700). Anal. Calc. C, 53.70; H, 5.28; N, 10.80; Found: 54.32, 5.55, 10.31. HRMS (EI) m/z: $[M]^+$ calculated for **2**: $C_{29}H_{34}N_5O_3F_3S$ Co 648.1666 found 648.1665.

Reactivity with H_2

A 100 mL Schlenk flask with 8 mg of **2** with 50-100 μL of toluene was prepared in the glovebox. This solution was frozen in liquid nitrogen and the headspace was evacuated under vacuum. The flask was then backfilled with 1 atm of H_2, which is equivalent to ~3.8 atm of H_2 at room temperature. The flask was then relocated into a freezer at −25 °C where it was allowed to react for 30-36 h without stirring. Upon completion of the reaction with H_2, the reddish-purple color of **2** converts to a pinker purple indicating the formation of **3**. Complex **3** is stable to vacuum and is relatively stable as a solid to air, but decomposes rapidly if exposed to air in the solution state. This complex is relatively stable below 0 °C, but slow decomposition occurs at this temperature and above. To characterize this product, the reaction vessel was pumped back into the nitrogen-filled glovebox and placed into a −35 °C freezer. The cold solution was then dried rapidly under vacuum and then analyzed by various techniques as described below.

Preparation of IR samples of 3

Nujol Mull

Complex **3** (8 mg), prepared in the method described above, was mixed in a cold mortar and pestle with minimal nujol in order to form a mustard-like suspension. This mixture was spread on a cooled KBr plate and with a second plate placed on top. The sample was then transferred in an air-free temporary container to the spectrometer, and a spectrum was collected.

Thin film on KBr plate

Complex **3** (8 mg), prepared in the method described above, was dissolved in cold, dry diethyl ether to form a concentrated solution. This was dropped on a cooled KBr plate and a second plate placed on top. The sample was then transferred in an air-free temporary container to the spectrometer, and a spectrum collected.

NMR spectroscopy

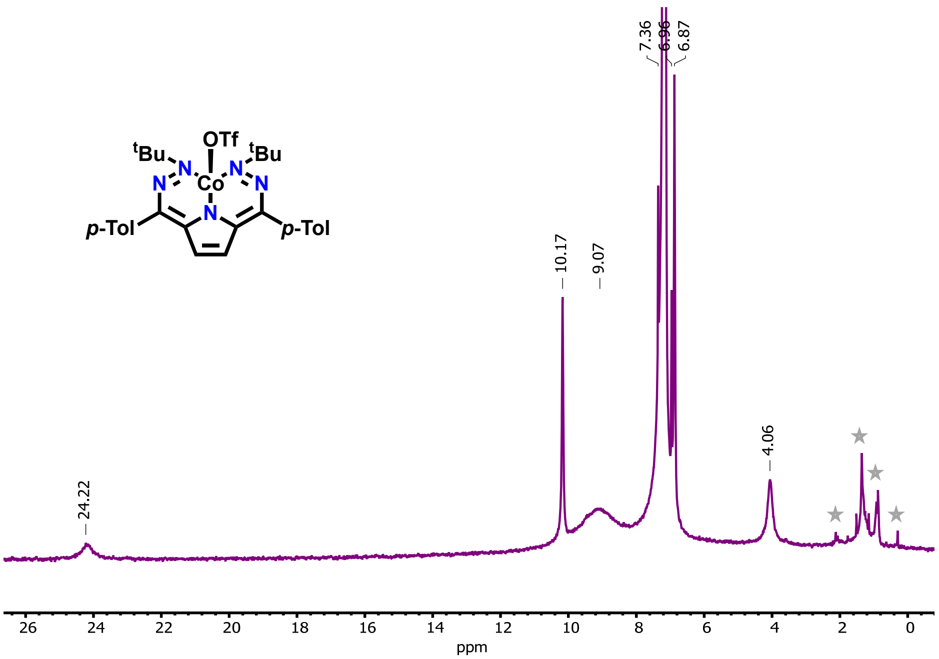

Figure S1. ^{1}H NMR of **2** in C_6D_6. Residual solvent or grease marked with stars.

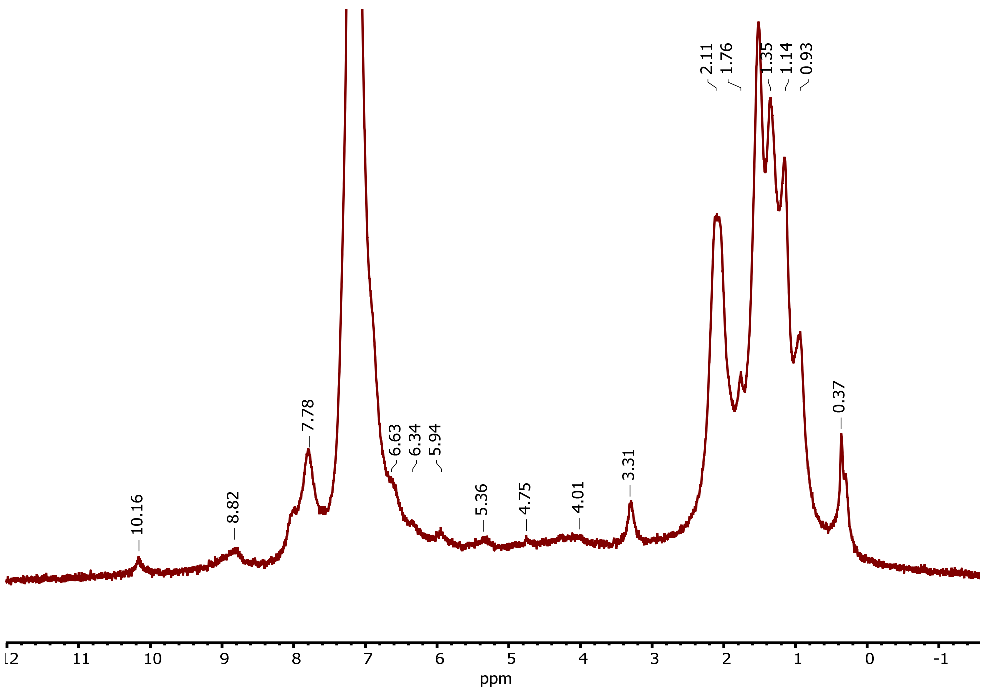

Figure S2. ^{1}H NMR of **3** in C_6D_6

UV-vis spectroscopy

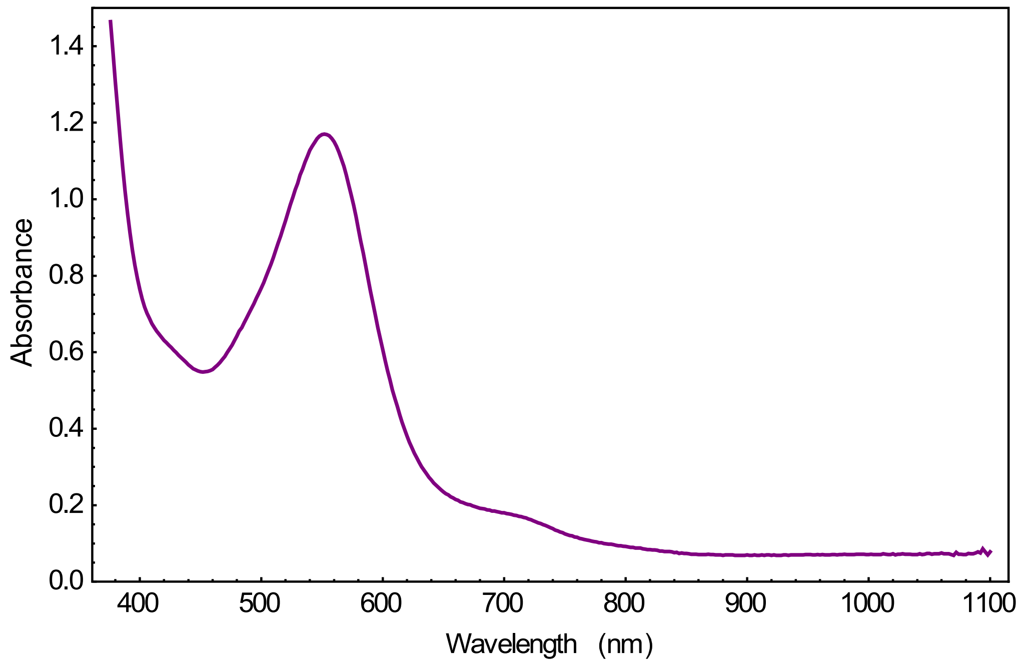

Figure S3. UV-vis of **1** from a 0.31 mM solution of **1** in toluene.

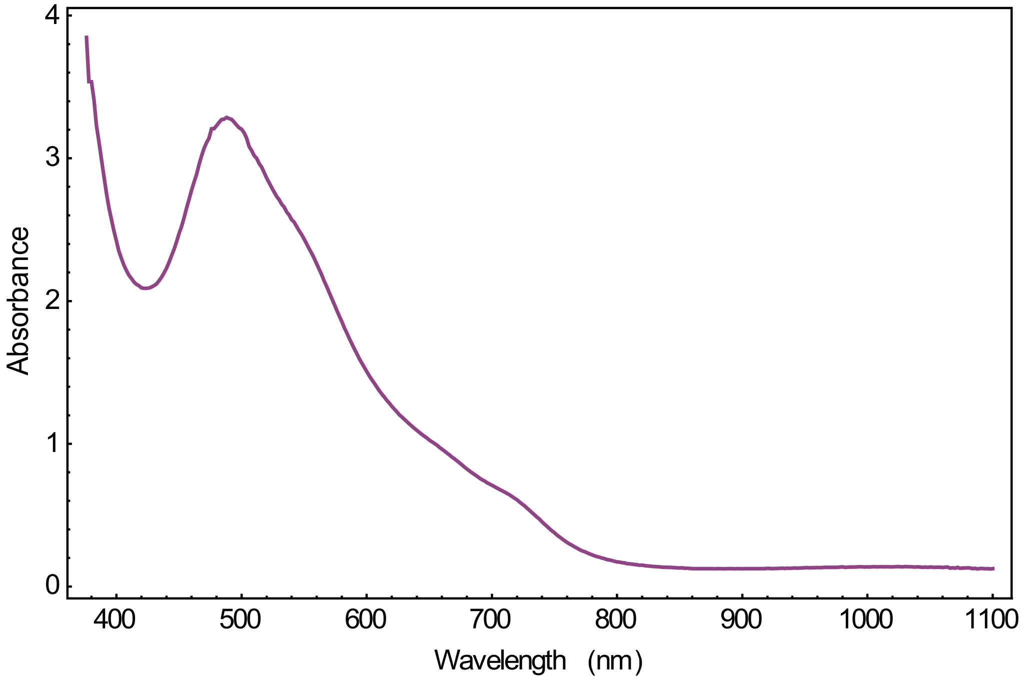

Figure S4. UV-vis of **2** from a 0.87 mM solution of **2** in benzene.

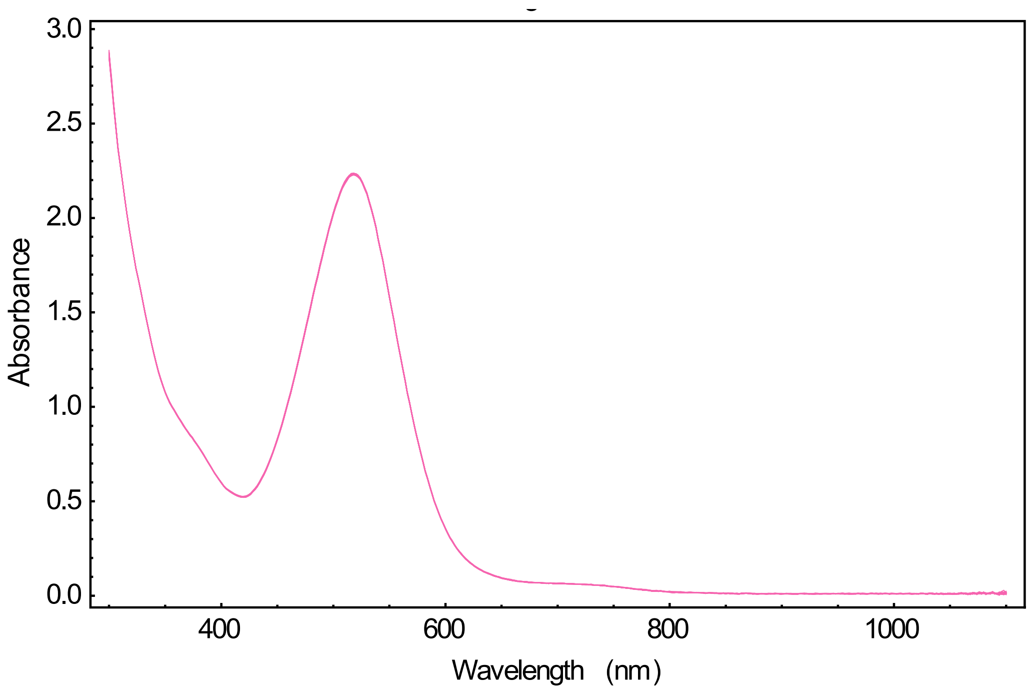

Figure S5. UV-vis of **3** from a solution in toluene at -35ºC.

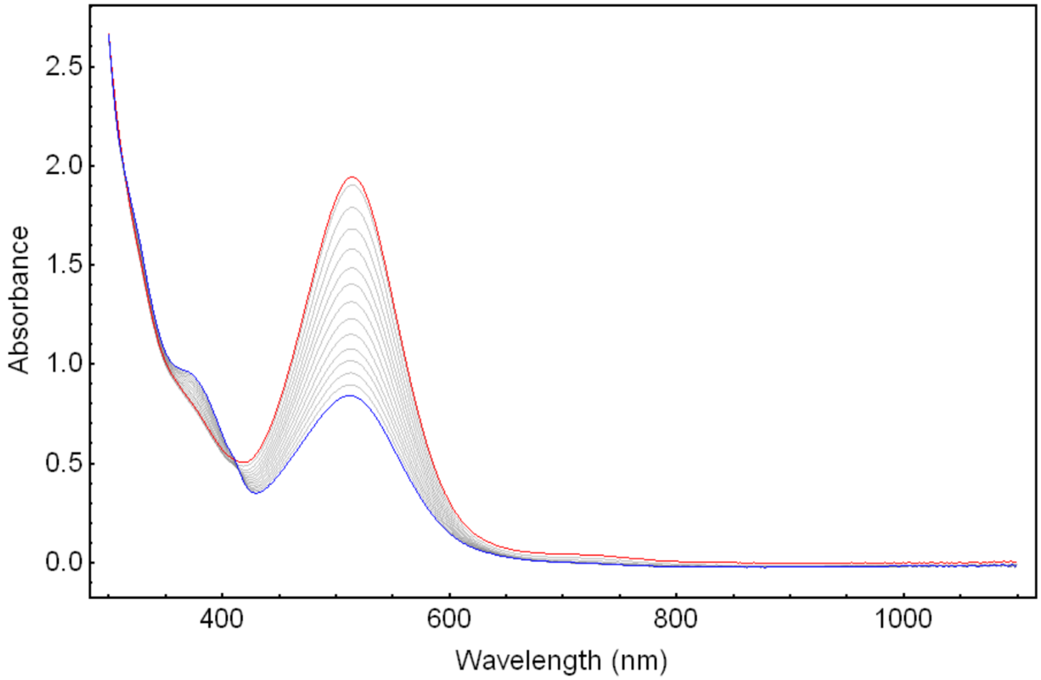

Figure S6. UV-vis of **3** in toluene at 0ºC over first 4.5 hours scans every 4.5 minutes

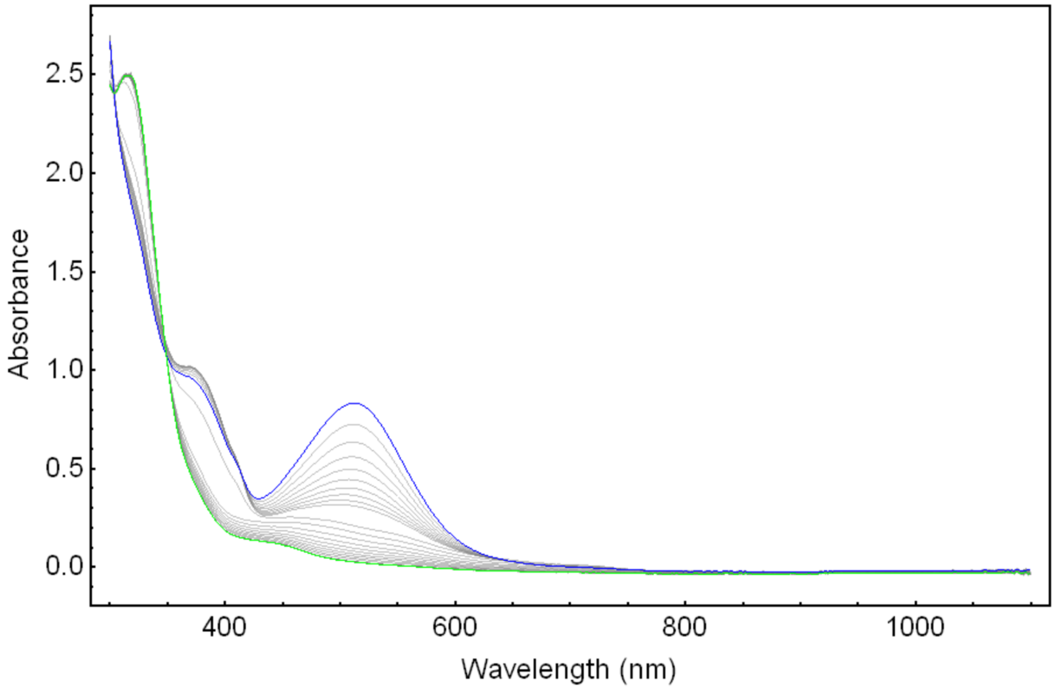

Figure S7. UV-vis of **3** in toluene at 0 ºC for second 10.5 hours (starting at 4.5 hours), scans every 31.5 minutes

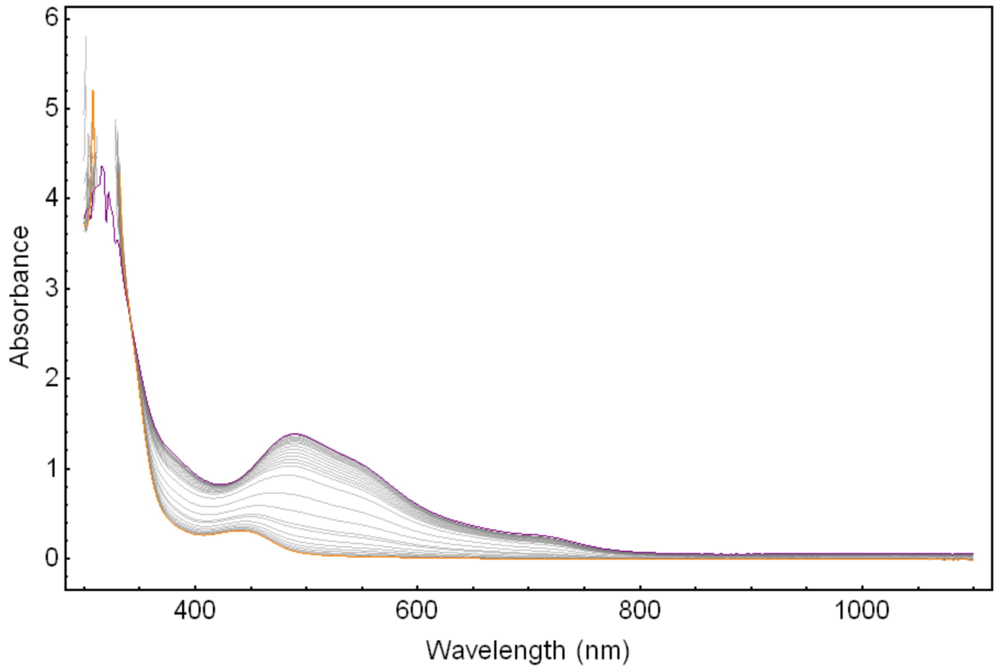

Figure S8. UV-vis of **2** + H_2 from a 0.24 mM solution of **2** in toluene (RT, scans every 13 minutes, 21 hours).

Vibrational Spectroscopy

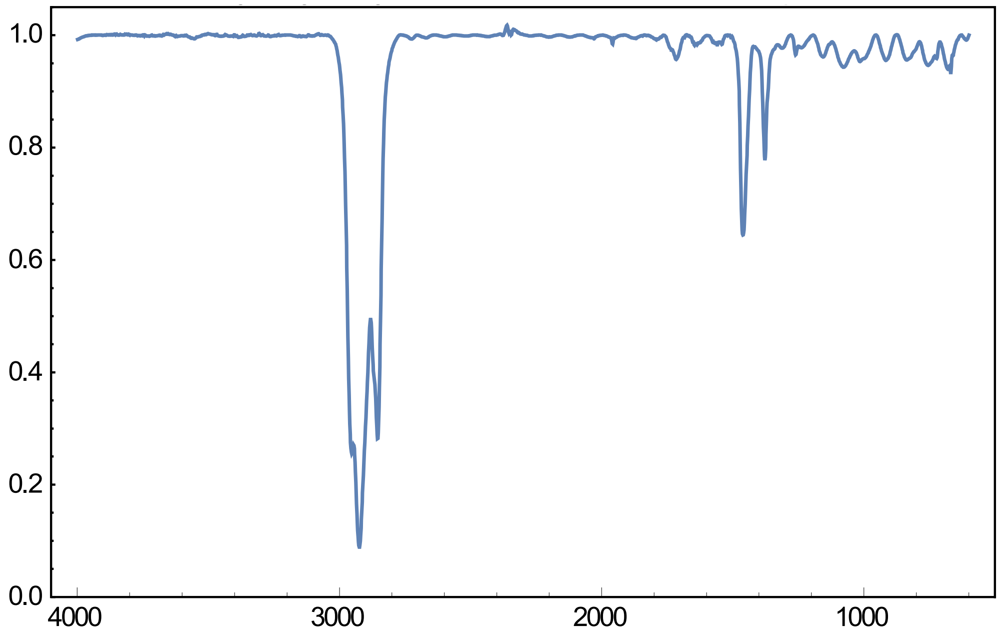

Figure S9. IR of nujol.

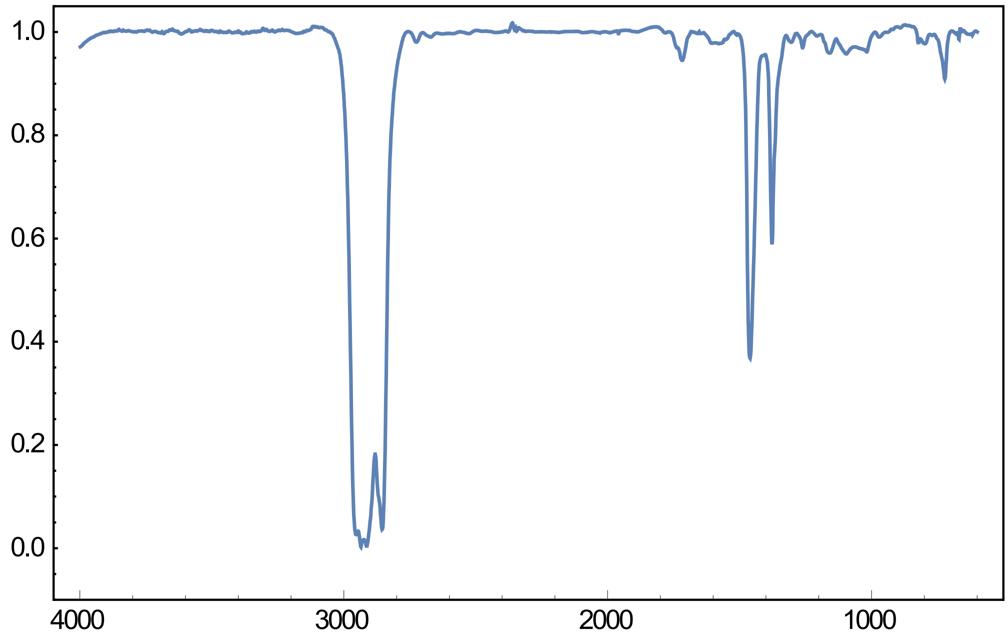

Figure S10. IR of **1** in nujol.

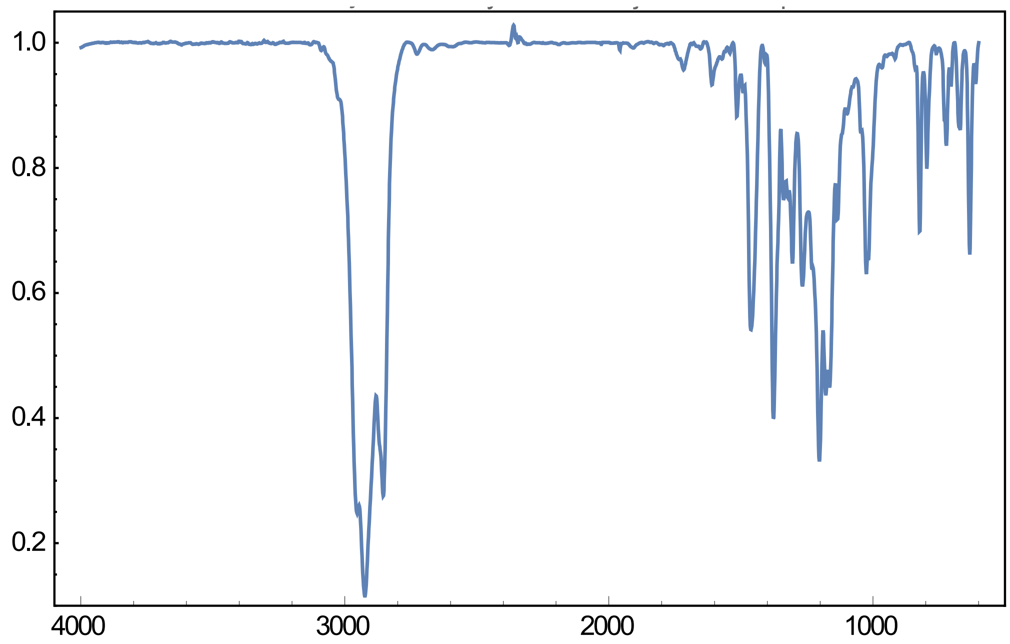

Figure S11. IR of **2** in nujol.

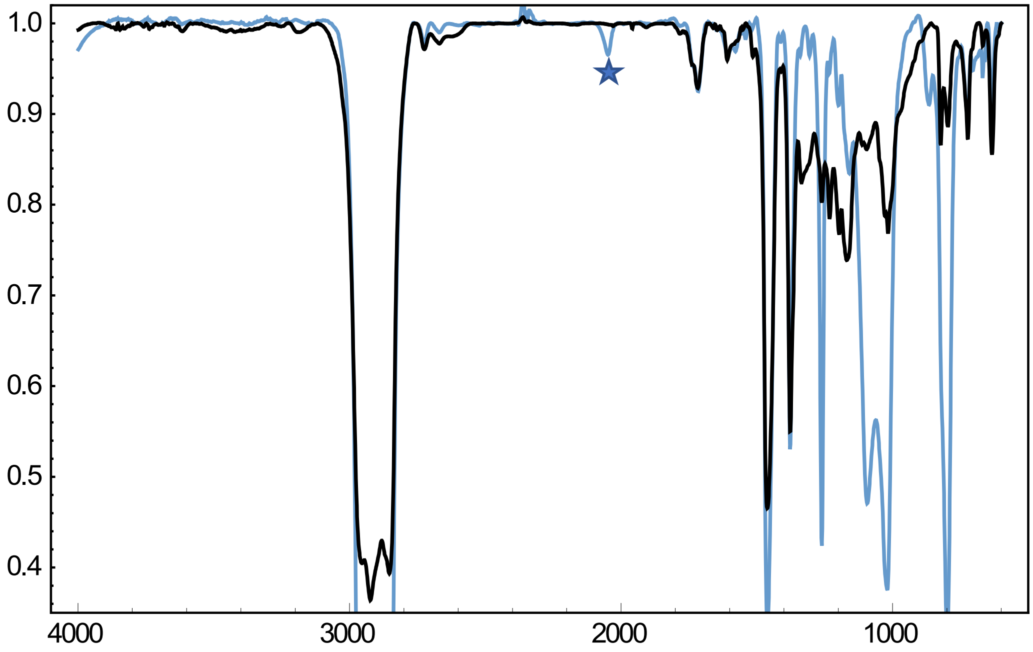

Figure S12. IR of **3** (black) and **3-D₂** (blue) in nujol. Star indicates the N–D stretch.

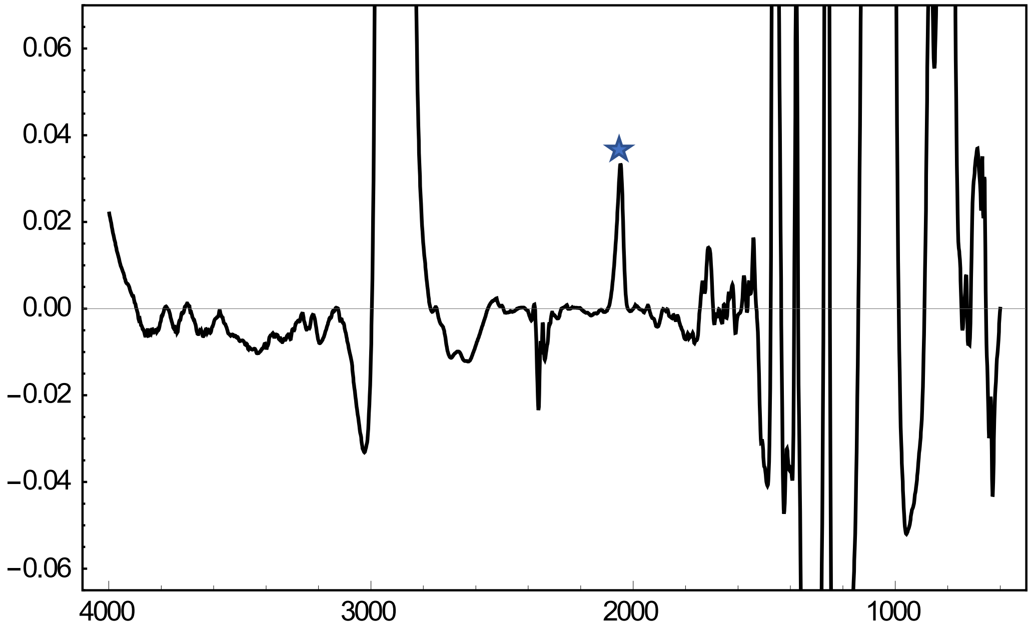

Figure S13. IR Difference Spectrum of **3** (black) and **3-D₂** (blue) in nujol. Star indicates the N–D stretch.

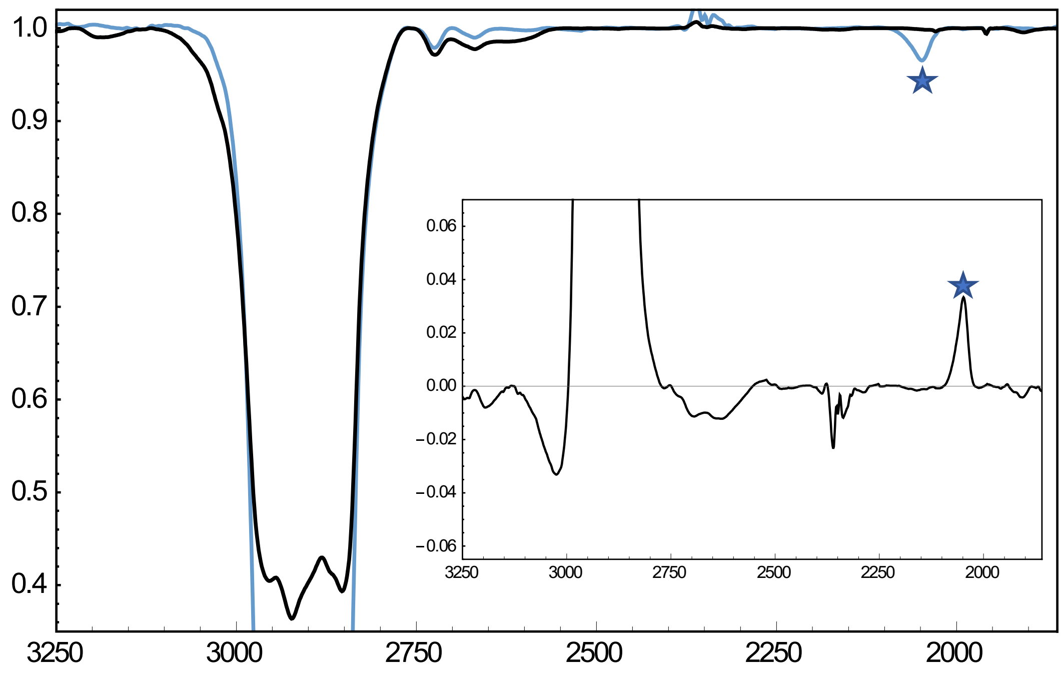

Figure S14. Zoomed IR with Difference Spectrum inset of **3** (black) and **3-D₂** (blue) in nujol. Star indicates the N–D stretch.

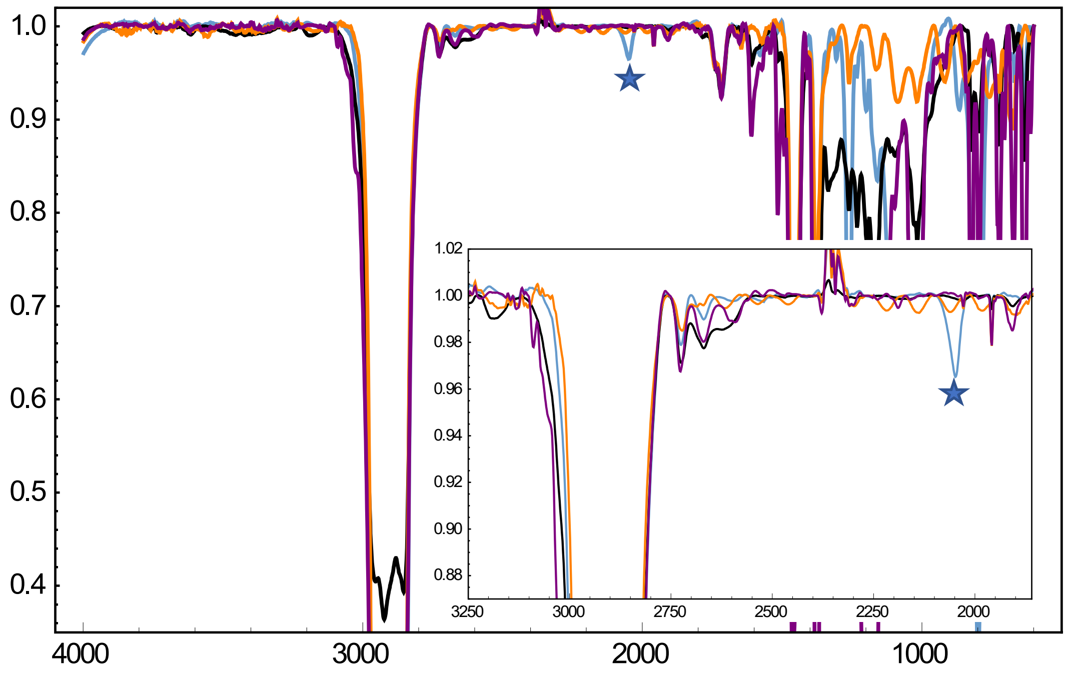

Figure S15. IR of nujol (orange) and **2** (purple), **3** (black) and **3-D₂** (blue) in nujol. Star indicates the N–D stretch.

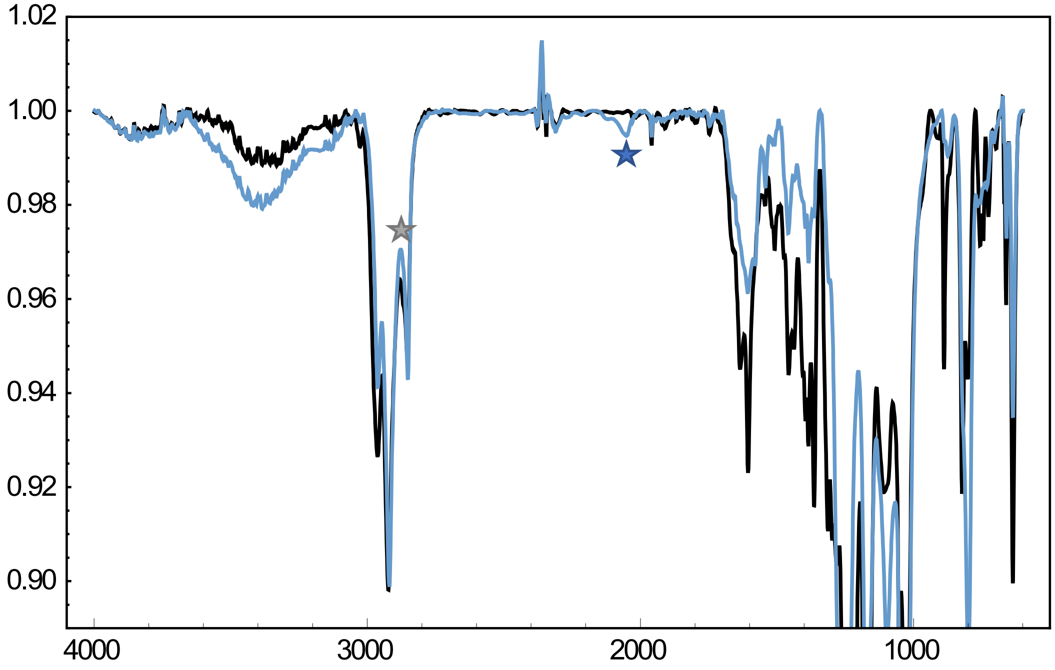

Figure S16. IR of **3** (black) and **3-D$_2$** (blue) as a thin film on KBr. Blue star indicates the N–D stretch. Gray star indicates the proposed position of the N–H stretch.

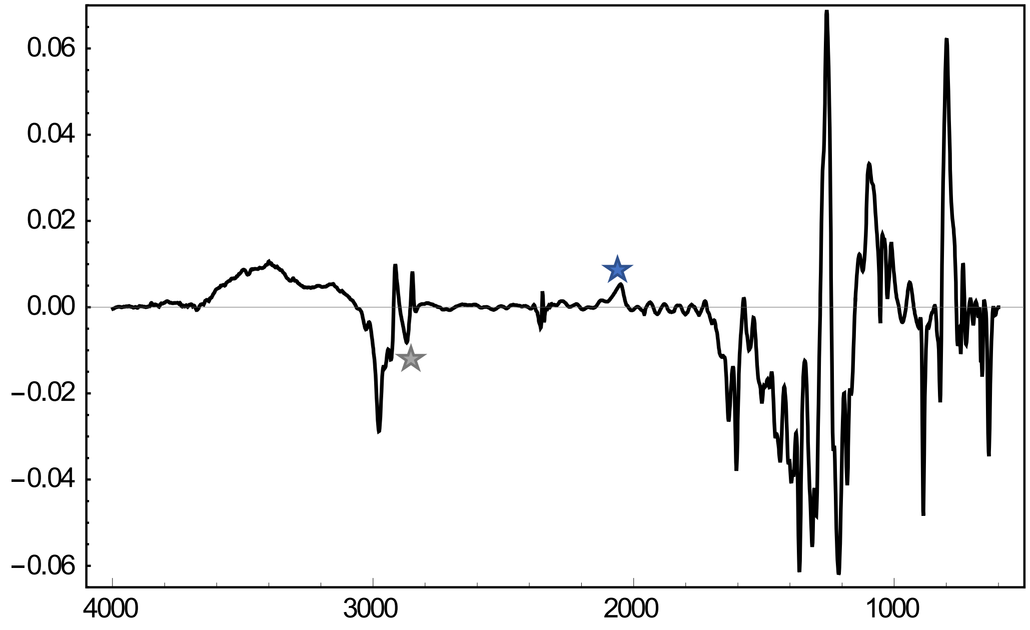

Figure S17. IR Difference Spectrum of **3** (black) and **3-D$_2$** (blue) as a thin film on KBr. Blue star indicates the N–D stretch. Gray star indicates the proposed position of the N–H stretch.

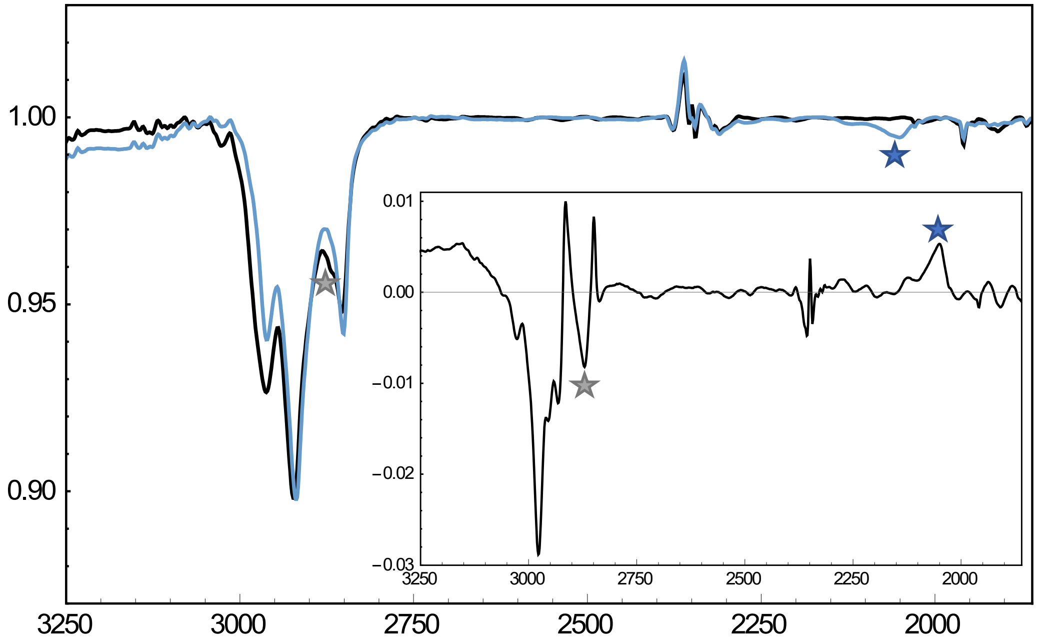

Figure S18. Zoomed IR with Difference Spectrum inset of **3** (black) and **3-D$_2$** (blue) as a thin film on KBr. Blue star indicates the N–D stretch. Gray star indicates the proposed position of the N–H stretch.

Cyclic Voltammetry

	Oxidative Wave 2	Oxidative Wave 2	Reductive Wave 1	Reductive Wave 2
$E_{1/2}$ (V)	0.30	-0.45	-0.99	-1.77

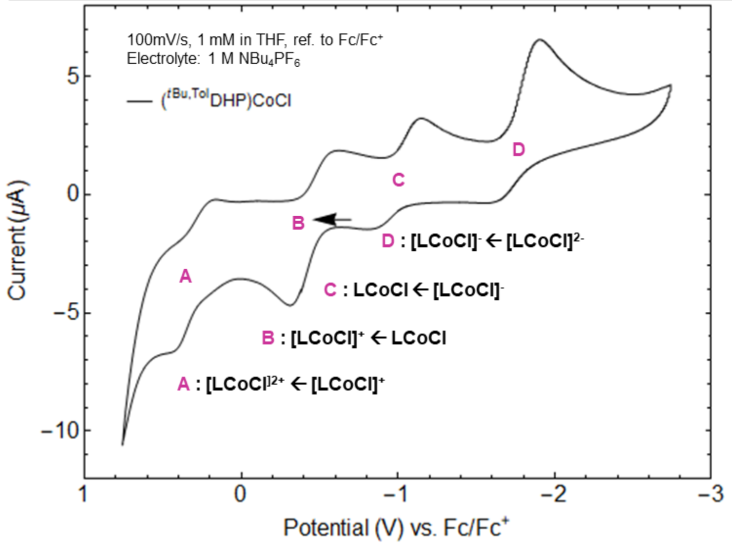

Figure S19. Cyclic Voltammogram of **1**.

	Oxidative Wave 1	Reductive Wave 0	Reductive Wave 1	Reductive Wave 2
$E_{1/2}$ (V)	0.443	-0.4065	-1.192	-2.105

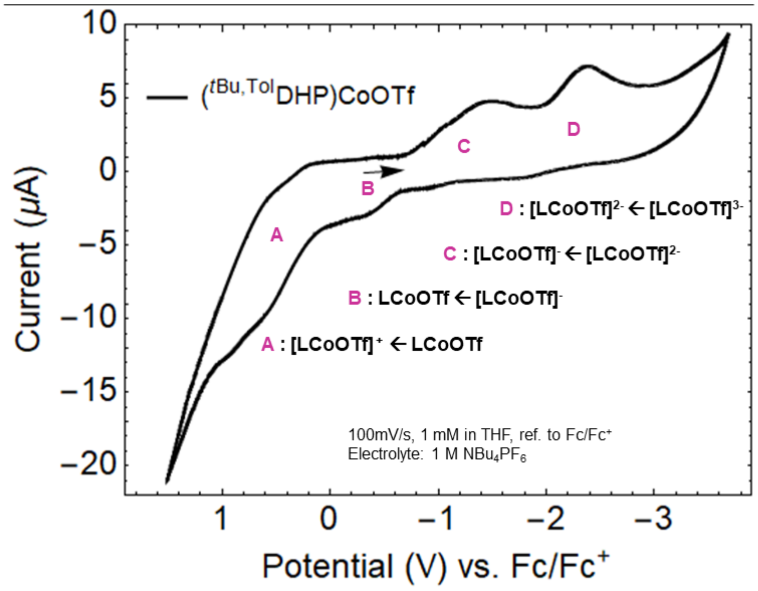

Figure S20. Cyclic Voltammogram of **2**.

EPR Spectroscopy

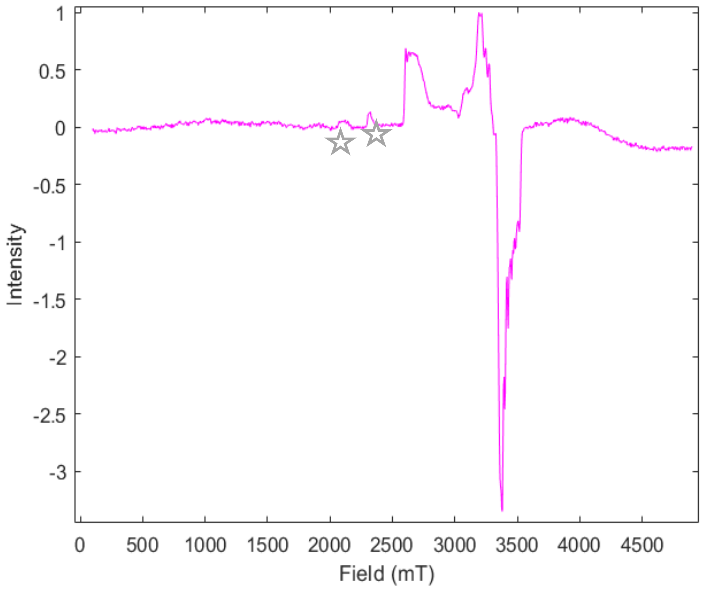

Figure S21. Full perpendicular-mode EPR spectrum of a 15 mM solution of **1** in toluene at 17 K. Some small unknown impurity peaks are marked. Conditions: MW frequency, 9.631 GHz; MW power, 2.0 mW.

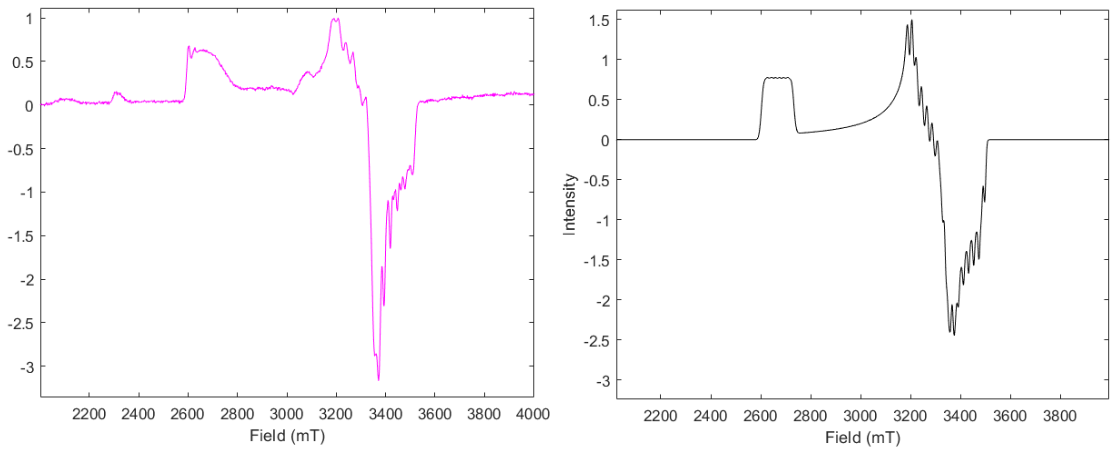

Figure S22. Small Window perpendicular-mode EPR spectrum (left) and simulated spectrum (right) of a 15 mM solution of **1** in toluene at 15 K. Conditions: MW frequency, 9.631 GHz; MW power, 2.0 mW.

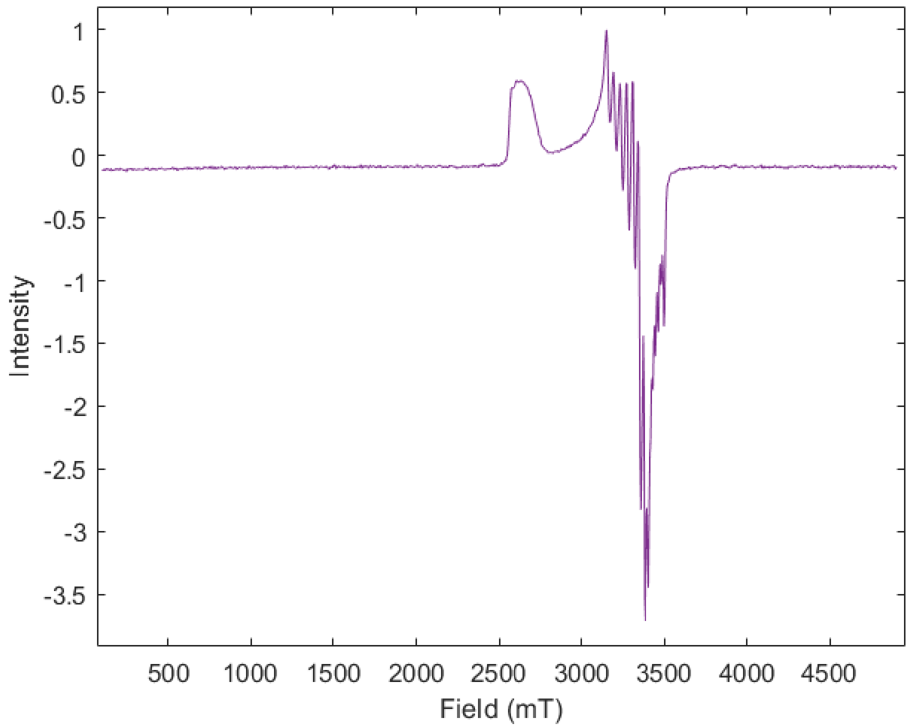

Figure S23. Full perpendicular-mode EPR spectrum of a 15 mM solution of **2** in toluene at 15 K. Conditions: MW frequency, 9.631 GHz; MW power, 2.0 mW.

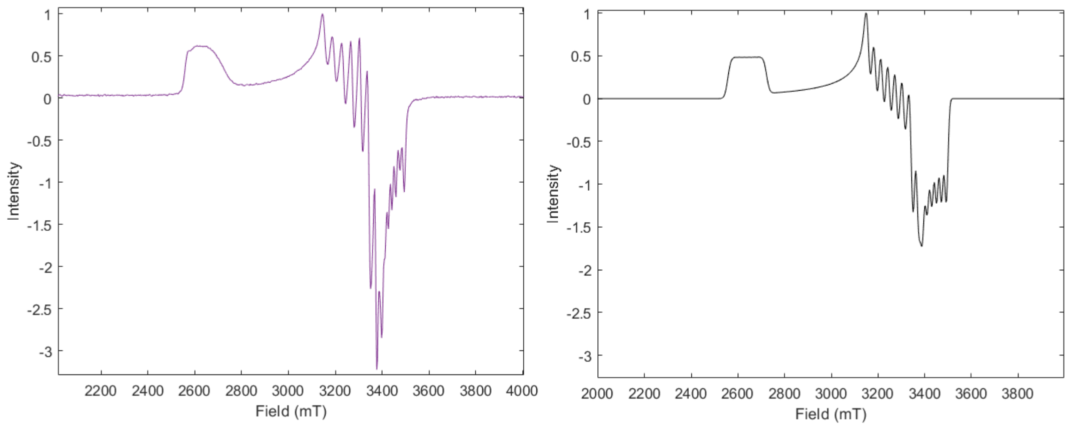

Figure S24. Small Window perpendicular-mode EPR spectrum (left) and simulated spectrum (right) of a 15 mM solution of **2** in toluene at 15 K. Conditions: MW frequency, 9.631 GHz; MW power, 2.0 mW.

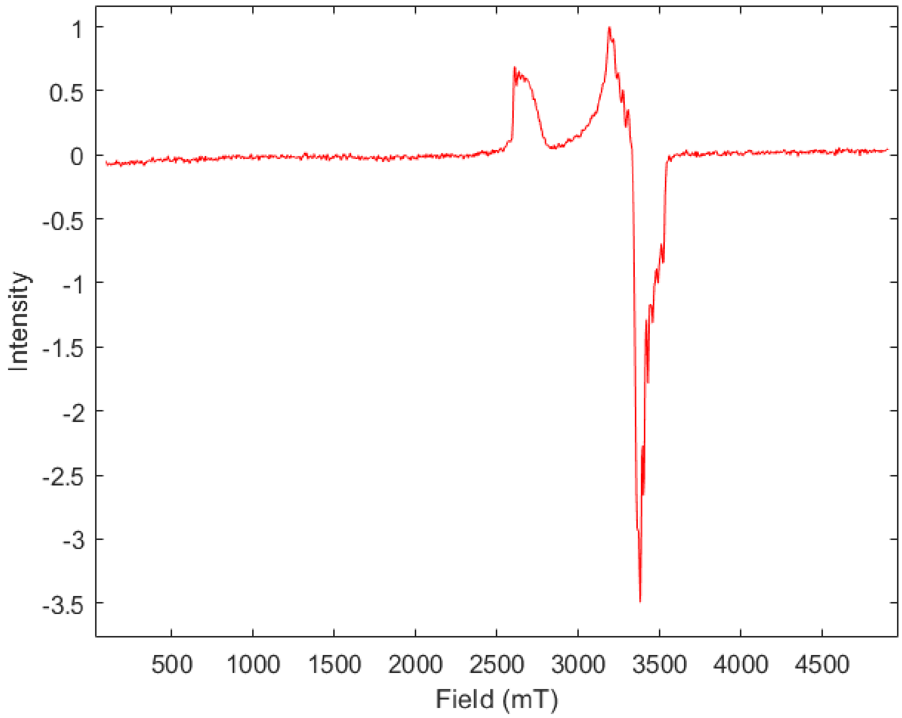

Figure S25. Full perpendicular-mode EPR spectrum of a 15 mM solution of **3** in toluene at 20 K. Conditions: MW frequency, 9.631 GHz; MW power, 2.0 mW.

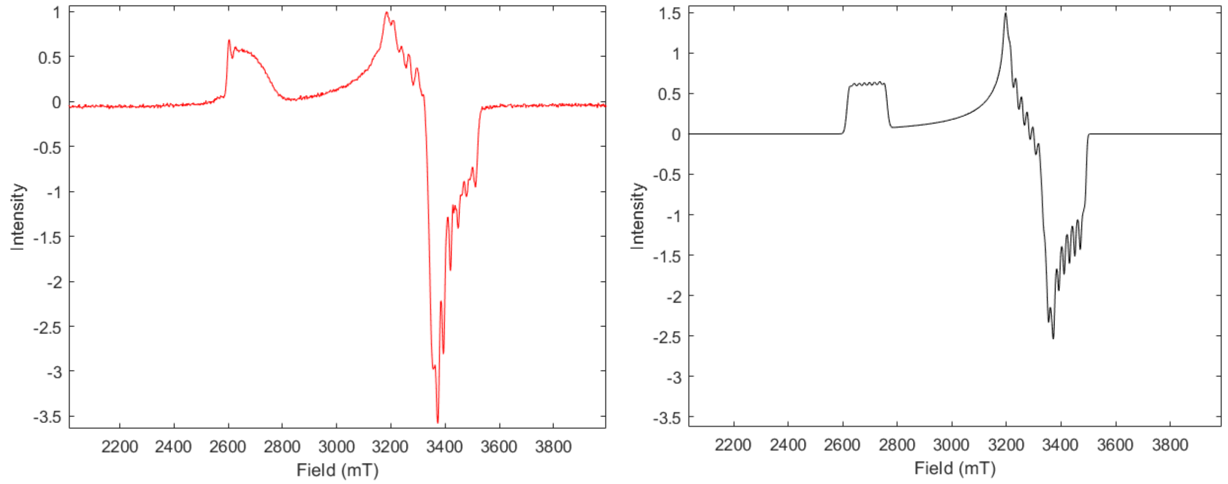

Figure S26. Small Window perpendicular-mode EPR spectrum (left) and simulated spectrum (right) of a 15 mM solution of **3** in toluene at 20 K. Conditions: MW frequency, 9.631 GHz; MW power, 2.0 mW.

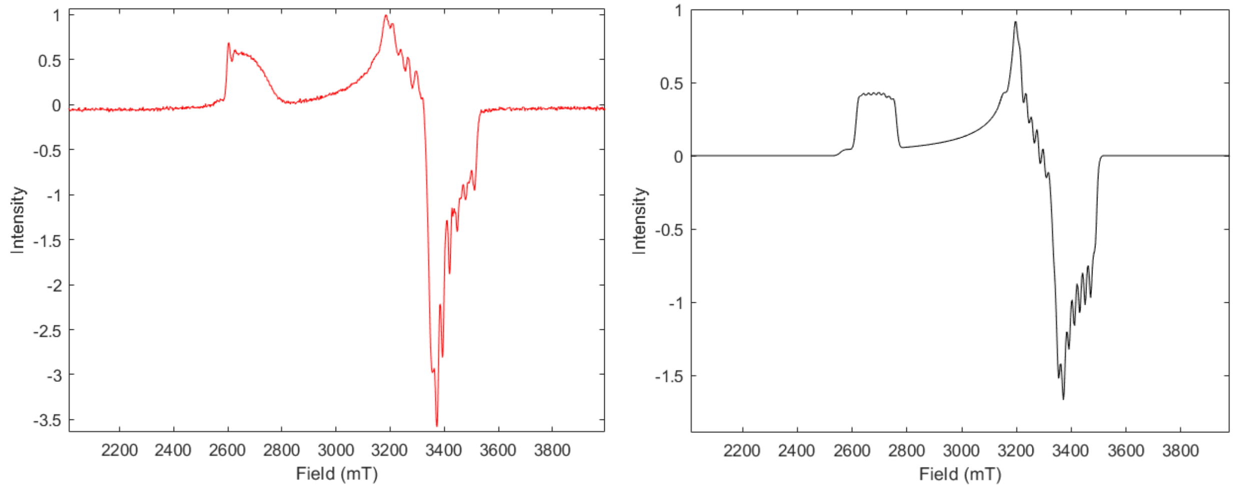

Figure S27. Small Window perpendicular-mode EPR spectrum and simulated spectrum overlay of a 15 mM solution of **3** in toluene at 20 K with 9.0% **2** impurity included. Conditions: MW frequency, 9.631 GHz; MW power, 2.0 mW.

Table S1. *g*-values for EPR

	g (x,y,z)	*Co-A*	*N-A*	*H-strain*
**1**	(2.0162, 2.0965, 2.5792)	(57.6, 62.4, 58.8)	(35.4, 44.7, 10.7)	(29, 35, 71)
**2**	(2.0102, 2.1071, 2.6066)	(56.5, 89.0, 75.6)	(12.1, 5.9, 11.2)	(40, 60, 102)
**3**	(2.0162, 2.0965, 2.5592)	(54.8, 61.0, 66.8)	(23.1, 24.5, -14.1)	(28, 37, 66)

Code to generate simulations of EPR Spectra.

1:

```
Exp2.mwFreq = 9.63;
Exp2.nPoints = 2048;
Exp2.Range = [150 450];
Sys2.g = [2.0162 2.0965 2.5792];
Sys2.Nucs = 'Co, N';
Sys2.A = [57.6 62.4 58.8; 35.4 44.7 10.7];
Sys2.HStrain = [29 35 71];
Vary2.g = [0.01 0.01 0.01];
Vary2.HStrain = [2 2 5];
Vary2.A = [2 2 5; 2 2 5];
plot(BCl,((ICl)/max(ICl)),BCl,1.5*pepper(Sys2,Exp2)/max(pepper(Sys2,Exp2)))
```

2:

```
Exp1.mwFreq=9.63;
Exp1.Range = [150 450]
Sys1.g = [2.0102 2.1070 2.6066]
Exp1.nPoints = 1024
Sys1.Nucs = 'Co, N';
Sys1.A = [56.5 89.0 75.6; 12.1 5.9 11.2];
Sys1.HStrain = [40 60 102];
Vary1.g = [0.04 0.02 0.02];
Vary1.HStrain = [10 10 10];
Vary1.A = [20 20 20; 10 10 10];
plot(Bo,(Io-Iblank)/max(Io-Iblank),Bo,pepper(Sys1,Exp1)/max(pepper(Sys1,Exp1)))
```

3:

```
Exp3.mwFreq=9.63;
Exp3.nPoints = 2048;
Exp3.Range = [150 450];
Sys3.g = [2.0162 2.0965 2.5592];
Sys3.Nucs = 'Co,N';
Sys3.A = [54.8 61.0 66.8; 23.1 24.5 -14.1];
Sys3.HStrain = [28 37 66];
Vary3.g = [0.02 0.02 0.02];
Vary3.HStrain = [5 5 10];
Vary3.A = [8 8 15; 8 8 15];
plot(BH2,(IH2)/max(IH2),BH2,pepper(Sys3,Exp3)/max(pepper(Sys3,Exp3)))
```

or

```
plot(BH2,((IH2)/max(IH2)),Bo,0.91*(pepper(Sys3,Exp3)/max(pepper(Sys3,Exp3)))+0.09*(pepper(Sys1,
Exp1)/max(pepper(Sys1,Exp1))))
```

X-ray Absorption Spectroscopy

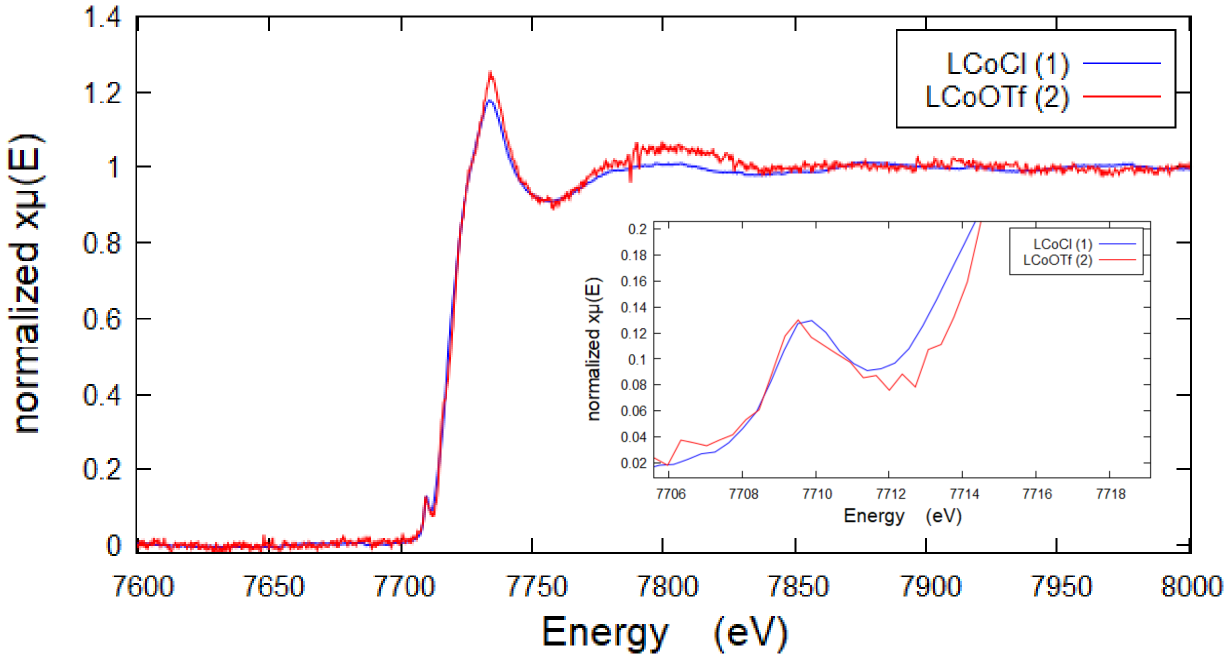

Figure S28. X-ray absorption spectra of **1** (red) and **2** (blue) with pre-edge features at 7709.7 and 7709.3 eV. These were all collected as frozen solutions at 183 K, with 1 and 2 in toluene. Inset: Pre-edge features.

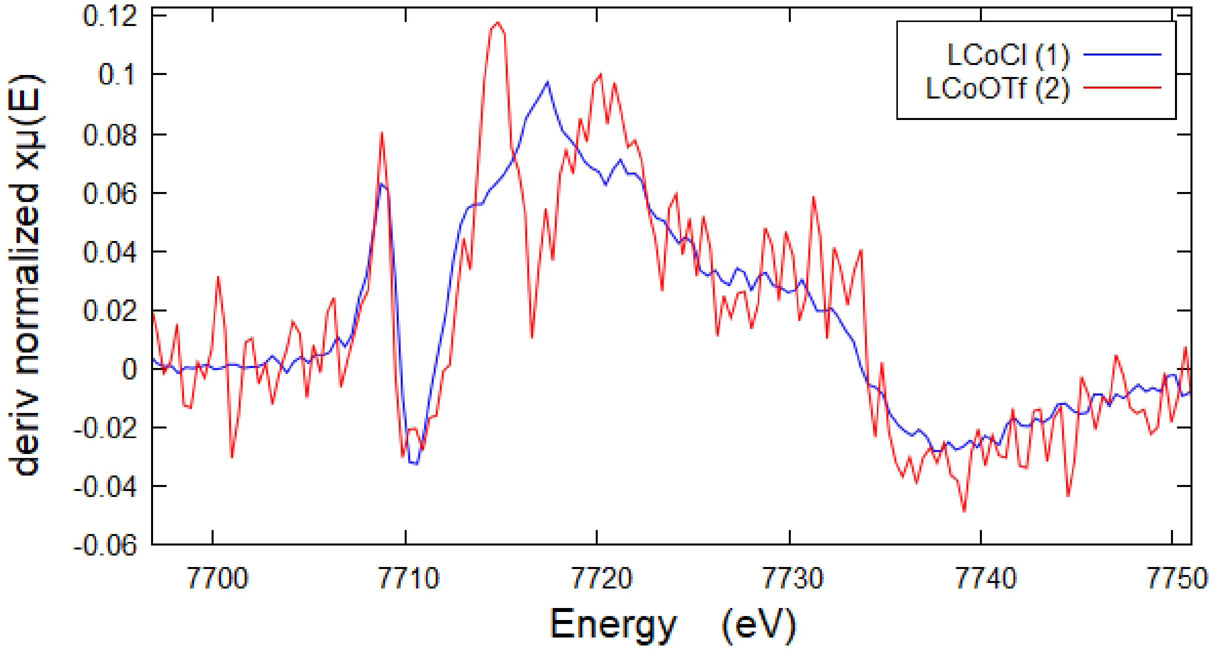

Figure S29. Derivative of X-ray absorption spectra of **1** (red) and **2** (blue).

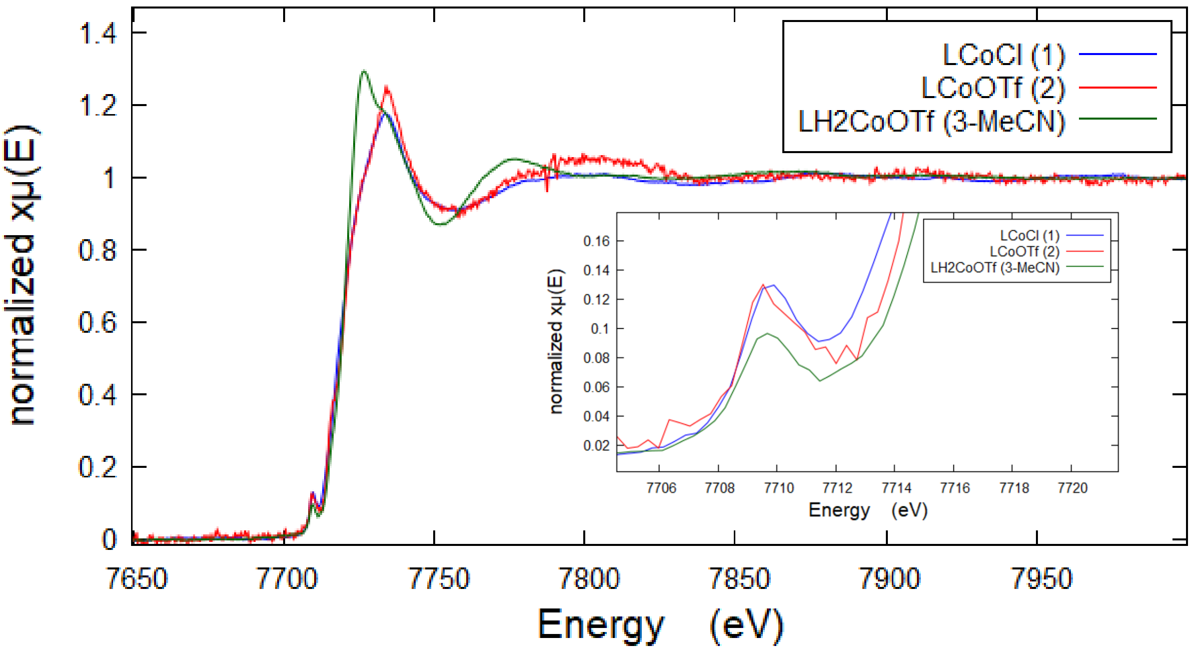

Figure S30. X-ray absorption spectra of **1** (blue), **2** (red) and **3-MeCN** (green) with pre-edge features at 7709.7, 7709.3, and 7709.3 eV respectively. These were all collected as frozen solutions at 183 K, with **1** and **2** in toluene and **3-MeCN** in a 1:4 MeCN:toluene mixture.

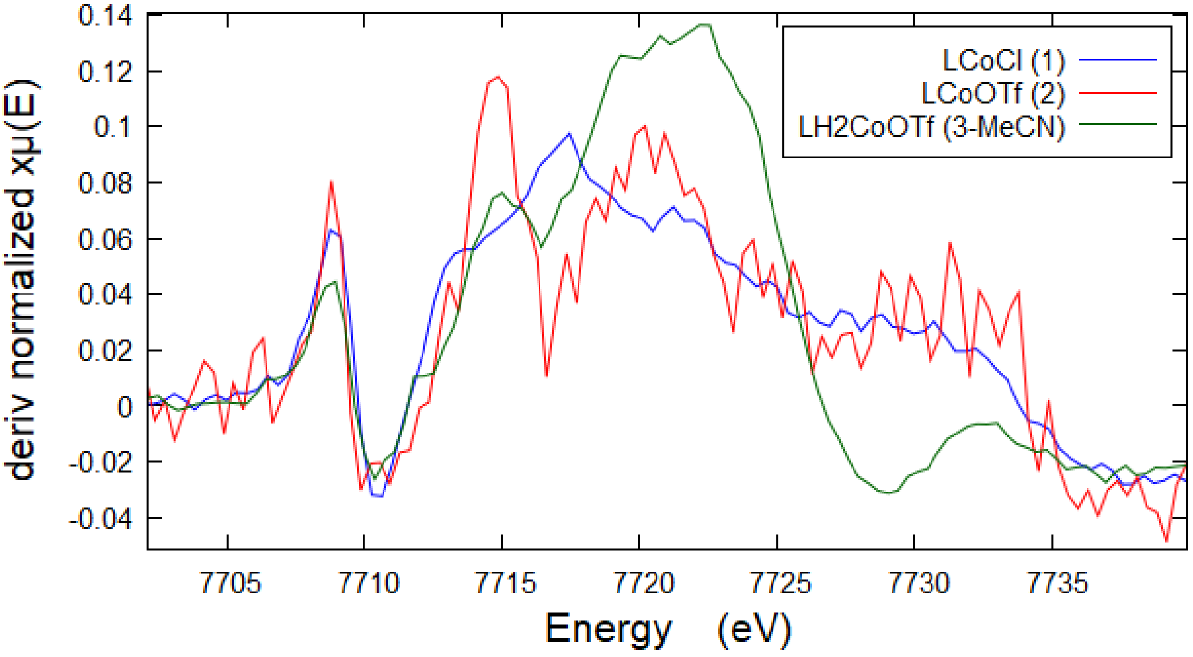

Figure S31. Derivative of X-ray absorption spectra of **1** (blue), **2** (red) and **3-MeCN** (green).

Single Crystal X-ray Diffraction

X-Ray Structure Determination.

The diffraction data were measured at 100 K on a Bruker D8 VENTURE with PHOTON 100 CMOS detector system equipped with a Mo-target micro-focus X-ray tube (λ = 0.71073 Å. Data reduction and integration were performed with the APEX3 software package (Bruker AXS, version 2015.5-2, 2015). Data were scaled and corrected for absorption effects using the multi-scan procedure in SADABS (Bruker AXS, version 2014/5, 2015, part of Bruker APEX3 software package). The structure was solved by the dual method implemented in SHELXT[4] and refined by a full-matrix least-squares procedure using OLEX23[5] software package (XL refinement program version 2014/7[6]). Suitable crystals were mounted on a cryo-loop and transferred into the cold nitrogen stream of the Bruker D8 Venture diffractometer. C–H hydrogen atoms were constrained to idealized geometries and allowed to ride on their carrier atoms with an isotropic displacement parameter related to the equivalent displacement parameter of the carrier atoms. Finally, we note some B-level alerts for **2** related to the quality of the diffraction which was weak at higher angles. Despite this weak diffraction, the connectivity, assignment, and bond lengths for **2** are still suitable for discussion.

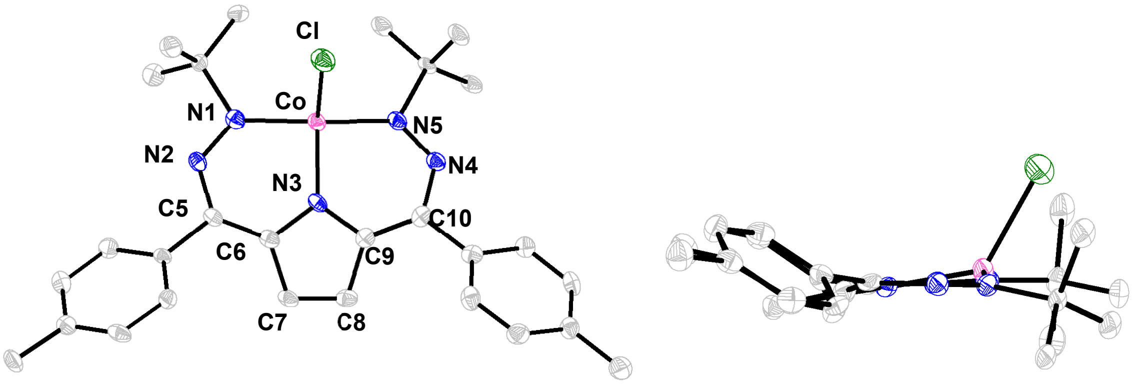

Figure S32. SXRD of **1**. Co (pink), N (blue), C (gray), Cl (green), H-atoms omitted.

Selected bond lengths (Å): Co-N1/N5: 1.883(4), 1.891(5); Co-N3: 1.861(4); N1-N2/N4-N5: 1.303(6), 1.288(6); Co-Cl: 2.198(2); N2-C5/N4-C10: 1.343(7), 1.338(7); C5-C6/C9-C10: 1.388(8), 1.383(8); C6-C7/C8-C9: 1.434(8), 1.433(8); C7-C8: 1.351(8). Selected bond angles (°): N1-M-N5: 164.7(2); N3-M-Cl: 129.1(2).

Table S2. SXRD of **1**.

Empirical formula	$C_{28}H_{34}ClCoN_5$
Formula weight	534.98
Temperature/K	100(2)
Crystal system	monoclinic
Space group	$P2_1/n$

a/Å	7.0908(10)
b/Å	15.967(2)
c/Å	23.272(3)
α/°	90
β/°	93.817(4)
γ/°	90
Volume/Å^3	2629.0(7)
Z	4
ρ_{calc}g/cm^3	1.352
μ/mm^{-1}	0.780
F(000)	1124.0
Crystal size/mm^3	0.15 × 0.04 × 0.025
Radiation	MoKα (λ = 0.71073)
2Θ range for data collection/°	4.338 to 50.804
Index ranges	-8 ≤ h ≤ 8, -19 ≤ k ≤ 19, -28 ≤ l ≤ 28
Reflections collected	34125
Independent reflections	4839 [R_{int} = 0.1513, R_{sigma} = 0.1075]
Data/restraints/parameters	4839/0/324
Goodness-of-fit on F^2	1.141
Final R indexes [I>=2σ (I)]	R_1 = 0.0852, wR_2 = 0.1510
Final R indexes [all data]	R_1 = 0.1433, wR_2 = 0.1714
Largest diff. peak/hole / e Å^{-3}	0.83/-0.63

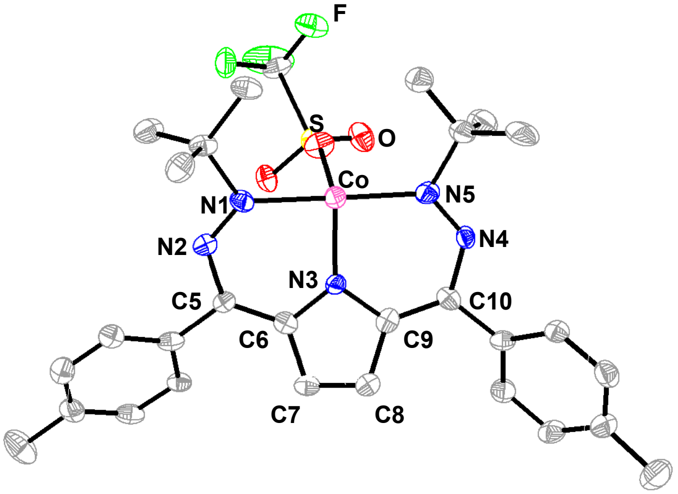

Figure S33. SXRD of **2** (polymeric structure bridged by triflates). Co (pink), N (blue), C (gray), F (lime green), O (red), S (yellow), H-atoms omitted.

Selected bond lengths (Å): Co-N1/N5: 1.994(8), 2.000(8); Co-N3: 1.916(8); N1-N2/N4-N5: 1.274(10), 1.251(10); Co-O/Co-O: 2.168(7), 2.126(7); N2-C5/N4-C10: 1.371(11), 1.385(11); C5-

C6/C9-C10: 1.383(13); C6-C7/C8-C9: 1.431(13), 1.452(13); C7-C8: 1.310(13). Selected bond angles (°): N1-M-N5: 178.1(3); N3-M-O: 113.2(3), 105.1(3).

Table S3. SXRD of **2**.

Empirical formula	$C_{29}H_{34}CoF_3N_5O_3S$
Formula weight	648.60
Temperature/K	100.01
Crystal system	monoclinic
Space group	$P2_1/c$
a/Å	10.791(3)
b/Å	23.464(6)
c/Å	11.749(3)
α/°	90
β/°	97.322(8)
γ/°	90
Volume/Å^3	2950.5(13)
Z	4
ρ_{calc}g/cm^3	1.460
μ/mm^{-1}	0.711
F(000)	1348.0
Crystal size/mm^3	0.37 × 0.135 × 0.073
Radiation	MoKα (λ = 0.71073)
2Θ range for data collection/°	4.926 to 51.438
Index ranges	$-12 \leq h \leq 13$, $-28 \leq k \leq 28$, $-14 \leq l \leq 14$
Reflections collected	33370
Independent reflections	5544 [R_{int} = 0.2243, R_{sigma} = 0.1243]
Data/restraints/parameters	5544/102/417
Goodness-of-fit on F^2	1.058
Final R indexes [I>=2σ (I)]	R_1 = 0.1276, wR_2 = 0.2773
Final R indexes [all data]	R_1 = 0.1696, wR_2 = 0.2994
Largest diff. peak/hole / e Å^{-3}	1.21/-0.76

Density Functional Theory (DFT)

Geometry Optimizations

Geometry optimization calculations were performed with ORCA[7] software suite using density functional theory (DFT). Geometries were fully optimized starting from coordinates generated from finalized cifs of the compound crystal structures. The BP86 functional was used for geometry optimizations, spin density plot calculations, and frequency calculations on **1**, **2**, and **3** in ORCA 4. In ORCA 5, O3LYP was used to calculate transition state structures for the reactivity of **2** with H_2, transition state structures for the reaction of **3** with 1-hexene, and the various isomers of 2-methyl-pent-1,3-ene and alpha-cyclopropyl styrene and their products. PBE0 in ORCA 4 was used for TDDFT calculations.

All these calculations were done with a basis set of def2-SVP on H. For the BP86 and PBE0 calculations, def2-TZVPP was used on Co, N, S, O and F, and def2-TZVP on C atoms. The resulting structures were confirmed to be minima on the potential energy surface by frequency calculations using ORCA. For the O3LYP calculations with triflate bound, def2-TZVPP was used on Co, N, S, O, most carbons and F, and def2-TZVP(-f) used on C atoms not part of the conjugated ligand. For O3LYP calculations without triflate bound, def2-TZVPP was used for Co, with def2-TZVP for all other N, C, O, S and F atoms.

Notation: LS: low spin, HS: high spin,

2-H$_2$: [($^{tBu, Tol}$DHP)Co-H$_2$]OTf, **2-H$_2$**$^+$: [($^{tBu, Tol}$DHP)Co-H$_2$]$^+$,

2H-H: [($^{tBu, Tol}$DHP-H)Co-H]OTf, **2H-H**$^+$: [($^{tBu, Tol}$DHP-H)Co-H]$^+$,

3: [($^{tBu, Tol}$DHP-H$_2$)Co]OTf, **3**$^+$: [($^{tBu, Tol}$DHP-H$_2$)Co]$^+$,

4: [($^{tBu, Tol}$DHP-H)Co]OTf, **4**$^+$: [($^{tBu, Tol}$DHP-H)Co]$^+$,

3-hexene: [($^{tBu, Tol}$DHP-H$_2$)Co(1-hexene)]OTf, **3-hexene**$^+$: [($^{tBu, Tol}$DHP-H$_2$)Co(1-hexene)]$^+$,

4-hexyl-β: [($^{tBu, Tol}$DHP-H)Co(n-hexyl)]OTf, **4-hexyl-β**$^+$: [($^{tBu, Tol}$DHP-H)Co(n-hexyl)]$^+$,

4-hexyl-α: [($^{tBu, Tol}$DHP-H)Co(2-hexyl)]OTf, **4-hexyl-α**$^+$: [($^{tBu, Tol}$DHP-H)Co(2-hexyl)]$^+$,

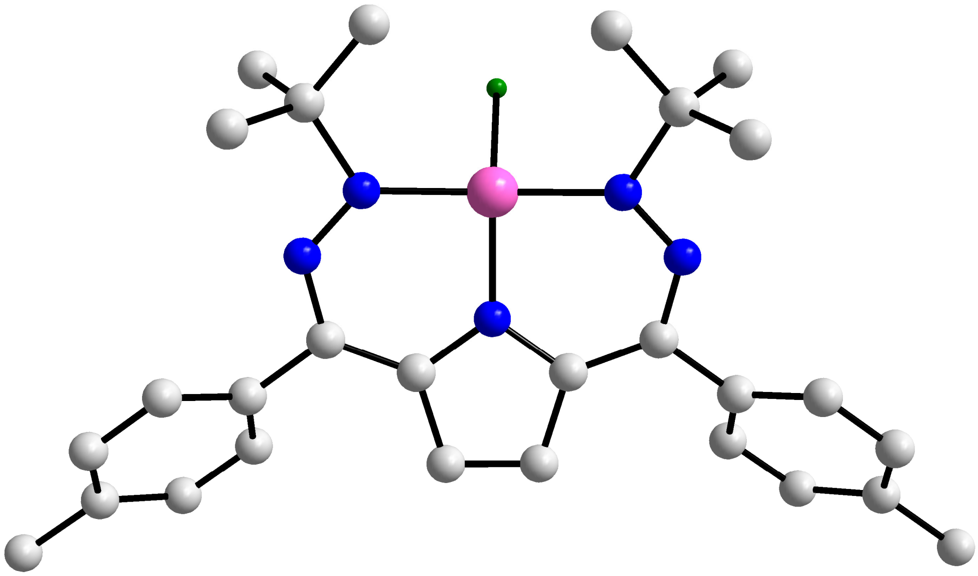

Figure 34. Calculated structure of **1**. All C–H hydrogen atoms have been removed for clarity.

Table S4. Calculated coordinates of **1**.

Co	2.271469	6.360438	5.604173
Cl	2.849481	4.259870	6.056730
N	3.954303	7.073722	3.371324

N	3.196142	7.932496	6.036680
N	1.160985	7.433294	8.044032
N	2.971470	6.382211	3.848598
N	1.093165	6.589993	7.064894
C	4.206092	8.510167	5.298473
C	5.851589	8.517849	3.387600
C	2.422697	5.500204	2.709333
C	-0.170635	5.727570	7.283726
C	3.046631	8.682382	7.182339
C	4.658716	9.701375	5.968372
H	5.400391	10.392738	5.579843
C	1.097523	4.860011	3.103397
H	1.221294	4.164793	3.940879
H	0.347356	5.620454	3.364693
H	0.711362	4.294513	2.242972
C	4.639354	8.001200	4.062883
C	2.091783	9.087119	9.481544
C	3.272638	9.372540	10.189843
H	4.232528	9.036835	9.793446
C	5.857224	8.759554	2.000759
H	4.945938	8.580154	1.428458
C	-0.522803	4.929245	6.034153
H	-0.701366	5.593197	5.176699
H	0.259303	4.206716	5.780136
H	-1.454014	4.375813	6.224624
C	3.940688	9.810045	7.128734
H	3.985771	10.607999	7.863954
C	8.184828	9.483488	2.078777

C	7.039590	8.748286	4.101820
H	7.078568	8.520942	5.168718
C	8.181946	9.223129	3.455637
H	9.096370	9.381733	4.033164
C	-1.356846	6.647361	7.619713
H	-1.192988	7.194572	8.554619
H	-1.534847	7.372082	6.810423
H	-2.260158	6.028463	7.726792
C	6.998947	9.237821	1.365154
H	6.970337	9.433522	0.289726
C	0.835170	10.162015	11.268055
H	-0.128352	10.475011	11.678480
C	9.413218	10.006957	1.380403
H	9.226491	10.998203	0.936905
H	10.257888	10.103210	2.076505
H	9.721359	9.339468	0.560139
C	2.012811	10.459154	11.972394
C	2.121270	8.362342	8.189083
C	0.869322	9.484493	10.051595
H	-0.057602	9.268707	9.518838
C	0.136823	4.778320	8.453172
H	1.000218	4.143020	8.211350
H	0.356040	5.354082	9.362673
H	-0.735803	4.134413	8.642596
C	3.229773	10.048003	11.408943
H	4.163506	10.248925	11.940730
C	3.473365	4.413568	2.431808
H	4.421665	4.871064	2.120021

H	3.645500	3.809987	3.334477
H	3.116272	3.753233	1.626161
C	1.968666	11.163550	13.303501
H	1.077240	11.801543	13.389825
H	1.932569	10.436196	14.131678
H	2.860065	11.789250	13.457292
C	2.191780	6.380467	1.469319
H	1.492813	7.199200	1.696544
H	3.129538	6.810372	1.100131
H	1.748872	5.764050	0.672990

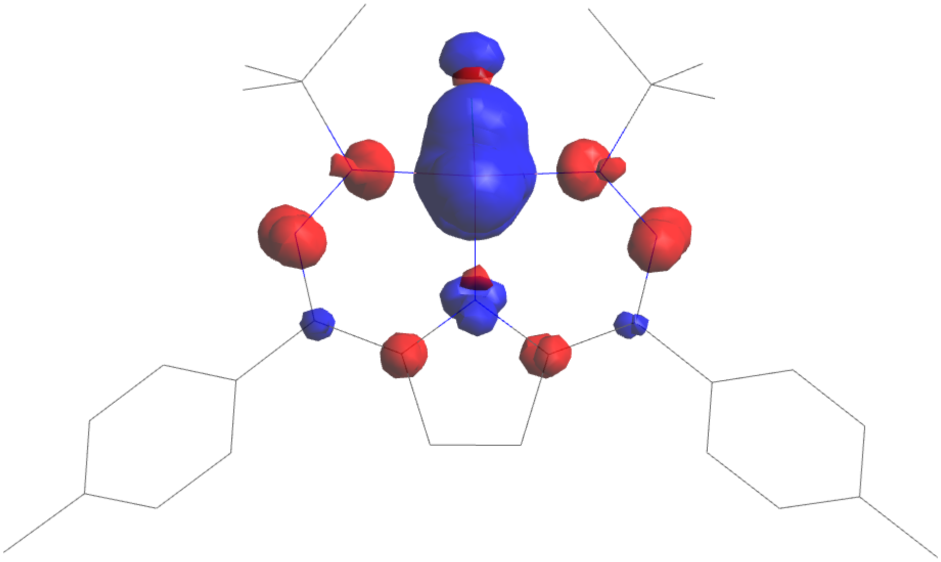

Figure S35. Spin density plot of **1** at an iso value of 0.003.

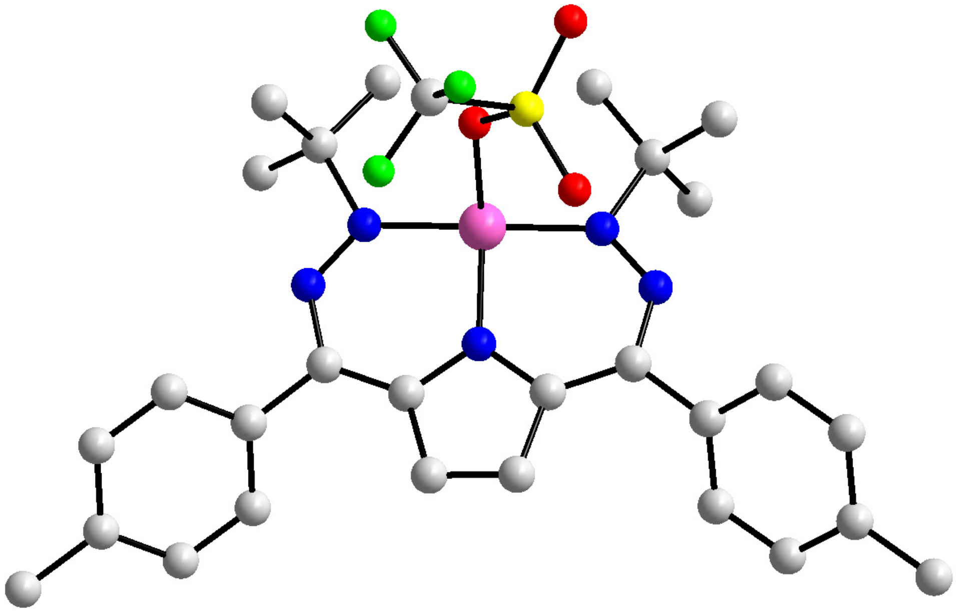

Figure S36. Calculated structure of **2**. All C–H hydrogen atoms have been removed for clarity.

Table S5. Calculated coordinates of **2**.

Co	6.232169	12.540735	5.268708
O	6.367346	14.480118	5.259589
N	4.723067	11.442324	5.260356
N	6.441905	12.324277	7.129481
N	5.629833	11.819681	7.994261
C	3.977021	11.113710	6.371614
C	2.724544	10.542694	5.941880
H	1.919072	10.238673	6.602740
C	4.422581	11.307987	7.684986
C	3.618622	10.895997	8.861350
C	3.056276	9.611654	8.972265
H	3.222940	8.879820	8.179401
C	3.438774	11.794830	9.925209
H	3.868043	12.795167	9.853591

C	2.702337	11.425153	11.050668
H	2.563857	12.146209	11.860745
C	2.326689	9.248835	10.102975
H	1.912100	8.239625	10.170661
C	2.130335	10.149252	11.162465
C	7.747639	12.692840	7.870214
C	8.770493	13.287645	6.911743
H	9.013778	12.585030	6.103117
H	9.700026	13.479497	7.466718
H	8.427372	14.237485	6.485915
C	8.308406	11.388602	8.463391
H	7.596747	10.941571	9.169352
H	9.246592	11.607569	8.994368
H	8.526010	10.659128	7.668136
C	1.335239	9.748654	12.378130
H	1.764653	8.854417	12.856642
H	0.295366	9.503089	12.110063
H	1.310309	10.555533	13.124025
C	7.423216	13.706414	8.978517
H	7.037907	14.646211	8.560814
H	8.347985	13.934098	9.530124
H	6.686416	13.293631	9.678961
C	4.576726	16.388226	4.837509
S	5.511004	15.424685	6.179294
O	4.462564	14.711848	6.946230
O	6.370643	16.421104	6.856179
N	6.378174	12.455860	3.383944
N	5.602805	11.885749	2.521844

C	4.007268	11.054611	4.150871
C	2.745875	10.498446	4.576481
H	1.959552	10.154983	3.911883
C	4.467557	11.232256	2.837383
C	3.743090	10.674004	1.670430
C	3.240990	9.358970	1.668506
H	3.399144	8.721152	2.540021
C	3.579615	11.443567	0.506364
H	3.969662	12.461883	0.485708
C	2.917954	10.925363	-0.605895
H	2.792580	11.549352	-1.494571
C	2.584848	8.846558	0.551204
H	2.216800	7.817628	0.572256
C	2.404123	9.619909	-0.606782
C	7.539638	13.116586	2.610260
C	8.698631	13.447248	3.545248
H	9.097990	12.537556	4.016704
H	9.510639	13.891585	2.951656
H	8.409798	14.173199	4.312190
C	8.045894	12.149061	1.527132
H	7.278339	11.944545	0.773151
H	8.921067	12.600986	1.037334
H	8.361338	11.193435	1.972138
C	1.688745	9.057808	-1.808236
H	2.186272	8.147343	-2.177029
H	0.652032	8.780507	-1.560152
H	1.656726	9.785065	-2.631501
C	6.985013	14.403323	1.978980

H	6.669823	15.110774	2.755739
H	7.770299	14.877945	1.370950
H	6.129486	14.173771	1.329777
F	3.730899	17.243682	5.415651
F	3.882126	15.542147	4.064106
F	5.432616	17.074253	4.068344

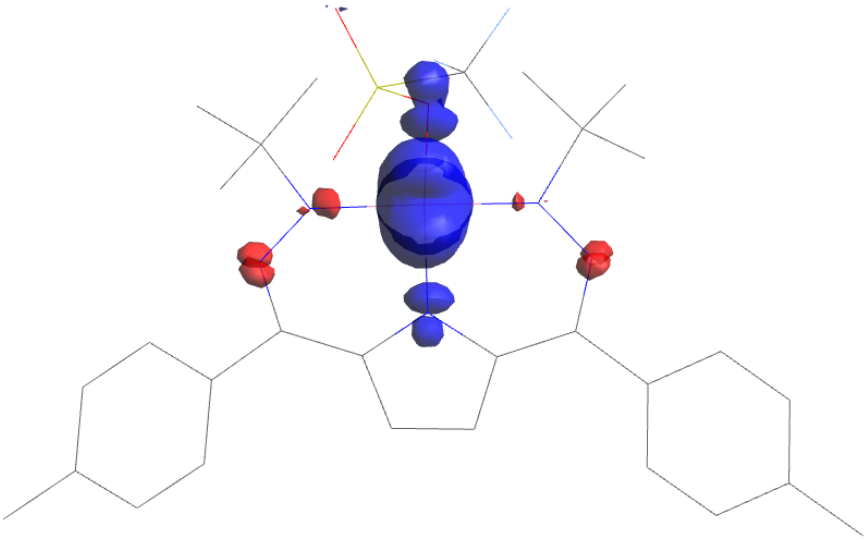

Figure S37. Spin density plot of **2** at an iso value of 0.005.

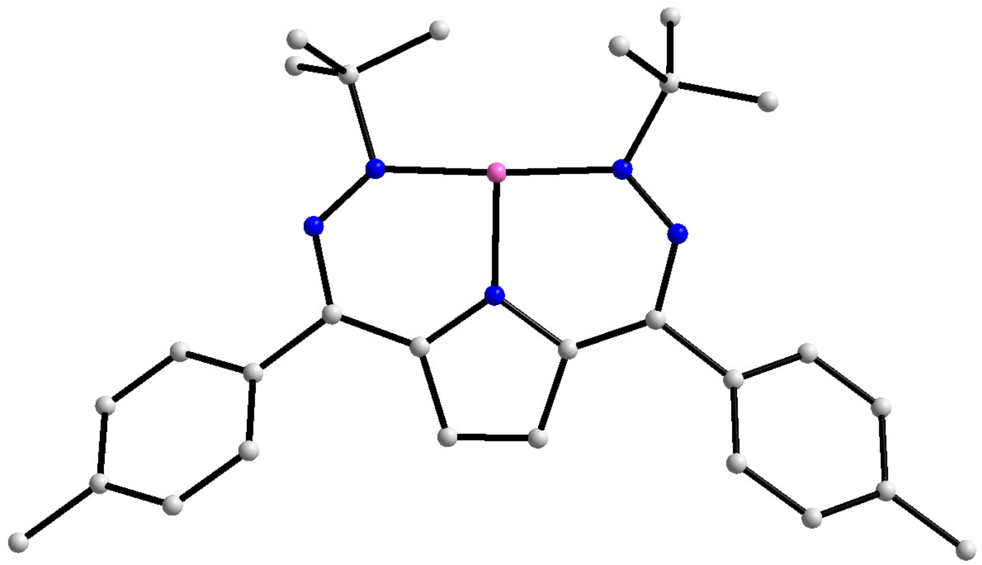

Figure S38. Calculated structure of [$^{tBu,\ Tol}$DHPCo]$^+$ (**2**$^+$). All C–H hydrogen atoms have been removed for clarity.

Table S6. Calculated coordinates of [$^{tBu,\ Tol}$DHPCo]$^+$ (**2**$^+$).

Co	6.39844868269788	12.23181322828487	5.35649781138017
N	4.75817748075237	11.42876877764973	5.35878048722366
N	6.34690981319396	12.37464941428733	7.17882957784680
N	5.59550055202483	11.92183864237038	8.08397355862211
C	4.03456773847263	11.10042081052333	6.46440099271619
C	2.73125517747985	10.67979517795477	6.04081548856934
H	1.92787208953704	10.40401501749954	6.70603004710218
C	4.48140468911355	11.23929750347771	7.77830630479577
C	3.75401623981483	10.69393733654292	8.92492698597566
C	3.16924068064993	9.42881278349186	8.88228330175910
H	3.26243964960653	8.82255895372871	7.98660865509988
C	3.66826653478845	11.41907545052069	10.11085865276530
H	4.12624550110158	12.40125147082288	10.16722039818357
C	2.99982144516995	10.90574947776210	11.20277499025954

H	2.93514769689698	11.49524645704269	12.11336197328555
C	2.50613290242917	8.92215984140853	9.97943542811216
H	2.06825074084207	7.92857993033013	9.92596597574720
C	2.40310325743712	9.65063426480654	11.16054801314924
C	7.49254790626826	13.19179161211051	7.62377820786494
C	7.98061349625413	13.74451812432274	6.30031733758019
H	8.16068283126843	12.90337332591747	5.57529463633582
H	8.98084018255119	14.18828990760727	6.36186361032528
H	7.29670404427542	14.50074463314383	5.89144319025972
C	8.53198635786890	12.30016932120453	8.27733108276924
H	8.10236845066493	11.82127104464957	9.16360850722092
H	9.39185575686743	12.90751131193293	8.58365366945925
H	8.87717394476708	11.52326856589990	7.58349910372219
C	1.68406357292156	9.09260237262077	12.33970504551253
H	2.12892313643873	8.13616087364251	12.64698335756783
H	0.63123990288646	8.89647855642049	12.09291360147585
H	1.71968227514696	9.78297410843354	13.18805009972026
C	7.04665160677932	14.30196618201388	8.55182985464214
H	6.24675437542475	14.89310384102300	8.08999825075764
H	7.89930322569742	14.96047458829591	8.75659830691735
H	6.68629191753509	13.88659496702621	9.49763200561745
N	6.58486887937870	12.07567369072583	3.48243581355350
N	5.69362111645960	11.74520726679843	2.64909054855695
C	3.99017474033347	11.19709130971814	4.26011804785292
C	2.69736242019195	10.75626876385327	4.69434562115360
H	1.86239911623502	10.54930847584128	4.04413117160405
C	4.44670594132855	11.35563402670531	2.95972215334892
C	3.61807308141397	11.04041707711214	1.78798381604131

C	2.88461957579888	9.85937934679113	1.69692244096269
H	2.92494283805149	9.13539585538832	2.50471988254936
C	3.59393928325600	11.91370585565096	0.70539038546048
H	4.16970363354819	12.83309455159337	0.74997582551353
C	2.83983389676128	11.62833506263231	-0.41491386550271
H	2.82779840595471	12.33316627322860	-1.24207671185992
C	2.13991405296411	9.57734830839284	0.57090942250346
H	1.58856665018416	8.64185671563896	0.51806393957566
C	2.09710869626150	10.45717494150350	-0.50575143538277
C	7.91593196603115	12.30986739996152	2.79128480417418
C	8.24560988160639	13.79466447680425	2.84474770912836
H	8.38656417297046	14.17264078874822	3.85873140373811
H	9.17799580328937	13.96605165087695	2.29428860241582
H	7.44781093204058	14.37080809534183	2.36070490730320
C	8.95936164353042	11.44846890528448	3.48843236496632
H	8.68622145087343	10.38906455728446	3.40841197909866
H	9.92806860607240	11.59586714951683	2.99780125626314
H	9.09124702228042	11.69051350805591	4.54719759878914
C	1.28983682202691	10.14064518623280	-1.71736737909048
H	1.60883512024572	9.18519700548679	-2.15597697841549
H	0.22645944295659	10.04070073234485	-1.45869765966054
H	1.39056722477855	10.92365141648770	-2.47527542445818
C	7.86075742863559	11.88831092598401	1.33414655377984
H	7.16004576696354	12.50098442338895	0.76087338285706
H	8.86597302704691	12.01869291098445	0.91698703629417
H	7.57048750490438	10.83770547086988	1.23176427654263

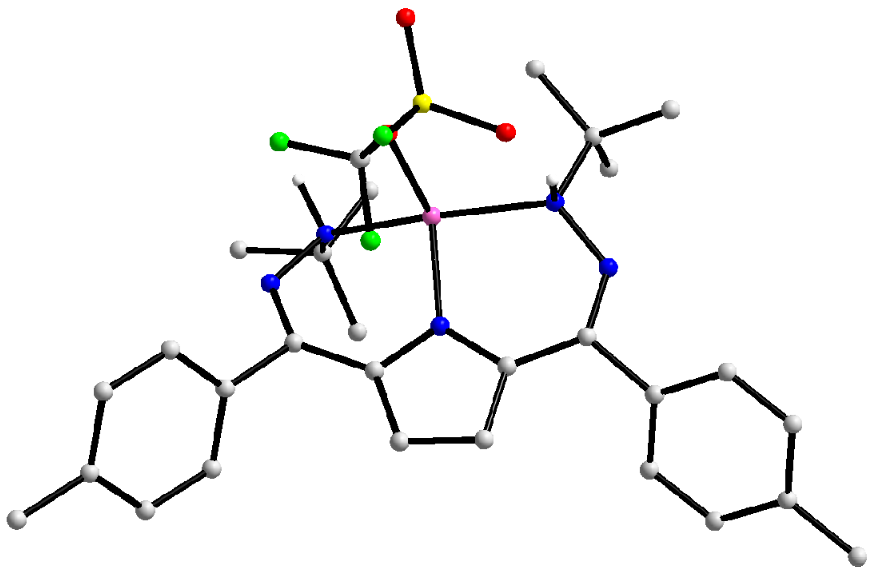

Figure S39. Calculated structure of **3**-HS. All C–H hydrogen atoms have been removed for clarity.

Table S7. Calculated coordinates of **3**-HS

Co	6.13855685228539	13.15382170874999	5.14644054394217
O	5.86089129146602	14.97445355687082	4.46680411519950
N	4.82357944686116	11.77768721636345	5.20554252279662
N	6.21449946055430	13.27570155792147	7.22789370588724
N	5.62978006476824	12.27728738396605	8.07816179589761
C	4.12610180517533	11.35881910683807	6.30391675843532
C	2.94282740009537	10.70921636242146	5.87263330951194
H	2.18079726796599	10.27193669163529	6.51100611235357
C	4.65733673291024	11.51588543660604	7.65312801370595
C	4.06812777862743	10.66487929390123	8.73107150600371
C	3.82922276477275	9.29342008870913	8.54713629846197
H	4.07009690481564	8.82649748852210	7.59067593391046
C	3.78047697704622	11.22672861894498	9.98558082846402
H	3.96937313041912	12.28982113072190	10.14133403541575
C	3.25903264018140	10.44550906188613	11.01449665603992

H	3.03428061910439	10.90948667747022	11.97858925467630
C	3.31724336496960	8.51495044194372	9.58542939415563
H	3.15445347925154	7.44622266025588	9.42183666612228
C	3.01536022209384	9.07486778769015	10.83575781956469
C	7.42416036625873	13.86302499060931	7.94983563093033
C	7.99799401896037	14.96691445989635	7.05750960889109
H	8.28376051945342	14.57897494157189	6.06663460698551
H	8.90203272156495	15.38087943711862	7.52649279593828
H	7.28461707252066	15.79122417430937	6.91637906516377
C	8.44687921361750	12.74298374918666	8.15129960386163
H	8.01775690254579	11.92990848587464	8.75190331546175
H	9.33192417806652	13.13774356989529	8.66997306897357
H	8.77464549343434	12.32967970567760	7.18499171712924
C	2.42462163093185	8.23935290089983	11.94147604522850
H	2.71431033706568	7.18319258879064	11.84497416757068
H	1.32210074419693	8.28235175209381	11.92084464015442
H	2.74611776339335	8.59837428294958	12.92977570256181
C	6.99050468327724	14.45614180924392	9.29862947903549
H	6.23391982332565	15.24229090554663	9.15164978649176
H	7.85760838201576	14.90717672537798	9.80315999801822
H	6.57179914441401	13.67574681474622	9.94695902687977
C	3.39707021681910	15.96835761944053	4.09574510933516
S	4.95257641956789	16.02213248063014	5.18526548724121
O	4.50872163242369	15.51694387195342	6.52415048732441
O	5.48213464679883	17.39245067204568	5.08204457355638
N	6.81775523577805	12.54995435747922	3.27866135829986
N	5.75035725045382	12.20967005094199	2.32696237883774
C	4.13255999970998	11.40588789664939	4.08012790444529

C	2.93842941893017	10.75399828107516	4.47490279487680
H	2.16698796429339	10.36719125574948	3.81643479888157
C	4.61886912938023	11.70344144394332	2.74243046220290
C	3.68742515473162	11.42261994914950	1.60116940014659
C	3.22537464851394	10.12576826579350	1.32796789173951
H	3.54213203983734	9.29258637986002	1.95855544122972
C	3.28752803156964	12.46963839905638	0.76055342588294
H	3.65266999847315	13.47879812223115	0.95647160918569
C	2.43267330181743	12.22642578806165	-0.31543397815338
H	2.12384030430647	13.05897899540058	-0.95275346258396
C	2.38045795411935	9.89003323398972	0.24388151667412
H	2.04143511986751	8.87035732442475	0.04249938603085
C	1.96173157405803	10.93593348425729	-0.59411121470025
C	7.99290044637623	11.58835199240079	3.11022576724534
C	9.14626929194199	12.19507209252913	3.91513576666440
H	8.88776214123296	12.26879567133144	4.98268410044624
H	10.04107102606652	11.56299480328439	3.82327092647921
H	9.40765740825711	13.20066523616730	3.54925202732493
C	7.59873315515768	10.22009657142743	3.66921073784305
H	6.75312674495945	9.79483120669690	3.11118674221188
H	8.44765473403534	9.52719398601665	3.57712099946954
H	7.31632988921190	10.28861388937537	4.72929699252403
C	1.03060024544433	10.67410196674365	-1.75008996973331
H	1.42494208142074	9.88560764603051	-2.41068930656566
H	0.04417972849318	10.33485810328583	-1.39562962003743
H	0.87789157891117	11.58010154793662	-2.35258935680144
C	8.38636834015225	11.48272336274825	1.62888667846097
H	8.66144394591990	12.46816914256883	1.22217430780910

H	9.25782131810630	10.81902953783993	1.52725019616564
H	7.56162799292027	11.08269905654767	1.02677839713661
F	2.48989810101636	16.82506538147191	4.56774265388646
F	2.87818669161050	14.73317390298983	4.09869044045000
F	3.71230552992990	16.30466076283273	2.84101448199417
H	5.51009753534099	14.05465767797202	7.16575977715981

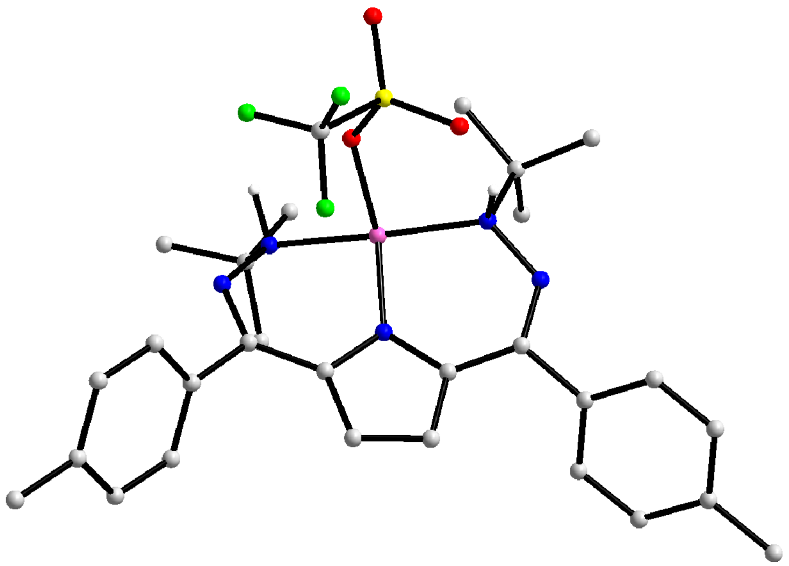

Figure S40. Calculated structure of **3**-LS. All C–H hydrogen atoms have been removed for clarity.

Table S8. Calculated coordinates of **3**-LS

Co	6.17834248041589	13.01396628736217	5.22831349603865
O	6.33218570194490	14.89005621493014	4.74382450213186
N	4.92652423594608	11.64990259775651	5.20643883629715
N	6.06468034923774	13.29176032870956	7.21538514573846
N	5.36959033218685	12.41191648211038	8.11775760111712
C	4.25864535365903	11.14771095139323	6.29644532209245
C	3.17636403582115	10.35311550387594	5.85620382850856
H	2.45820834638614	9.84768835361723	6.49424083031762
C	4.57641390799207	11.48689864850748	7.66884622721493

C	3.90793530261423	10.70918188827174	8.75760857090301
C	3.88595446812435	9.30435538135615	8.75402888302797
H	4.34731173683712	8.75815698625466	7.92931106881122
C	3.32934493667666	11.38209780116073	9.84308587284331
H	3.34698227762583	12.47311566045405	9.85837192128538
C	2.73476742604342	10.67187511480590	10.88631782233728
H	2.27877216467399	11.21927699978586	11.71548760543133
C	3.30024992546317	8.60171491282971	9.80587064477657
H	3.30704445720249	7.50810350259229	9.78783498477288
C	2.70817888712896	9.27020230895837	10.88969504451200
C	7.39467489801564	13.62742152299776	7.94219257398703
C	8.20208409844907	14.64634360570026	7.13382592819068
H	8.45041004438734	14.29137388352979	6.12677265828150
H	9.14700815693995	14.83238402753159	7.66549488325842
H	7.68098208363266	15.60818266639879	7.04473896123314
C	8.18248473504454	12.32359871822633	8.09746606655021
H	7.62812952922257	11.60160830749553	8.71159880112880
H	9.14724595376843	12.52776079077155	8.58379452152874
H	8.38716307441116	11.86497533129510	7.11727999663357
C	2.06840694938889	8.49935623065690	12.01555654770085
H	2.79971006202760	7.84440760564487	12.51618712497687
H	1.25643943570807	7.85311787587271	11.64584389903275
H	1.64584252692642	9.17423457666155	12.77262634509395
C	7.07545363163384	14.23922146732341	9.31610479518874
H	6.49032339262450	15.16534978951018	9.20451102096880
H	8.01795803052287	14.49692119581393	9.82082794829162
H	6.51336974313978	13.53772463820628	9.94295833718095
C	4.02628198002736	16.10047570897134	4.12144441723480

S	5.52375511255809	16.11112557203842	5.29356366617885
O	4.95400684541859	15.84331560539566	6.65002083320776
O	6.22843446925225	17.38054534113418	5.05128103432604
N	6.57637046957426	12.76009969413378	3.29404120233861
N	5.57405676519025	12.38211099888970	2.32224658306499
C	4.27426965917665	11.20001425208351	4.07283744634087
C	3.17667386407432	10.40055552795834	4.46390839197989
H	2.45772031657495	9.93746534730238	3.79616511610272
C	4.59024041305414	11.63207816351568	2.72897008445496
C	3.65068132939546	11.25888967626819	1.62479735787844
C	3.37919781803856	9.91894503332833	1.30573246376565
H	3.85019649352516	9.11733626754389	1.87791453016887
C	3.05697206779617	12.26561464718111	0.85222462502688
H	3.27580435378130	13.30989377066094	1.08203869273918
C	2.19698662766531	11.93983324762184	-0.19742394219873
H	1.73649128535375	12.74184892419723	-0.77973595384846
C	2.52758840427243	9.60237304425953	0.24796360541887
H	2.33887151760967	8.55193223812788	0.00978286264986
C	1.91369382902116	10.60517939724631	-0.51915854221398
C	7.88962141520630	12.02423729488698	2.96836110764099
C	8.96996402547465	12.63784144003757	3.86324984081326
H	8.77408280181642	12.43106951296183	4.92700315098686
H	9.94813784979718	12.20273825848667	3.61086004950908
H	9.04172240149055	13.72805382568084	3.72473787706193
C	7.72264181685570	10.53423875193761	3.26707370995712
H	6.95263631262729	10.08339534645674	2.62642227362953
H	8.67214867321978	10.01439168140369	3.07281682936778
H	7.44470991165792	10.36634334752119	4.31747989585830

C	0.98260512865393	10.25009042051640	-1.65026910512871
H	1.48495613365991	9.61173150457198	-2.39481269763443
H	0.10679330780060	9.69189823481889	-1.28266728334082
H	0.61758561089424	11.14933946724133	-2.16417753405911
C	8.24954460158875	12.24978884516054	1.49182668803543
H	8.34172515712743	13.32349095809678	1.26613674635180
H	9.21889272543446	11.77563235699990	1.27773375987737
H	7.48903316984351	11.82514723009227	0.82634011023413
F	3.23055863500596	17.13113490912782	4.40844801775004
F	3.33747790935251	14.95979180349146	4.26437309128005
F	4.44048533466076	16.20055793184086	2.85358410572649
H	5.51761062050948	14.18360516460111	7.22739408617533
H	6.76194016413943	13.74617109784044	3.05091218790708

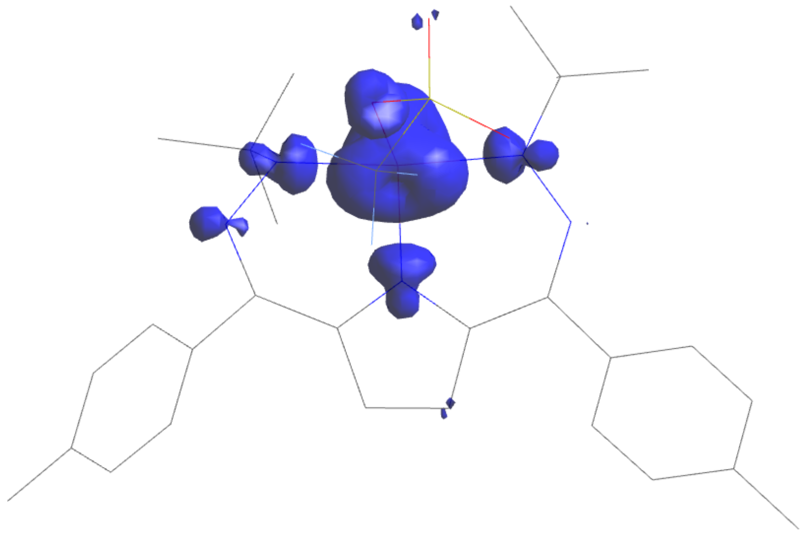

Figure S41. Spin density plot of **3**-HS at an iso value of 0.005.

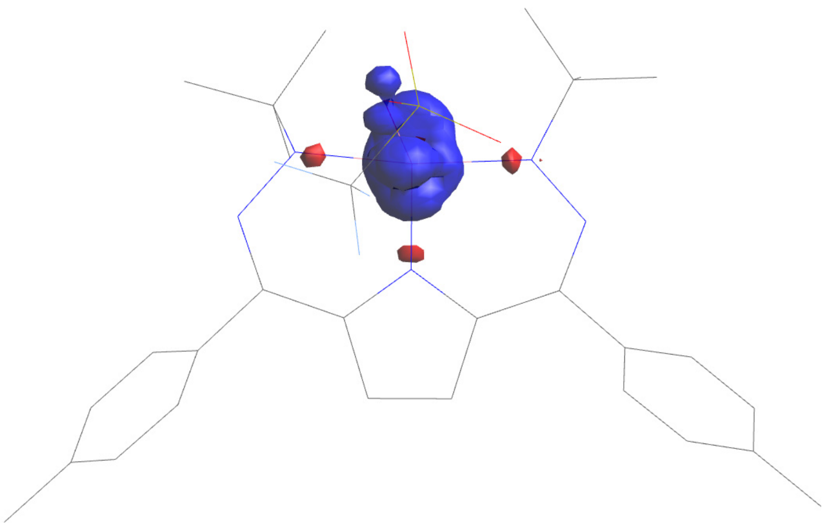

Figure S42. Spin density plot of **3**-LS at an iso value of 0.005.

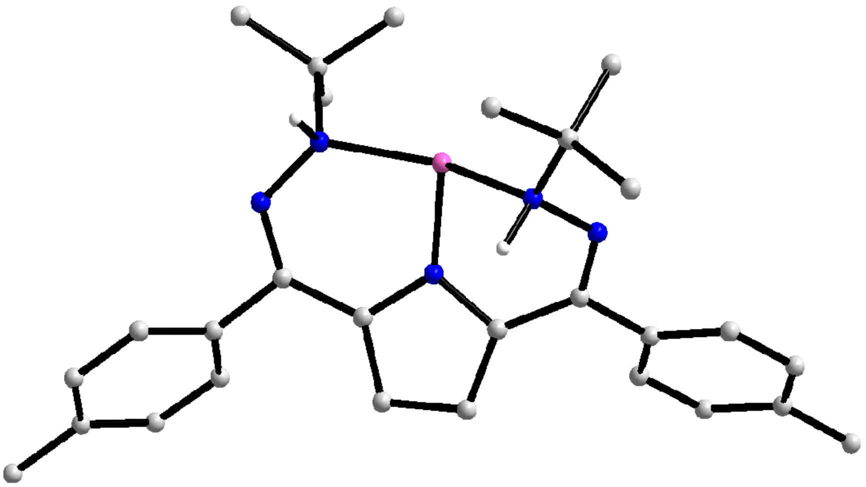

Figure S43. Calculated structure of [$^{t\mathrm{Bu},\,\mathrm{Tol}}$DHP-H2Co]$^{+}$-HS (**2-H$_2$$^{+}$**). All C–H hydrogen atoms have been removed for clarity.

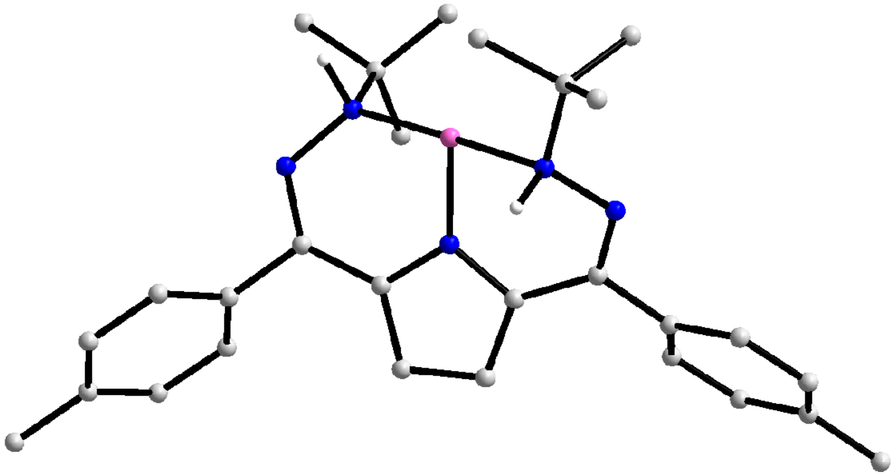

Figure S44. Calculated structure of [$^{tBu, Tol}$DHP-H$_2$Co]$^+$-LS (**2-H$_2$$^+$**). All C–H hydrogen atoms have been removed for clarity.

Table S9. Calculated coordinates of [$^{tBu, Tol}$DHP-H$_2$Co]$^+$ -HS (**2-H$_2$$^+$**)

Co	6.70301364928555	12.04911148844683	5.40704772924566
N	4.92853592924737	11.47077788633519	5.30588291697293
N	6.25220570098277	13.05258110377191	7.08836822663526
N	5.79445053740508	12.09440498104105	8.01247482466980
C	4.12556395282810	11.37421149976109	6.39730880911723
C	2.80208996392599	11.29587871200284	5.98083007164300
H	1.93278808056399	11.22093945544625	6.61733976944502
C	4.74186740764292	11.39602596860032	7.71378785688195
C	4.19256349509799	10.56143418385422	8.78597784019320
C	3.49893873582914	9.38801905229269	8.50072738467182
H	3.35557519855243	9.08537697216740	7.46862685019064
C	4.37626477744829	10.91354082480632	10.12273055486578
H	4.90767349379276	11.82956286559699	10.35793120527906
C	3.88328709807105	10.11759570686346	11.13203994223309
H	4.03143103680396	10.41392638487995	12.16730875966350
C	3.01338841594766	8.59320513777853	9.51874926295099

H	2.48713051987602	7.67461632850107	9.27300040563129
C	3.18983245783289	8.94235253605745	10.85233494232166
C	7.21325339940537	14.00969125281083	7.72375836652121
C	7.59789241332050	14.99708608023985	6.63969054984075
H	8.14211854001987	14.50371686383565	5.82274999838782
H	8.25494161597496	15.76449320526892	7.06246799985967
H	6.71210035516067	15.49770243395448	6.22568136236824
C	8.42212754916847	13.24185621597962	8.21346820850825
H	8.13488546109169	12.51183829775205	8.97607683689861
H	9.14080408940421	13.94429542375082	8.65013255344326
H	8.92089295834935	12.71806300106477	7.38737443905043
C	2.63610533227178	8.10052058997911	11.94854038802068
H	2.52824384199310	7.05795592266870	11.62999989690490
H	1.64010552558891	8.46562920195621	12.24081769056832
H	3.27573389234188	8.14034512823774	12.83776555259666
C	6.52089277177206	14.72569931636243	8.86933424184868
H	5.64702931778818	15.28328376979532	8.50650127823168
H	7.21605627330007	15.43667820248705	9.32913804490602
H	6.19533179074370	14.00824947258608	9.62929374187037
N	6.90169694612804	12.21694219425472	3.41526731950229
N	5.86511294711681	11.98476093308671	2.46764010842476
C	4.15152330806688	11.46939215802053	4.19127506047735
C	2.81695591704132	11.36824783572189	4.58948885824720
H	1.95580294988714	11.36979813228544	3.93900482131470
C	4.68028613398978	11.64595019575257	2.85412243142757
C	3.74185178231749	11.44754220977285	1.72684239083406
C	2.95145086948797	10.30523936762667	1.64177634913699
H	3.00093632966252	9.55490832410056	2.42463469170778

C	3.67387256446589	12.38011594650329	0.70042232426661
H	4.28983014808640	13.27190559500499	0.75282739482046
C	2.82789924823275	12.18252587164787	-0.37325929390689
H	2.77921665540551	12.93223093535943	-1.15861028979969
C	2.12191999766695	10.10710310315702	0.55772250342287
H	1.52480658332283	9.20023647758995	0.50250927546159
C	2.03842672009004	11.04350010941864	-0.46802784910260
C	8.10029929767916	11.33793203171455	3.11654014979717
C	8.97835806648808	11.47684234196036	4.34779476288267
H	8.51502911627760	10.99957162662883	5.24655682689505
H	9.92217840537393	10.93314123735272	4.21815902466058
H	9.22259169397913	12.52673160039432	4.56304463035937
C	7.64702102852956	9.90259410926216	2.95297800401927
H	7.05130855209394	9.78548576879414	2.04338965187719
H	8.53136085236556	9.25965951145314	2.87955011328575
H	7.04932255711656	9.56771118488884	3.81100400951981
C	1.12850898112944	10.82393742054755	-1.62751482403389
H	1.37562677288988	9.88532639372269	-2.14231348822508
H	0.08519878084110	10.74526953219734	-1.29156098709204
H	1.19985123687754	11.64532426847818	-2.34734907159967
C	8.81408264935459	11.83424830134490	1.87707230326517
H	9.18975632009720	12.85623649970219	2.02568916953926
H	9.66717350624324	11.18345557190415	1.65190027802393
H	8.12873667845576	11.82368894045897	1.02289685128491
H	5.44642678712797	13.61084114980979	6.75977164456399
H	7.22005403728200	13.16953765314743	3.18970635230342

Table S10. Calculated coordinates of [$^{tBu, Tol}$DHP-H_2Co]$^+$-LS (**2-H$_2^+$**)

Co	6.46466792765504	12.74700101845773	5.37422130448439
N	4.98487234998561	11.69346440359125	5.29312343494659
N	5.96182123900051	13.26682135094137	7.14463098778996
N	5.59337304021710	12.32832469771175	8.11630694753958
C	4.27400803977419	11.26889247507610	6.37070946911046
C	3.07852248765384	10.70274743925948	5.91584178471129
H	2.30123929407046	10.28864148412840	6.53974659732498
C	4.73961805255138	11.43464162887199	7.72845618738609
C	4.24032336778104	10.53289536347676	8.77565365930423
C	4.01648780306492	9.18298368967842	8.52223907837954
H	4.19829759633254	8.78417889394972	7.52962472246436
C	4.02234129510202	11.01015419093692	10.06582556469346
H	4.19250653846318	12.06090956065220	10.27607762674358
C	3.58664551394730	10.16444816874815	11.06288932558826
H	3.41240124734925	10.55953871484218	12.06049487764507
C	3.59097220526416	8.34045796171989	9.52936502056144
H	3.43814629259018	7.28640733412376	9.31322471258568
C	3.35946098233628	8.81355712402097	10.81546424761419
C	7.08800264554559	14.16423400249002	7.54790502182184
C	7.58469389466210	14.66045610604306	6.19492592508616
H	7.98472334715800	13.82201286238210	5.55225777598501
H	8.48078363492343	15.28322322517271	6.30192386633363
H	6.83021428837973	15.25237904456033	5.65810616380125
C	8.16261236608690	13.36723169845961	8.25360005950992
H	7.80732637232128	13.01975311423306	9.22688779623026
H	9.03592380183552	14.01236823258930	8.40155722081107
H	8.47074923789530	12.49566885144972	7.66410004504021
C	2.86389727800469	7.91058064195814	11.89158019129392

H	3.10382898829450	6.86478023589379	11.67127737544565
H	1.77057364746711	7.99262670785393	11.98314332202099
H	3.29642320186962	8.18078467081703	12.86238727966090
C	6.58765342178789	15.29907678573193	8.41287187207886
H	5.83322290609700	15.89588920863235	7.88236721349863
H	7.42039148917318	15.95795572250239	8.68626234637320
H	6.14486249023424	14.89768484461873	9.33190830103396
N	6.92085930676009	12.30606958902474	3.52999772717269
N	5.91843374106198	12.22109784686776	2.52731339351224
C	4.28203373477177	11.41829932899814	4.16923144269188
C	3.07036573068858	10.82042369998350	4.53889159150123
H	2.28686331757959	10.50783476157534	3.86725885727540
C	4.75769507177415	11.76372589284729	2.85263618247556
C	3.82145532304411	11.64123302483551	1.71273274591692
C	3.25005490165809	10.41922338046365	1.37526909936544
H	3.47932076032473	9.53358299947906	1.96008643658068
C	3.53552859210236	12.74936813779442	0.92844541761124
H	3.98537019186734	13.70633107053185	1.17380383449542
C	2.68420026466826	12.64190383965223	-0.15472817543697
H	2.46509079534740	13.52468500630480	-0.74954748236910
C	2.41225676313779	10.31787804284129	0.28356882517696
H	1.98673200241145	9.35075026110046	0.02734807993967
C	2.10540030638168	11.42717058124884	-0.49779718601290
C	7.90176342052734	11.14206812898455	3.42799260236362
C	8.88900016422403	11.34560009651583	4.55996458524968
H	8.39444221493012	11.27359567828994	5.54360917428834
H	9.64721425602000	10.55492348650076	4.53039720874964
H	9.40579098961418	12.31055248227844	4.47849874369741

C	7.18861743361177	9.81812909690141	3.58627932876123
H	6.47792744838544	9.64658777103612	2.77278854322749
H	7.94014788357282	9.02149413880073	3.55136577993922
H	6.66324233682823	9.75184954336492	4.54408958303884
C	1.18436963636129	11.30658202587048	-1.66347245790746
H	1.53441938198900	10.53005913536796	-2.35700083886128
H	0.17724421023091	11.01712656638205	-1.33200751839579
H	1.10962453058564	12.25324413527727	-2.20769074252805
C	8.60943847180054	11.21422595404063	2.08762144823615
H	9.14143463107954	12.16909058992708	1.97644063413013
H	9.34543489148497	10.40462182298363	2.02451806349354
H	7.89528770640084	11.10635567790184	1.26569359385332
H	5.13809650830777	13.86857017625768	6.96784395684419
H	7.48284479559068	13.10157457419574	3.19781019702469

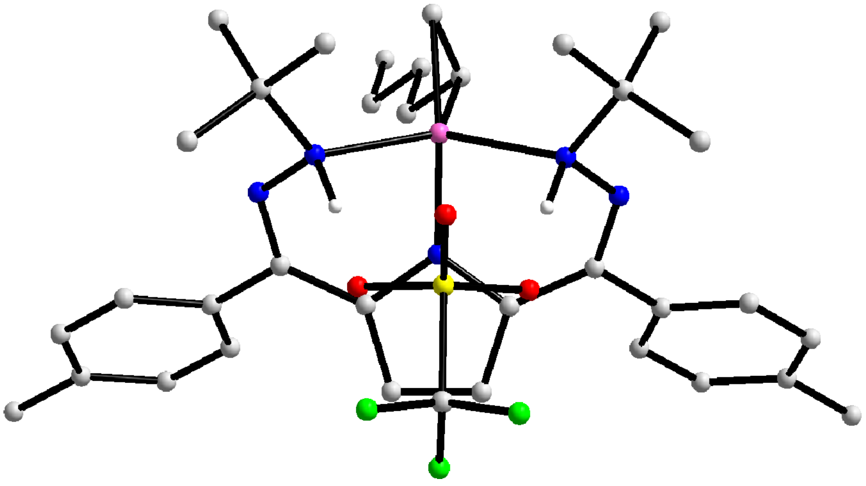

Figure S45. Calculated structure of a high spin **3-hexene**. All C–H hydrogen atoms have been removed for clarity

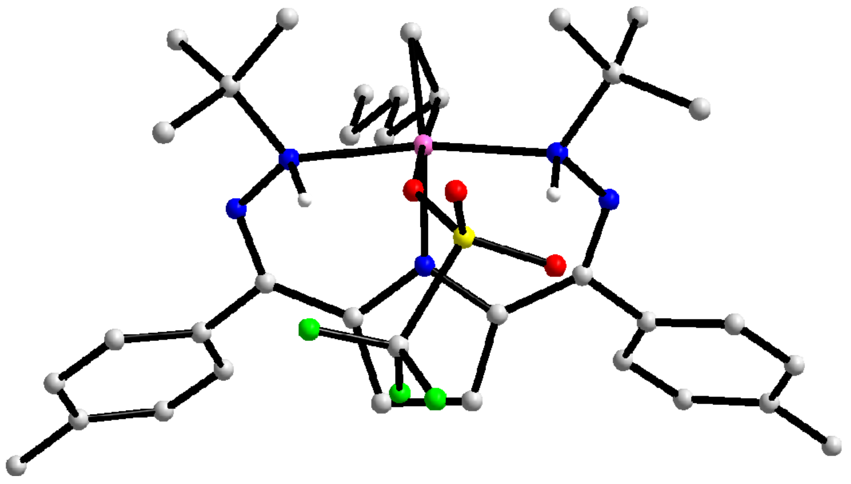

Figure S46. Calculated structure of a low spin **3-hexene**. All C–H hydrogen atoms have been removed for clarity

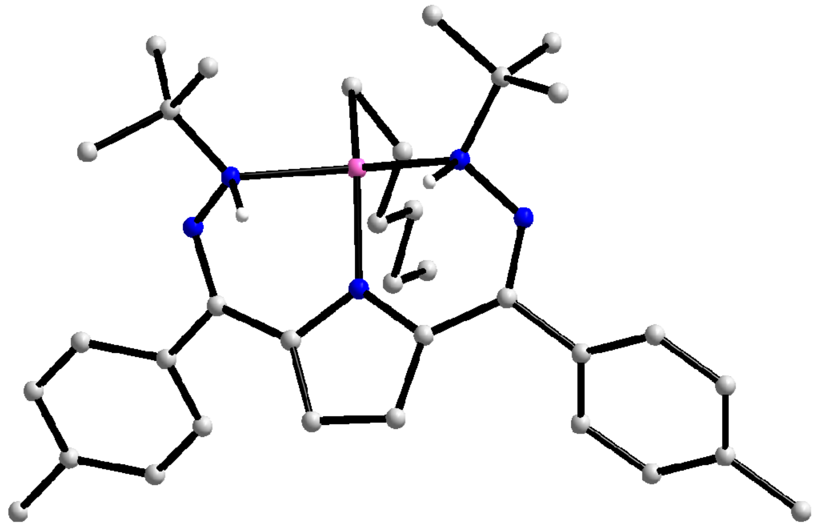

Figure S47. Calculated structure of a high spin **3-hexene**$^{+}$. All C–H hydrogen atoms have been removed for clarity

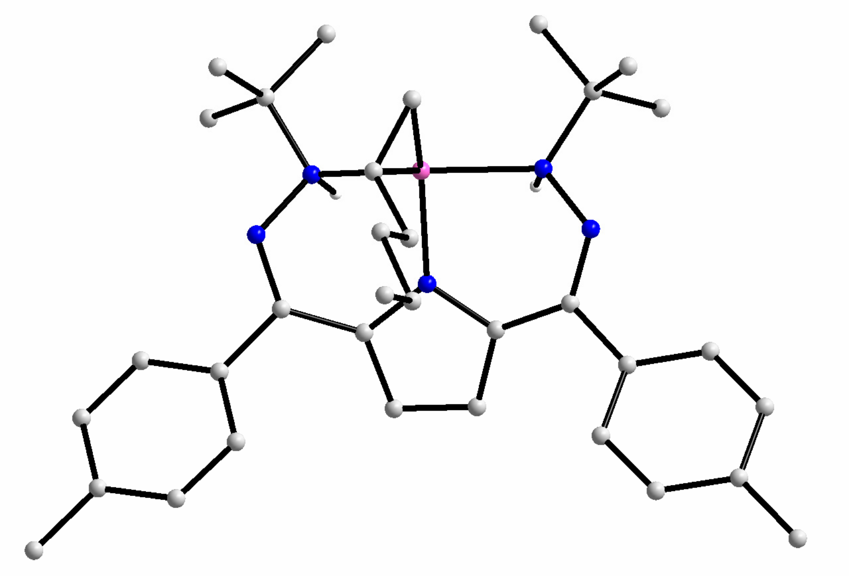

Figure S48. Calculated structure of a low spin **3-hexene**$^{+}$. All C–H hydrogen atoms have been removed for clarity

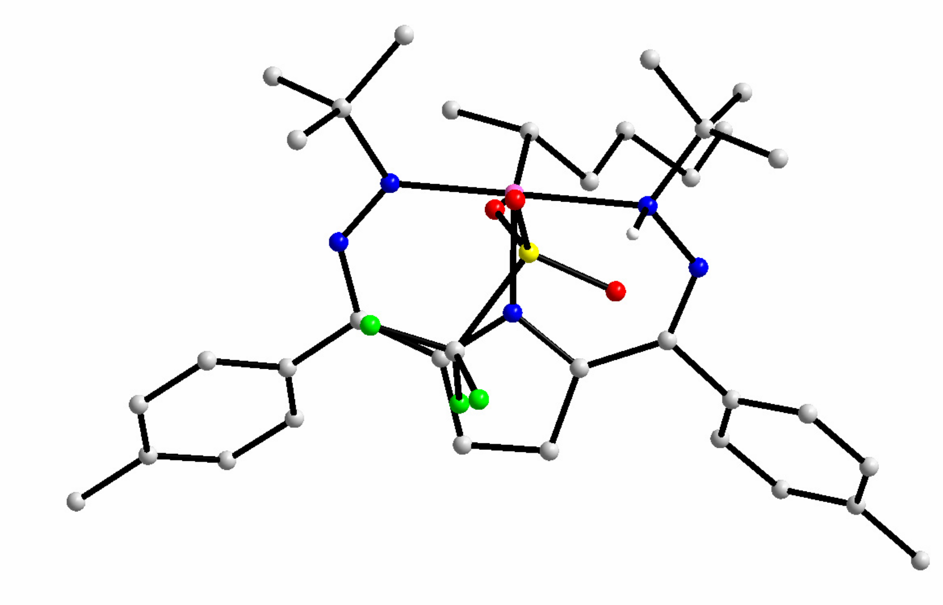

Figure S49. Calculated structure of a high spin **4-hexyl-α**. Most C–H hydrogen atoms have been removed for clarity

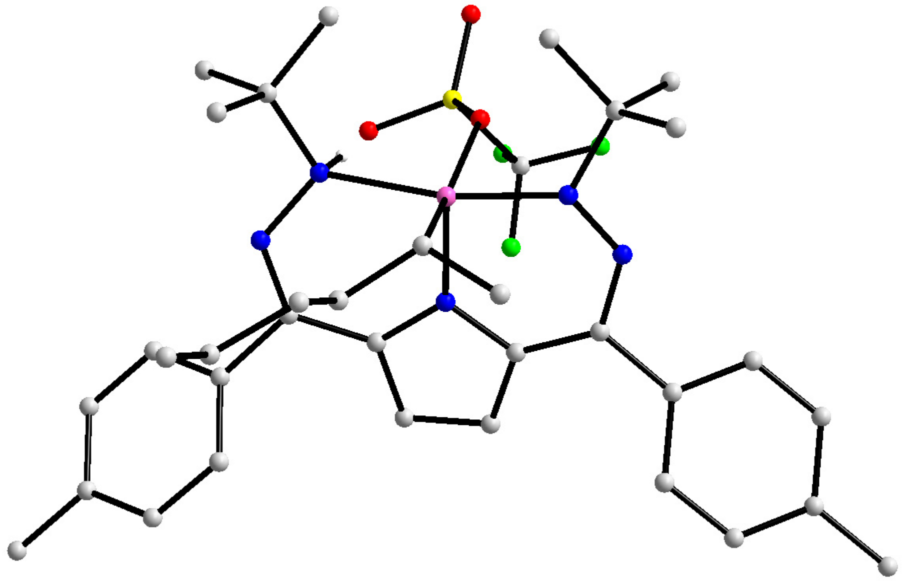

Figure S50. Calculated structure of a low spin **4-hexyl-α**. Most C–H hydrogen atoms have been removed for clarity

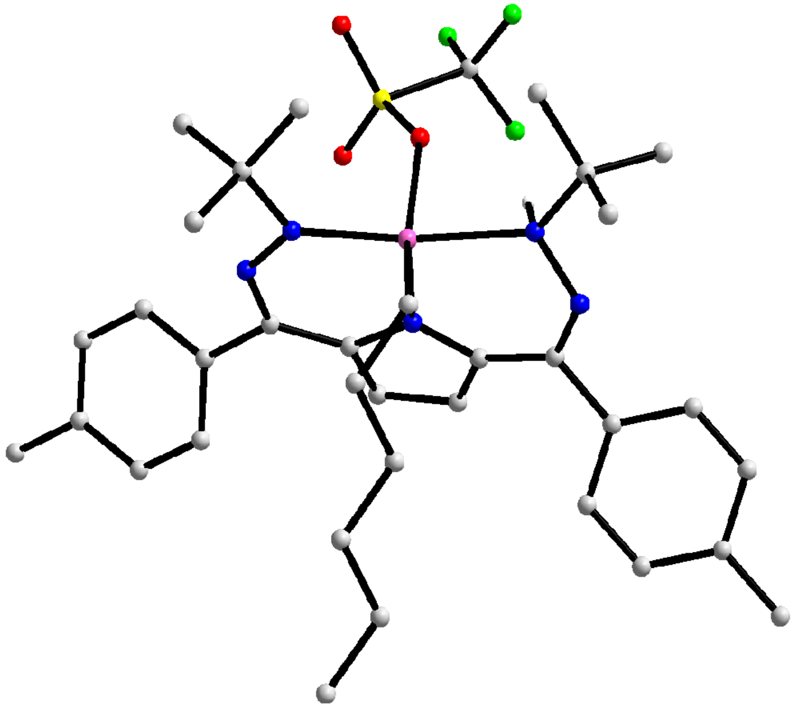

Figure S51. Calculated structure of a high spin **4-hexyl-β**. Most C–H hydrogen atoms have been removed for clarity

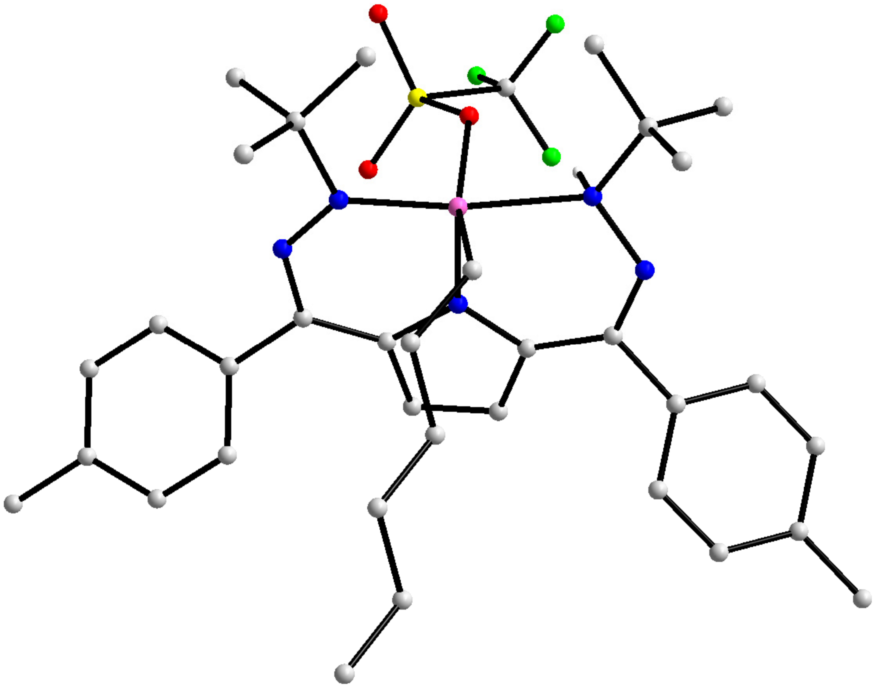

Figure S52. Calculated structure of a low spin **4-hexyl-β**. Most C–H hydrogen atoms have been removed for clarity

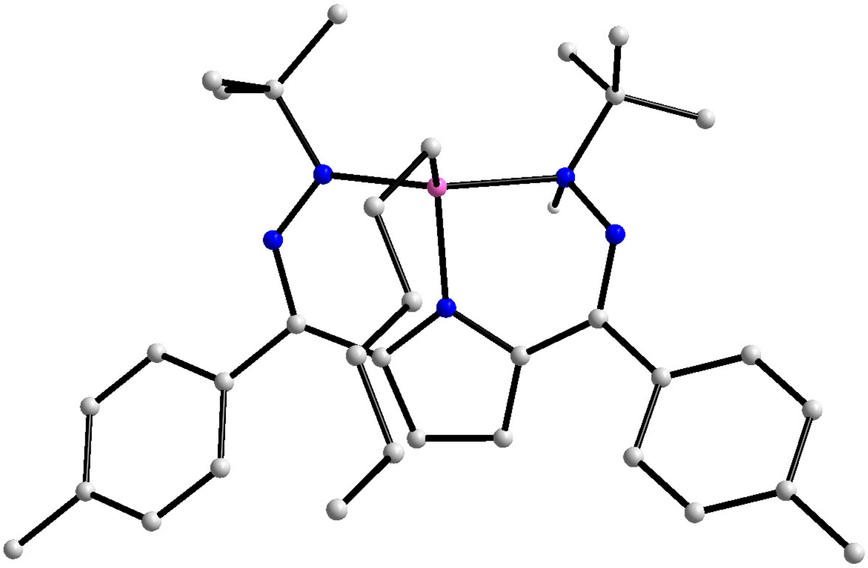

Figure S53. Calculated structure of a high spin **4-hexyl-β^{+}**. Most C–H hydrogen atoms have been removed for clarity

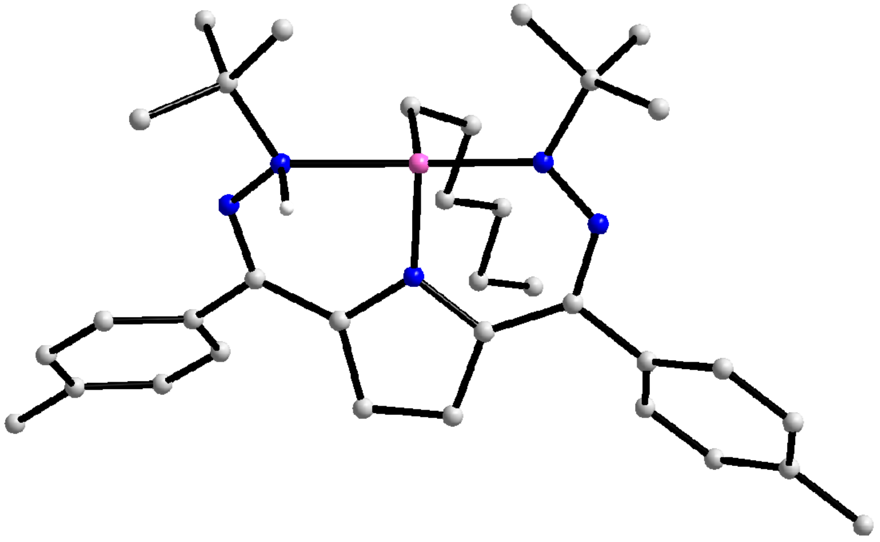

Figure S54. Calculated structure of a low spin **4-hexyl-β**$^{+}$. Most C–H hydrogen atoms have been removed for clarity

Table S11. Calculated coordinates of **3-hexene**-LS

Co	6.73436587163072	12.39108511306156	5.64628294460025
O	6.31478383844030	14.53916143843198	5.09424334878500
N	4.85819383347786	11.92750731373243	5.63067606152073
N	6.52754256415895	13.01647853972345	7.56122627117691
N	5.68157873634310	12.32999790725944	8.45354291064046
C	4.02007687407713	11.97108827051822	6.69523930168151
C	2.69927161924034	11.79754548908537	6.26187282415634
H	1.81951176242275	11.81450210668425	6.88671566971477
C	4.51335403421406	11.97095527926826	8.04915524425509
C	3.64049667774198	11.44828753366754	9.12292652254126
C	2.89544663243298	10.28349965744163	8.96538690203124
H	2.93540409961642	9.74665760586802	8.02337848986374
C	3.58196569867242	12.10710366606962	10.34483760018394
H	4.16300317337757	13.01343436870154	10.48102123004993
C	2.79314534699330	11.62401968330851	11.37086898351743

H	2.75197540619956	12.16470310110790	12.31294603420962
C	2.12023672690310	9.79859133159778	9.99857769703340
H	1.55739811736087	8.87886690214069	9.85765006555486
C	2.04700846543891	10.46240895126797	11.21897913346490
C	7.70865970711443	13.52518346316756	8.37218686439387
C	8.63956202990412	14.29857694087161	7.45923801220527
H	8.99403994549212	13.69699547417001	6.61765747073750
H	9.51576989773385	14.60622262664950	8.04123075537558
H	8.16377749152047	15.20126199436365	7.06471647235402
C	8.43159787798887	12.37491483734189	9.04486514882183
H	7.72644534913906	11.76465100584140	9.61642850956543
H	9.17152562043885	12.79326716355646	9.73684034205446
H	8.96551132927274	11.73702660777424	8.33669689269525
C	1.18674373450238	9.94257316019524	12.32055939791746
H	1.41103407967784	8.88724564493863	12.52636882538316
H	0.12447245968813	10.00198919236506	12.04347525989886
H	1.33400297635982	10.51780381439932	13.24036346712264
C	7.17560891710117	14.46745887307810	9.44430473714558
H	6.59910867667671	15.28479031835878	8.99657780758812
H	8.02586673468075	14.89843199839539	9.98562357207608
H	6.54271595959222	13.92713100232283	10.15395256323194
C	4.24063707155968	16.00027803858420	4.47857062443161
S	5.72176523104178	15.80339151770440	5.56528699841154
O	5.18065131747845	15.68363720854996	6.89935552190420
O	6.52407690359716	16.95631746266167	5.27836260929049
N	6.68759988114383	12.37682801975147	3.60330717651703
N	5.89907331300044	11.46921333459381	2.88352465926141
C	4.10649159654010	11.64329120317193	4.53681564139040

C	2.75397884440048	11.59046204288531	4.89920103003737
H	1.92578661048429	11.40968810751502	4.23121667058535
C	4.69860903639944	11.23782756321737	3.28752936653331
C	3.90355066008126	10.40325491069104	2.36050269659564
C	3.14345504796239	9.32734587805009	2.80785727581225
H	3.11126919322493	9.09933690244528	3.86824119423136
C	3.94310766566557	10.65809572802882	0.99480738690920
H	4.54015230046806	11.48896207160996	0.63284315729284
C	3.23421144848814	9.87174943291077	0.10777392662008
H	3.27148926773088	10.09842620813042	-0.95480505332774
C	2.44851781895002	8.53669522189207	1.91527032231025
H	1.87393558632060	7.69134940807902	2.28640232908554
C	2.47148460005467	8.79789686393366	0.54948301090724
C	7.88145724108404	12.73844745409889	2.73204120102987
C	8.88684486416336	13.53554920187410	3.53976009391725
H	9.36926963360865	12.92113387180473	4.30449470956196
H	9.66805948100369	13.89379470515380	2.86005118670962
H	8.42540409894927	14.40606527903251	4.01471571753788
C	8.53193184374457	11.49703340863413	2.15197215957101
H	7.85708483816628	10.98368007165122	1.46339304833941
H	9.42865719663281	11.80991670904292	1.60426676039510
H	8.84048772696934	10.79147028153333	2.92814511549586
C	1.68765066441730	7.95970142754479	-0.40275538526021
H	1.77205313484542	6.89517964228366	-0.14985018104041
H	0.62056582694446	8.22339010386788	-0.35992033222490
H	2.03042628348432	8.10706557487134	-1.43252373468069
C	7.35086307915303	13.60589466565959	1.59872777756711
H	6.94145024779432	14.54687045029834	1.98898652719986

H	8.16742178915203	13.84602917713288	0.90801820965121
H	6.56816768324682	13.07631377504764	1.04472270135137
F	3.60524806499149	17.12492193590170	4.78137319465607
F	3.39910622017152	14.98583544549841	4.62849774736667
F	4.60727004698048	16.05407705039409	3.19973361928987
H	6.01222719978478	13.87830900892647	7.29744524095465
H	6.15613082177338	13.26012388323058	3.67036640272338
H	7.99005975562204	5.33878226726789	5.70422879239339
H	8.17525563428205	7.89210583810503	5.87311138920307
H	8.71509894335587	10.80811527440452	4.80781675011714
C	8.33633783215902	11.07678364622180	5.78722485964794
C	6.91737394458489	5.44238778530756	5.91920049888274
H	6.51814183755084	6.83780942673373	4.33462150804780
H	9.04005322841976	11.57420571038565	6.44643277633010
C	7.09380537809355	7.95458120993635	6.07030613889258
H	6.73817196289004	9.37536782409292	4.48805918430455
H	6.39173951329118	4.60967821122936	5.43511377504985
C	6.39657797958743	6.77426371488487	5.42722064266006
C	7.24352397419134	10.41228544534690	6.27818606336320
C	6.57045736476459	9.29513800368661	5.56888873720282
H	6.78041149517430	5.34661230943006	7.00535944354662
H	6.96568427732728	7.90011021367299	7.16319827238625
H	7.02469156038610	10.48065088337084	7.34469184050163
H	5.31596312909946	6.84465929338273	5.62752528483507
H	5.48821854193999	9.33963431792361	5.74351238417480

Table S12. Calculated coordinates of **3-hexene**-HS

Co	6.84402632090437	11.83281680049117	5.66676984195459

O	4.99095732979976	14.85865917932512	4.10201252374904
N	4.93927004625487	11.70041091685179	5.60044587245366
N	6.65267179912370	12.96845886529669	7.41008617919078
N	5.79104703991429	12.34107268992714	8.32497258045456
C	4.08674541019708	11.87365645342121	6.63665709395744
C	2.77358343591387	11.84381083138421	6.15774884070853
H	1.87826025200879	11.98127800178164	6.74486141721246
C	4.60545645437685	11.96026762841909	7.97942524430241
C	3.75339293021741	11.51847706605930	9.10201744030672
C	2.88673200215740	10.43849567481074	8.98241832269124
H	2.81966634047969	9.90595410053988	8.03958329344999
C	3.82283010127641	12.17216222899222	10.33028226411622
H	4.49463761357571	13.01715377029292	10.43654549386388
C	3.04842549161166	11.76212586637427	11.39358764763893
H	3.11175049947420	12.29679548230297	12.33840990099576
C	2.11918688157531	10.02645570727167	10.05578158012569
H	1.45735590230229	9.17195515718400	9.93982555869648
C	2.17976360202008	10.68016555962415	11.27861835575324
C	7.80879359048586	13.53958072914164	8.18814722255916
C	8.74258759802979	14.20410202613589	7.20306714258399
H	9.22655982263967	13.46386381366808	6.55631760062501
H	9.52992217461814	14.72978848415012	7.75454287322317
H	8.21262093156020	14.93432093239977	6.58223780276028
C	8.52805411494402	12.42958831097195	8.92642139177266
H	7.85746218005832	11.93456930432749	9.63435866778529
H	9.36990485494446	12.86321771412156	9.47857664251255
H	8.93087312211683	11.68308101882838	8.23215581102907
C	1.33896919754064	10.25076425413215	12.43245704313652

H	0.77062703534514	9.34659358536865	12.19203474039666
H	0.62853331207930	11.04280638351250	12.70873869853298
H	1.96141387443217	10.05026025878145	13.31519694933218
C	7.28306842607047	14.57421059150353	9.17126986156340
H	6.77953793300763	15.39019019464263	8.63982733460793
H	8.12259817224685	14.99042637889526	9.74005652509667
H	6.57891518750322	14.11450257702585	9.87072139178690
C	3.34278785703072	16.54089049251341	5.18285309559503
S	5.06417790096594	15.87423867363598	5.13960284685006
O	5.24790068275857	15.33380837363161	6.47817364500875
O	5.88024469254219	17.01781054175334	4.82525767233951
N	6.76065580502678	12.55232696954202	3.70492500757658
N	6.09277671977014	11.59094614141939	2.93367659550279
C	4.20946745862044	11.52944566114699	4.47558648411486
C	2.85124786489587	11.62346804164494	4.79067269604747
H	2.02931142091247	11.55501223293697	4.09399024179257
C	4.89140771130150	11.22274700113477	3.24046381407248
C	4.22335660847733	10.36618018042044	2.24351128094271
C	3.34421733944258	9.35705922149785	2.62019250763505
H	3.13653743473472	9.19245600282352	3.67211999800131
C	4.47479183662604	10.54410438832168	0.88414235015017
H	5.15253646008660	11.33269983199737	0.57616371042782
C	3.86566861511212	9.74426030548997	-0.05744709341763
H	4.06725892850735	9.91238238675752	-1.11276813036985
C	2.74240605624642	8.55291862293596	1.67051453489342
H	2.06571147695447	7.76511221771135	1.99136260725360
C	2.98730943184863	8.73037016367183	0.31606835596613
C	7.85333066590084	13.15824378860239	2.85957753946573

C	8.55279843806992	14.21394509439653	3.68512398471767
H	9.11195545043212	13.75852323248153	4.50632056170572
H	9.26675632143601	14.74605067979248	3.04660555892825
H	7.84708385353102	14.94693638350711	4.08985086611629
C	8.84287987306054	12.09470017737560	2.43265025596998
H	8.33954334506983	11.27780091533347	1.90694623097847
H	9.57488726573431	12.55069000950868	1.75591685114981
H	9.38744838386158	11.69261007053014	3.29327296763976
C	2.33519631146607	7.86939480576096	-0.71124355260089
H	1.62928849181056	7.17136912402851	-0.25008017791206
H	1.79348752142508	8.48226853604233	-1.44463914973227
H	3.08664110993940	7.28850999014103	-1.26471408156297
C	7.21447881993776	13.80655897478925	1.63973061048241
H	6.49509535433925	14.57536217950682	1.94499028080906
H	7.99468283711716	14.27917483307042	1.03216949501190
H	6.70049286576810	13.05830507918561	1.02927883046866
F	3.22149973590448	17.48136246348358	6.11621862783242
F	2.46424509584583	15.57714954839320	5.44998485368405
F	3.02229925348836	17.07754070627034	4.00809087392650
H	6.14330375650153	13.78426171983261	6.99416701824347
H	6.10350941160603	13.33803508331406	3.91941202234407
H	7.30678350602180	4.59645511008071	5.78852194350005
H	7.88230820866697	7.09493254600974	5.83483014819239
H	8.53746272599591	10.03928446239169	4.53872663501257
C	8.39494208812411	10.31018232777606	5.58312452280692
C	6.32602317985302	4.85537194062954	6.21090013490239
H	5.81301625851239	6.28430125884825	4.69091411691194
H	9.18693436594439	10.88641982378940	6.06261944360648

C	6.88102718961583	7.31628821318127	6.23498406639080
H	6.43519617303912	8.75082023912884	4.68684747508339
H	5.60060847542237	4.10594947497170	5.87055777436580
C	5.90848390373363	6.24556984313724	5.78723610315823
C	7.39971532306216	9.74102199435690	6.30280431218097
C	6.46444419318811	8.71118489447591	5.78407861209394
H	6.40011688717129	4.78552910306090	7.30503770859461
H	6.96359359238495	7.29751827302679	7.33325482346255
H	7.35862991958405	9.93035662950564	7.38044916247412
H	4.91284495326036	6.47332812799016	6.19864869815440
H	5.45138361150589	8.91585235334741	6.15611348013300

Table S13. Calculated coordinates of **3-hexene**$^{+}$-HS

Co	6.78310461260120	11.83881137092636	5.67457044841525
N	4.86953962825589	11.78300601044771	5.65786894776574
N	6.65503364832161	12.91941572577965	7.46538714352435
N	5.78536090866022	12.30154421506701	8.38242489371540
C	4.03736895915562	12.00606953340107	6.70373740840731
C	2.72324147895254	12.07293836048482	6.23192611892484
H	1.84108413143374	12.25737611398245	6.82657756708943
C	4.57258753434556	12.00376508688244	8.04605978505948
C	3.71128382553362	11.56172950684891	9.15701603459034
C	2.76705945140267	10.55055980676515	8.99152309456048
H	2.65077170934126	10.07640741439879	8.02273795641229
C	3.84759458848456	12.13467609671381	10.41741885850347
H	4.57966854173432	12.92179081824864	10.56299712815728
C	3.06113838316551	11.71712937159526	11.47148967678830
H	3.17766033026382	12.18853498964056	12.44378445875330

C	1.99183972082048	10.13139819363780	10.05204631294404
H	1.27126273056493	9.33118234400310	9.90207829912662
C	2.11757729352243	10.70896440697194	11.31156899757592
C	7.84680699432299	13.44244806375497	8.22661298292608
C	8.75964421947609	14.12825473896883	7.23697199040150
H	9.15380754883443	13.42424745774710	6.49386815515003
H	9.61246904771171	14.55473535387717	7.77606471736148
H	8.24253086085063	14.94595232335199	6.72075666041732
C	8.56116289750529	12.28747499837903	8.89668598036952
H	7.89559206487877	11.77043014360887	9.59328094201410
H	9.41939439939982	12.68056686957519	9.45291468987407
H	8.93789645422966	11.56952706036150	8.15963654887089
C	1.25999383340362	10.26144746163644	12.44464697276062
H	1.32308220610049	9.17252025340024	12.57307476918923
H	0.20600503895584	10.50546729805107	12.24972796752357
H	1.55813845433548	10.74384934723874	13.38071108941314
C	7.36910297167936	14.44771296358824	9.26113157448278
H	6.85071261682659	15.28742379753201	8.77779178478756
H	8.23199088200201	14.84684406509062	9.80602808107493
H	6.69022158948890	13.97483638379237	9.97597370306878
N	6.58623257415700	12.68261297653370	3.76730345693699
N	6.00019265700429	11.65455688480310	3.01551999615871
C	4.11971563889336	11.66332633046936	4.53621932510712
C	2.77509717513743	11.85186853096893	4.86141897793059
H	1.94256816273109	11.82825715254198	4.17376818349822
C	4.79561093418731	11.28035099771033	3.31268352231139
C	4.14760841263828	10.35912609156110	2.37157546676609
C	3.20917222827732	9.42255786005164	2.80154835031191

H	2.94723081051530	9.36955548267860	3.85289522508497
C	4.47536342360507	10.38982364525191	1.01818620596364
H	5.19704027124812	11.11900066399877	0.66648593942630
C	3.88414768470802	9.51646137961279	0.13011047404610
H	4.14821774104461	9.56518650574759	-0.92299640037843
C	2.62727195059978	8.54825279373980	1.90805936364984
H	1.90706131277054	7.81767420876314	2.26783892564835
C	2.94838164485769	8.57929503068832	0.55474857507124
C	7.57350038200280	13.43265219768706	2.91497063587326
C	8.00425840873355	14.64967832631886	3.70261980420036
H	8.54749032003965	14.36394942355827	4.60508401055780
H	8.67196329706075	15.25937524681204	3.08436521466403
H	7.14114366190484	15.26982765515550	3.98205339293551
C	8.75866655263965	12.54559363879587	2.60368707719566
H	8.43060222206632	11.61226870658992	2.13482352595457
H	9.42437494002050	13.07007735988277	1.90914979378361
H	9.32646935825822	12.31638427019141	3.51216121883007
C	2.29868123721072	7.64409239711633	-0.40541798017335
H	2.35463632079478	6.61011508072434	-0.04021318373181
H	1.23346547889145	7.89120477830319	-0.52073030428594
H	2.77157648539936	7.69686596845604	-1.39105872077165
C	6.88287650056624	13.87743316167197	1.63579821471469
H	5.99165659724841	14.47843125784696	1.86277660571291
H	7.57374054376406	14.49578351419833	1.05207678735143
H	6.58405800796086	13.01734338646921	1.03082146538793
H	6.17271929471417	13.74987014502766	7.08111053296261
H	5.85487256296788	13.36756895497270	4.02341392905336
H	7.34148268480215	4.59484216282523	5.42625789417865

H	7.89330751744756	7.09378860415743	5.56812034453728
H	8.46951799005980	10.16334557023388	4.36632194040924
C	8.35321997948058	10.34952784198432	5.43306631721545
C	6.35739034410294	4.82816548712738	5.85538066823710
H	5.83490616721907	6.30928047551910	4.38919663112480
H	9.14915312341452	10.90273094058118	5.93356082036014
C	6.88990218107024	7.28893796680285	5.97614467244331
H	6.42600129132571	8.78177563964326	4.48689328282338
H	5.64003831157554	4.08608252402014	5.48410337201524
C	5.92705432943862	6.22915131335126	5.48351571873339
C	7.39367793961484	9.70635439306201	6.13879538277178
C	6.46028573671736	8.69800726655282	5.58117414543895
H	6.42880354689271	4.71729662783185	6.94613160133621
H	6.97156985969927	7.22677176727191	7.07270730319141
H	7.38510169290855	9.81918167192904	7.22882711098281
H	4.92847298278007	6.43237375854943	5.90073558813776
H	5.44758997026842	8.87877603790578	5.96825785831475

Table S14. Calculated coordinates of **3-hexene**$^{+}$-LS

Co	6.46466792765504	12.74700101845773	5.37422130448439
N	4.98487234998561	11.69346440359125	5.29312343494659
N	5.96182123900051	13.26682135094137	7.14463098778996
N	5.59337304021710	12.32832469771175	8.11630694753958
C	4.27400803977419	11.26889247507610	6.37070946911046
C	3.07852248765384	10.70274743925948	5.91584178471129
H	2.30123929407046	10.28864148412840	6.53974659732498
C	4.73961805255138	11.43464162887199	7.72845618738609
C	4.24032336778104	10.53289536347676	8.77565365930423

C	4.01648780306492	9.18298368967842	8.52223907837954
H	4.19829759633254	8.78417889394972	7.52962472246436
C	4.02234129510202	11.01015419093692	10.06582556469346
H	4.19250653846318	12.06090956065220	10.27607762674358
C	3.58664551394730	10.16444816874815	11.06288932558826
H	3.41240124734925	10.55953871484218	12.06049487764507
C	3.59097220526416	8.34045796171989	9.52936502056144
H	3.43814629259018	7.28640733412376	9.31322471258568
C	3.35946098233628	8.81355712402097	10.81546424761419
C	7.08800264554559	14.16423400249002	7.54790502182184
C	7.58469389466210	14.66045610604306	6.19492592508616
H	7.98472334715800	13.82201286238210	5.55225777598501
H	8.48078363492343	15.28322322517271	6.30192386633363
H	6.83021428837973	15.25237904456033	5.65810616380125
C	8.16261236608690	13.36723169845961	8.25360005950992
H	7.80732637232128	13.01975311423306	9.22688779623026
H	9.03592380183552	14.01236823258930	8.40155722081107
H	8.47074923789530	12.49566885144972	7.66410004504021
C	2.86389727800469	7.91058064195814	11.89158019129392
H	3.10382898829450	6.86478023589379	11.67127737544565
H	1.77057364746711	7.99262670785393	11.98314332202099
H	3.29642320186962	8.18078467081703	12.86238727966090
C	6.58765342178789	15.29907678573193	8.41287187207886
H	5.83322290609700	15.89588920863235	7.88236721349863
H	7.42039148917318	15.95795572250239	8.68626234637320
H	6.14486249023424	14.89768484461873	9.33190830103396
N	6.92085930676009	12.30606958902474	3.52999772717269
N	5.91843374106198	12.22109784686776	2.52731339351224

C	4.28203373477177	11.41829932899814	4.16923144269188
C	3.07036573068858	10.82042369998350	4.53889159150123
H	2.28686331757959	10.50783476157534	3.86725885727540
C	4.75769507177415	11.76372589284729	2.85263618247556
C	3.82145532304411	11.64123302483551	1.71273274591692
C	3.25005490165809	10.41922338046365	1.37526909936544
H	3.47932076032473	9.53358299947906	1.96008643658068
C	3.53552859210236	12.74936813779442	0.92844541761124
H	3.98537019186734	13.70633107053185	1.17380383449542
C	2.68420026466826	12.64190383965223	-0.15472817543697
H	2.46509079534740	13.52468500630480	-0.74954748236910
C	2.41225676313779	10.31787804284129	0.28356882517696
H	1.98673200241145	9.35075026110046	0.02734807993967
C	2.10540030638168	11.42717058124884	-0.49779718601290
C	7.90176342052734	11.14206812898455	3.42799260236362
C	8.88900016422403	11.34560009651583	4.55996458524968
H	8.39444221493012	11.27359567828994	5.54360917428834
H	9.64721425602000	10.55492348650076	4.53039720874964
H	9.40579098961418	12.31055248227844	4.47849874369741
C	7.18861743361177	9.81812909690141	3.58627932876123
H	6.47792744838544	9.64658777103612	2.77278854322749
H	7.94014788357282	9.02149413880073	3.55136577993922
H	6.66324233682823	9.75184954336492	4.54408958303884
C	1.18436963636129	11.30658202587048	-1.66347245790746
H	1.53441938198900	10.53005913536796	-2.35700083886128
H	0.17724421023091	11.01712656638205	-1.33200751839579
H	1.10962453058564	12.25324413527727	-2.20769074252805
C	8.60943847180054	11.21422595404063	2.08762144823615

H	9.14143463107954	12.16909058992708	1.97644063413013
H	9.34543489148497	10.40462182298363	2.02451806349354
H	7.89528770640084	11.10635567790184	1.26569359385332
H	5.13809650830777	13.86857017625768	6.96784395684419
H	7.48284479559068	13.10157457419574	3.19781019702469

Table S15. Calculated coordinates of **4-hexyl-α**-LS

Co	6.55929869005963	12.09858754581786	5.34922637366510
O	6.16576932932527	14.29638709822737	4.98905626017564
N	4.80145773385742	11.62156145994689	5.35590958295464
N	6.43356971446278	12.65011193692283	7.35780854852389
N	5.54592155084161	12.03684265818298	8.24820826270567
C	3.99495675024068	11.49938041445106	6.43857792309103
C	2.69037817176208	11.19058825274682	6.00252336835226
H	1.82930778865696	11.08659990623386	6.64379504103238
C	4.42415664260360	11.59052192482299	7.80660767868450
C	3.52907632027514	11.06596097926123	8.86165419212681
C	2.95759373561715	9.80056866716834	8.77358386499661
H	3.14652290231909	9.18637534021815	7.89852173176012
C	3.28831091217098	11.82236945609113	10.00040465841949
H	3.73235227912007	12.80955144540580	10.08083416752569
C	2.48905477969701	11.33203081279855	11.01577774150762
H	2.30420004883479	11.94677144328627	11.89291850941785
C	2.17088604461598	9.31200924567887	9.79624699297395
H	1.74592277047643	8.31448408515253	9.71333770677186
C	1.91630342309363	10.06979608379683	10.93491500700530
C	7.65035530776786	13.06576462255724	8.16721004191165
C	8.63141208029010	13.76189575764773	7.25193497967968

H	9.04750571873534	13.06802287105441	6.51645233056607
H	9.46571610149708	14.13465503453467	7.85630956311774
H	8.17994825517373	14.61359926305254	6.73384826750788
C	8.31205830767335	11.85498377043030	8.79062705387418
H	7.60673334899977	11.29601481428821	9.41228009409886
H	9.13462434076642	12.20233637148690	9.42672489220346
H	8.73151849475792	11.18951198753558	8.03047565853282
C	1.05411845907863	9.53647838268909	12.02857268437585
H	1.44962912926545	8.58401415382099	12.40763242389866
H	0.03600619881465	9.34343565430898	11.66222347846723
H	0.99267834761249	10.24371711086193	12.86198671239622
C	7.20035618519236	14.02955042880293	9.25572006816525
H	6.70840080820950	14.90757692637992	8.82084652506038
H	8.07975346906233	14.36528076838146	9.81760510871362
H	6.50833942588261	13.53659120451454	9.94431345051887
C	3.98840621749041	15.63552557916253	4.45312038382321
S	5.48678765396247	15.48050082551498	5.52505919072949
O	4.95616084961828	15.25624160258646	6.85300820558808
O	6.19963783922997	16.70624312587418	5.30604789531257
N	6.69086691008580	12.09513687713649	3.48255226156899
N	5.80712546104486	11.77576483723643	2.62909751618923
C	4.06652405711866	11.33270164463815	4.24798861345982
C	2.72918690974110	11.09551697069003	4.63975552812753
H	1.90546520163915	10.90139898920737	3.97233817879626
C	4.59953862585519	11.32148903328008	2.94407983918043
C	3.81725780429032	10.85895946839679	1.79384339188532
C	3.00751282371122	9.72512959715861	1.85636095852901
H	2.95335750946107	9.15514325705121	2.77779420761218

C	3.90143276420334	11.53367949374178	0.57740562122189
H	4.53658534130186	12.41018239470948	0.50555824356899
C	3.18224878135612	11.10764303448847	-0.52003298375754
H	3.25459226797097	11.66307119986866	-1.45161411527229
C	2.29912997186045	9.30050050104513	0.75198770152692
H	1.68598362430993	8.40541331120528	0.82534773556473
C	2.36333011456037	9.98630443030573	-0.45664911422063
C	7.88048832430331	12.72020147721591	2.79717851765625
C	8.90615315660416	13.12743120768590	3.82861224110884
H	9.23260885215242	12.26977480968579	4.42938280143126
H	9.79284415349174	13.50699839361127	3.31010433011574
H	8.53117483884554	13.92572267609146	4.47239227648939
C	8.50077634991899	11.70981134281639	1.84045443219107
H	7.73797038165232	11.28969071905392	1.17816550682269
H	9.25173469583595	12.22913551421135	1.23333989598336
H	9.00339528560676	10.90001365775429	2.37948313375271
C	1.57835779348175	9.52898617039708	-1.63830886289716
H	1.77875858024628	8.47080948732876	-1.85469315268623
H	0.49982416587489	9.62320859788242	-1.44661074350990
H	1.82257767650803	10.12032295503754	-2.52660995050105
C	7.41606083424179	13.93947440780218	2.01342952197471
H	6.92582515087111	14.65971498980119	2.67293708318857
H	8.29077042370476	14.41533773152159	1.55410678111907
H	6.72149945700777	13.64060068485175	1.22242117289550
F	3.27041513973557	16.69086240825694	4.81978222861452
F	3.21955030476014	14.55510149122320	4.54283553964574
F	4.33908999664279	15.79019551880686	3.17836244431458
H	5.97445347214426	13.54688552128036	7.10331522443815

H	8.39851424566864	5.89713742716953	8.22650189714598
H	7.90081523028768	7.67511660722487	6.45718657712440
C	7.16023655263663	9.39656812478570	4.34026101042135
C	8.26549544369844	6.67772080128429	8.98870267211925
H	6.31260274033371	7.29098118196741	8.33571668624700
C	7.76057689986818	8.43196219960920	7.24193006922727
H	5.81713238107930	9.04403649804789	6.56059787442294
H	7.90157544140845	6.19803006418905	9.90613850892449
C	7.30243839297347	7.74125369848458	8.51034029376992
C	7.25025095769267	10.26892133783136	5.56150013912049
C	6.78871663451355	9.51169967358557	6.78149187657949
H	9.25234991190576	7.11242686898739	9.20108197930631
H	8.74797674011100	8.88747586804466	7.42086399673973
H	7.16652382409407	8.49555191302806	9.30051646204839
H	6.62766606864000	10.17531753573950	7.62863067391506
H	8.28406576612699	10.64773908529443	5.70944123520380
H	6.11219347658089	9.21449077699702	4.07063767886298
H	7.60553957343032	8.41525621440755	4.56018792364854
H	7.67364638969752	9.80557030914838	3.47244181881120

Table S16. Calculated coordinates of **4-hexyl-α**-HS

Co	6.58205071807362	12.15847837707769	5.36781530935087
O	6.27206441695614	14.26198587778411	5.05817161213765
N	4.74919101328381	11.59653847094912	5.39295497030506
N	6.43086895624428	12.64902645457538	7.43954556088722
N	5.51850278413696	12.00025137070865	8.27773229403571
C	3.91810827427299	11.53300049572948	6.46468067146929
C	2.61418831713751	11.25339016766414	6.02363418176496

H	1.73748623981325	11.17405089785215	6.64757062932223
C	4.37878838660231	11.59571382485283	7.82986695473817
C	3.48335995698210	11.07454451526660	8.88856311566177
C	2.85641187992064	9.83731903175988	8.77790239229595
H	3.00454971457991	9.23905919739412	7.88437203884693
C	3.29224005413882	11.80880087756515	10.05152637326451
H	3.78038217635861	12.77313128324720	10.15111265143055
C	2.48758430594583	11.32664326600791	11.06635939819573
H	2.34181819218667	11.92531455764481	11.96183425479992
C	2.06533106044151	9.35522488123869	9.80038200794207
H	1.59739903683379	8.37888163540413	9.69865220167902
C	1.85966312728783	10.09256730615934	10.96203913034056
C	7.62448559918142	13.04648172774694	8.28243486314863
C	8.62571916330737	13.74881457035133	7.39001483672176
H	9.00812886279999	13.08068078077742	6.61068238956778
H	9.47809568260280	14.06665374199272	8.00106240686110
H	8.19883423860854	14.63801597191467	6.91531389201716
C	8.25751097459436	11.82243722181560	8.91206030551282
H	7.51753733028933	11.24965930805111	9.47881032310639
H	9.04248148986923	12.15505541156570	9.60115081761578
H	8.71969983330720	11.17118483940790	8.16550767021641
C	0.99017470694024	9.56867010874197	12.05454923671105
H	1.34297128756286	8.58597868550422	12.39672157650650
H	-0.04259457491930	9.43887743410137	11.70146941209160
H	0.97880022169456	10.25144459581842	12.91026172837667
C	7.15709869922578	13.99773646938542	9.37496659699165
H	6.68057656938347	14.88539466235613	8.94298729338050
H	8.02271047752013	14.31881654327923	9.96597092039477

H	6.44500331048346	13.49599230307119	10.03716168753329
C	4.11373256021278	15.63517705532829	4.49916758138234
S	5.60753603908965	15.46802236286306	5.57689277382202
O	5.06558088900647	15.26843653700567	6.90239370069242
O	6.35056161838461	16.67024204257257	5.34067771526572
N	6.70127446509014	12.13010281019045	3.42622048051895
N	5.78078099751584	11.74492505137357	2.63758281755299
C	4.02214146067949	11.30501735815832	4.28379485462605
C	2.67372627693280	11.11594166272550	4.65646791840747
H	1.84954934440840	10.92957258713294	3.98601940026858
C	4.58513855112529	11.27311153827055	2.98417168550745
C	3.81277685074202	10.75971589523964	1.84680533437567
C	3.01329785785440	9.62136325602266	1.95299179979390
H	2.96122740440011	9.09060594839366	2.89781353029930
C	3.89135705590232	11.38526796678601	0.60358361379531
H	4.51427615254754	12.26712717454740	0.49926291199210
C	3.18018449598707	10.90678505350658	-0.47712119759809
H	3.24754767763630	11.42513676378496	-1.43024635526444
C	2.31268511669802	9.14372535071012	0.86535376131131
H	1.70800557994068	8.24641316476497	0.97406394712913
C	2.37417358583446	9.77911427734064	-0.37053045425630
C	7.81329231668846	12.79730945557790	2.65577218129098
C	8.93865692311071	13.15450094153471	3.60274995360354
H	9.34051405688354	12.27197037944453	4.11218975487540
H	9.75413760141672	13.60269987428726	3.02493686813035
H	8.61442839433654	13.88955842452824	4.34513851176276
C	8.32206665351717	11.88347147017230	1.54756338499768
H	7.49676527007363	11.57761362183470	0.89850399900627

H	9.05253968318621	12.44231354328032	0.95069395069210
H	8.81752070567431	10.99172861492465	1.94091441392947
C	1.60044281641861	9.26324044678970	-1.53541570046586
H	1.83600084823485	8.20675004802062	-1.72352933978638
H	0.52004259386657	9.32596568792172	-1.34187747640183
H	1.82204647426706	9.83697036256704	-2.44103291701904
C	7.25758974488060	14.06284575299371	2.01211299853928
H	6.89198010423641	14.75878097411364	2.76864157303092
H	8.05760225912569	14.54736400329781	1.43941469316991
H	6.44111982721664	13.80809487365789	1.32853126675246
F	3.41046228608947	16.69762969162324	4.87193766854215
F	3.33585603119043	14.56290644942622	4.59019635563530
F	4.46463049000746	15.78950285537987	3.22630971511850
H	5.97298919421306	13.54814197193254	7.19348072644692
H	8.12456931157238	5.82684274535008	8.08792547075260
H	8.04531011745267	7.73206263662823	6.39944197842092
C	7.96940710747861	9.67882700549069	4.35168554299076
C	7.88697964017768	6.56625870164174	8.86548354675959
H	6.10406476908336	7.30062006331414	7.91892658913080
C	7.76043947122534	8.44615506506649	7.18491496357608
H	6.02067301607343	9.10204608343868	6.10706342001072
H	7.35174701453572	6.05032898669628	9.67276687452582
C	7.05843812082366	7.69787286109845	8.29897589601600
C	7.62242570509088	10.42802606483050	5.60365469500294
C	6.89343134413469	9.55879543392584	6.59771250488384
H	8.83554771482340	6.94299669145735	9.27315460865929
H	8.69456641999710	8.87881485097282	7.57673592815645
H	6.80491293899444	8.40599667971663	9.10251544803197

H	6.51165776407568	10.13896571702567	7.43974205982081
H	8.51723771806186	10.89179595469074	6.05148199089266
H	7.07339274098076	9.36763792817124	3.79961728714495
H	8.51516874346670	8.75996973329110	4.62110745753142
H	8.61453960368045	10.24301663240340	3.68062363099700

Table S17. Calculated coordinates of **4-hexyl-β**-LS

Co	6.68954615599290	12.60476493538929	5.53860442376682
O	5.94741300478923	14.62229609630896	5.48363776739623
N	5.04282698836044	11.84152574562391	5.48434864781049
N	6.76958734349470	12.61217308463774	7.38781486289134
N	5.89730916181605	12.24052109014419	8.23537135469942
C	4.26191730420451	11.59895002668522	6.56932744808382
C	2.99560398663626	11.15887019248385	6.12119323489007
H	2.14704644549933	10.94813367963240	6.75176961159304
C	4.72301617392755	11.72228861575204	7.89471835974923
C	3.90367427986824	11.29250563737823	9.03206664929870
C	3.22571515104959	10.07433337397651	9.03984232044204
H	3.30811073904862	9.40836330725067	8.18654027227204
C	3.81924738361395	12.09730917347364	10.16530736295725
H	4.34515593015740	13.04635280860298	10.17642239731181
C	3.06153786019513	11.70972473057295	11.25159157911362
H	2.99683200880745	12.36540024213422	12.11609609519109
C	2.47961553294977	9.68958182917130	10.13426110909220
H	1.97089530318079	8.72862688957695	10.12100833519401
C	2.37487253650034	10.50157306936805	11.25913472670623
C	7.95769671506311	13.23370315290214	8.06121966406239
C	8.82965222348941	13.90204093920511	7.02067566612128

H	9.22387763823428	13.18218151050151	6.29444910877149
H	9.69671372251930	14.34524725085836	7.52108106679498
H	8.29636684562932	14.70588840796602	6.50485297609994
C	8.73713893890188	12.13297886077651	8.76574231356494
H	8.08639808422825	11.60084616379046	9.46748206532686
H	9.56410698238118	12.58864189029112	9.32380548580824
H	9.15682149517553	11.41863039984401	8.04916514048574
C	1.55325035526517	10.08190182947149	12.43001948669816
H	1.88451683964019	9.10541499422068	12.80955273189059
H	0.49652499770386	9.97863449660298	12.14528150729549
H	1.62201541529370	10.81308809853860	13.24174530141661
C	7.49211704016850	14.27348250549711	9.06865537421469
H	6.86985353522044	15.03308228071781	8.58552061540148
H	8.37531293908474	14.76362299540741	9.49529905301255
H	6.92041074845220	13.80643459151530	9.87482737771371
C	3.91356071052750	16.09896793130223	4.90658809470685
S	5.15248364773002	15.63967062241256	6.19788348826286
O	4.36360317763663	15.12099406780351	7.27949381920648
O	5.88929838207513	16.84965237874905	6.43677066931205
N	6.60364076033237	12.92819339822612	3.49753391843486
N	6.01825893866480	11.96785752971254	2.67070045150404
C	4.35832144403849	11.46859285922830	4.37655322968342
C	3.06417803797570	11.06403417739091	4.75791639675515
H	2.28298184104993	10.75873178398292	4.07919932726252
C	4.94827535859632	11.37517145959004	3.07012122022568
C	4.33590546829291	10.47207830501583	2.07302464782758
C	3.88300526698218	9.20127758555053	2.41337423472375
H	3.95952210910547	8.86091436899302	3.44092986398723

C	4.25423916335957	10.86627811150929	0.74247539509766
H	4.61612377362234	11.85018922081729	0.46240023073703
C	3.72439129561986	10.02231967883872	-0.21299318115391
H	3.66316190053399	10.35627209440934	-1.24569569241929
C	3.36321058193769	8.35877881160249	1.45119234317214
H	3.02993883716039	7.36429079102046	1.73761697208866
C	3.26535088621642	8.75403360493876	0.12177572753608
C	7.72987729108197	13.56616915953158	2.70566100447678
C	8.44308157722359	14.59044285364956	3.56018275132804
H	9.00956548533759	14.11082405977335	4.36185347590978
H	9.16181345950890	15.12337150515829	2.92798487427728
H	7.75253378564471	15.32019971445746	3.99242853897242
C	8.71189382159144	12.50234930684913	2.25805660052897
H	8.21646363463806	11.73933572695126	1.65157534479015
H	9.49038509761631	12.98310572758463	1.65429927508560
H	9.19539101548368	12.02289138312093	3.11547294005436
C	2.67249932687241	7.85027584340903	-0.90533082954366
H	2.96278896685669	6.80777426572105	-0.72570411999234
H	1.57386554358287	7.89683562720133	-0.87079442175857
H	2.98887183039746	8.13820095931719	-1.91384963641133
C	7.11030528228567	14.26137671746122	1.50251402108512
H	6.42437108775305	15.05533477289496	1.82806652121270
H	7.90314598866815	14.71947420126197	0.90019921596094
H	6.56172737060847	13.54752506001080	0.88060823805546
F	3.09511825891688	17.03688133217799	5.36619206611147
F	3.18326971152591	15.04302773883637	4.55219667266939
F	4.51565969053155	16.56637543582854	3.81318168519723
H	5.91793800296045	13.68440733876109	3.65468559416111

H	6.10050587014745	4.78040902088739	7.08103475028205
H	7.28021932235465	7.00442509878579	6.59739339243819
H	7.47771771964361	10.66052792677081	4.27444402621868
C	7.63523737474403	10.94248138846510	5.32132283614934
C	5.16640375608790	5.34848270244357	7.19280304672549
H	5.27522147162523	6.30763042352892	5.27458036035998
H	8.64756127932019	11.37109125263771	5.42849127248988
C	6.32030001267734	7.54010213633983	6.68375062521377
H	6.49978414504137	8.48846371404959	4.76736163440215
H	4.33298174978039	4.69034183521021	6.91603196270358
C	5.19081318387929	6.59408263713690	6.33506214313014
C	7.46029000907320	9.74562116207605	6.21751903147786
C	6.36095910947089	8.78247638338909	5.82018013244764
H	5.05643651043566	5.60882759168276	8.25491374914355
H	6.22160453537546	7.83949476972978	7.74028509100081
H	7.31965776205675	10.04350791347595	7.26359386228175
H	4.23365241759147	7.12949880978626	6.43932704130261
H	5.38907204875719	9.28674124175411	5.88033794499789
H	8.41626610485087	9.18867558452835	6.19394663497406

Table S18. Calculated coordinates of **4-hexyl-β**-HS

Co	6.65480360836571	12.60177736503218	5.57307917946447
O	6.01391026423647	14.57658458733850	5.49360289752703
N	4.94480664594634	11.75106030081088	5.52286265736355
N	6.74677956954410	12.64896838338359	7.48160187739264
N	5.82926028230693	12.25697765405003	8.27748542840113
C	4.16604713857257	11.55268581417186	6.61401526549463
C	2.88308980956524	11.14485725226652	6.18675193657207

H	2.02750228316438	10.97091024746455	6.82018471155078
C	4.65209563073557	11.73852686121198	7.93106425430383
C	3.82206723896651	11.37163317955917	9.08558644083899
C	3.11281635112614	10.17253121355582	9.13758883036520
H	3.17784633646577	9.47496448350483	8.30874190717641
C	3.75270367676624	12.21998709722260	10.18789735948589
H	4.30301592394374	13.15450018797137	10.16707670844895
C	2.98296360980392	11.89254803267419	11.28515029111235
H	2.93243510328858	12.58220248273567	12.12393375879769
C	2.35480968857566	9.84677207124051	10.24318638398704
H	1.82339581985578	8.89826774841850	10.26389046750099
C	2.26761820891187	10.70203748710872	11.33667153832882
C	7.91246623345909	13.21757994150730	8.24039576818977
C	8.81779718871253	13.98108332445306	7.29501692581650
H	9.23942681792111	13.33751816131212	6.51717783262233
H	9.65839972853276	14.38265852497002	7.87084731164385
H	8.29236695703290	14.82048432072512	6.82936318230823
C	8.67260620396945	12.07190405728000	8.89519672365873
H	7.99304322119150	11.47948446421964	9.51741160232036
H	9.46228410648173	12.48782982835067	9.53262070919564
H	9.14040303469253	11.41879466012411	8.15140184982751
C	1.43527388539940	10.34633429979552	12.52113428165449
H	1.73745409067454	9.37341490156846	12.93247913228483
H	0.37534592416948	10.26617267513321	12.24074694299822
H	1.52725549606005	11.10175764542704	13.30805541221820
C	7.40603115803914	14.17298656927851	9.31294820680523
H	6.78711469243258	14.96007226814343	8.86983277014899
H	8.27235474233977	14.64179129425502	9.79431415031981

H	6.82582352840926	13.63978363211871	10.06987780050791
C	4.38155004887198	16.44013516467492	4.80720527788339
S	5.15970286145471	15.54170099254527	6.22295524490822
O	4.05934029729975	14.93743675719184	6.91651486824158
O	5.91676018277835	16.54747507380463	6.91255732987958
N	6.53827558422567	12.83165343333701	3.48702320780915
N	5.93888034862444	11.83097600467822	2.72261659307450
C	4.22892975255860	11.40340923745528	4.42331105197653
C	2.93091690621895	11.04199911309685	4.81375247597356
H	2.13018752408706	10.74259233686741	4.15490714822938
C	4.85127907785826	11.26870580802224	3.12764443736901
C	4.24609432428580	10.33907235430689	2.14962193548337
C	3.75461929628771	9.09349506697055	2.52756000922169
H	3.80271027964145	8.78876160985275	3.56814886973630
C	4.20093305578818	10.68268098079149	0.80293107695060
H	4.59191308475529	11.64613917756395	0.49288614552979
C	3.67034863350812	9.81519055728384	-0.13055370681216
H	3.63823918315674	10.11059144401957	-1.17628856963484
C	3.23486965572024	8.22623686502099	1.58744489851673
H	2.87217427930744	7.25176376635712	1.90516563376932
C	3.17348816315007	8.57180385720644	0.24231618925060
C	7.63094221542785	13.46931248978296	2.65738200881492
C	8.30959483674322	14.54678061901956	3.47585588996035
H	8.75191451137333	14.13516364644780	4.38877066595444
H	9.12120340615039	14.97346331999120	2.87571581565018
H	7.62005249395149	15.34940312634300	3.75120802808613
C	8.64965432542674	12.42669026052935	2.24041519627426
H	8.16333775517250	11.57023582066943	1.76405111074457

H	9.33612527313689	12.88529528296760	1.51953019287410
H	9.24059649596929	12.08031783696396	3.09383493837926
C	2.57821864815872	7.64450015915253	-0.76217294063051
H	2.81500414344731	6.60069690683248	-0.52268620251417
H	1.48222156049766	7.74025845235281	-0.77166054694259
H	2.94245739579262	7.86920329691190	-1.77074803372669
C	6.98449756079077	14.09458063668997	1.43031847047641
H	6.23882879545543	14.84409559237435	1.72768788425878
H	7.75205472717348	14.59539965694480	0.82920371321598
H	6.49761564294971	13.33044493456542	0.81652759729583
F	3.51715789962663	17.33840406121896	5.25959337913890
F	3.73061978119116	15.59473634810553	4.00891192016420
F	5.29988773959963	17.06949078137276	4.07812491482675
H	5.84293916699517	13.57714170626770	3.64916614703291
H	6.23893446087806	4.82080494875799	6.77423137097984
H	7.44791911843009	7.08837492686060	6.57219694507422
H	7.88073226368250	10.84659016777596	4.35223997479885
C	7.90303567433133	11.06742536206539	5.42232385422598
C	5.29350887044636	5.35764416524279	6.61339518376319
H	5.89673770241626	6.31341066138314	4.78670842839150
H	8.85980211414036	11.53414668968900	5.68536396047062
C	6.47628803406529	7.58457711888991	6.41189678965487
H	7.06410333635688	8.56802876773868	4.59644426415699
H	4.59158874894834	4.67029617684328	6.12430091318432
C	5.51524088499334	6.59980908196678	5.77964617820935
C	7.62844876733314	9.82967210124624	6.23506481634452
C	6.67960789499856	8.84022927538854	5.59259863600907
H	4.88352695259190	5.61786396058998	7.59940202872389

H	6.09863300764560	7.86339708389480	7.40968510111048
H	7.27095314492402	10.07362284411711	7.24263118023251
H	4.55057037483315	7.10337548158954	5.60814153316904
H	5.70736466572994	9.31992526296938	5.43127206409275
H	8.60085386898616	9.32241236805496	6.38366301408405

Table S19. Calculated coordinates of **4-hexyl-β**$^{+}$-LS

Co	6.81735999456260	12.04233684574667	5.53850429424786
N	4.96717760962236	11.65576211191497	5.52332227753318
N	6.84572835554064	12.57136894240330	7.35990486558153
N	5.92794828679665	12.24080129618514	8.19281872178347
C	4.15439809813700	11.66053282407559	6.60517979892276
C	2.81402037056797	11.49770719333726	6.15930787213675
H	1.93077841980060	11.51639264492545	6.77894661390976
C	4.68753808911268	11.82011149040817	7.90226680916454
C	3.87449614352998	11.53892820820569	9.08279752400180
C	2.94858884152921	10.49229786928634	9.09781831000923
H	2.84718380347601	9.85303797557558	8.22734324750758
C	4.02537356563106	12.29678296740461	10.24514885035291
H	4.74789244936074	13.10501721969361	10.25778593017128
C	3.25868461753658	12.03809952111900	11.35970044702920
H	3.38140610867852	12.65440935109483	12.24615862391665
C	2.19644215470518	10.23221948114370	10.22112215884176
H	1.49696493017239	9.40017370862579	10.21420256085904
C	2.32771290632837	11.00361268831727	11.37308556527406
C	8.04768074460160	13.11107973384108	8.07715938120875
C	8.96949242213927	13.76055696666323	7.06982967967072
H	9.22759574823362	13.08734223093470	6.24535802814815

H	9.90230313867061	14.04073657187528	7.57084390131604
H	8.51769896351331	14.67075152225185	6.66290493524826
C	8.74895734732924	11.97046031661615	8.79837228759481
H	8.04510942061195	11.45402388430853	9.45995079111903
H	9.56606303596002	12.38048696560551	9.40332067274193
H	9.17353203838865	11.25136922100354	8.09045291772189
C	1.50195327674383	10.71876733325436	12.57781101783800
H	1.63177055711909	9.67578559352479	12.89790492346152
H	0.43478992730777	10.85745634009015	12.35296945521244
H	1.77129642082937	11.37786057297338	13.40861421930242
C	7.59536231148199	14.16619132415051	9.07676111840078
H	6.96829208704552	14.91894880954159	8.58335873089938
H	8.48328403090977	14.66477193786865	9.48209561697948
H	7.03609758557393	13.71836168284230	9.90152800317759
N	6.50139109106926	12.86860399491373	3.66374340955559
N	6.01823899960120	11.83522794109619	2.85228305532850
C	4.21320385447688	11.44898849620272	4.42112931774768
C	2.85962820105525	11.35391792694909	4.79260654570564
H	2.02450960628233	11.21215412340907	4.12294905339959
C	4.89583754966740	11.26525300944976	3.15410137094602
C	4.35344464974288	10.32466710230318	2.16715465022270
C	3.63250899877744	9.19818555902442	2.55834631316867
H	3.46387423982983	9.00365616356651	3.61279632656486
C	4.57916339641462	10.52601561222175	0.80779282840230
H	5.13844245613565	11.39883666509822	0.48861461246069
C	4.09519461285036	9.63324986531682	-0.12440598095046
H	4.27670379943205	9.81408328830646	-1.18057055729049
C	3.15833461267183	8.30638354902723	1.61960923745666

H	2.61133286153815	7.42658557591996	1.94857090426069
C	3.37367268571011	8.50766435888841	0.26010204535606
C	7.44261759635733	13.74903941267895	2.88851004875639
C	7.63469688928967	15.00319071379557	3.71458581800394
H	8.05861813812685	14.77580670815155	4.69544686240243
H	8.32548831745927	15.67384993274524	3.19271963840854
H	6.68270250336095	15.53460132809638	3.85121276782434
C	8.74917421776707	13.01576436369466	2.68159033066224
H	8.57680275809046	12.05829020784415	2.17804044041548
H	9.40902182400667	13.62427106683731	2.05347924729638
H	9.25611937808107	12.83420599308755	3.63608674121782
C	2.83349255735268	7.55423294142360	-0.74840151830054
H	2.96936634525915	6.51680829626654	-0.41821032659869
H	1.75405422674645	7.71542421856036	-0.88579699932064
H	3.32159565399567	7.68682174917900	-1.71939638562463
C	6.81138811705947	14.11771974019376	1.55578622347290
H	5.82092494627220	14.56791794048702	1.70458424194803
H	7.45093977473009	14.85288655472987	1.05472052055503
H	6.70927514437377	13.24214230075503	0.90952646616064
H	5.71279058720361	13.47155035350179	3.95237319798476
H	6.08606119376931	4.44842414323601	6.25848492977093
H	7.42735992028199	6.63193024710742	6.11969378442735
H	8.03000548218537	10.51670557858091	4.27189319573668
C	8.13417509321158	10.62447004759827	5.36385564626562
C	5.18286788756901	5.05896166335753	6.39544016181103
H	5.46585902563391	6.25032336112572	4.63133187129544
H	9.10714228223441	11.05262346718863	5.62784448697329
C	6.49829865764183	7.21215548502641	6.24324895848498

H	6.80454621293583	8.39943617379843	4.48209809818508
H	4.33106021071227	4.50125068125506	5.98678048854243
C	5.33443811654152	6.40083570821421	5.71458137105997
C	7.81132707980271	9.36688286692751	6.10662863322543
C	6.65535096629116	8.55447217946646	5.56249256335821
H	5.02166848868516	5.18400686546391	7.47526016917741
H	6.36699393773109	7.36810228948195	7.32682998786217
H	7.65973815298660	9.56865758281232	7.17790817091291
H	4.40688376594662	6.98275514477533	5.83865475565345
H	5.72179868789566	9.12229149834198	5.67144892394467
H	8.72594044560751	8.74173461571012	6.06039320442858

Table S20. Calculated coordinates of **4-hexyl-β⁺**- HS

Co	6.84254430949170	12.40896155864941	5.58198774950084
N	5.12607026117667	11.84643567334404	5.50455750812379
N	6.84085926762556	12.43495431471087	7.38728436982192
N	5.95742965524711	12.15240918468427	8.26047251808902
C	4.30367164359252	11.64918787843195	6.57890868798833
C	3.01612856880597	11.32781771691607	6.09941396073260
H	2.13838554735592	11.18557432950480	6.70929684500352
C	4.75243011828515	11.70620552979913	7.91446855537285
C	3.90908537841477	11.30180866576297	9.03754111645761
C	3.09896143647918	10.16639811890354	8.98506462806329
H	3.09846555650547	9.54411395393609	8.09594397811077
C	3.93613368782663	12.03555333315615	10.22314283836459
H	4.56998240221314	12.91370012918100	10.28694074772288
C	3.15512280076481	11.66786270348022	11.29780292777539
H	3.17890535491078	12.26809356116491	12.20335523438156

C	2.33298199075725	9.79870645174067	10.06951525541761
H	1.72309762246047	8.90074255398440	10.01035718646157
C	2.33610454557479	10.54509173759580	11.24498069405828
C	8.06935697887110	13.06116250071655	7.92223161618959
C	8.66476880723285	13.75132670176060	6.71261555252897
H	8.70949641504837	13.07795215964498	5.82418633716335
H	9.71502732109517	14.01899401671304	6.87579586587940
H	8.10655207406438	14.65948207323324	6.46781370350529
C	8.99313499253916	11.98620901657256	8.47236718556033
H	8.47290693717391	11.40972746797972	9.24512477859545
H	9.86955642386684	12.46840540838321	8.92148141012907
H	9.33735262451211	11.30606800132581	7.68737407286144
C	1.49402263472036	10.14262844834259	12.40491005620490
H	1.74087514011620	9.12135758535056	12.72656333585343
H	0.43019857979471	10.14688849393082	12.12869379025920
H	1.63643080885825	10.82046154004612	13.25205017927024
C	7.74570063504217	14.08180916041613	8.99606931027097
H	6.98431367631228	14.78797225208178	8.64375513955872
H	8.65942535973276	14.63932885580545	9.23446923917939
H	7.38470745506048	13.59122737553961	9.90406634297752
N	6.63052963093496	12.97380238801954	3.60921912619278
N	6.15519215468607	11.96951119605099	2.75386176445893
C	4.42656355598765	11.52823271727193	4.37919565052247
C	3.10199889649046	11.23694629327034	4.73315994470265
H	2.31072458211204	10.99549581876830	4.03992135217043
C	5.06930973593818	11.36883293661205	3.09701614303422
C	4.50983327231107	10.42609788393983	2.11501975184514
C	3.93585733600155	9.21680550232888	2.49926475686308

H	3.88555722625239	8.95145785595742	3.55013115153015
C	4.58532193155283	10.71865424094667	0.75669191601990
H	5.03391149673089	11.65476918350492	0.44205470278921
C	4.09563288599478	9.83501386709187	-0.18270404442019
H	4.15785366894792	10.08932117752993	-1.23751739069109
C	3.45675322199019	8.33463309456315	1.55326815042345
H	3.02628838917302	7.38994578302010	1.87565906950194
C	3.51941028316649	8.62744645895872	0.19482234377479
C	7.60069934962533	13.83115931101185	2.82040187571282
C	7.96258835385618	15.04812022845871	3.64898960383361
H	8.70376837809304	14.81820002667833	4.41468167239960
H	8.39924193184443	15.80381031556841	2.98764012352321
H	7.07640117693316	15.49338420669410	4.12280103583263
C	8.82852727699644	13.01385961312869	2.48630503508605
H	8.56242045186007	12.14500761255545	1.87622643601787
H	9.52824490181468	13.63988244533882	1.92109905959455
H	9.33598094031239	12.67154446514460	3.39544128426990
C	2.97054724946326	7.68214067999164	-0.81739707412037
H	3.23102955390689	6.64605913441852	-0.56761138896619
H	1.87280422403627	7.74669716962003	-0.84472392617313
H	3.34794109347818	7.91272795569920	-1.81903141175711
C	6.90192607329330	14.30183111539836	1.55356096023897
H	6.01548550598042	14.90276547022291	1.80057398557265
H	7.59297095123116	14.93179430042122	0.98287882258343
H	6.59752524259815	13.45802242016658	0.93002118894820
H	5.83590339009546	13.60435397005641	3.81738069228208
H	5.68502614666087	4.71461733470699	7.07068735805042
H	7.04805548738318	6.80301125529899	6.47529917575094

H	7.45862532361405	10.53746773730528	4.21999759277728
C	7.72969947023562	10.71289024641679	5.26871339812371
C	4.84074037351520	5.38098266167886	7.29511522156300
H	4.84134196341060	6.36776664729277	5.38757573990435
H	8.77304021745243	11.05446915253690	5.33766696212365
C	6.17211161350222	7.43898442206244	6.68304444741974
H	6.24320603135616	8.42076910684886	4.77498190985065
H	3.91223442153287	4.82727226065917	7.10790503957150
C	4.90955186995334	6.63588642009557	6.45387987576012
C	7.49261833636094	9.51264954265421	6.13081973075747
C	6.25388249584860	8.69461949298098	5.84125972957988
H	4.88103173204010	5.62642278887763	8.36554849826259
H	6.23029110600377	7.71685774284950	7.74847234970030
H	7.52229116145012	9.77931400850345	7.19546808483102
H	4.03792321193220	7.27322713702107	6.67334367490655
H	5.35780153088353	9.29999420820056	6.02234685654692
H	8.37263618164539	8.85841254281132	5.97016927348902

Table S21. Compared single point energies of intermediates along catalytic cycle for 1-hexene hydrogenation without accounting for entropic contributions.

	**2**	H_2	1-hexene	**3**	**3-hexene**	**4-hexyl-α**	**4-hexyl-β**	hexanes
HS- S = 3/2 (Eh)	-3702.05	N/A	N/A	-3703.25	-3938.56	-3938.48	-3938.52	N/A
LS- S = 1/2 (Eh)	-3702.06	N/A	N/A	-3703.24	-3938.54	-3938.53	-3938.54	N/A
S = 0 (Eh)	N/A	-1.15	-235.31	N/A	N/A	N/A	N/A	-236.52
Δ (HS-LS) (kcal/mol)	8.26	N/A	N/A	-0.25	-9.47	29.87	8.40	N/A

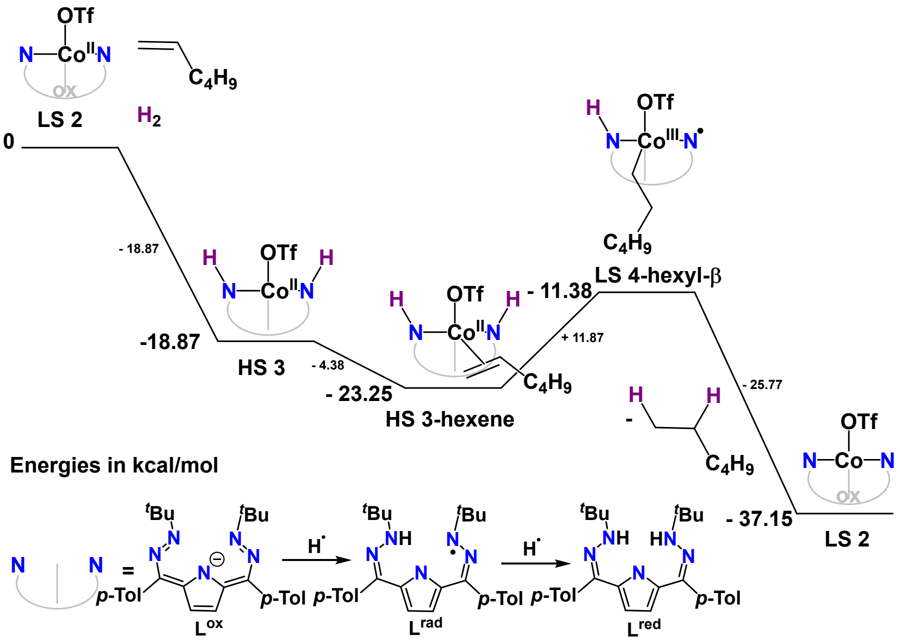

Figure S55. Simplified catalytic steps towards 1-hexene hydrogenation with the beta-carbon being the first to be hydrogenated (from single point energies, without accounting for entropic contributions).

Table S22. Compared Gibbs free energies of intermediates along catalytic cycle for 1-hexene hydrogenation with accounting for entropic contributions.

	**2**	H_2	1-hexene	**3**	**3-hexene**	**4-hexyl-β**	**TS 3-hexene -> 4-hexyl-β**	**TS 4-hexyl-β -> 2 + hexanes**	hexanes
HS- *S* = 3/2 (Eh)	N/A	N/A	N/A	-3702.67	-3937.85	N/A	-3937.803	-3937.810	N/A
LS- *S* = 1/2 (Eh)	-3701.52	N/A	N/A	N/A	N/A	-3937.83			N/A
S = 0 (Eh)	N/A	-1.16	-235.18	N/A	N/A	N/A	N/A	N/A	-236.37

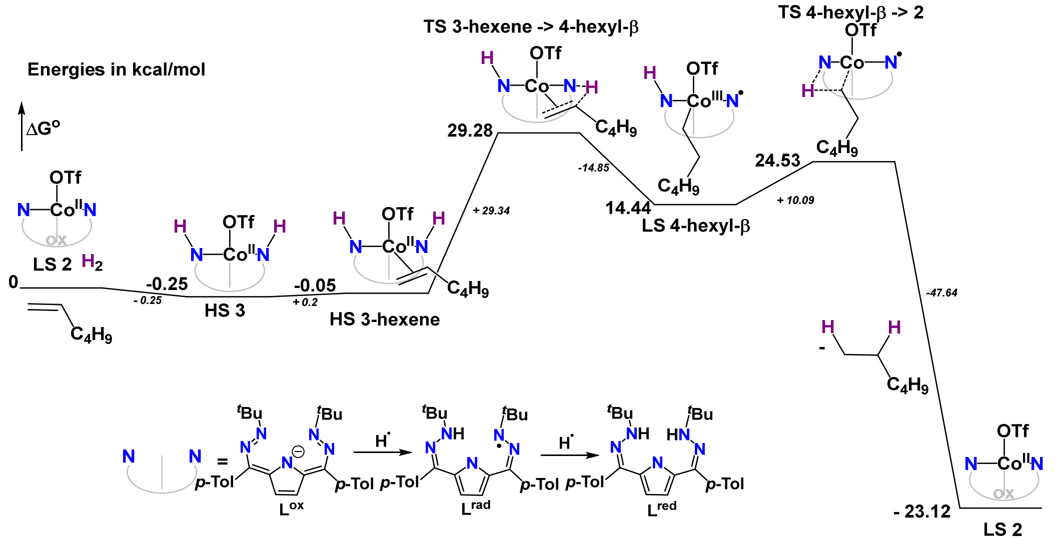

Figure S56. Simplified catalytic steps towards 1-hexene hydrogenation with the beta-carbon being the first to be hydrogenated (accounting for entropic contributions).

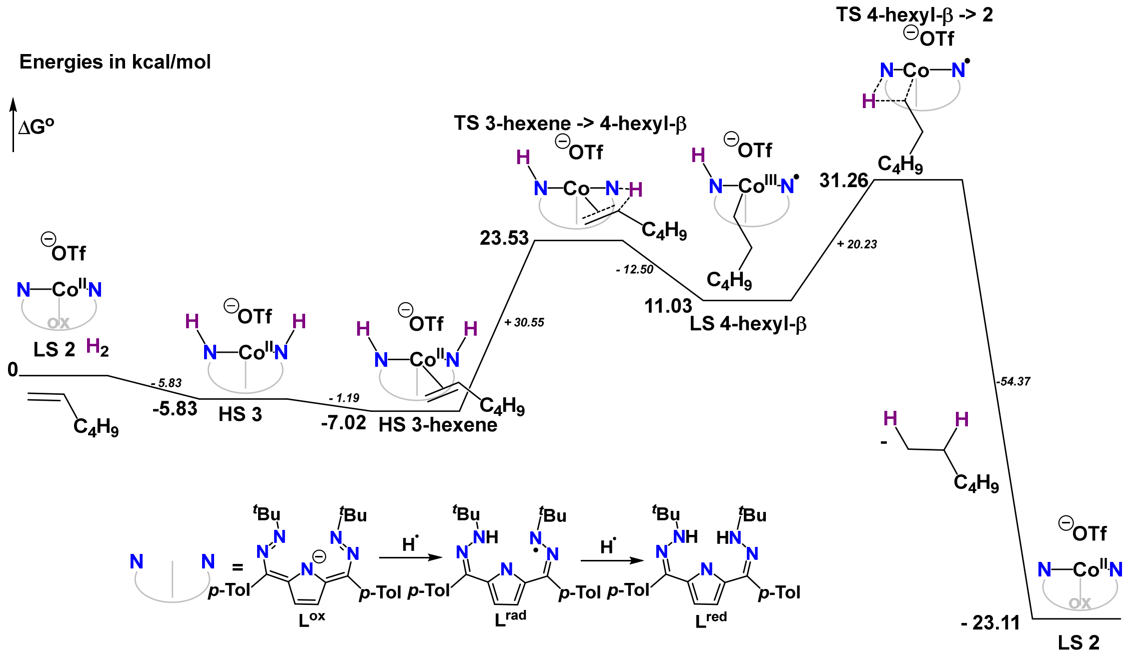

Figure S57. Simplified catalytic steps towards 1-hexene hydrogenation with the beta-carbon being the first to be hydrogenated without triflate (accounting for entropic contributions).

Table S23. Compared Gibbs free energies of intermediates along catalytic cycle for 1-hexene hydrogenation without triflate bound with accounting for entropic contributions.

	**2**	**3**	**3-hexene**	**4-hexyl-β**	**TS 3-hexene -> 4-hexyl-β**	**TS 4-hexyl-β -> 2 + hexanes**
HS- $S = 3/2$ (Eh)	N/A	-2741.80	-2976.98	N/A	-2976.93	-2976.92
LS- $S = 1/2$(Eh)	-2740.64	N/A	N/A	-2976.95		

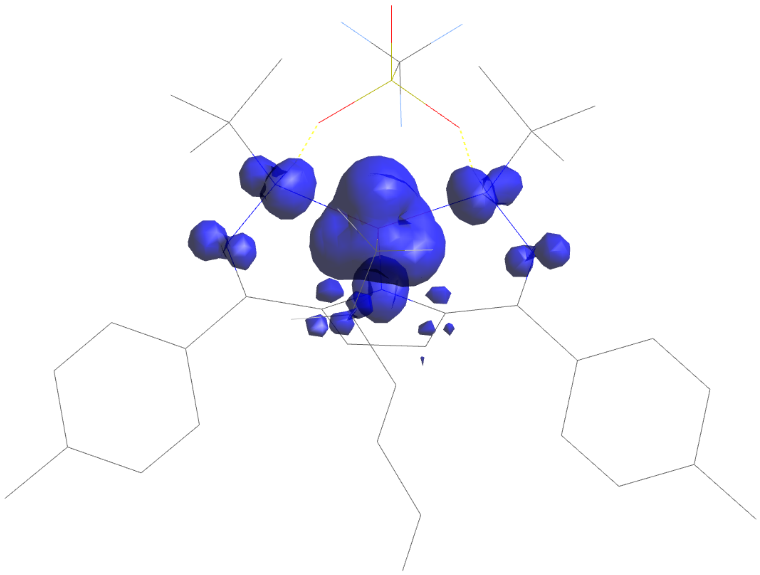

Figure S58. Spin density plot of **3-hexene** at an iso value of 0.005.

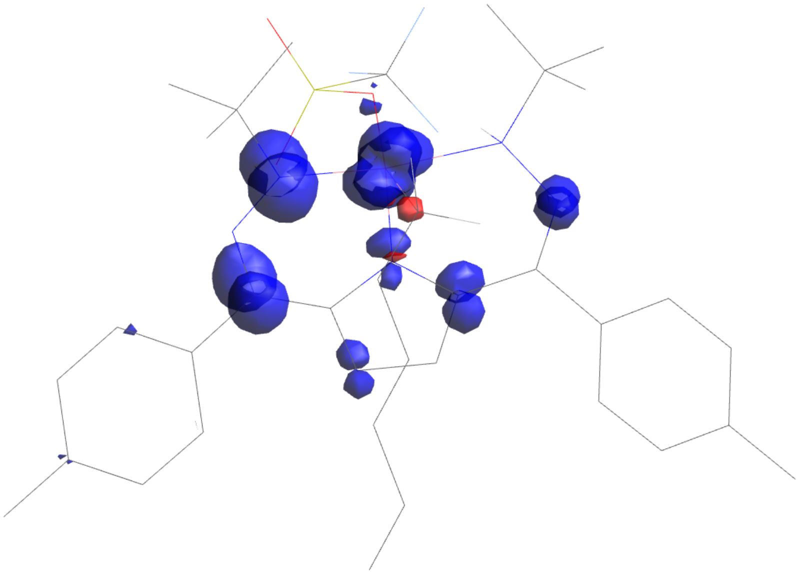

Figure S59. Spin density plot of **4-hexyl-β** at an iso value of 0.005.

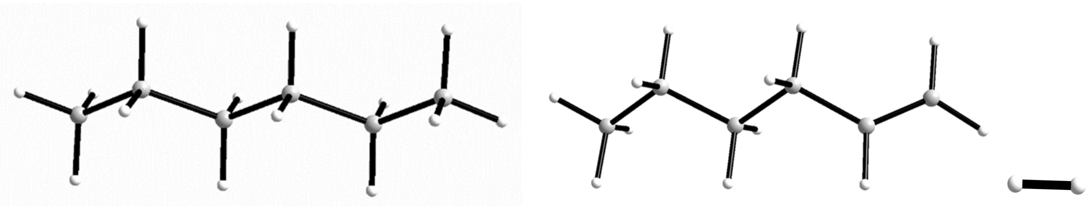

Figure S60. Calculated Structures of Hexanes, 1-hexene and H_2.

Table S24. Calculated coordinates of hexanes

C	2.99849846513162	0.01424611645290	1.71939328032738
C	2.15981043304487	-0.07731080989336	0.46386216053545
C	0.67328980992607	0.04308763675020	0.72447093058640
C	-0.17700807705380	-0.04039549983932	-0.52458641895658
C	-1.66369498316686	0.07693906382146	-0.26352918915267
C	-2.50220983026633	-0.00903152268144	-1.51955646907949
H	0.47465380461547	1.00117963036376	1.23321950107690
H	0.36607401990107	-0.75169878409947	1.42439570655254
H	0.02329894878552	-0.99742619574417	-1.03467784447940
H	0.12865419565579	0.75589698613086	-1.22347890580147
H	-1.86380064508342	1.03369829472505	0.24466380409731
H	-1.96863592829424	-0.71789741029821	0.43561364460931
H	-2.34494522835264	-0.96999121940441	-2.02950400576857
H	-2.23417385034205	0.79182667705056	-2.22297235748334
H	-3.57167985085146	0.08295446705449	-1.29112252592892
H	4.06760771361485	-0.08396438244650	1.49186404567254
H	2.72693423823331	-0.78033523427009	2.42856671930624
H	2.84524793403625	0.97955626799810	2.22226502247108
H	2.35881079574808	-1.03688644997195	-0.03940259799095
H	2.46560803471792	0.71360236830152	-0.23935750059381

Table S25. Calculated Coordinates of 1-hexene

C	2.94796208014444	0.37040881184751	1.36683988544666
C	2.07561918907461	-0.39869357703492	0.73395680004431
C	0.60046866713673	-0.34179051852796	0.89326763339751
C	-0.13168448037554	-0.04132278358332	-0.40452390782771
C	-1.63851177787894	-0.04657960050051	-0.26104841059725
C	-2.36152547992025	0.26080447132618	-1.55335963000052
H	2.62229956811711	1.12377508676529	2.08512628166378
H	2.44715388757168	-1.13915161322345	0.01893369695696
H	0.34107580549604	0.41345689893004	1.64971664776696
H	0.24451667155679	-1.31697076072404	1.26898402958051
H	0.16202691759900	-0.78396797091388	-1.16406909745765
H	0.19545251508905	0.94065778740421	-0.78130212229182
H	-1.92798646382255	0.68987430490683	0.50531460105417
H	-1.95833860368375	-1.03167070224608	0.11445619411045
H	-2.11041069518123	-0.47890748011855	-2.32644190969393
H	-2.07766500780093	1.25254855881128	-1.93242374973484
H	4.01920361845586	0.27839361268824	1.19855804720703
H	-3.44966741157811	0.24913747419314	-1.41197298962461

Table S26. Calculated Coordinates of H_2

H	-2.13766183877736	2.55159400052843	0.00000000000000
H	-1.36957816122264	2.52889599947157	0.00000000000000

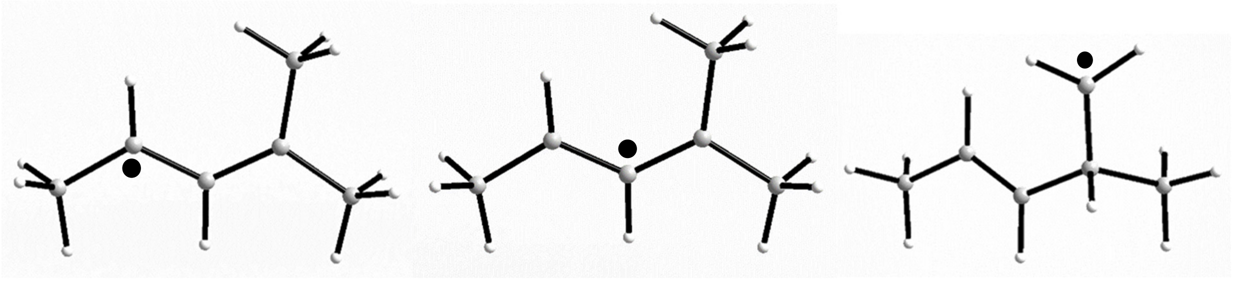

Figure S61. Energetically Compared 2-methyl-pent-1,3-ene Radical Isomer Structures. From left to right: tertiary radical structure, secondary radical structure and primary radical structure.

Table S27. Compared Gibbs free energies of 2-methyl-pent-1,3-ene radical isomers

S = 1/2 (Eh)	Tertiary Radical	Secondary Radical	Primary Radical
	-234.68343	-234.68344	-234.65
Energy Comparison	Second Highest	Lowest Energy	Highest Energy

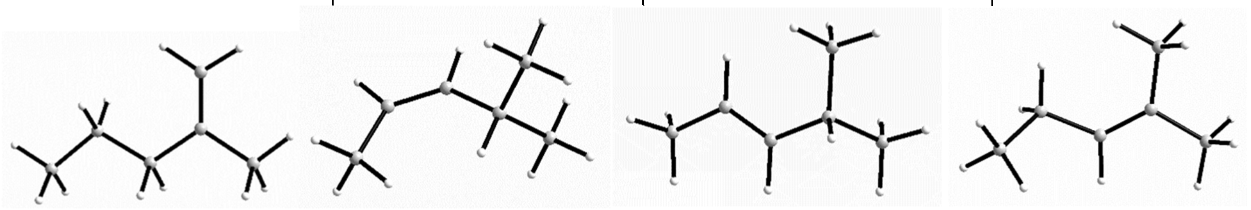

Figure S62. Energetically compared 2-methyl-pent-1,3-ene non-radical isomer structures

Table S28. Compared Gibbs free energies of 2-methyl-pent-1,3-ene non-radical isomers

S = 0 (Eh)	Terminal Olefin	Internal-cis Olefin	Internal-trans Olefin	Isomerized Olefin
	-235.3097	-235.3080	-235.3095	-235.307
Energy Comparison	Lowest Energy	Second Highest	Second Lowest	Highest Energy

Table S29. Calculated coordinates of 2-methyl-pent-1,3-ene radical byproduct isomers: primary radical structure

C	2.86828909379728	0.24221530407834	1.77278853596027
C	1.95097059042582	0.28334934445281	0.60706873767142
C	0.83067330307598	-0.41733410588801	0.49741595177513
C	-0.13832993939669	-0.39794352858801	-0.64619612173949
C	-1.49627418397962	0.12580690983716	-0.19084767379832
H	-2.23588077204183	0.02338201739200	-0.99526877568832
H	-1.41908240064333	1.18630718271312	0.08210194113216
H	-1.85365426256002	-0.43209028940234	0.68349395478160

H	3.87707232383660	-0.07533966285340	1.46790750322849
H	2.50302903587342	-0.44986537734144	2.54114743968106
H	2.97428323703969	1.24077086577891	2.22324930192393
C	0.34313589668296	0.27782068869884	-1.86333538505981
H	2.23443703320956	0.95074334952062	-0.20990087954052
H	0.54458602499800	-1.07578843877678	1.32308914401366
H	-0.34997756806706	0.81215987842899	-2.50835380617669
H	1.35719427292692	0.12170995169799	-2.22307265703443
H	-0.29814168517769	-1.47025408974881	-0.90806721113016

Table S30. Calculated coordinates of 2-methyl-pent-1,3-ene radical byproduct isomers: secondary radical structure

C	3.07270101389865	-0.36499633294761	1.89663652760739
C	2.20594519208429	0.03559760699537	0.76798665067106
C	0.84769288270428	-0.17634438226365	0.73480058689301
C	-0.05476169092122	0.15364838048049	-0.26331377552600
C	-1.49662524122281	-0.16047129504769	-0.10797602729533
H	-1.84759164619262	-0.81718557670550	-0.92247859100342
H	-2.10865755469104	0.75572015268687	-0.17054416044443
H	-1.70437724945514	-0.65213464188930	0.84843255458732
H	3.85596498489358	-1.07023754519756	1.57170851044532
H	2.49032646913426	-0.84225504092458	2.69417381753133
H	3.60098637863018	0.50269335444193	2.32584798724169
C	0.32938206338384	0.82545810607973	-1.52894103858938
H	-0.21347659293788	1.77895961431956	-1.63949444705961
H	0.04264886090494	0.20635720809943	-2.39527089311475
H	1.40064269415088	1.03128683340958	-1.59418860947279
H	2.69455931306008	0.52403584340409	-0.07464817443737

H	0.42141012257571	-0.67069228494117	1.61249908196595

Table S31. Calculated coordinates of 2-methyl-pent-1,3-ene radical byproduct isomers: tertiary radical structure

C	3.00652624562489	0.02984770049970	1.74031877137071
C	2.12088454315958	0.24159521620346	0.57586348465731
C	0.75983724873035	0.04744861628376	0.60490512605102
C	-0.15842337338278	0.21341087685250	-0.41925808539861
C	-1.60015539319044	-0.04956363506806	-0.18731741411807
H	-1.97470390314218	-0.82806909354888	-0.87398502519555
H	-2.20430233650660	0.85148027950565	-0.39025682907133
H	-1.79318437948376	-0.37192509472360	0.84152377111727
H	3.77623987333983	-0.73072957832353	1.52714747610531
H	2.43628226577386	-0.29569647381150	2.61888292947695
H	3.55152308823352	0.95158945064476	2.00426433361469
C	0.20839981246599	0.65235341667253	-1.78769751920487
H	-0.32414139172698	1.58180856159741	-2.05008649394930
H	-0.10394267000033	-0.10042826607820	-2.53044988876506
H	1.28070071165692	0.82729618342186	-1.90597160899965
H	2.59652598310910	0.56996655559278	-0.34796547320504
H	0.34588367533900	-0.28122471572065	1.56256244551423

Table S32. Calculated coordinates of 2-methyl-pent-1,3-ene starting material and product isomers: 2- methyl-pent-1,3-ene

C	2.96254455047709	0.08552263316729	1.75697001907149
C	2.06552799688513	-0.00430095362348	0.57202761272456
C	0.75626104930866	0.23827900864651	0.59183958956036
C	-0.15759477755340	0.19024511452574	-0.54855110939566
C	-1.57748617955619	-0.13575898022105	-0.21871021040607

H	-2.21396354927185	-0.09511629191723	-1.10827911273567
H	-1.96437178424796	0.57744311997697	0.52449005311852
H	-1.65384525138357	-1.13685889805073	0.23072179941323
H	3.93143930604881	-0.38535331688159	1.55778436618839
H	2.51443799601051	-0.39636583694486	2.63621118406194
H	3.15590583055761	1.13621614934533	2.02292166726855
C	0.22489983170779	0.45445012474115	-1.79898807651920
H	-0.47614860229784	0.39279338675356	-2.62837763211712
H	1.24668576591692	0.74269247312507	-2.03464621315387
H	2.52625917607916	-0.28403960059236	-0.37733736556909
H	0.28596864131912	0.44213186794966	1.55855342848964

Table S33. Calculated coordinates of 2-methyl-pent-1,3-ene starting material and product isomers: 2- methyl-pentane

C	2.98713352487811	0.00746889450540	1.78615793206422
C	2.20495088019021	-0.24324399858494	0.51520250923976
C	0.72560415760213	0.04188262675625	0.66871050222206
C	-0.13556161340381	-0.26402325591341	-0.54818581629458
C	-1.60749534402839	-0.14194870187675	-0.20471692728621
H	-2.23584458574952	-0.39799800841321	-1.06783123621357
H	-1.84585727926217	0.88979157059273	0.09387784055504
H	-1.87590960642641	-0.80668678495142	0.62701347424998
H	4.05309824962069	-0.21368290073661	1.64742261015675
H	2.61268561236508	-0.62138429144728	2.60615751254711
H	2.89577334746562	1.05692859286113	2.09979698429106
C	0.20770084150575	0.60777542510795	-1.74123726637741
H	2.62375617153329	0.37565106601023	-0.29079270749768
H	2.33625637909252	-1.29291518023299	0.20680207678501

H	0.34574050575887	-0.54775164466828	1.51866784643078
H	0.58874487896943	1.10326582219306	0.93953036313947
H	0.05796319720031	-1.31348178148993	-0.83271887873809
H	0.05057675996248	1.66862032642975	-1.49493933020625
H	-0.43386908105224	0.36098398868235	-2.59722810502203
H	1.25060300377804	0.48054823517600	-2.05354938404542

Table S34. Calculated coordinates of 2-methyl-pent-1,3-ene starting material and product isomers: 2- methyl-pent-2-ene

C	2.12245656805375	-0.29231941797034	0.41179206462727
C	0.64096671670688	-0.41933122128949	0.37684747156482
C	-0.23960298177738	0.24433183694348	-0.37376406476556
C	-1.70171573734443	-0.03344017694314	-0.25965283712377
H	-2.11370858938366	-0.34725134258630	-1.23152795618839
H	-2.24785137628592	0.87683995583751	0.03383728046089
H	-1.90635535226014	-0.81723089655097	0.47750013546115
C	0.11212497747670	1.29596834409407	-1.37031653526279
H	-0.37356067792567	2.24778822637641	-1.10426248557030
H	-0.27060632930132	1.01775359154661	-2.36447341285528
H	1.18788013272833	1.47014180067864	-1.44828765345823
C	2.84864962869010	-1.62185351406734	0.17073298297062
H	2.13381369534151	-2.42451282225501	-0.04904962378368
H	3.43524706438769	-1.92924509473831	1.04472366634701
H	3.53425882566780	-1.55062539319377	-0.68219059636600
H	2.41189485916437	0.09498965935894	1.40206250184408
H	2.45872109119430	0.45527214825864	-0.31429900727112
H	0.23772748486709	-1.16825568349964	1.06459806936929

Table S35. Calculated coordinates of 2-methyl-pent-1,3-ene starting material and product isomers: (*E*)-4-methylpent-2-ene

C	2.88571075310181	0.21099893258268	1.78668792614543
C	1.99064515763254	0.23026311898498	0.60255897713653
C	0.81602058341500	-0.38182121420133	0.52986485054619
C	-0.13461108747693	-0.39338298663373	-0.62254897253364
C	-1.50561537261957	0.09748079157764	-0.17741504982830
H	-2.23780780472027	-0.01502084510505	-0.98771929912115
H	-1.45641763339571	1.15971245257368	0.10020837065625
H	-1.86291656727804	-0.46797240408617	0.69279745635204
H	3.86554996691977	-0.22111785196024	1.53212419273491
H	2.44855121153565	-0.37497505933163	2.60417254013529
H	3.07677351128220	1.23155695883340	2.15192612832074
C	0.34742564449470	0.36229099638129	-1.84190769377907
H	-0.25996784908137	-1.45291847469805	-0.91454765884419
H	0.47791441531597	1.42914581239529	-1.61240779441400
H	-0.38848057947317	0.27748072537774	-2.65102397546590
H	1.30553963246756	-0.02929929777482	-2.20583969506808
H	2.35021760588504	0.79531756379974	-0.25939152347965
H	0.47496841199481	-0.94003921871544	1.40790122050660

Table S36. Calculated coordinates of 2-methyl-pent-1,3-ene starting material and product isomers: (*Z*)-4- methylpent-2-ene

H	2.49603668205003	-0.02192531837901	1.70733706237951
C	1.98596212501743	-0.38667306506286	0.81282682393742
C	0.87806536409338	0.25002814152795	0.44560544060547
C	-0.01005438953496	-0.03206897692367	-0.71908694240085
C	-1.41198583473668	-0.38881275433274	-0.24786034643925
H	-2.06687532403176	-0.58887644973427	-1.10642406256564

H	-1.84601616584601	0.44282967125497	0.32562773152778
H	-1.39738085024103	-1.27711601224154	0.39562394733954
C	-0.04665295222521	1.15855982527093	-1.66514886380917
H	0.38919673635186	-0.89237377121582	-1.27322532631593
H	-0.43018190911228	2.04824395568975	-1.14521089243772
H	-0.70625386224640	0.95190901243191	-2.51856946110307
H	0.95545729667987	1.39063971286875	-2.04628377606928
C	2.63030938014001	-1.55303999389111	0.15571171314684
H	0.55542972746689	1.09498943356949	1.06162444722574
H	2.08066214964396	-1.89699252686119	-0.72543870513472
H	2.71726098166387	-2.39270634738119	0.86145698330891
H	3.65458084486701	-1.29902453659035	-0.15727577319557

Table S37. Calculated coordinates of 2-methyl-pent-1,3-ene starting material and product isomers: 2- methylpent-1-ene

C	2.95036346312825	0.05801418341314	1.79894811955802
C	2.17781078837276	0.04649792837333	0.49787415201369
C	0.68218776353442	0.06920895943004	0.71253834651847
C	-0.19237404017038	0.05827342607261	-0.50104729488335
C	-1.65652013695898	0.07914328647121	-0.21637081178740
H	-2.24495613246343	0.07789356361361	-1.13986504484990
H	-1.92258175856968	0.97078371271364	0.37149135307627
H	-1.94430032171610	-0.79497337841543	0.38755075924961
H	4.03177099765625	0.03795999401422	1.61483719146441
H	2.69413448931471	-0.81529117590630	2.41523178351268
H	2.72200869099652	0.96085039226248	2.38287738707243
C	0.27275959634801	0.03166203910104	-1.74544875853712
H	-0.40450474626384	0.02478441739875	-2.59741922942700

H	1.33620714940613	0.01559223876032	-1.96764960274045
H	2.47307862231448	0.91575166848459	-0.10828721447900
H	2.45025050722655	-0.84948053133239	-0.07931188113317
H	0.39606816415136	-0.79214423279544	1.34253084256034
H	0.41766690369293	0.95955350834059	1.31084990281146

Table S38. Compared single point energies of intermediates towards ligand hydrogenation without accounting for entropic contributions.

	**2**	H_2	**3**	**2-H_2**	**2H-H**
HS- S = 3/2 (Eh)	-3702.05	N/A	-3703.25	-3703.19	-3703.20
LS- S = 1/2 (Eh)	-3702.06	N/A	-3703.24	-3703.21	-3703.21
S = 0 (Eh)	N/A	-1.15	N/A	N/A	N/A
Δ (HS-LS) (kcal/mol)	8.26	N/A	-0.25	+ 9.13	+ 10.12

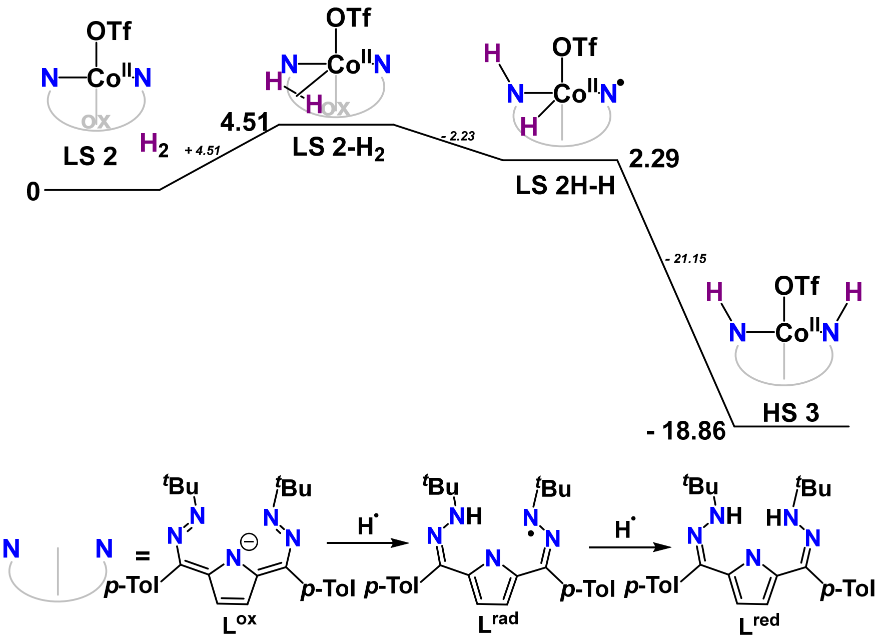

Figure S63. Simplified catalytic steps towards ligand hydrogenation (without accounting for entropic contributions).

Table S39. Compared Gibbs free energies of intermediates along catalytic cycle for ligand hydrogenation with accounting for entropic contributions.

	**2**	H_2	**3**	**2-H_2**	**2H-H**	**TS H_2 -> 2H-H**	**TS 2H-H -> 3**
HS- S = 3/2 (Eh)	-3701.52	N/A	-3702.68	N/A	N/A	-3702.639	-3702.642
LS- S = 1/2 (Eh)	N/A	N/A	N/A	-3702.659	-3702.656		

LS- S = 1/2 (Eh) outer sphere OTf	-2740.64	N/A	-2741.80	-2741.770	-2741.772	-2741.75	-2741.76
S = 0 (Eh)	N/A	-1.16	N/A	N/A	N/A	N/A	N/A

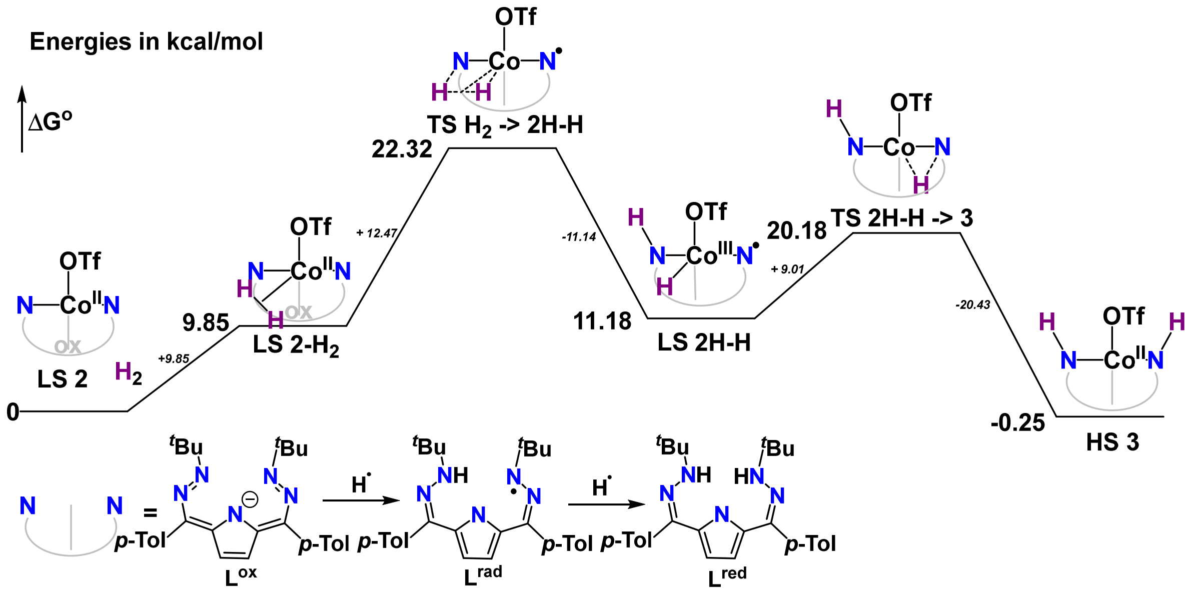

Figure S64. Simplified catalytic steps towards ligand-centered dihydrogen reactivity with triflate bound (accounting for entropic contributions).

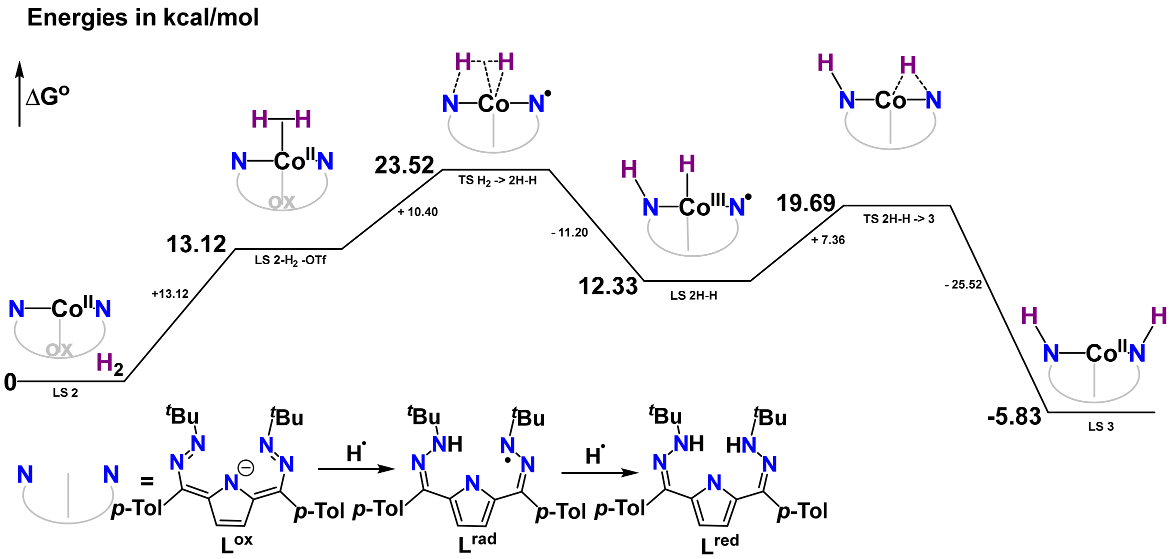

Figure S65. Simplified catalytic steps towards ligand-centered dihydrogen reactivity with the triflate anion outer-sphere (accounting for entropic contributions).

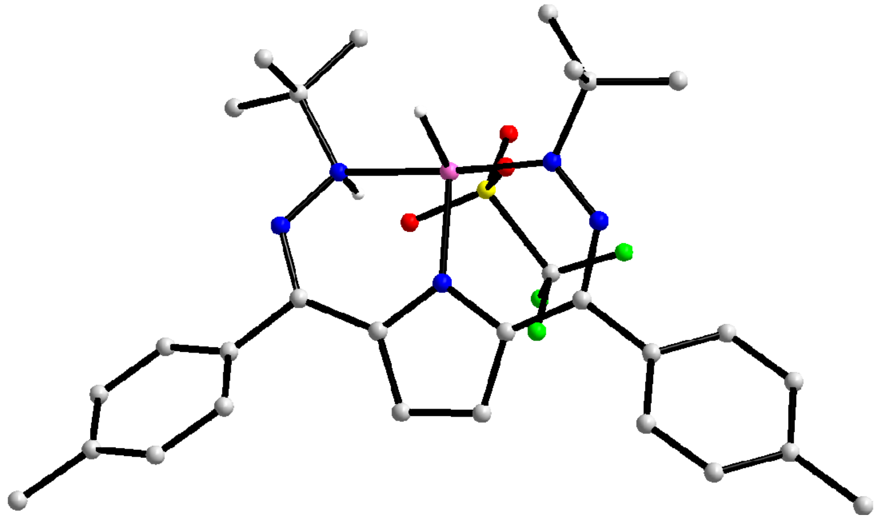

Figure S66. Calculated structure of high spin [($^{t\text{Bu, Tol}}$DHP-H)CoH]OTf (**2H-H**). All C–H hydrogen atoms have been removed for clarity.

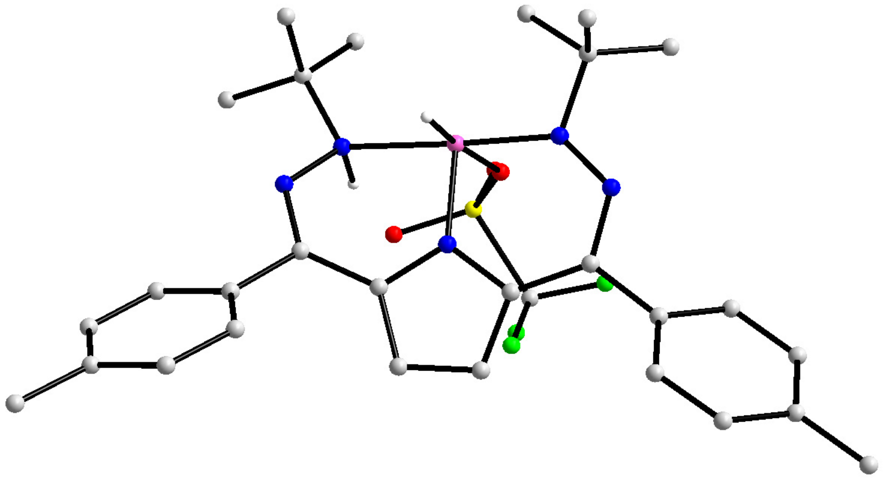

Figure S67. Calculated structure of low spin [($^{t\text{Bu, Tol}}$DHP-H)CoH]OTf (**2H-H**). All C–H hydrogen atoms have been removed for clarity.

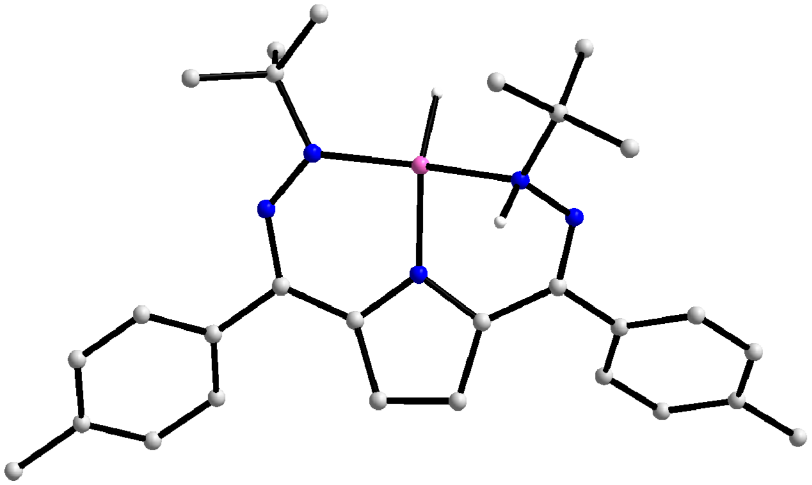

Figure S68. Calculated structure of high spin [(${}^{tBu, Tol}$DHP -H)CoH]$^+$ (**2H-H$^+$**). All C–H hydrogen atoms have been removed for clarity.

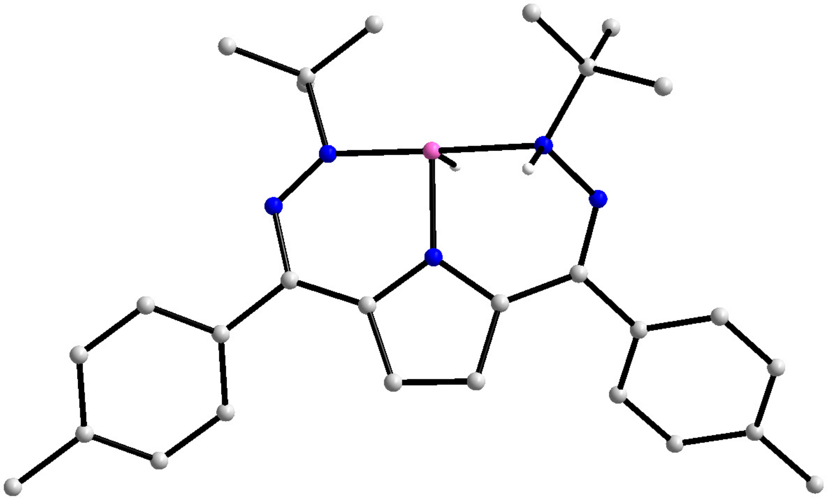

Figure S69. Calculated structure of low spin [(${}^{tBu, Tol}$DHP -H)CoH]$^+$ (**2H-H$^+$**). All C–H hydrogen atoms have been removed for clarity.

Table S40. Calculated coordinates of high spin [(${}^{tBu, Tol}$DHP -H)CoH]OTf (**2H-H**)

Co	6.45181453572060	12.85260101641201	5.22105373464912
O	5.79379080827970	14.71747646104187	4.70737078997113
N	4.88491311103222	11.79035881329290	5.28983773727762
N	6.35849665245940	13.17676886847695	7.22706689367485

N	5.79143851391906	12.22830854351614	8.08029967322086
C	4.16984696654165	11.49268142212910	6.40659908201043
C	2.90693590030153	11.01219767756025	6.03450178160616
H	2.11841347637662	10.71472843991900	6.70849085198615
C	4.74803280350037	11.56227736393791	7.72391710755220
C	4.14546600570130	10.75869057507308	8.80840963494647
C	3.72852115448837	9.44605153281170	8.61457805941013
H	3.82800114300600	8.98732977036396	7.63587935439909
C	4.01786812644400	11.31097029770813	10.07807545871046
H	4.33660894894942	12.33569455831587	10.23902476306330
C	3.48308458367359	10.57662497897216	11.11701746588853
H	3.37871348442137	11.03587275765033	12.09679709747968
C	3.20444173206554	8.71348235498654	9.66107162177715
H	2.89481015498310	7.68552732574972	9.48915538977806
C	3.07030714996353	9.26249587451687	10.93131731068345
C	7.56187962930362	13.76431834853550	7.93101148086738
C	8.12672431910855	14.87449576312146	7.07120440112636
H	8.41271473984071	14.51317431825907	6.07952153228116
H	9.02766322090618	15.26659132446529	7.55641514509850
H	7.41740760011223	15.70116789944382	6.95830651649577
C	8.59135370888128	12.67666539979363	8.14649775548082
H	8.16372152439214	11.84962137357510	8.72278010990469
H	9.43786486195786	13.09373674039853	8.70398319346838
H	8.95903845438933	12.29262560804089	7.18783385609484
C	2.51913897366573	8.45956911933403	12.06023692052550
H	2.12126102402924	9.10844793860150	12.84822682906311
H	3.30701545978944	7.83426984741548	12.50614719102882
H	1.72252326988968	7.78912256593785	11.71565244839293

C	7.11673481476524	14.34736585715671	9.26453846032690
H	6.32379690151024	15.09150014280858	9.11639858127828
H	7.97292280955377	14.84626565448902	9.73301888853102
H	6.75388850847872	13.56333960697323	9.93426950514889
C	3.41764477214533	15.72595334617878	4.35205369583797
S	4.97166382707709	15.76462097957829	5.35096667699167
O	4.55017086358553	15.39217156602009	6.68409372823988
O	5.49105427673424	17.08492711967490	5.16643710939974
N	6.71157165394430	12.51229293991281	3.37148652511995
N	5.84052230395897	12.03412991005671	2.57688946219216
C	4.12903900873740	11.46636595902728	4.21075321326740
C	2.86951454918557	11.00956938948304	4.65822265705064
H	2.03609869468008	10.72821218288895	4.03443258856233
C	4.62445305027023	11.59668325892952	2.89507800119843
C	3.81737544712908	11.21637668509877	1.72784431510074
C	3.08263759112797	10.03326926039353	1.67884612218805
H	3.10583069651861	9.35021236162175	2.52146696009248
C	3.80709700764304	12.03766605832619	0.60369268266004
H	4.38302867701101	12.95757416323212	0.61747982996722
C	3.07011275696112	11.70106249493059	-0.51367097385972
H	3.06923049686392	12.36900404951329	-1.37130717615481
C	2.35615017602766	9.69849738548407	0.55476298305554
H	1.80424227264284	8.76166070878996	0.53791415506233
C	2.32901447543102	10.52660466905638	-0.56225356494520
C	8.02379002767140	12.73070090692708	2.67872857147273
C	8.87228350051973	13.70976293285053	3.46213566197958
H	9.18576283562801	13.29404263133487	4.42440797823587
H	9.77832640836203	13.92330471739386	2.88432623066253

H	8.33873049117055	14.65247165137601	3.62792484231195
C	8.71685998465147	11.37973221176170	2.59032198649373
H	8.11206887167969	10.68183078308856	1.99995311099601
H	9.69417397747230	11.50141633919650	2.10751956775050
H	8.87114815009864	10.96170528362767	3.59286881861431
C	1.53785578178280	10.15567112472585	-1.77012674016938
H	0.47399816329694	10.04265461033406	-1.51880389222068
H	1.63058962731773	10.91749499054152	-2.55071274085595
H	1.87944044622873	9.19447549369173	-2.17900513527674
C	7.78187684494917	13.30164941014620	1.28859907066588
H	7.20917701298752	14.23498160204143	1.35521748128409
H	8.75275636708639	13.51931783056345	0.82939294156221
H	7.23923829314231	12.59544865189923	0.65671731713703
F	2.56847221882159	16.63458854489101	4.81309478404940
F	2.84014486637349	14.53263433089538	4.42403220250206
F	3.67725122808174	15.99290369660484	3.07801829501455
H	5.66244340836786	13.95254825738548	7.15065151489326
H	7.77521382423320	12.17160336974100	5.43593251867172

Table S41. Calculated coordinates of low spin [($^{t\mathrm{Bu},\ \mathrm{Tol}}$DHP -H)CoH]OTf (**2H-H**)

Co	6.48184235250837	12.77470501038003	5.14684273451931
O	5.77406755564657	14.67140389224754	4.56692946914705
N	4.87050557715576	11.93680332679661	5.23663621271516
N	6.38221275561532	13.25411571545363	7.12049876628544
N	5.86245673876537	12.28977554792591	7.99259255145728
C	4.18763875613914	11.63313233337331	6.36081865784142
C	2.90189822311086	11.18097742363699	6.00580292260586
H	2.12445960752689	10.90343272712107	6.70064560713000

C	4.80157278616957	11.64462076272365	7.66051703116947
C	4.23812679054496	10.79155734526470	8.72713143160407
C	3.79324834389963	9.49687370028020	8.48231324355155
H	3.83745280696526	9.09342621511156	7.47571446148088
C	4.17988322683195	11.27355093525823	10.03025011109449
H	4.52046093024135	12.28400631122168	10.23109231321391
C	3.68674821921553	10.48825603888736	11.05226732997975
H	3.63701657060834	10.89267826089793	12.06014981836305
C	3.30967001570274	8.71304095311308	9.51127837004812
H	2.97634613933745	7.70033170032416	9.29824420346502
C	3.24633893410601	9.19132289892707	10.81490903598863
C	7.55298426669379	13.89744982489928	7.84068926673461
C	8.01784634966709	15.12354243102563	7.07933353051477
H	8.34236091296077	14.90104202670183	6.06309860945643
H	8.87482152921078	15.54867695380099	7.61397276395987
H	7.23419384700436	15.88693318493777	7.03945759063426
C	8.66232262776898	12.87604522660467	7.97933260077204
H	8.28827434792194	11.97723718423274	8.48063772844209
H	9.47059293619755	13.30427951352141	8.58330043745413
H	9.07751147863196	12.59143579479757	7.00661993827664
C	2.74034342382319	8.33369967764754	11.92476789064743
H	2.34866656312718	8.94365540890709	12.74651746946398
H	3.55253916271532	7.71069460374093	12.32832845497287
H	1.95005846389328	7.65926796653066	11.57417595885761
C	7.09080696569251	14.35825404985084	9.21772810563395
H	6.21711216284985	15.01611998515138	9.12721276319770
H	7.90273719478799	14.92873998157812	9.68347818064944
H	6.83797776040489	13.51128314632746	9.85922469178647

C	3.41304802592813	15.71749119421276	4.18054506103955
S	4.98673819297387	15.77046953764405	5.14942551750920
O	4.57714564759738	15.50283044627813	6.51220262416757
O	5.52590516477852	17.06940683770275	4.87405444496933
N	6.65182708279280	12.36112376335698	3.37134112793886
N	5.81774831913322	11.97566659298683	2.49840305869550
C	4.10004185751388	11.61015296885149	4.16089262501567
C	2.83692751944953	11.18585023370196	4.63884789330288
H	1.99327710234318	10.91654924544996	4.02477681867155
C	4.57141674436224	11.63462761799085	2.83311074198031
C	3.73753341436090	11.23890497091810	1.69252605586826
C	2.95968830286225	10.08259597451586	1.69421249261529
H	2.95924415776960	9.43512357735403	2.56458328106309
C	3.75625310740693	12.01144918641065	0.53434376571870
H	4.36328752834204	12.91092773326276	0.50971774340160
C	3.00637214222250	11.65442944872261	-0.56821723554428
H	3.02952376527443	12.28460128209943	-1.45361261696423
C	2.22066839143872	9.72726234411308	0.58486968893056
H	1.63487065856564	8.81135594027974	0.60786918269430
C	2.22327323093548	10.50682435262354	-0.56704156000578
C	8.02644807391720	12.59267781088102	2.90164791720455
C	8.62735864181126	13.45905457838378	3.99110260498187
H	8.40602225789834	13.02029793263094	4.99911625212887
H	9.72225870023411	13.45540901587889	3.95576974602621
H	8.26556396977466	14.48837815469761	3.92915774246389
C	8.73517198701436	11.24975524797014	2.81139921025512
H	8.22347395667948	10.60775817102996	2.08564303931309
H	9.76942882118344	11.40633628287066	2.48161248305770

H	8.74455245841516	10.75105729694660	3.78768699275643
C	1.41831774342846	10.11298420682208	-1.75851954327297
H	0.34991774231891	10.06101054014806	-1.50628256337858
H	1.54691146475386	10.83034421544688	-2.57530789436809
H	1.71642003548364	9.11817022230590	-2.11800742526034
C	8.05264513929627	13.31292447154550	1.56853675186855
H	7.44846745637180	14.22638678962319	1.61611873925120
H	9.08950121380721	13.58648189165558	1.33774542463920
H	7.66865849594154	12.67011740635619	0.77137897650255
F	2.64213643226029	16.74471472848084	4.51520799615950
F	2.74276135927638	14.59628751465134	4.41893505675603
F	3.66649372336945	15.78648825685229	2.87707737298938
H	5.66191879325625	14.00158979443676	7.02518723404973
H	7.07289485401377	11.51197416270898	5.50232292169300

Table S42. Calculated coordinates of high spin $[(^{tBu, Tol}DHP\ \text{-H})CoH]^+$ (**2H-H**$^+$).

Co	6.69811442748852	12.18944119545528	5.37872670984087
N	4.92914818963639	11.59429198253227	5.37186941260400
N	6.33651972256815	13.06233255181936	7.13943827954128
N	5.89192588258893	12.09737690877636	8.04935580005301
C	4.16175921379896	11.46022779762739	6.48422222488969
C	2.82097780086236	11.28846617797707	6.10388108709740
H	1.98201776322544	11.17489377691036	6.77393755263356
C	4.80289709284125	11.44608304447751	7.78143609904816
C	4.25376486307447	10.61732890784931	8.85668534446549
C	3.55660250103406	9.44260845745502	8.58305157974232
H	3.41243007662273	9.12445701135998	7.55576830719933
C	4.44072697020547	10.98346381731206	10.18890796374648

H	4.97575483262820	11.89946387114573	10.41530418345265
C	3.94161543653272	10.20216322379229	11.20690750517371
H	4.08568370704292	10.51296635741575	12.23833050129516
C	3.06647261923886	8.66293271806302	9.60946787645432
H	2.53348390886126	7.74570400526717	9.37388189169554
C	3.24783321994925	9.02452012688928	10.93970932729350
C	7.31305334214328	14.00495450116460	7.79353106302598
C	7.60325422342258	15.08840147875584	6.77635096898612
H	8.09188268757074	14.68273528161593	5.88568836316352
H	8.27696301891912	15.82719956959780	7.22308396119902
H	6.68236040549232	15.60714587963050	6.47565488780049
C	8.56464454723754	13.24945623194704	8.17624176495200
H	8.32582195603587	12.42319377400839	8.85362070729235
H	9.24949303303828	13.93505775923375	8.68750262156726
H	9.06910838089197	12.85129814943341	7.28861125647642
C	2.73346603900392	8.16948417180095	12.04454347950938
H	2.48797451762379	8.77052378683710	12.92728583533960
H	3.49972757481539	7.43816346892739	12.34289747840460
H	1.84504476638310	7.61021388850868	11.73082138763246
C	6.64641183122360	14.61193748178016	9.01667763838760
H	5.70999739770667	15.11653675749665	8.74207037858922
H	7.32104873950318	15.35596192178985	9.45407859954373
H	6.43251140628941	13.84586218358057	9.76754802896543
N	6.84426512031070	12.28559794876483	3.50572592946063
N	5.93805573244992	11.88125491609465	2.70445285224854
C	4.12829289686131	11.49255155693048	4.28582722990126
C	2.78956325215574	11.32506737615393	4.73024572196997
H	1.91429590243739	11.27244463838158	4.10193242805401

C	4.66849147829084	11.56849664890643	2.98380791352995
C	3.85865305487306	11.27881143353710	1.80337997309703
C	2.89345459620465	10.26904949111228	1.80066792191449
H	2.75572483159232	9.65243699293494	2.68227803917350
C	4.06038293185931	11.99898048770841	0.62546102547895
H	4.81886878450169	12.77412055197092	0.60294294710495
C	3.30058352286209	11.74357110557004	-0.49465598187718
H	3.46341778966359	12.32980800418393	-1.39487765786008
C	2.14885743523257	10.01136979598173	0.67162395514135
H	1.41803367430690	9.20688847497509	0.68701245260275
C	2.32767958441523	10.74835670740546	-0.49622685195717
C	8.16488373072135	12.47507671097947	2.81205489210121
C	9.01723954523142	13.42420266140217	3.62500523003359
H	9.20856112492424	13.03739761721255	4.63249394064719
H	9.98514640618046	13.54120143086321	3.12591328020783
H	8.54344029992565	14.41046898610217	3.69424324409104
C	8.82657579417318	11.11198153793045	2.69281582750913
H	8.18412089390859	10.42765478578358	2.12668859075801
H	9.78108668395666	11.22077612449412	2.16441007940288
H	9.02054467640037	10.68644754570479	3.68468103030651
C	1.50740575197775	10.46781675830163	-1.70564764656942
H	0.44663248446429	10.67680120303402	-1.50580973521982
H	1.82834419246512	11.08008521283049	-2.55377935401989
H	1.58083137150269	9.40820090509877	-1.98605441839746
C	7.93362744574145	13.07629283507424	1.43407549787489
H	7.34350152559039	13.99785506136582	1.50957505502486
H	8.90928976750418	13.32204249908263	1.00068550380577
H	7.41932323302284	12.37521112771727	0.77313448566896

H	5.53116246128017	13.64454905735890	6.84851234357948
H	8.05607992751047	11.58141359081659	5.57883418814994

Table S43. Calculated coordinates of low spin [($^{tBu, Tol}$DHP -H)CoH]$^+$ (**2H-H**$^+$).

Co	6.65919959367125	12.51568474552370	5.17565744938332
N	4.95383868267680	11.93845917049109	5.26500584090481
N	6.43477439237559	13.19001542793400	7.06426647885996
N	5.98621880480071	12.22279410519593	7.97977566185215
C	4.24181711656555	11.70828461096858	6.39938659863267
C	2.90968230975310	11.43881651859193	6.05242996859651
H	2.10750374588576	11.25822202862165	6.75176826615940
C	4.89433876038391	11.60896206857706	7.68527779377873
C	4.34282650442261	10.71364148583206	8.71238356078523
C	3.73008044791064	9.50827477114606	8.38136380694124
H	3.64274689675307	9.21261728626718	7.34087271933273
C	4.45372737210447	11.05172533592873	10.05826756637941
H	4.92647650056639	11.98957916215153	10.33006507414193
C	3.96016444909957	10.21470484681772	11.03650023611734
H	4.04363237268675	10.50659492129622	12.08013007948112
C	3.24627337431575	8.67268031939867	9.36668604900919
H	2.77869763370460	7.73269523543316	9.08535519410021
C	3.34965009699858	9.00753863745720	10.71229165439537
C	7.41943123615721	14.09817943842544	7.76787839137623
C	7.72309674588316	15.26230744796515	6.84550359902076
H	8.33320427389808	14.96914016682319	5.98955972593636
H	8.28572930954609	16.01608277463112	7.40631535922127
H	6.79873625983406	15.73593032612219	6.48555069968746
C	8.66563179478043	13.31486527562581	8.11378897396789

H	8.42274009094531	12.48076381778787	8.77962407996729
H	9.37301376182494	13.97799958797980	8.62441751722662
H	9.15561167973341	12.92184501341012	7.21632090551329
C	2.83645519047302	8.09352225798496	11.77083570436305
H	2.61106920132011	8.64238058211209	12.69158854719223
H	3.59118297128324	7.32996129808966	12.01132573914517
H	1.93330555097963	7.57036097707197	11.43541785470772
C	6.76004156629676	14.63805154396961	9.02758537427040
H	5.84534170686149	15.19441000156289	8.77991597439392
H	7.45478357277655	15.32591624927937	9.52182538526733
H	6.50926648508699	13.83113618569829	9.72067128731527
N	6.74155079449175	12.18689112897988	3.40744664890141
N	5.88186881967679	11.87719594308549	2.52720820895129
C	4.13750650792062	11.70322201880227	4.19116726880834
C	2.83379106723678	11.45913225821078	4.68327692374213
H	1.95311492889757	11.31234062787861	4.07937930925646
C	4.60820304864168	11.63874541608252	2.86520254307042
C	3.75033464191348	11.27577973598881	1.73950205710791
C	2.80903922531610	10.24808673321326	1.81613163194146
H	2.71366822972680	9.67065424957972	2.72901171253526
C	3.89762654417877	11.94202011953371	0.52314001547429
H	4.63637273284963	12.73228311200183	0.43800265584067
C	3.10506521582268	11.62038026770413	-0.55767614418457
H	3.22538963681451	12.16829857132661	-1.48831021709582
C	2.03215059370617	9.92355688409997	0.72597948967824
H	1.31899702075281	9.10693737274927	0.80534655954288
C	2.15442649885532	10.60803840930213	-0.48010491576126
C	8.13191890176813	12.37085275414377	2.96973436969735

C	8.70383680271395	13.17500496174512	4.12396463149952
H	8.47746628382094	12.69553243262765	5.12750900971156
H	9.79983841497244	13.15974029900276	4.14147910754646
H	8.36757356297459	14.21535756366695	4.08796101637101
C	8.79748966339228	11.01060507075846	2.84374241584836
H	8.28267122007851	10.41592630558512	2.08107852040192
H	9.84266426771940	11.14705651178451	2.54169011385781
H	8.76990330453183	10.47231398506237	3.79816328293860
C	1.29984314456828	10.25421555453644	-1.64658672201903
H	0.23833016099333	10.40816336736849	-1.40648913994938
H	1.55084516911682	10.86227064103960	-2.52100588567777
H	1.42203175660442	9.19409422400033	-1.90826037076081
C	8.22060606266110	13.14598869649945	1.67194044974965
H	7.65546679389828	14.08327631153528	1.73895163030769
H	9.27316377452913	13.38092217148462	1.47356234149406
H	7.82869224622444	12.55071225235852	0.84229486964047
H	5.62746594725277	13.80248336689096	6.84886790260622
H	7.13770656302193	11.20890705919790	5.50960959150329

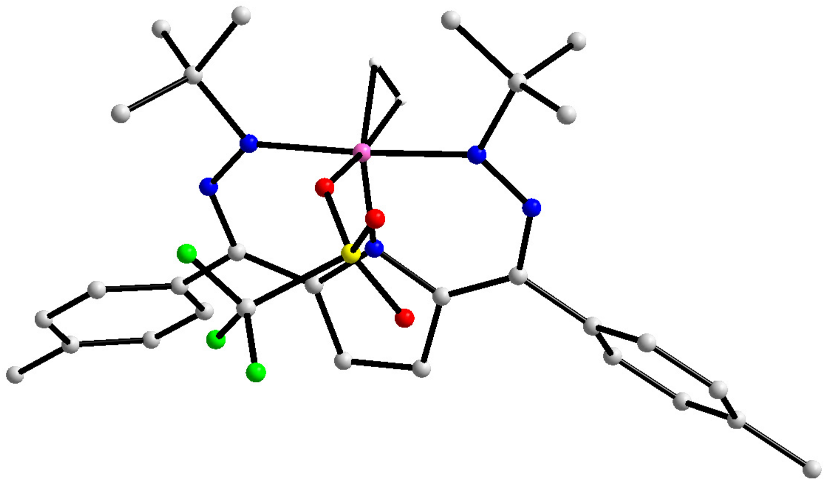

Figure 70. Calculated structure of low spin [($^{t\text{Bu, Tol}}$DHP)CoH$_2$]OTf (**2-H$_2$**)**.** All C–H hydrogen atoms have been removed for clarity.

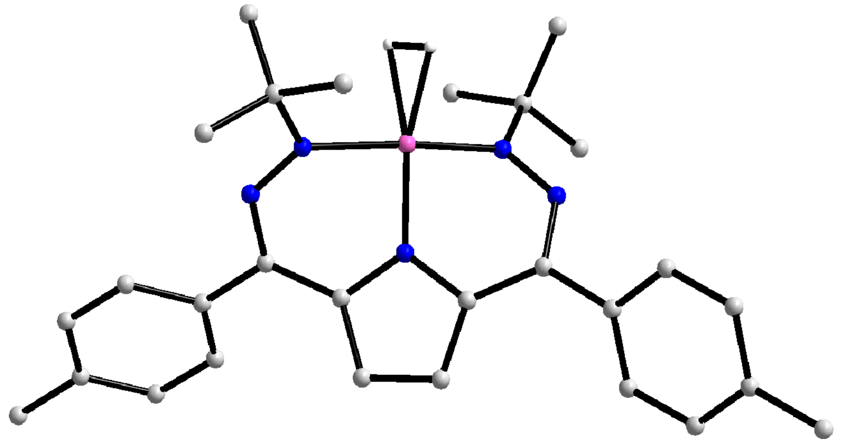

Figure S71. Calculated structure of high spin [($^{t\text{Bu, Tol}}$DHP)CoH$_2$]$^+$ (**2-H$_2$$^+$**)**.** All C–H hydrogen atoms have been removed for clarity.

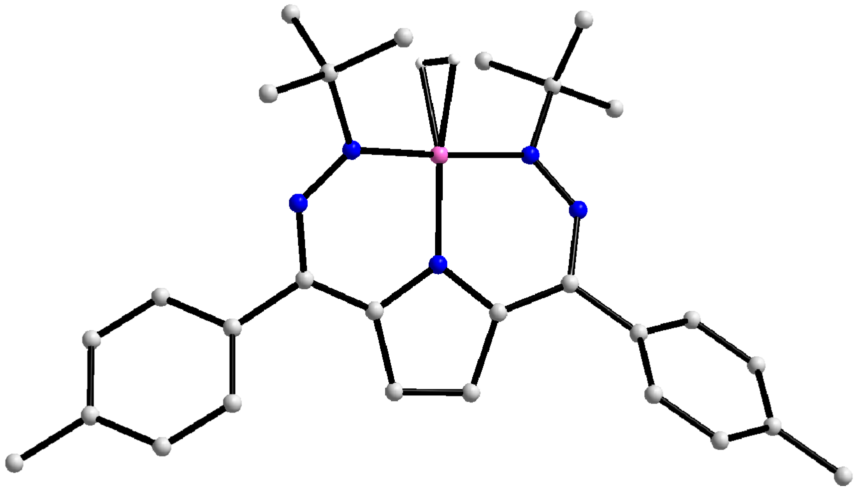

Figure S72. Calculated structure of low spin [($^{tBu, Tol}$DHP)CoH2]$^{+}$ (**2-H$_2$$^{+}$**). All C–H hydrogen atoms have been removed for clarity.

Table S44. Calculated coordinates of low spin [($^{tBu, Tol}$DHP)CoH2]OTf (**2-H$_2$**) (no optimized geometry for a high spin isomer could be found).

Co	6.47150574049633	12.47277316436262	5.27195139620523
O	6.06761265080637	14.49465106290502	5.27811226574305
N	4.81835431588970	11.54260711024438	5.28172715680755
H	7.23005410118617	10.74162335440445	5.37765659111263
N	6.56661152410094	12.40388152700150	7.15014048945440
N	5.72991884537954	11.96229458897595	7.98385995733116
C	4.08065962817530	11.26203797006276	6.37932302088978
C	2.79193820074963	10.77683982959938	5.96603719328988
H	1.97763361952026	10.53129836246004	6.62929620506512
C	4.54198643703976	11.42519020532504	7.67903843875059
C	3.76137937674467	11.00370042835887	8.85085612239672
C	3.12981410896097	9.76382850776612	8.91579635125364
H	3.21863545852302	9.07219898237796	8.08366452897745
C	3.66975528559099	11.84210355468485	9.95746862441815

H	4.15965959455913	12.81023662347645	9.92798365130907
C	2.95399496954877	11.46255940742147	11.07553082117020
H	2.88741384574575	12.14322398614768	11.92046942571200
C	2.41869887533372	9.38955210717431	10.03784883507308
H	1.94383139095047	8.41162721716596	10.06582398184329
C	2.31217622040875	10.23195044660112	11.13907796436236
C	7.81490108052995	12.85277921758346	7.87446170752383
C	8.78628495187311	13.48802786597224	6.90907322809922
H	9.13042665130925	12.77450939566374	6.15399054025888
H	9.66927973514212	13.80863984912133	7.47206268477290
H	8.35606535170828	14.36890594318963	6.42595824002114
C	8.44681955080005	11.61334667337280	8.49154156133568
H	7.76406264308923	11.15891934627058	9.21632936551915
H	9.37385830751321	11.89956643047349	9.00238598087579
H	8.69149978673914	10.87564226242026	7.71687190903573
C	1.54072426050561	9.81871157361924	12.34647258419039
H	1.95617954993941	8.89766066612428	12.77882178104865
H	0.49272826409647	9.61287087717624	12.08755246784624
H	1.56109511533458	10.60057787827819	13.11239021317309
C	7.44080083816000	13.85981055000473	8.94931733379551
H	6.97047143751421	14.74418738105202	8.50588138629434
H	8.35563106353250	14.17363451683898	9.46653146360428
H	6.75735336661277	13.41347613338001	9.67639907107955
C	4.27080053857685	16.23227910066803	4.62843249569470
S	5.15497900032989	15.39290713936368	6.01783678731934
O	4.11834268999395	14.71080247553258	6.74003660753677
O	5.84875301256098	16.45693818606153	6.68855755666143
N	6.48651548916434	12.51695114258201	3.38097340866200

N	5.67254761890253	12.00086695852041	2.56585448000440
C	4.09260677670402	11.20475721287571	4.19478966447326
C	2.80147984182859	10.73503016058966	4.61882110566798
H	1.99635572716091	10.44983309278275	3.95987084300758
C	4.55338530692499	11.34331304015883	2.88949167988153
C	3.83701168631542	10.77590652027552	1.73928929677891
C	3.27539946140775	9.50088491275386	1.78461378608830
H	3.36875872491254	8.90699521731629	2.68837181131090
C	3.74606153518848	11.48470452333822	0.54453693083139
H	4.18927692665785	12.47329957139943	0.48310986658266
C	3.10171433689230	10.94643235013040	-0.55135262111099
H	3.03720585146231	11.52590630392075	-1.46881138109434
C	2.63336177374428	8.96881232958324	0.68536984534580
H	2.21413591504323	7.96721144013925	0.74610220824593
C	2.53026445963916	9.68051120161123	-0.50472606452587
C	7.59731858609233	13.19302533128056	2.60745530260313
C	8.65271268254089	13.73855803308735	3.53936285462593
H	9.16080069577193	12.93636797539139	4.08438177367143
H	9.41138669705682	14.24686163703836	2.93447527229356
H	8.23940359846554	14.46782745543929	4.23931117352916
C	8.23469835225145	12.16042012186554	1.68913127748254
H	7.50967342246572	11.78761626489786	0.96063401691807
H	9.07255457555252	12.62690707500317	1.15755594872794
H	8.62247313290913	11.31532462524708	2.27275385300165
C	1.83816184935360	9.09463222809405	-1.68851080136565
H	2.29880947424664	8.13913159910465	-1.97519879195190
H	0.78213825243035	8.89153049616174	-1.46121506718669
H	1.88259521832978	9.77299194361468	-2.54660170532400

C	6.99047968140896	14.33202132743845	1.80399944338224
H	6.55623222945489	15.08495949326063	2.46958777394345
H	7.77824171178580	14.80486139218408	1.20490599967182
H	6.21375621218880	13.95430893507703	1.13195373846601
F	3.39521106821056	17.10785196628282	5.10859814189514
F	3.61192870483869	15.34926170253739	3.88156404936429
F	5.12461923891735	16.88361387272116	3.84201018143195
H	7.83089179820713	11.07316864761061	5.00461271781792

Table S45. Calculated coordinates of high spin [($^{tBu, Tol}$DHP)CoH$_2$]$^+$ (**2-H$_2^+$**).

Co	6.52866870507974	12.43260196608984	5.37722031168163
N	4.99621692645696	11.40902041915177	5.35804296652141
H	7.77371860550147	13.52968573241904	5.78389478552872
N	6.89175910708536	11.72272645154579	6.99098680286456
N	6.03526904428657	11.61209562112869	7.95136351126774
C	4.24284814236949	11.12489424587402	6.45034412532182
C	3.05191753979267	10.51393138464750	6.01402144394578
H	2.22672669291454	10.20322040839461	6.63283949491997
C	4.74840321173557	11.41391652780406	7.75809197844664
C	3.91240140293663	11.45010752753450	8.95771006005765
C	2.95104794997118	10.47902211613044	9.23448553172635
H	2.81233581827054	9.64195880856587	8.56079426591156
C	4.10916775227728	12.46723395866452	9.88853189153091
H	4.86278733716368	13.22504566443205	9.69599222961661
C	3.34895891875825	12.52840570287589	11.03793599923879
H	3.51126562504406	13.34075402772376	11.74130965029588
C	2.20783695225043	10.53907086860746	10.39334837638362
H	1.47856103458665	9.75923702102403	10.59782024365641

C	2.38191571030054	11.56825768917656	11.31372751774012
C	8.22012151602924	11.10234743698717	7.28800159524715
C	8.77362658192608	10.57859827176389	5.97065299324803
H	8.09779628925360	9.83196476096014	5.53535600074654
H	9.73895079082873	10.09546567059469	6.15776862362116
H	8.95730696781725	11.37281268890098	5.23575581798004
C	8.05848640572328	9.94141791383437	8.25249623218127
H	7.71709846678672	10.28228897204114	9.23384128477760
H	9.03189551672175	9.45176524604249	8.36815599093922
H	7.34266000326912	9.20815646074727	7.86258847093924
C	1.55772305197850	11.62969018684996	12.55241397727652
H	1.59857847385036	10.67500296587749	13.09327014399113
H	0.50373741988562	11.81745972182200	12.30297290036484
H	1.90145154520014	12.42734574152061	13.21794971015392
C	9.12916179057869	12.16175997453960	7.89418642571055
H	9.30870722599972	12.99321916657616	7.20369167936256
H	10.09731474897346	11.70428457594540	8.13073605917243
H	8.68892782953450	12.55216522621419	8.81901793290291
N	6.02525330480916	13.10879125823821	3.65138180829760
N	5.54535479524396	12.36510889532815	2.72504649077785
C	4.35657382129683	10.96994092455998	4.25400331972408
C	3.13957455812526	10.38500915894561	4.64558949343760
H	2.39925992730428	9.96548200253424	3.98164408798888
C	4.85426096559293	11.26441970431813	2.94370422703439
C	4.53050431673215	10.45290767134495	1.78429218566019
C	4.21632053030998	9.09723646619337	1.91292277933392
H	4.21678750218523	8.63588381946044	2.89472458444072
C	4.55853702246364	11.00475808230861	0.50078264874972

H	4.79122361741864	12.05754052658080	0.38451637821979
C	4.27290085077453	10.23259172576597	-0.60191870177653
H	4.28830754205180	10.68649516034832	-1.58929966191528
C	3.93793401284297	8.33290307656991	0.80256611363644
H	3.70887573015866	7.27744122135647	0.92551489375025
C	3.95378404926391	8.88307999346376	-0.47708379969693
C	6.14312276452935	14.54856479587380	3.27017231954790
C	7.55645234561514	14.81684225800972	2.77294914931985
H	7.77139170594013	14.19613849589324	1.89535557774202
H	7.63781501744583	15.87215512018651	2.48616992877974
H	8.31373385643328	14.62118879956734	3.54053356572106
C	5.14516134470365	14.89444073485562	2.17838169654624
H	4.12579947974538	14.63079198961113	2.48399372524660
H	5.18877948147520	15.97493035737893	2.00231893399797
H	5.37836451228280	14.37783330395506	1.24357926003627
C	3.62866012428189	8.05209186088786	-1.66746909230320
H	4.20661356944379	7.11827234748319	-1.65962679113247
H	2.56434333600372	7.77433443529870	-1.65946010171910
H	3.83649767747231	8.59110123619905	-2.59698972818712
C	5.81990502403880	15.37074048581112	4.50888131101150
H	6.54048511075419	15.21925919626268	5.31989139197897
H	5.84793041522536	16.43403867936477	4.24734024488244
H	4.81371540160402	15.13224565640500	4.87480323855338
H	7.99177518529215	13.26343943662952	5.05132149704322

Table S46. Calculated coordinates of low spin [($^{t\text{Bu, Tol}}$DHP)CoH_2]$^+$ (**2-H$_2$$^+$**).

Co	6.46888832179415	12.37717213038368	5.34199224728397
N	4.87495776196703	11.45682196492320	5.33507726533927

H	7.56725794646326	13.56615107630850	5.52728821749965
N	6.75704643535459	11.98834721116622	7.07571076646073
N	5.89818880215895	11.67772243289204	7.95529807047710
C	4.13510499537438	11.15857194916102	6.43360839713627
C	2.88415911677867	10.60293844206929	6.00754640304410
H	2.06830031560399	10.33166817705765	6.65842088394281
C	4.62578223871348	11.33659054882136	7.72086836220835
C	3.83777931993746	11.07571648001971	8.93371229981617
C	3.08524317774525	9.91657510366974	9.10335037717989
H	3.08188746617279	9.15558991881736	8.32971328096511
C	3.87206501459590	11.99506125382011	9.97688214379281
H	4.46383675965212	12.89908058687552	9.86978332437093
C	3.15674937313821	11.77508227073728	11.13699524473694
H	3.19159241335239	12.51418953607031	11.93304221673167
C	2.37736736501822	9.70126396926368	10.26759096340065
H	1.80855662337175	8.78204803272509	10.38197924193359
C	2.39313278276920	10.62711156925447	11.30573501326637
C	8.15211978187903	12.06685158021845	7.69642815472530
C	9.16748195322418	11.46456865969748	6.73221787219777
H	8.81186630270371	10.50036057389863	6.34644746793011
H	10.09884667777717	11.28567058807759	7.28116533002601
H	9.43105282355145	12.11283764413677	5.88999212221210
C	8.19267905925556	11.24329821934488	8.97530522503153
H	7.54239905954961	11.65829325916587	9.74771257066236
H	9.22490961046653	11.24805870733660	9.34219605315877
H	7.89410234456463	10.20627186185347	8.78211303219355
C	1.62121187158497	10.38516615952251	12.55719212286502
H	1.89642056703882	9.41971653346545	13.00274771294123

H	0.54318132186980	10.35092040938630	12.34575988324014
H	1.80412620391083	11.17467886087472	13.29291857732131
C	8.46837772397880	13.51407762114497	8.03715199490236
H	8.51604851832263	14.16712671757963	7.15836803714119
H	9.44848277712906	13.55259636844216	8.52794157153701
H	7.71629494908993	13.90912264671890	8.73012147611607
N	6.23987386996886	12.84283334029836	3.50047399186184
N	5.52812848546720	12.22820438386176	2.66154985581429
C	4.18684214849239	11.04866460442677	4.23957415005035
C	2.93031265564468	10.50452769295914	4.66113142008750
H	2.15929769572144	10.14412554699095	3.99736195748100
C	4.63263887834093	11.27055846665356	2.94187876650739
C	4.08435758796021	10.57237106727281	1.78327261148458
C	3.70208457939331	9.23274172062345	1.86786094031403
H	3.82494277070101	8.69630926061739	2.80378825738088
C	3.96847559595936	11.21323070463614	0.55066412579697
H	4.27190051451758	12.25044400233486	0.46257560471879
C	3.46838087237987	10.54308143006674	-0.54528500124737
H	3.37738945020994	11.06679269134552	-1.49321558538040
C	3.20562982563154	8.57085681973498	0.76609303621118
H	2.92682782170964	7.52373235363868	0.85412088764750
C	3.07350362990605	9.21192090989132	-0.46231425918049
C	6.95957833437009	14.01602228753243	2.85733428296813
C	8.43242535440051	13.67245674029418	2.68767094300395
H	8.53873012851095	12.76251129907793	2.08524649076531
H	8.92353266962082	14.49831653651213	2.15936048410161
H	8.96280401455946	13.53326255388044	3.63462460361614
C	6.37768388910770	14.30950688404998	1.48483876768932

H	5.30016134179138	14.49832590505576	1.54354184378786
H	6.87097702800500	15.20870516957906	1.09991511459611
H	6.55192810726289	13.48709222350985	0.78661474849555
C	2.53213615465606	8.48817905931901	-1.64566032368917
H	3.08882197861811	7.55757787578050	-1.82032278340613
H	1.48133508850268	8.21105423150640	-1.47936791832319
H	2.58811606645021	9.10589120671600	-2.54715530551164
C	6.74946386611502	15.24744844570100	3.72743091130721
H	7.26404858194474	15.20479238669408	4.69234162241480
H	7.14708679824549	16.11975855454490	3.19669000664068
H	5.67927263785158	15.40992443815177	3.90548404509833
H	7.99516380212452	12.94639014184089	5.18935778310857

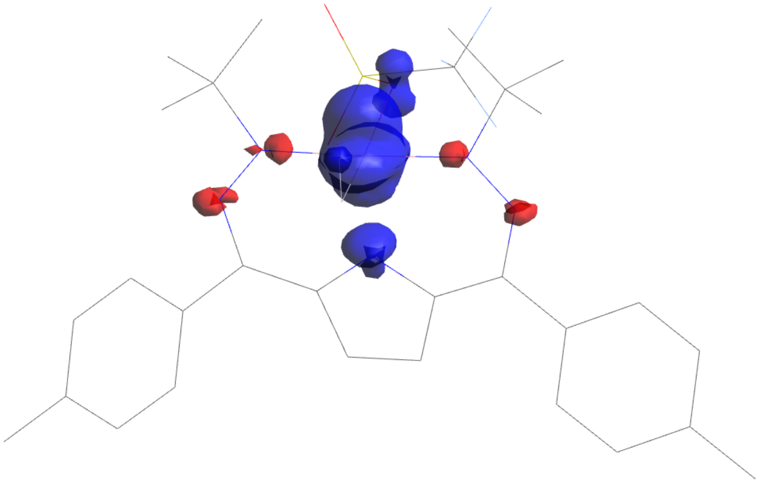

Figure S73. Spin density plot of **2-H₂** at an iso value of 0.005.

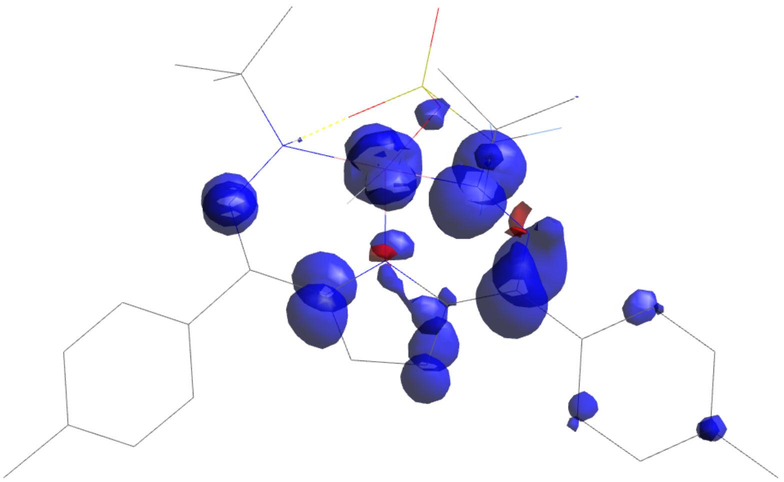

Figure S74. Spin density plot of **2H-H** at an iso value of 0.005.

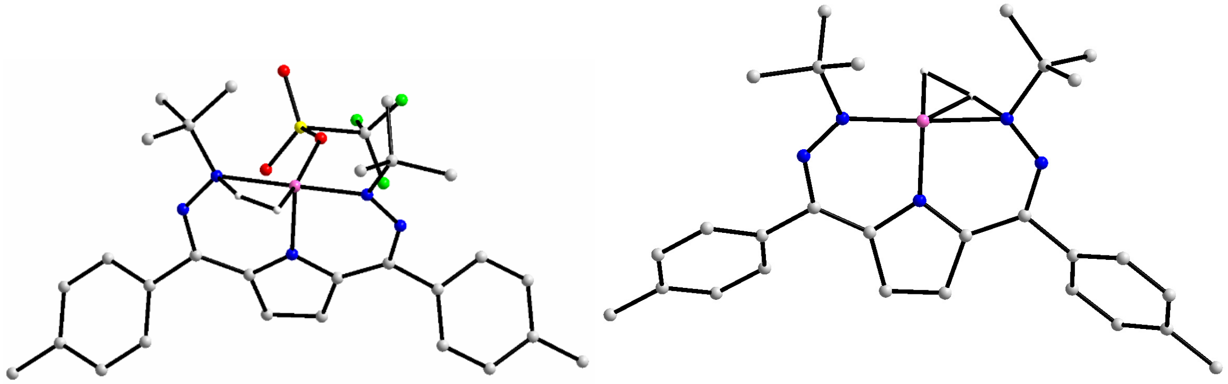

Figure S75. Transition state structure between low spin $[(^{tBu,\,Tol}\mathrm{DHP})\mathrm{CoH_2}]\mathrm{OTf}$ (**$\mathbf{2\text{-}H_2}$**) and $[(^{tBu,\,Tol}\mathrm{DHP\ \text{-}H})\mathrm{CoH}]\mathrm{OTf}$ (**2H-H**) and the triflate-free transition state. All C–H hydrogen atoms have been removed for clarity.

Table S47. Calculated coordinates of transition state structures between low spin $[(^{tBu,\,Tol}\mathrm{DHP})\mathrm{CoH_2}]\mathrm{OTf}$ (**$\mathbf{2\text{-}H_2}$**) and $[(^{tBu,\,Tol}\mathrm{DHP\ \text{-}H})\mathrm{CoH}]\mathrm{OTf}$ (**2H-H**).

Imaginary Frequency: -1136.61 cm^{-1}

Co	6.53784538366069	12.55027186130617	5.35085053179544
N	5.10617398521347	11.24878740522287	5.50207080754712
H	7.63907654513321	11.56101915822341	5.70334734100341
N	6.52492788307215	12.82822261741846	7.31131716940399
N	5.77267030702735	12.21241152943991	8.18761296576423
C	4.42396905925831	10.94636069450131	6.60429159489238

C	3.16830025689227	10.32516878503266	6.24001797415211
H	2.39523781720456	10.00167971660947	6.93416952716243
C	4.85485112185865	11.31689573325169	7.91477688360876
C	4.16752753868218	10.76052152908902	9.10187714388309
C	3.87680625557799	9.39377445769092	9.19931670412843
H	4.16448000379497	8.71920886333741	8.38791240971975
C	3.81384714447519	11.58568086405703	10.17541432761272
H	4.02441373731126	12.65630530092433	10.11506887944092
C	3.18328171773301	11.06277654650346	11.29738064889190
H	2.90457287842150	11.73397657508591	12.11593305482498
C	3.24885505165606	8.87651117306793	10.32620067343080
H	3.03923045348063	7.80307310476674	10.37625569383646
C	2.88725240074848	9.69909338007115	11.39866277883473
C	7.39762256826411	13.87002777782448	7.96058511001955
C	8.14601496923941	14.61610447434775	6.86804486316480
H	8.79956375118462	13.94719165487338	6.28315131365471
H	8.79532360646631	15.37615813396186	7.33095461466431
H	7.44999243597673	15.13319667071955	6.19043053924201
C	8.37731994960322	13.17755431890924	8.90154176292670
H	7.83588926424842	12.59844292536822	9.66646238240467
H	9.00712748952891	13.92652372800806	9.40996250970409
H	9.04456975186148	12.49169944339244	8.35009681142624
C	2.22432933293490	9.13145727612009	12.61542486526794
H	1.39120125902896	8.45964325383832	12.34598626186047
H	1.82718074825257	9.92533018190878	13.26801476480773
H	2.93546098294385	8.53326544564704	13.21453111675073
C	6.50323433817453	14.84300348775633	8.71894386709624
H	5.75867674557018	15.29936756616380	8.04777882714504

H	7.12461359408329	15.64759704941494	9.14577259889123
H	5.97556301023184	14.33766425648036	9.54169006009020
N	6.78741739281267	12.23462238354263	3.53969378002417
N	5.96769754676399	11.69513864845924	2.75679690150947
C	4.37126632949802	10.87060662758948	4.42133889130737
C	3.13461833280259	10.27929240291918	4.88150860266039
H	2.31930353128647	9.93288974537654	4.25023059002376
C	4.79138369036201	11.10834645615964	3.11611556532070
C	3.99120629458137	10.69045117839798	1.95086381430919
C	3.44901228418552	9.40396705435267	1.83551668639997
H	3.63775244319506	8.66377932242792	2.61738481453616
C	3.78120432667211	11.59125037293605	0.89794485723128
H	4.19364431864071	12.60109051309242	0.96846932665746
C	3.03509570616849	11.22465894819216	-0.21334637523178
H	2.87661336711651	11.95253706849556	-1.01537420667727
C	2.71198347529175	9.04115432342522	0.71443449907395
H	2.31475745068510	8.02355784253811	0.64320113447918
C	2.48125765140513	9.94390413379249	-0.32865405757969
C	8.04162783557813	12.69930003271376	2.84383538965385
C	9.23864625341280	12.04491312867854	3.52749507887955
H	9.16138521923166	10.94549398995552	3.49506570108284
H	10.15858164746632	12.34218596398883	2.99799023598785
H	9.34271195476252	12.35510310659819	4.57857601427710
C	8.03787560278751	12.32114858936630	1.37025410289569
H	7.19738252882632	12.78217333839153	0.83169023214454
H	8.97871930649937	12.67875667088342	0.92040690716888
H	7.97550057360685	11.23181375250646	1.22781249759041
C	1.65314432788765	9.56132998369345	-1.51510857017247

H	1.75302363739406	8.48931666022979	-1.75235619015828
H	0.58032706566950	9.75133483829890	-1.32361742375364
H	1.93057522221917	10.14315511662939	-2.40902013353215
C	8.09979352613040	14.21942920781219	2.98257343916502
H	8.15210399997920	14.53616955987418	4.03426484938320
H	9.00007292525184	14.59464864900048	2.46836456621214
H	7.21260506273548	14.69103852886183	2.53204508336936
H	7.28212192409163	12.05281540043902	6.69586725666958
O	4.55374987734518	16.20265121150819	5.83634843472229
S	4.25752690755031	14.83510730054640	5.41475568003980
O	5.46482604896020	14.14680994108003	4.81421727331672
O	3.46911191221084	13.99560207731798	6.31311063500153
F	3.88297456853253	15.65859800531152	2.94095004839639
C	3.21172367871360	15.02648033207499	3.89247789626712
F	2.12323611683034	15.71923648647327	4.18032404547323
F	2.84809479604991	13.83617416570230	3.43019274681047

Table S48. Calculated coordinates of transition state structures between low spin [(^{tBu,Tol}DHP)CoH$_2$]$^+$ (**2-H$_2$$^+$**) and [(^{tBu,Tol}DHP -H)CoH]$^+$ (**2H-H$^+$**)

Imaginary Frequency: -1292.76 cm^{-1}

Co	6.59045483906453	12.21096210517983	5.18787658658933
N	4.88074811105477	11.44455919477654	5.27459484698134
H	7.88443848416072	11.41621596273238	5.14176720846254
N	6.79983441454889	12.24332275485652	7.07553181561063
N	5.92535791363581	11.81522380960302	7.94858861277900
C	4.21210404851222	11.10840309956018	6.39239658641758
C	2.91296763543242	10.62745982879044	6.03958517003853
H	2.13690010680784	10.34954829301912	6.73508542130855

C	4.76258321030488	11.27245727384081	7.68687793061595
C	3.99303844993915	10.88096465927542	8.88313291788268
C	3.38320289606879	9.63733903836550	8.98472720417908
H	3.47193152013735	8.92144405562107	8.17303716007986
C	3.89904337595444	11.75532500847840	9.96258975217571
H	4.38421729613732	12.72473578655000	9.90407224166451
C	3.19453202460963	11.40247821531740	11.09278629759956
H	3.12246866115690	12.10669691729134	11.91804476680245
C	2.68594547460981	9.28698686100681	10.12634150084470
H	2.22731077173227	8.30375217016728	10.18777596983533
C	2.57082846199033	10.16182906866047	11.19761613657769
C	7.97902896004268	12.87351478173301	7.80570152550013
C	9.10036819102646	13.19432025654944	6.84217971105250
H	9.54510298253787	12.29342131796909	6.40446194482629
H	9.88320232570649	13.71165070397627	7.40705643708003
H	8.77803868662138	13.86556908243015	6.04104530100359
C	8.48694597424212	11.90681913529858	8.86093547243432
H	7.72231037303090	11.71190049549323	9.61710795081830
H	9.36561044874538	12.34677239272610	9.34598833030639
H	8.78791800443180	10.95609853910973	8.40162649990774
C	1.80979697769432	9.79325215341104	12.42469317493127
H	0.97235479357535	10.48720268199759	12.58195715784324
H	2.45332077831005	9.85357354552336	13.31308250044704
H	1.40980728561859	8.77720893725319	12.35153798167926
C	7.45642559885470	14.15327786710726	8.43448727719396
H	7.12409809072483	14.85647458653687	7.66105150758799
H	8.26254001479932	14.62194012934498	9.01054241158679
H	6.62206514459770	13.93659286396033	9.10847778872903

N	6.44170306371724	12.50466958601239	3.38102313063362
N	5.58112044737343	12.07346160313705	2.57582345428351
C	4.09915543353237	11.12155515057007	4.20784424461871
C	2.84386017908196	10.63373498519053	4.68573640366390
H	2.00013767942932	10.36279922330620	4.06983899558594
C	4.54099833538382	11.27513017391416	2.89887683789196
C	3.87950642872772	10.66167925365039	1.75705179812874
C	3.30006247924385	9.39653404658032	1.85478105725333
H	3.36247616282905	8.84697274948098	2.78806973898930
C	3.84668910037686	11.31125949335788	0.51890177180733
H	4.30390133455549	12.28959237891697	0.42307950751327
C	3.22641890562456	10.72746938486338	-0.56024860472401
H	3.19574647812420	11.25675230053249	-1.50921918554575
C	2.68707677619539	8.81747631372989	0.76399488844012
H	2.25477913758396	7.82536714728110	0.86018385372170
C	2.63051182084368	9.47058672354527	-0.46179251476884
C	7.38087392850756	13.51102512798897	2.76631686491935
C	8.78005453781740	12.91574004992783	2.77613837999635
H	8.79999302701807	11.98240309904807	2.20107362237467
H	9.46456269120467	13.63122657787938	2.30625379285785
H	9.13539961721511	12.71514299116628	3.79104604204333
C	6.98009342722311	13.85165181693338	1.34736155340535
H	5.95891185706016	14.24440317251146	1.30412578062317
H	7.66808100243372	14.62385798818597	0.98490133220534
H	7.04993261547965	12.98002761868978	0.68950223535642
C	1.96673801084359	8.85110805194813	-1.64015233682027
H	1.56610177204392	7.86250777813749	-1.39676124013007
H	1.14283855918672	9.48683584311852	-1.99398877058800

H	2.67613031911188	8.74872225723914	-2.47322308984433
C	7.29459525573402	14.75807324535767	3.63389508163484
H	7.52314743546125	14.54204127989364	4.68435907211184
H	8.02372682875301	15.49531486929408	3.27919017601746
H	6.29113964540313	15.19733284878646	3.57957890195202
H	7.40804338045607	11.41318129629902	6.26992612501266

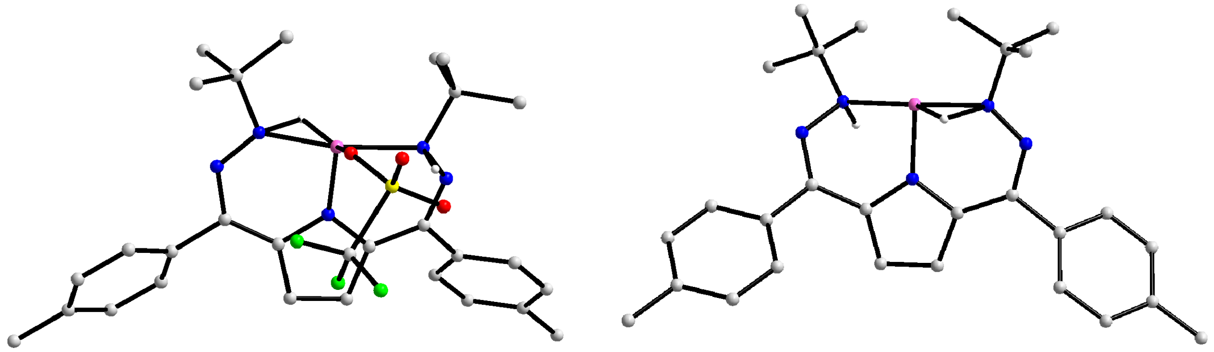

Figure S76. Transition state structure between low spin [($^{tBu, Tol}$DHP -H)CoH]OTf (**2H-H**) and **3** and triflate-free version. All C–H hydrogen atoms have been removed for clarity.

Table S49. Calculated coordinates of transition state structure between low spin [($^{tBu, Tol}$DHP -H)CoH]OTf (**2H-H**) and **3**.

Imaginary Frequency: -1214.45 cm^{-1}

Co	6.30973685763506	13.01706418080616	5.15375610113105
O	5.93091627338514	15.04589521099561	4.94826312913044
N	4.81602814430359	11.94623204602917	5.24347440257560
N	6.35625853849440	13.19959338220599	7.18180703787229
N	5.75415394663601	12.26346053608050	8.02650409067722
C	4.12337329250126	11.60306117994691	6.34469100525616
C	2.89021984989409	11.03308141918563	5.95802224699298
H	2.11252466758300	10.71416207371624	6.63474704245358
C	4.67820856351121	11.66152805419087	7.66623021617142
C	4.04357468085560	10.87230082684068	8.74535431546117

C	3.66436769455733	9.54651339388138	8.57863217323826
H	3.81175724090833	9.05987119459900	7.61824436954584
C	3.86362238647665	11.45597235759046	9.99588765217980
H	4.16297042120178	12.49043258369408	10.13982709626703
C	3.31008849336990	10.74112672908420	11.03668287598337
H	3.16914833664457	11.22485590664321	12.00175001703357
C	3.12134586307342	8.83155469356168	9.62993887512278
H	2.84647803400878	7.78942982138930	9.47870757256342
C	2.92774311396811	9.41286492936767	10.87603419973517
C	7.67258844884620	13.57544535210311	7.83699467747041
C	8.23881369011171	14.81057007463519	7.16732509736003
H	8.39850646453899	14.67550504519122	6.09851704949530
H	9.21089870865240	15.02885102869927	7.62367115985930
H	7.58963370508328	15.67997520735893	7.31809225574255
C	8.61618822678905	12.39819807571621	7.70984046918084
H	8.16629660820509	11.50274658277655	8.15293128129057
H	9.54851930758890	12.62153091553790	8.24108718433507
H	8.85525015674413	12.19072183897339	6.66080881280501
C	2.32821611459393	8.64852935811646	12.00634727100072
H	2.97907539522170	8.68159304105439	12.89134336894053
H	2.16608009849137	7.60016981172415	11.73291866815939
H	1.36025846019719	9.08090362179658	12.29925230089825
C	7.43886413399578	13.90835630323726	9.30490223590788
H	6.64548026737927	14.65968518946301	9.40758654792173
H	8.36487988701682	14.33049943068310	9.71283109048080
H	7.16706330909518	13.02228531996556	9.88184349582512
C	3.51967733060528	15.99437923744667	4.68318566907819
S	5.09897509727566	16.04669848399828	5.64196283068155

O	4.72307069242992	15.62054370864551	6.97290784649280
O	5.57172499294538	17.38904623314899	5.48826370774340
N	6.62329646578814	12.57691870768708	3.40036477060917
N	5.85275386793191	11.96865818246268	2.54617568526173
C	4.10781174896701	11.51988656744539	4.15411920320853
C	2.86978859503850	10.99888816244450	4.59147543774748
H	2.08452262278560	10.62088073499940	3.95765818762612
C	4.65223073643050	11.52425056867566	2.85787369722341
C	3.89515609311679	10.99748924694291	1.70908196225872
C	3.26599483685731	9.75886961445115	1.73051099745762
H	3.33115862084810	9.13690060477652	2.61858350748151
C	3.83202866289405	11.74512749866725	0.53475248582667
H	4.33051874222403	12.71007248610249	0.49784594103264
C	3.13895775456771	11.28155682604913	-0.56196115999156
H	3.09048249177176	11.89344016730380	-1.46102030680747
C	2.58118030297362	9.29536406982404	0.62237827202737
H	2.10642073197215	8.31695812880275	0.66206086488275
C	2.49572917388503	10.04689387288825	-0.54130854918128
C	7.89336631194336	13.01474710025396	2.72860031315437
C	8.74365049299588	13.76095518740995	3.72810494895806
H	8.97634460346174	13.13729167401552	4.60059244865838
H	9.69618633489606	14.02445344185150	3.25662213117245
H	8.25894569890453	14.69125210484404	4.04047084829507
C	8.62913698779511	11.78672006243871	2.22397251049517
H	8.00849885566546	11.23011950624826	1.51523115911457
H	9.55015073591869	12.10371685261891	1.72066095909444
H	8.89835025754261	11.12906605903370	3.06108195489763
C	1.74686524399364	9.55619619651459	-1.73248722166670

H	0.93437269199882	10.24890294610812	-1.99519496189519
H	2.40732009690180	9.48462545371138	-2.60864296897594
H	1.31148470689596	8.56839131714470	-1.54642354361802
C	7.52308599145595	13.94559558536700	1.58485139230634
H	6.93626231813141	14.79241929302935	1.95979596408139
H	8.44480410408579	14.33013349047057	1.13256016383149
H	6.94616704820107	13.41395986338084	0.82361702163546
F	2.66048806146887	16.87821812743942	5.17424158739964
F	2.96658808226649	14.78769522610859	4.74863832510175
F	3.74230214806917	16.28471377224036	3.40578658025033
H	5.75452963316716	14.05004692447165	7.23447035489711
H	7.09570065136837	11.79359999568591	4.61658159608378

Table S50. Calculated coordinates of transition state structure between low spin [($^{tBu, Tol}$DHP - H)CoH]$^{+}$ (**2H-H^{+}**) and **3^{+}**.

Imaginary Frequency: -966.95 cm^{-1}

Co	6.70917635908639	12.24924034407965	5.26265982975565
N	4.95967719836499	11.78567597606461	5.30968798386277
N	6.34123161530812	13.18449789314699	7.01642094035371
N	5.95130549607396	12.24112737681157	7.98972421884177
C	4.23763862511463	11.58395408346419	6.43270612547413
C	2.90632983211694	11.32784020124935	6.07077370106661
H	2.09215849008852	11.16150856153365	6.76006912859189
C	4.88985728056552	11.55876221925318	7.72903703985640
C	4.38540719897955	10.67924815152608	8.78903219914501
C	3.74787139535471	9.47779993586030	8.49768578374473
H	3.60423185041874	9.17729173716727	7.46514306060702
C	4.57261676236721	11.02183342533093	10.12814135437528

H	5.06544066661920	11.95795865536244	10.36845078586203
C	4.13194629736517	10.19121540418830	11.13321442598477
H	4.27876688963646	10.48261101540265	12.17030240971524
C	3.31474638994064	8.64720722537224	9.51264244115394
H	2.82911739858170	7.70827920495993	9.26107414990145
C	3.49288660022622	8.98633866740214	10.84715072978177
C	7.23259765949943	14.22638954483807	7.64266268128439
C	7.39140366119993	15.34064623994186	6.62714773669355
H	7.87634544342945	14.99949955385127	5.70850638846905
H	8.01372563250302	16.13159406295368	7.05922651782865
H	6.41750524132441	15.77873477149136	6.36958225808489
C	8.55826978460542	13.60288924721403	8.01819961488615
H	8.40051519800143	12.74585596213742	8.68146690421581
H	9.16604665653052	14.34584536273733	8.54639506715576
H	9.11799865061666	13.27570971851927	7.13584846167526
C	3.01436073033029	8.09949656446820	11.94365157365485
H	2.25883175989638	8.61454900105610	12.55342363157531
H	3.84274694914000	7.82992333507008	12.61311899010184
H	2.57230122969169	7.18118628764955	11.54527293236117
C	6.53944116565510	14.78181452521460	8.87721576638051
H	5.54603039903115	15.17503658172240	8.62223242689998
H	7.14099645045444	15.60491304195540	9.27855567687055
H	6.43042754126247	14.01484146413698	9.64864867497530
N	6.80099218135826	12.01979584324426	3.50380943331261
N	5.92364658849173	11.76180634669569	2.58783235434622
C	4.15086013009133	11.58931980110538	4.22395660079502
C	2.84440336227510	11.35292290948456	4.69755491807828
H	1.96847454123504	11.20777553571159	4.08673078831263

C	4.65169243900535	11.56964928289348	2.89916527374012
C	3.78289226972707	11.30629720555001	1.74855924916223
C	2.83893241130163	10.28401401811602	1.75163484960323
H	2.73545414147682	9.64512384415909	2.62167817601661
C	3.93352368526595	12.06432397354673	0.58691648391614
H	4.67567072649449	12.85587943177036	0.56431010413810
C	3.14307280143096	11.82658608606099	-0.51453617348170
H	3.26519914956935	12.44178552780972	-1.40244442993307
C	2.06154757987381	10.04267265510223	0.63623838005860
H	1.34362237029544	9.22727394636078	0.65570619267832
C	2.18964042735852	10.81038648428468	-0.51374772632526
C	8.20969886093824	12.16806606931687	3.07465249201942
C	8.87299246674909	12.68715688552938	4.34222989049276
H	8.55805728084506	12.10246391109646	5.26050851078293
H	9.95276448759371	12.50066533323345	4.34508080878418
H	8.71120873776562	13.75912332305715	4.47314056557295
C	8.76430385053319	10.82420629663767	2.64727625543393
H	8.17482408210308	10.42599449871489	1.81451343874091
H	9.80332053172586	10.94902864324527	2.32122890020214
H	8.74245529560027	10.10850868182667	3.47972079919316
C	1.34336608180665	10.55901257594756	-1.71287667463172
H	0.70397722971991	11.42870106746312	-1.92086800305443
H	1.96844133025510	10.39652021738251	-2.60147302684713
H	0.70257380759390	9.68380889047993	-1.56873190929344
C	8.30473998634924	13.18843272814507	1.96002566646026
H	7.80767925860735	14.12286215079226	2.24517282862670
H	9.36352220233891	13.39589607195457	1.76523659058300
H	7.84529646343824	12.79900823766383	1.04699230603279

H	5.50155223765514	13.69467483207054	6.71814577728505
H	6.93656050374716	10.95007135040802	4.55010869800833

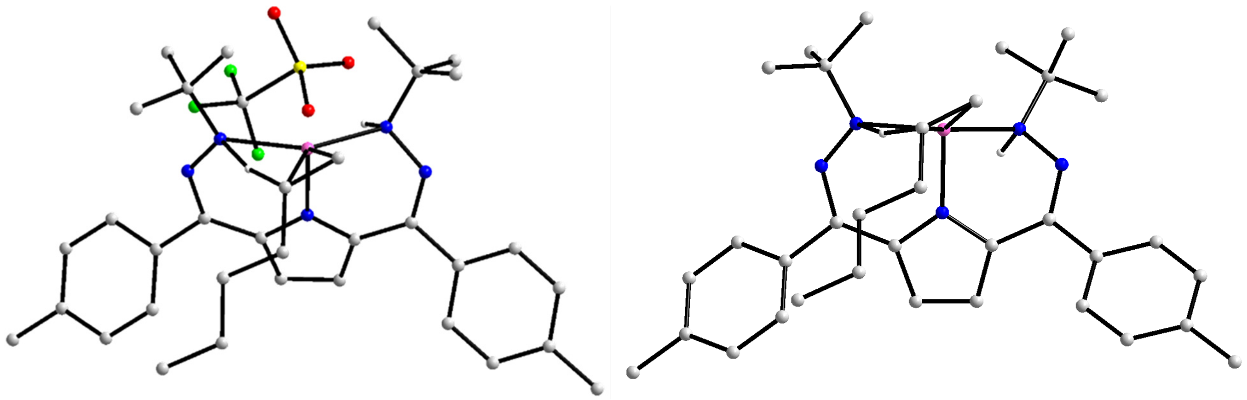

Figure S77. Transition state structure between high spin [($^{t\text{Bu, Tol}}$DHP -H2)Co](1-hexene)OTf (**3-hexene**) and low spin [($^{t\text{Bu, Tol}}$DHP -H)Co](hex)OTf (**4-hexyl-β**) (and triflate free version). All C–H hydrogen atoms have been removed for clarity.

Table S51. Calculated coordinates of transition state structure between high spin [($^{t\text{Bu, Tol}}$DHP -H_2)Co](1-hexene)OTf (**3-hexene**) and [($^{t\text{Bu, Tol}}$DHP -H)Co](hex)OTf (**4-hexyl-β**).

Imaginary Frequency: -1290.78 cm^{-1}

Co	7.02131373853181	11.66174792210106	5.31406973712270
N	5.12428406663871	11.16195813782804	5.36699226692566
N	7.00010789231208	11.91522944992219	7.37730028388188
N	5.99049727160026	11.72132735736430	8.16034192911668
C	4.32654360650400	11.07379971655358	6.46715870723369
C	2.96942755420305	10.91660522620864	6.04711073198623
H	2.09944405066944	10.87800001776963	6.69917392726653
C	4.79901339639849	11.25828671459504	7.78875049843873
C	3.90684925211629	10.99387879284912	8.93554649199087
C	3.11558061749878	9.83758498906224	8.99640610894876
H	3.15675980935731	9.10998983936997	8.18160730205874
C	3.86278110072986	11.87873028573545	10.02181534739820
H	4.46482994280218	12.78910450500678	9.99128426612945
C	3.04352170344886	11.62397366563705	11.11275101746223

H	3.01835626785463	12.33983307316320	11.94055458144990
C	2.30706285257627	9.58450369111424	10.09720678754035
H	1.71359523596325	8.66491340357016	10.12396951236497
C	2.24712514277301	10.47434189254740	11.17558728383974
C	8.16101861306029	12.50262344871698	8.13102599795455
C	9.32026001293948	12.69765647969625	7.16962356171025
H	9.72796438576687	11.74685802208306	6.79220866470558
H	10.14066821284371	13.21387751108320	7.69263693871637
H	9.02974711745217	13.33257343104462	6.31960705052544
C	8.56606392084433	11.56736054997951	9.26625473576857
H	7.72934381743856	11.42015742816231	9.96593474132867
H	9.41315123078011	12.00478864220591	9.82001931773954
H	8.88146879712238	10.58081394044285	8.88502612606330
C	1.34748350181656	10.21276164553071	12.34225931654844
H	1.64962508462635	10.79495949909769	13.22759640458730
H	1.33800060628069	9.14412002969268	12.61545216204804
H	0.30379985243261	10.49244281840741	12.10565809225551
C	7.71961777241408	13.85693203651955	8.67914048144178
H	7.36780504799513	14.52495223345305	7.87635296723591
H	8.57207349105609	14.33693276629264	9.18816564353856
H	6.90404345181987	13.73600255425180	9.40787504818403
N	6.57671709569990	12.66836772355920	3.54642706973623
N	5.91560207832201	11.88274801573909	2.61525723380487
C	4.33886740325330	11.06390022295294	4.28378717981486
C	2.98111681162673	10.89861670673550	4.67249585778321
H	2.12811276001029	10.82685725007334	3.99996179276456
C	4.88287056269420	11.18153717439944	2.93909674265262
C	4.22129363564464	10.45874847703133	1.82987701190394

C	3.62003230932005	9.21112889781293	2.02893430599166
H	3.62812685011743	8.75568816975914	3.02278126920258
C	4.20465484360538	10.99824280929081	0.53596063951188
H	4.65892365726068	11.97719456020994	0.36438966111657
C	3.60744222866961	10.31469012803675	-0.51224376321468
H	3.59884714418816	10.76629338287863	-1.50967959146507
C	3.02797430440656	8.52776377727187	0.97132098925843
H	2.57211533208789	7.54959725230814	1.15455400967358
C	3.00556757653260	9.06380537263678	-0.31848722047227
C	7.48816645829669	13.62665799253274	2.81691580162976
C	8.31868651503601	14.37209547006492	3.84871360263926
H	9.04487252533313	13.69963905474371	4.33252140560607
H	8.89505388878903	15.16618447903275	3.34700317775242
H	7.69565570678455	14.84724912134986	4.62137121883211
C	8.40272766132514	12.87159165794204	1.86503954457559
H	7.83026853748368	12.33823095988639	1.09209108760883
H	9.07244424406607	13.59162700149451	1.36646379236463
H	9.03165458018507	12.14290882463117	2.40032755063589
C	2.36350980497182	8.33785661027680	-1.45935008973634
H	1.57818052140288	8.95277278041010	-1.93360801800271
H	3.10068448834503	8.10164549452137	-2.24776082341290
H	1.90319402331773	7.39194824505078	-1.13256713593576
C	6.61288160067836	14.61225242189971	2.04437393452367
H	5.96562288001909	15.19222465677618	2.72361948552054
H	7.24778471522005	15.32224102818266	1.48898969913800
H	5.97484578763668	14.08193376196168	1.31900144934797
H	7.37594977099553	10.77358206234521	6.63980447020150
H	5.90867930781027	13.25617297815270	4.10125083269518

H	6.51268013609451	6.06154186897203	9.11645915333848
H	7.69173579850680	7.90775348314063	7.73343411839853
H	7.88539493479658	10.06248408295514	3.56008780211500
C	8.33597701764506	10.41718242078048	4.49481185115111
C	5.61976665522052	6.22996626171484	8.48828865005498
H	6.60756527672500	5.95226519322975	6.59059533805505
H	9.29179129074675	10.94704955643130	4.39744148071508
C	6.77714573329840	8.03250224046693	7.12209711847110
H	7.75236667704947	7.72874594197703	5.23215181721392
H	5.06401357533686	5.27807027062680	8.43694723105450
C	6.00391615201384	6.72339134586938	7.10551089679214
C	8.01561997178627	9.76918867697828	5.71267084319323
C	7.16492105558131	8.52034240284508	5.73616555788730
H	4.98060881779371	6.96289107591007	9.01224774435812
H	5.09257923805555	6.85077386055642	6.49131520852170
H	6.17215315470477	8.80211302120010	7.63464169972098
H	6.26462207446895	8.67253185127121	5.11746616952848
H	8.82986640400678	9.76205249643645	6.45486944871343
O	4.33744823164815	16.21222686511543	4.69250795120197
O	6.19609802272279	16.21336956197719	6.36062966864969
S	5.04942021956277	15.51404044410946	5.76525976047145
O	5.30378042040244	14.06817033085817	5.48730085750384
C	3.81473355341122	15.40540020873785	7.14784813839861
F	3.39634902424165	16.61191678825934	7.49736957456027
F	2.76492394158560	14.68212630603137	6.78377868842245
F	4.36554659278716	14.82900720453378	8.21473502595150

Table S52. Calculated coordinates of transition state structure between high spin [($^{tBu, Tol}$DHP -H_2)Co](1-hexene)$^+$ (**3-hexene$^+$**) and [($^{tBu, Tol}$DHP -H)Co](hex)$^+$ (**4-hexyl-β$^+$**).

Imaginary Frequency: -1274.71 cm^{-1}

Co	6.95706727344555	11.61709393725638	5.26997500713851
N	5.03972842216106	11.26954932562482	5.35382615978028
N	6.96555439466797	11.95158222436892	7.26119364511347
N	5.97012486775359	11.81887459144364	8.07970954682073
C	4.23972714493516	11.22167014683301	6.44955775330502
C	2.89363083853851	11.09670697649497	6.02354977614193
H	2.02345974469445	11.08813025770509	6.66131767218140
C	4.74988337063911	11.39744406310003	7.75767926444311
C	3.89994574360904	11.15412892785868	8.92543722918691
C	3.01895294565455	10.07544739765590	8.96544569426820
H	2.96403206817264	9.39586109550024	8.12142536475569
C	3.99133504675119	11.97270352322249	10.05377240338740
H	4.67946430915357	12.81110146602598	10.04180371749435
C	3.20982647144923	11.73335632839297	11.16013727762008
H	3.28400349657721	12.39463543874551	12.01986253751006
C	2.24977036847103	9.83418564849791	10.08565247997644
H	1.58323773314747	8.97618128394496	10.09943059968726
C	2.32287299731674	10.65829751678846	11.20149949831365
C	8.17243424318877	12.51080063116060	7.95130212588425
C	9.33112977515127	12.53534245062027	6.97569266195957
H	9.71498238062043	11.53209655230393	6.76557179045111
H	10.15056992952482	13.11474946049237	7.41369707814784
H	9.05766381567285	13.02371234532183	6.03175931022756
C	8.52780594218001	11.65561088395093	9.15594565465318
H	7.71787387162614	11.66913175701317	9.89054988813540

H	9.43786636547154	12.05361006024627	9.61938451510720
H	8.71858267117686	10.61807992031076	8.85377967476327
C	1.49070201005426	10.40453464273125	12.40931756763253
H	0.88325837692062	9.50230837745858	12.28963669149634
H	0.82053892560826	11.25465514998248	12.60029623391834
H	2.12505826056247	10.28682281502453	13.29874957344472
C	7.82375299399917	13.92848631887165	8.37783882461378
H	7.58706138215337	14.54851599119089	7.50497975745333
H	8.68373922226615	14.36789316707021	8.89607788266573
H	6.96633454581416	13.92300216030162	9.05788236443550
N	6.39391133880427	12.79930731290919	3.66291259593793
N	5.90452358612371	11.93781324388290	2.67010967554007
C	4.26102608656394	11.16181073231615	4.26392915754642
C	2.91187971847387	11.04616267689713	4.65038266803766
H	2.06469815451011	10.96915445530674	3.98531394636255
C	4.86988873938969	11.21603724486962	2.94049592101807
C	4.32331752771274	10.40039724637601	1.85035338307195
C	3.65366318367375	9.20946338999241	2.11066634654906
H	3.52010326725163	8.87841644931562	3.13572325621029
C	4.48938408375074	10.78843968191829	0.52086774905827
H	5.00218005767481	11.71964884096224	0.30432367500473
C	4.00190139783760	10.00964328030487	-0.50369017759336
H	4.13417936118021	10.33582742572404	-1.53241727951334
C	3.17200120680171	8.43068653744501	1.07594592867971
H	2.66001627708339	7.49962387177523	1.30351961198871
C	3.33278503223232	8.81373959533282	-0.24846873147457
C	7.18625550208108	13.91747882276732	3.05119325228450
C	7.41428066660453	14.93381387947365	4.15153222212719

H	7.97035134919260	14.50283843692402	4.99028408716016
H	7.99809961030253	15.77042158174458	3.75293416640005
H	6.46082641733942	15.33091540793838	4.52572654904882
C	8.49602620352234	13.37870005958585	2.52039847573449
H	8.31839152038854	12.54482991602968	1.83300362142136
H	9.01965909896968	14.17340368149984	1.97764012128677
H	9.14323530957797	13.03992768799232	3.33699946047587
C	2.80557503214618	7.98297460150953	-1.36660787897740
H	2.33985000738970	7.06607544007285	-0.99254584455466
H	2.05734825707608	8.54335985210107	-1.94457529929959
H	3.61281558913323	7.70979827835519	-2.06009264795057
C	6.37468299813986	14.55440890367785	1.93469432539023
H	5.39486907883482	14.88408261781675	2.30549738022910
H	6.91215380658372	15.43148101518401	1.55747236960296
H	6.22299253742207	13.85154252707139	1.11061095814487
H	7.30411224527273	10.72052391592221	6.56337442032355
H	5.59247245516409	13.24561381027993	4.13768288721209
H	6.06150157781953	6.16221774631056	8.94571629874831
H	7.40722976802383	7.89396394111864	7.60429590992664
H	7.79385623207672	10.08810521969944	3.46909955737016
C	8.26612630495123	10.37227668635802	4.41095622626243
C	5.21022060514537	6.37404408877249	8.28417546116306
H	6.26830228074346	6.00069346985271	6.45222891026983
H	9.22671448290243	10.88022755248136	4.33555346563790
C	6.53247216989111	8.06597338222087	6.95634722780044
H	7.58311025271981	7.68080777154481	5.13306945408496
H	4.59911599806420	5.46586100270495	8.21612066679811
C	5.68624833955489	6.81169856547134	6.91736009355082

C	7.91870712117171	9.69520623389497	5.58830346062629
C	7.00602776169852	8.50396940894317	5.58763117520714
H	4.60371523275277	7.15884066912679	8.75639016168126
H	4.81762226835086	6.98866595557671	6.26353517414722
H	5.95014971622985	8.87399139930086	7.42166079868847
H	6.14859034089404	8.68801213229019	4.92731363200345
H	8.70590089367075	9.64933951953795	6.35251475143123

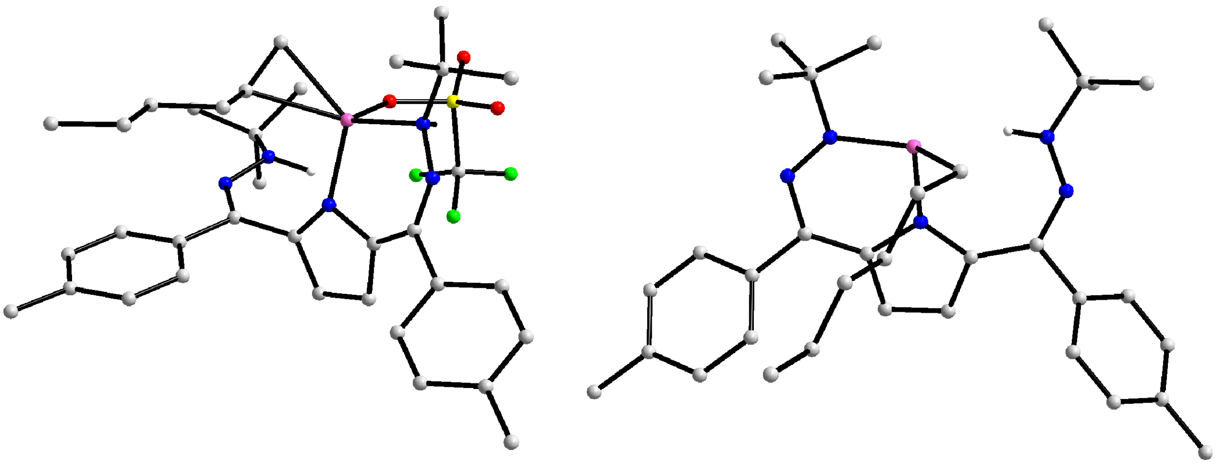

Figure S78. Transition state structure between low spin [(^{t}Bu, ^{Tol}DHP -H)Co](hex)OTf (**4-hexyl-β**) and [(^{t}Bu, ^{Tol}DHP)Co]OTf/hexanes (**2**) and triflate-free version. All C–H hydrogen atoms have been removed for clarity.

Table S53. Calculated coordinates of transition state structure between low spin [(^{t}Bu, ^{Tol}DHP -H)Co](hex) OTf (**4-hexyl-β**) and [(^{t}Bu, ^{Tol}DHP)Co]OTf/hexanes (**2**)

Imaginary Frequency: -131.33 cm^{-1}

Co	6.78569953999166	12.60967430518220	5.93119840835624
O	6.57876360172032	14.55441419462492	5.58340120846367
N	5.03693115387514	11.97123773649460	5.46423898496620
N	6.81069706290481	12.38067499337773	7.76555391801084
N	5.75634787646278	12.08859190486244	8.42311737783890
C	4.18051393455972	11.78970575940500	6.53253512069369
C	2.86600081944207	11.52632344346872	6.03216339755998

H	1.96799441223637	11.41160902270684	6.63457160767282
C	4.56906149945553	11.77043322877991	7.88992398915921
C	3.60045623017180	11.33616305404739	8.92326015073762
C	2.89556682740239	10.13034218396429	8.80879254976984
H	3.05932348406156	9.49063334328220	7.93686936477446
C	3.41412620966471	12.09823938015857	10.08339968174636
H	3.96287933433218	13.03648741674370	10.19863125819253
C	2.53372398094621	11.68292692314197	11.07409540019368
H	2.39795653997638	12.30504691288716	11.96451466699871
C	2.02447429863112	9.71573644151033	9.80887437588691
H	1.50012754661762	8.76090715063995	9.69937499336318
C	1.81691441694000	10.48655792410258	10.95794544587115
C	7.95301225250838	12.75918167092655	8.66470492450023
C	9.12711380545987	13.17098172903982	7.79090242702190
H	9.55304061972980	12.31704728067870	7.24334968162576
H	9.92655792812567	13.59164753152155	8.42109678178962
H	8.83743539635963	13.95525813530814	7.06981951497029
C	8.32384915769904	11.55562378318996	9.52690325420092
H	7.47068414229950	11.24935779485995	10.15190776216897
H	9.16343661000649	11.82521444985771	10.18894111794324
H	8.63674929228227	10.69655908092203	8.91215613187219
C	0.84956167254687	10.05194733315544	12.01451796823746
H	0.88108216958229	8.96024527013697	12.16895272318039
H	-0.18856190661744	10.30569500964865	11.72914172197698
H	1.05256171545334	10.54423277023437	12.97929994523150
C	7.53238899327175	13.92481595641078	9.55587237447334
H	7.25510842527533	14.80615485603712	8.95845269964911
H	8.37041349316489	14.19422124416879	10.21983746790402

H	6.67041897794684	13.64556637259781	10.18073364908306
C	4.42996325260874	15.95026285832568	5.15292816509132
S	6.00380105335332	15.81574852834386	6.11425881334011
O	5.57039024443878	15.72692276704320	7.47736676829615
O	6.77495516698444	16.95691998983199	5.71866056526409
N	6.51139542717921	13.18997209800738	2.94837542062229
N	5.87354724845932	12.17410578891347	2.42443302556447
C	4.33837407410102	11.71563110377090	4.33264691821849
C	2.97278421845356	11.45599004068537	4.67004927454147
H	2.17763039806582	11.26753883705885	3.95049291288513
C	4.88291132088427	11.54792585551074	2.99338511673316
C	4.27843822166343	10.48923109472084	2.14520514542444
C	3.85767322259604	9.26319975603331	2.67613178106739
H	3.95559764711252	9.07448858016492	3.74867635212445
C	4.15090075296316	10.67355964320604	0.76073424495699
H	4.46147171380572	11.62598074560572	0.32376380088864
C	3.63052394513965	9.67607779452381	-0.05161486833637
H	3.53613503192251	9.85717923847215	-1.12749165286590
C	3.33735776400286	8.26615932042441	1.85724234507424
H	3.02572250314778	7.31655874338708	2.30424526748623
C	3.21311639946955	8.44802094734777	0.47740154680606
C	7.44534261573744	13.89841174456052	2.04699368099543
C	8.18953668350671	14.94299903400285	2.86605015583166
H	8.75103996587182	14.47918679058667	3.69303892814044
H	8.90598261397442	15.47085692529793	2.21632724291087
H	7.50832502844706	15.69341724483406	3.29592239517612
C	8.43325223899490	12.89192968889363	1.46988149750888
H	7.91850653379766	12.12252063166019	0.87460588389179

H	9.15259235313811	13.41344726067697	0.81738279796411
H	8.99698762500821	12.39047303205738	2.27461447858468
C	2.67627912347260	7.36848042942657	-0.41015564622130
H	2.14864304620269	6.59250832758292	0.16728279884323
H	1.97825402840016	7.77230045647917	-1.16339938917632
H	3.49119575588036	6.86945149781675	-0.96716034481084
C	6.65401424590379	14.57358053031642	0.92692353309114
H	5.94031357422863	15.30851231085369	1.33627844870519
H	7.33560752166112	15.10316855116057	0.24016163437416
H	6.08997979942517	13.82525307821978	0.34589424727508
F	3.84280588954509	17.10318080109073	5.41690091543635
F	3.60486782230386	14.96429399735250	5.47338753177621
F	4.66914800822178	15.88509675285122	3.84359325550924
H	6.04303735113384	13.77297254839312	3.63927080894850
H	6.40238405112828	4.87618741618273	6.59024214146018
H	7.59862279867941	7.07379966514662	5.95610068523152
H	7.47864258835264	11.11165025447710	4.27344616724540
C	7.89958202926590	11.16701782266061	5.28703632527529
C	5.69859922528223	5.47861936423467	7.17216160982459
H	5.15384397783089	6.79090686851830	5.56128935154163
H	8.85890154989240	11.70827474151993	5.28935219103679
C	6.85743713133073	7.64037236959029	6.54382066554919
H	6.31158581064965	8.94775243373292	4.93454987236160
H	4.73552558070637	4.95230005953538	7.18604679442265
C	5.55683182926520	6.86469570785349	6.58407227210651
C	7.98925266223163	9.82544006035893	5.93525782329976
C	6.70324623379069	9.02728950160993	5.96006687406113
H	6.07478900948094	5.52828114794447	8.20373767205362

H	7.26054814986789	7.71869813227275	7.56722682414125
H	8.39145109057005	9.91579316050732	6.95270790696049
H	4.81966316251925	7.43787483149154	7.16857270048422
H	5.95262235944000	9.57904676321970	6.54040860962382
H	8.74305781401261	9.24950274555809	5.36131766928832

Table S54. Calculated coordinates of transition state structure between low spin [($^{t\text{Bu, Tol}}$DHP - H)Co](hex)$^+$ (**4-hexyl-β**$^+$) and [($^{t\text{Bu, Tol}}$DHP)Co]$^+$ /hexanes (**2**$^+$)

Imaginary Frequency: -132.34 cm^{-1}

Co	6.99530829663424	11.96563822227842	5.88037008762212
N	5.26713295866858	11.55726837588935	5.46615534260880
N	6.63766942726170	12.40535275883425	7.59653502596904
N	5.68447852176757	12.11402855533815	8.37107842257992
C	4.33844108325945	11.40602961198161	6.47334465266875
C	3.07574899320654	11.09052165416175	5.88754263482816
H	2.15075896045536	10.98322235140211	6.43154338323890
C	4.55795320979447	11.59114710190566	7.83991883412033
C	3.55926794010894	11.20616736857577	8.83862198250601
C	2.88066023182890	9.99269117006485	8.76312273067275
H	3.09360778466292	9.30755041064259	7.94846516140196
C	3.31176025842118	12.03287327172814	9.93539221118971
H	3.84349114263330	12.97518129792030	10.01947537353303
C	2.39593366474578	11.66976435575315	10.89648772474563
H	2.20736830114025	12.33679377181972	11.73397458241374
C	1.97158458624568	9.63114083224222	9.73783860955313
H	1.46597103011999	8.67206500114426	9.66476251106987
C	1.70581525810731	10.46146575784948	10.81873758118997
C	7.87008214669516	12.94096613705154	8.19706148295979
C	8.76349664059345	13.09326835335625	6.97280489143282

H	8.77506798439307	12.15405063258019	6.34665222846522
H	9.82223339252731	13.17620971812022	7.24412925896891
H	8.49584197919737	13.97040497869389	6.37424837218450
C	8.44392313466544	11.96072633268970	9.20234606298158
H	7.72573754891575	11.79444190442685	10.01141646241813
H	9.36618613507554	12.37569644988901	9.62526120134520
H	8.67584829170705	11.00147512901002	8.72591292303058
C	0.72463051913306	10.07765285463094	11.87105290057506
H	0.31217999033228	9.08148534309271	11.68377659254898
H	-0.10490101230834	10.79801490069455	11.90529520625753
H	1.19814145821545	10.08154386308295	12.86244537798388
C	7.59027130792572	14.28673945204140	8.83876684153465
H	7.11320447827621	14.96666835590161	8.12265813714660
H	8.53606775683163	14.72991076793022	9.17257812450148
H	6.93303547839113	14.15793456592455	9.70504949432086
N	6.97999497184032	12.76014430707860	2.99312416215117
N	5.98272724394464	12.23027915665467	2.42059958906503
C	4.63667479252109	11.30370695483531	4.28447573405348
C	3.25572932827555	11.02938841773246	4.55239703048243
H	2.50782052923579	10.85370809788205	3.79600002078264
C	5.07697276457974	11.42108507412143	2.93048354905163
C	4.28840023210549	10.73071173067292	1.89175888804412
C	3.95156157401875	9.39028019504993	2.04288117836113
H	4.25969384844354	8.85710228899038	2.93801212914101
C	3.90708327100257	11.38070804479669	0.71943798146453
H	4.17011992029112	12.42454367669514	0.58178869472230
C	3.19364976087350	10.71342117316275	-0.25099112166234
H	2.89104411337699	11.24408044794967	-1.15050059715678

C	3.24073634243749	8.72602011595598	1.06114291586128
H	2.99554423501672	7.67635791408933	1.19926812626673
C	2.84442661008334	9.37250973709130	-0.10150884662252
C	7.76604487300879	13.79598439750385	2.29440659416418
C	8.79505204860730	14.30521205271474	3.28105595507423
H	9.42091739785491	13.48519698549252	3.65697225538644
H	9.44532918625277	15.02622030956870	2.77588645083664
H	8.31252516059667	14.81062743600096	4.12637827700062
C	8.44366517998215	13.13897294696020	1.10000054052369
H	7.69181606118461	12.71914493890266	0.42315080494946
H	9.02731765816438	13.89224383236524	0.55907706493783
H	9.11831727016299	12.33943364615380	1.42910953853324
C	2.07520863513259	8.66399406104997	-1.16247851741663
H	1.86141729998337	7.63048066109197	-0.87286553443236
H	1.12344199396167	9.17692353263731	-1.35846493734025
H	2.63776367871292	8.65234262830443	-2.10652113898110
C	6.84046437649617	14.91271581059834	1.84855110459553
H	6.34298083836115	15.36993487721872	2.71250853891252
H	7.42883130489682	15.68065371434744	1.33418305154832
H	6.08102116883204	14.53216621303505	1.15730471139274
H	7.31702609985914	12.42440563686588	3.90544694723188
H	6.86422910034697	4.67079981285387	8.24526630118636
H	7.79792854583825	6.61882456949474	6.85838958148388
H	7.14802071549872	10.02992947521932	4.27319256816925
C	7.80732606761961	10.37271159699585	5.08333192681924
C	6.25385941851524	5.40348927656332	8.79121821115916
H	5.34234422195408	6.25822540146944	7.04574807384501
H	8.75377862534296	10.73481373203980	4.64331797299582

C	7.16282939671787	7.32314272336909	7.42029204695827
H	6.25337570238722	8.18425144169809	5.67851462175484
H	5.33935382282771	4.89959307140496	9.12817008604498
C	5.93167189531271	6.59304516348235	7.91436018714074
C	8.08032278317155	9.23920676135976	6.04346386424726
C	6.84814299956303	8.51700285134686	6.54519859213231
H	6.81829275985560	5.71547889280797	9.68097554182093
H	7.75563861841107	7.65654923543743	8.28818441192315
H	8.67057537753546	9.59330199443088	6.90261194261764
H	5.29511039515746	7.29872878064220	8.47141149624713
H	6.21525995018773	9.21755270952427	7.10653037525519
H	8.72366095402652	8.50511988763320	5.52412864670415

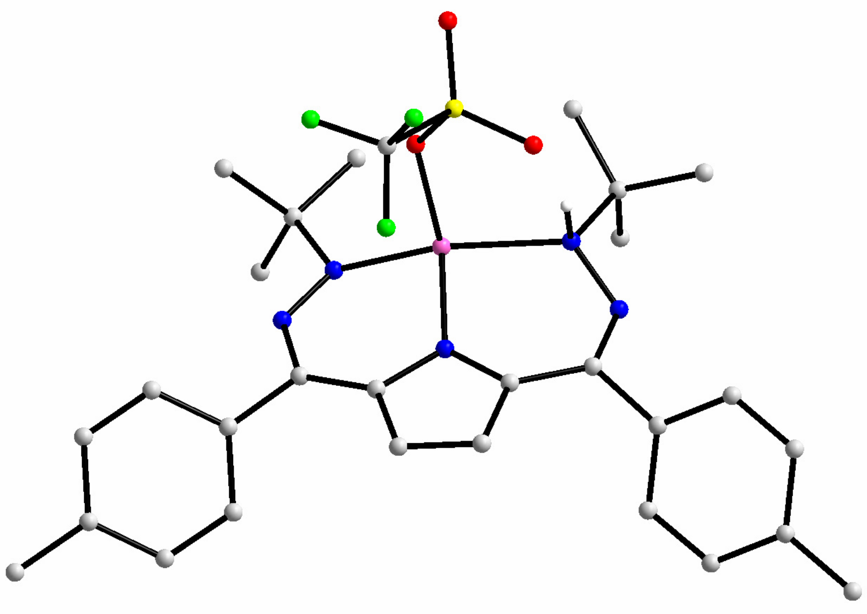

Figure S79. Calculated structure of high spin [($^{tBu, Tol}$DHP -H)Co]OTf (**4-HS**). All C–H hydrogen atoms have been removed for clarity.

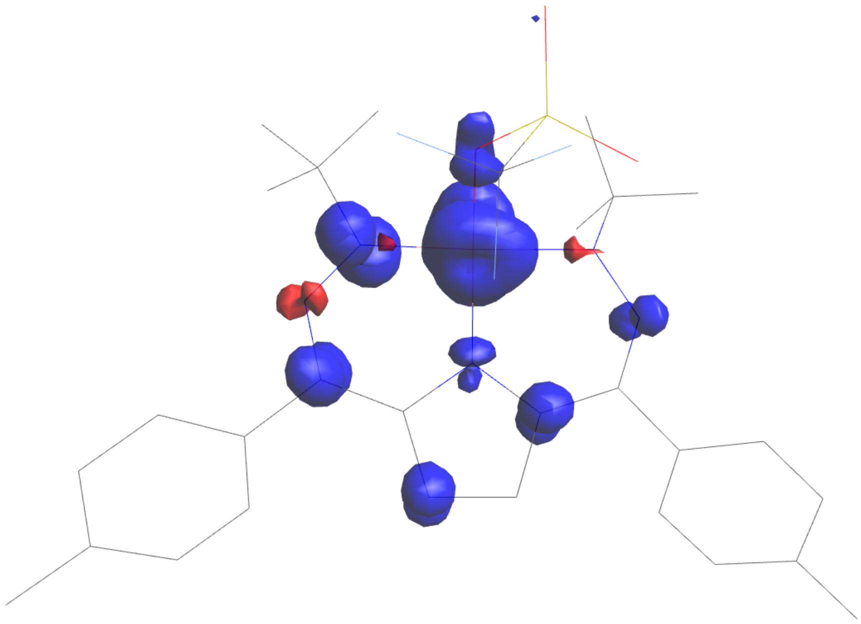

Figure S80. Spin density plot of **4-HS** at an iso value of 0.005.

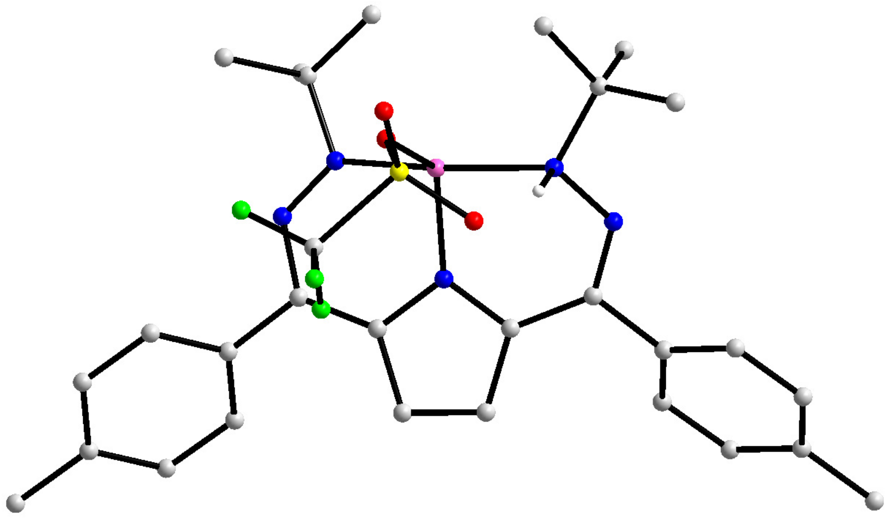

Figure S81. Calculated structure of low spin [($^{t\mathrm{Bu},\ \mathrm{Tol}}$DHP -H)Co]OTf (**4-LS**). All C–H hydrogen atoms have been removed for clarity.

Table S55. Calculated coordinates of high spin [($^{t\mathrm{Bu},\ \mathrm{Tol}}$DHP -H)Co]OTf (**4-HS**).

Co	6.29557073775491	12.87041139536100	5.16760718938753
O	6.14282860807697	14.82316151669285	4.81947143106197
N	4.68110671314602	11.83865009219276	5.28511198691994

N	6.41631403434599	12.90100910919156	7.27966496100316
N	5.66434425591159	12.04260273493970	8.07670403191556
C	3.93982289020520	11.60054470177494	6.37974723751192
C	2.64724860620074	11.16317305709814	5.98529816406642
H	1.82946932040147	10.92890604642781	6.64951259381140
C	4.50980761428981	11.59993738948842	7.70137034067013
C	3.77692046664184	10.89736834875331	8.78050569804958
C	3.24568691274850	9.62236133454295	8.61255447063802
H	3.34854516072362	9.11746325461448	7.65706865398211
C	3.64979789817854	11.50500805680138	10.02285932292506
H	4.06426265451896	12.49747620682210	10.16842377865442
C	3.00066717643376	10.86385715697173	11.06074103264998
H	2.90464086383152	11.36532776805800	12.02036155091031
C	2.60846185808803	8.98275057075519	9.65618475006694
H	2.21407405671787	7.98046751967992	9.50593924817790
C	2.46866644936596	9.59174855902056	10.89912539392562
C	7.73059828544865	13.15083753138859	7.97035479451909
C	8.44834578823462	14.25967711431374	7.23129519919498
H	8.63486417874583	13.99636847736324	6.18727110615242
H	9.41856869106357	14.43149620955613	7.71049741169789
H	7.88219169098316	15.19779058701805	7.26548262858933
C	8.53070094274137	11.86563827895140	7.91825436941743
H	7.98119183030366	11.05369481740169	8.40701121851687
H	9.48539143082501	12.00679237026107	8.43772597481444
H	8.73876296252220	11.58011078792586	6.87917138291004
C	1.76969335693567	8.89447274035992	12.01669570092096
H	2.24695289748561	7.92847267170293	12.23184004320757
H	0.72270646684068	8.68898693856414	11.75304728845275

H	1.78380894960015	9.50021726873701	12.92841667480127
C	7.49709538693201	13.58632175416996	9.40836303852161
H	6.85913794773400	14.47932748103289	9.43965518339922
H	8.46244107915714	13.83802651147119	9.86306294510758
H	7.02630797297377	12.79088486623646	9.99102455489390
C	3.82824909415693	15.99619224785584	4.52794250900935
S	5.37415730714704	15.87370761656427	5.53224731530839
O	4.93002782493810	15.43148324048238	6.83385295914578
O	5.98157234721693	17.16337454882897	5.43239073875737
N	6.63679566637973	12.26584779975369	3.37047961880592
N	5.75147560659719	11.81391927031375	2.56222281350504
C	3.94989666874123	11.48623314683138	4.19384930682688
C	2.64390602498382	11.11026372590368	4.62158049801539
H	1.82325638815997	10.82358596173334	3.98368234213635
C	4.51242083875719	11.43573523765496	2.90770220401434
C	3.74910378210625	10.90349463917259	1.76849281782479
C	2.99812325592012	9.73250888148163	1.85757070222432
H	2.97550630641700	9.18004545665104	2.79090093674688
C	3.80247850401620	11.55134829229001	0.53695103347557
H	4.39577069505466	12.45506951723855	0.44357059010402
C	3.10764286318893	11.06403183622436	-0.55129490763944
H	3.15470163474846	11.59897504135010	-1.49635124096566
C	2.31432409671070	9.24569073318219	0.76287899557715
H	1.74845831243690	8.32178378101427	0.85641898345822
C	2.34812628370217	9.90375421705232	-0.46197445849486
C	7.89675384783332	12.59063546903598	2.63620715269385
C	8.89346816128448	13.14046773430529	3.63210483358991
H	9.04978026612056	12.43530393629548	4.45850959603785

H	9.85763879321516	13.29499402326471	3.13586704660204
H	8.56406596192816	14.10773220869172	4.02749801725271
C	8.43544909563755	11.30542095501479	2.02606863808252
H	7.71591558251270	10.88977605747981	1.31389632171769
H	9.37440730963956	11.52087264340726	1.50216711749638
H	8.63444064378545	10.56248199404192	2.80919706505383
C	1.60000452429694	9.37172409633848	-1.63647939727978
H	1.91988710132560	8.34667774150151	-1.86970983126098
H	0.52171681008441	9.33603269905308	-1.42663538398947
H	1.75986866166626	9.99584894040837	-2.52144284834914
C	7.61376115770132	13.62583956379470	1.55919791819778
H	7.19647073472685	14.53650325543686	2.00560129245764
H	8.55047817890046	13.88291164937483	1.05012367981785
H	6.90738855184650	13.23075837287459	0.82285821627241
F	3.02981890694615	16.91920937216408	5.04574654360020
F	3.18320342510298	14.83547907145375	4.51426832640246
F	4.11540343439061	16.33399348916683	3.27745101807760
H	5.90268921356667	13.80920030967468	7.26071956824504

Table S56. Calculated coordinates of low spin [($^{t\text{Bu, Tol}}$DHP -H)Co]OTf (**4-LS**).

Co	6.33638362480113	12.65004787563723	5.20340964829650
O	6.21812574608762	14.60082547954793	4.69760937901436
N	4.76796973008128	11.68380075462852	5.29696149252513
N	6.27743039385915	13.04161145543291	7.20393183240640
N	5.67025238402299	12.12911869753185	8.07704696908834
C	4.03030292828532	11.46786634040396	6.40906280973856
C	2.73154758396136	11.06648327652338	6.03979455069867
H	1.92772592754184	10.84164788789372	6.72394472533627

C	4.59465488424212	11.51706749977029	7.72759182008867
C	3.94766283553217	10.74810321701763	8.81162710846301
C	3.46470853911971	9.45726538120661	8.61747135269080
H	3.54552498557718	8.99327941167717	7.63941859380696
C	3.84570600722517	11.30556338072491	10.08033515681982
H	4.21916620129255	12.31126693646332	10.24381211712164
C	3.27062300480215	10.59919774992702	11.11874548426374
H	3.19136166346127	11.06200066219845	12.09910363997304
C	2.90091290691115	8.75307581393757	9.66162971126348
H	2.54245317739609	7.74079613342093	9.48989346464287
C	2.78804107332026	9.31045210995396	10.93133347431779
C	7.51730209543044	13.54029161071034	7.92462931640157
C	8.16131200086775	14.66734308945942	7.14462900583640
H	8.47280342478062	14.36360014895335	6.14479201847612
H	9.05629458726662	14.98420755659575	7.69190418391968
H	7.49607244127943	15.53224058915100	7.05913946708649
C	8.47842534850255	12.37937711960959	8.07909266848584
H	8.00930346064191	11.56172386545519	8.63572076905307
H	9.36338920222741	12.71771667793226	8.63017334988120
H	8.80772702770829	12.00208795720743	7.10413272804019
C	2.16504732962802	8.54425444826217	12.04855176116178
H	2.69146620475684	7.59323231446684	12.20946835113135
H	1.11819395998651	8.30169391959482	11.81746730856660
H	2.18930535051379	9.11768777505987	12.98073205552203
C	7.11519439102646	14.08148181257848	9.28990750757738
H	6.35350723180314	14.86508684261239	9.18252283470631
H	7.99779632997209	14.52538376752606	9.76494663596046
H	6.72561370854893	13.28928952112926	9.93301123703458

C	4.02870380093740	15.74322199026431	3.85782364317449
S	5.34545058855111	15.70324283067479	5.15349082594738
O	4.64671628463527	15.38856108286210	6.37839991965850
O	5.97389642173845	16.98568576555761	5.05690880517055
N	6.57203713594281	12.11791982553382	3.45576323215717
N	5.73151304351695	11.72115643477056	2.58435060769169
C	3.98807259549806	11.38510011291277	4.21636685843100
C	2.69599679445370	11.03080787226242	4.67248530418087
H	1.85246080896428	10.78702704562538	4.04732692148218
C	4.47251545451642	11.41706333108318	2.89845489280460
C	3.63823920418375	11.05440719933179	1.74609176052217
C	2.83230714338017	9.91769744817937	1.73488324412090
H	2.81161403457130	9.26462965119528	2.60095735568887
C	3.68074686139432	11.83656719921965	0.59581743694600
H	4.30968607419968	12.72122538873780	0.58157123705088
C	2.92675020932469	11.50750122427242	-0.51304965174326
H	2.96863495466890	12.14436663208957	-1.39294794361017
C	2.08946916982692	9.58974142543497	0.61977291262494
H	1.48096069411796	8.68855965617386	0.63246912496145
C	2.11608007786085	10.37928831395307	-0.52498311571995
C	7.95212283376752	12.21787073897907	2.90612359926725
C	8.83155962020338	12.81239735126409	3.98500676203955
H	8.71869416820381	12.26400344600373	4.93392618169059
H	9.88425012712995	12.72518937064756	3.69669335746112
H	8.61001080841080	13.87385614004368	4.13260460469927
C	8.42338079192784	10.81483673747157	2.55741344595645
H	7.75463703009608	10.36504051895711	1.81644215102806
H	9.43607695578970	10.86685877217884	2.14014887823202

H	8.44445806468825	10.18165130163820	3.45349045112832
C	1.30675708296414	10.01511053886359	-1.72297795845421
H	1.58779969708833	9.01959212927424	-2.09404524379634
H	0.23703102467613	9.97934776914028	-1.47371829714446
H	1.45020974358108	10.73982787889922	-2.53076051197469
C	7.95652893271393	13.11653783398438	1.68127775061271
H	7.54699520515678	14.10207999083503	1.93199008612583
H	8.98994632305848	13.24178002831431	1.33616160826613
H	7.36239097424481	12.67413353449645	0.87651654805815
F	3.20007759832494	16.75288204798803	4.08954243638201
F	3.32865704483702	14.61463306236538	3.85354821215213
F	4.56871767083868	15.90694817416442	2.65470411346344
H	5.63413925755361	13.85573312415350	7.12416392386894

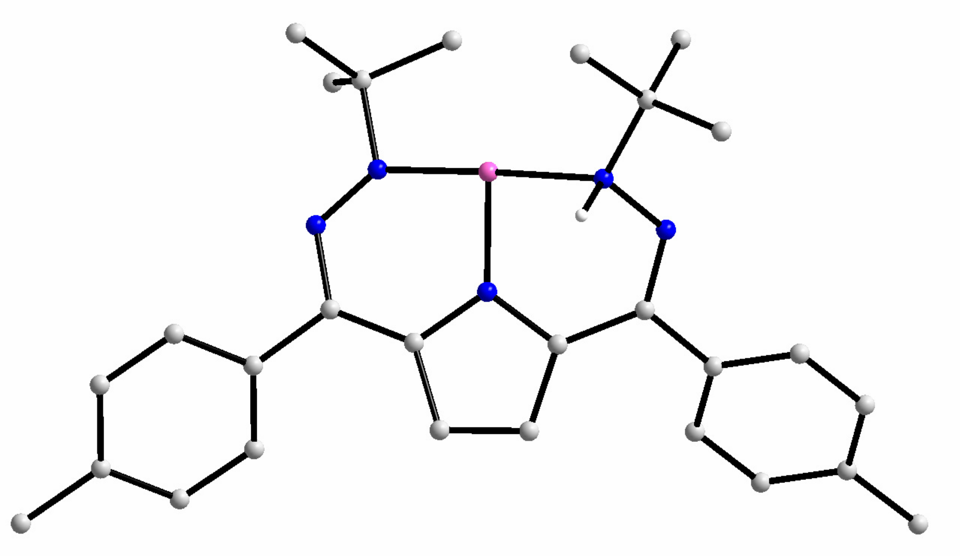

Figure S82. Calculated Structure of high spin [($^{tBu, Tol}$DHP -H)Co] (**4⁺-LS**). All C–H hydrogen atoms have been removed for clarity.

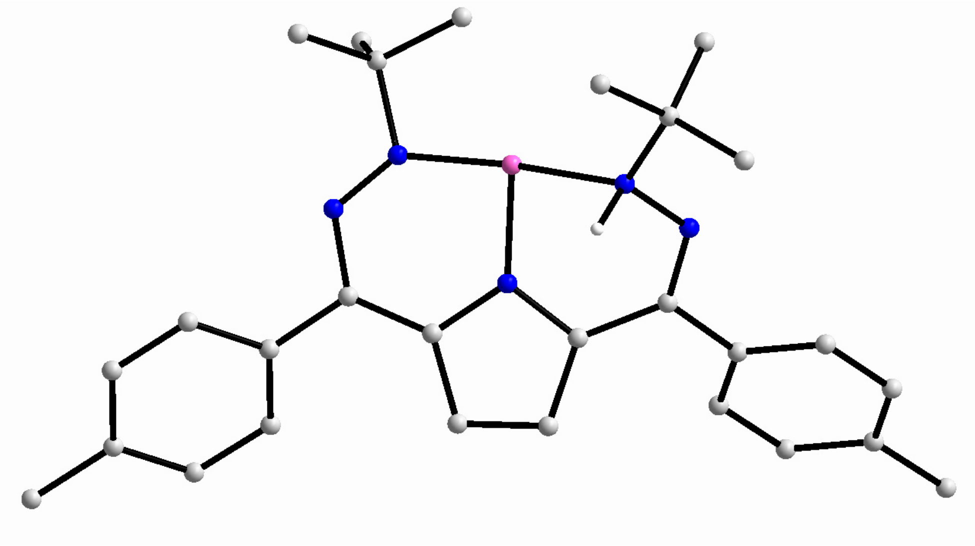

Figure S83. Calculated structure of low spin [($^{tBu, Tol}$DHP -H)Co] (**4**$^{+}$**-LS**). All C–H hydrogen atoms have been removed for clarity.

Table S57. Calculated coordinates of high spin [($^{tBu, Tol}$DHP -H)Co] (**4**$^{+}$**-HS**).

Co	6.61409662986703	12.54227036954242	5.17907371617000
N	4.93480601648953	11.84358998009669	5.25646354876667
N	6.31157553606001	13.26858879879456	7.02443466003325
N	5.92802408050474	12.29486134836166	7.96237701512280
C	4.21839471538889	11.63649779170338	6.39238343381838
C	2.89096838552694	11.35878739293154	6.04383126418601
H	2.08659805509868	11.16984359599181	6.73870273813628
C	4.87704351992492	11.60138667080553	7.68092605722042
C	4.38378336625478	10.69945437607971	8.72946970715109
C	3.79455973159675	9.47656986699592	8.42256642748737
H	3.68114436434340	9.17541737314444	7.38626161571320
C	4.52700445074654	11.04702105259429	10.07119277569310
H	4.97789007990157	12.00090368009311	10.32360258236850
C	4.09194336993358	10.19979661785142	11.06661692287690
H	4.20039556660776	10.49750217838025	12.10643285494483
C	3.36776176717702	8.63063182866297	9.42614540184901

H	2.91757581955222	7.67705243728884	9.16327727243633
C	3.50729830120063	8.97264257365046	10.76587355948159
C	7.24163131678064	14.26200827061875	7.67750352530721
C	7.47025229875580	15.38781563768000	6.68701832257274
H	8.02015746686644	15.06281589401488	5.79778319573040
H	8.06537298477910	16.17031451393888	7.16998045203960
H	6.51862992303027	15.83582476354052	6.36921997558768
C	8.53378332816268	13.57083364193990	8.05281378581191
H	8.33869392605333	12.74386422284289	8.74297272755124
H	9.19279309372443	14.29269601031276	8.54798853093633
H	9.05767442269160	13.18334188097558	7.17223514391970
C	3.06148853095487	8.05072976713516	11.84738029327171
H	2.68929311864138	8.61076379298598	12.71318479047183
H	3.90494930203269	7.43328685401110	12.19090820638648
H	2.27549610713824	7.37485802430080	11.49319946995660
C	6.55893065143117	14.82473618975186	8.91354640585847
H	5.60574107792995	15.30196496098431	8.64758362334926
H	7.20795031054466	15.58325143485603	9.36470999754909
H	6.36977893675824	14.03852302486435	9.64948618002972
N	6.72994363453698	12.10886970381442	3.43360627906718
N	5.88041204743235	11.81685734658616	2.53150704347771
C	4.11926832563006	11.63612960331901	4.18170041080755
C	2.81853425281936	11.38350566591647	4.67016278822966
H	1.93726207966712	11.23643644587078	4.06698514258051
C	4.59906608357477	11.60867469171475	2.84883033942285
C	3.73967715445968	11.29538798697059	1.70853777941610
C	2.78923629365531	10.27420095241200	1.74599289608433
H	2.68137875886395	9.66985506498229	2.63976196969207

C	3.90024127737879	11.99819609364805	0.51528561248774
H	4.64531145367183	12.78547153046683	0.46051520481127
C	3.11652942344003	11.71404000168362	-0.58282827166977
H	3.24997578576030	12.28822808817523	-1.49570940296366
C	2.02032631390831	9.98779159441740	0.63928811291821
H	1.30070286222436	9.17440060207596	0.68772457070384
C	2.15921773382024	10.70609113482167	-0.54490154875233
C	8.15203413403773	12.16392840165781	3.07029727401138
C	8.76613384389918	12.44787034675228	4.43301168005970
H	8.67471509037791	11.58224334331545	5.11582540633048
H	9.83012192444853	12.70070722903705	4.38942547374187
H	8.32030782317075	13.38453454052972	4.87224423676290
C	8.62647262323048	10.83863769026846	2.51064671547687
H	8.12835775116394	10.63575452153693	1.55703764437944
H	9.70914610241524	10.88272611294579	2.34287948413704
H	8.40863513475294	10.02146100865277	3.20885314585293
C	1.31312459510180	10.39348518861902	-1.72958855690419
H	0.25508066056641	10.59672712520240	-1.51089996260727
H	1.60877894730452	10.99223382884188	-2.59657446467923
H	1.39124126487826	9.32971371285502	-1.99216688645304
C	8.39838281664666	13.30345912906670	2.09928384663336
H	8.06685353585777	14.25857434691394	2.52491686585883
H	9.47031103594538	13.36623024801657	1.87748159609782
H	7.85380341422351	13.11882168995258	1.16767399460399
H	5.46671526868462	13.81801821123647	6.78573740059769

Table S58. Calculated coordinates of low spin [($^{tBu, Tol}$DHP -H)Co] (**4^{+}-LS**).

Co	6.67941978911565	12.34145304649281	5.22428668218065

N	4.94659796835511	11.81905490670091	5.27745496598237
N	6.35775148062624	13.20884523803324	7.02413824813181
N	5.93139041917933	12.26549836124306	7.97886738066229
C	4.22733452708330	11.61600860770519	6.40036851126596
C	2.87986390120540	11.38921097679600	6.05703344428116
H	2.07457628109540	11.22426063261894	6.75678415167866
C	4.88024312975579	11.57831408653858	7.69902375001967
C	4.36868546058631	10.68679554420792	8.74652433207520
C	3.74759753317401	9.48106925572437	8.43481885662764
H	3.61838226221386	9.18975060759954	7.39740482802852
C	4.52989850599949	11.02147804426658	10.08956202525444
H	5.00572714693982	11.96217712249751	10.34559699665969
C	4.08244895928108	10.17705435988264	11.08149910730162
H	4.20576637247444	10.46400709234260	12.12267669010672
C	3.30931204479066	8.63721393029612	9.43532281534685
H	2.83494647366791	7.69652974433907	9.16874504813554
C	3.46764147085288	8.96551741015096	10.77619841886782
C	7.26483367879507	14.21646631048710	7.68404314656578
C	7.46736364910073	15.34921290349252	6.69750465758131
H	7.97035687349676	15.02032252333985	5.78439722107299
H	8.09236100431024	16.11885976809566	7.16317507173657
H	6.50829231447182	15.81092618095080	6.42577493933289
C	8.56973306811910	13.54806532053041	8.05518454460108
H	8.38494342509873	12.68842997328579	8.70821813109328
H	9.20053473901326	14.26483568787223	8.59259043875914
H	9.11783150951016	13.21310914654138	7.16786775645592
C	3.01027396862171	8.04578229251161	11.85463297397826
H	2.64287384108214	8.60766063213554	12.72137823893648

H	3.84697424954215	7.41911629881083	12.19801927168860
H	2.21780817137662	7.37909194229058	11.49777317487052
C	6.57375079288429	14.76434072970743	8.92288455916341
H	5.59439808191455	15.18980023062771	8.66477095318258
H	7.19222668287218	15.56227386896498	9.34853950622603
H	6.43451563149516	13.98557794517825	9.67740702754055
N	6.74743169160981	12.05252983948599	3.46940852169844
N	5.88367300675785	11.84363829346012	2.55782046622794
C	4.11775967967676	11.65411399469160	4.20248896006259
C	2.80222950638971	11.43647101865686	4.69174975896854
H	1.91503233341118	11.33213936088111	4.08834145677516
C	4.59595491427340	11.62444126121565	2.88350716946680
C	3.74731487664014	11.31578448903636	1.73896395609978
C	2.76445387704357	10.32434981298090	1.78251293156956
H	2.63280252590259	9.73479739739885	2.68279576524188
C	3.94584262429591	11.99411159723285	0.53541154101270
H	4.71767111935049	12.75427911478652	0.47788336579850
C	3.16086351002008	11.72188230627369	-0.56336267076482
H	3.31938041496812	12.27844232333064	-1.48294951132400
C	1.99835598547691	10.04661409621294	0.67319009139409
H	1.25463345364519	9.25554732384269	0.72404573423529
C	2.17045386976513	10.74579024353216	-0.51905234736469
C	8.14889006418688	12.19040375847437	3.03412257501022
C	8.81875820336394	12.71453004951768	4.29491475223200
H	8.54416931743742	12.08991131967239	5.19391665512975
H	9.90714690311700	12.59527357219497	4.26929236877791
H	8.60329280161359	13.77599115104675	4.45056326305470
C	8.67242645178451	10.81545993396915	2.65789195180246

H	8.09749303919800	10.41774865862342	1.81483661504775
H	9.72524623263417	10.90052418120427	2.36397560642171
H	8.59581601414637	10.12255483296013	3.50474170259920
C	1.32481240915853	10.44440366410124	-1.70566024594862
H	0.27352477115483	10.69121175965078	-1.49866320691421
H	1.64976078525783	11.01580500637355	-2.58038310800732
H	1.36399851471874	9.37348674377604	-1.94696478245457
C	8.28778744269869	13.17242252393433	1.89051335035272
H	7.82453414875102	14.13267795935722	2.14717079377340
H	9.35359675827053	13.33722038439249	1.69195242968383
H	7.81681565090248	12.77650401558868	0.98597870363170
H	5.53220169427673	13.75053728988356	6.71826752131714

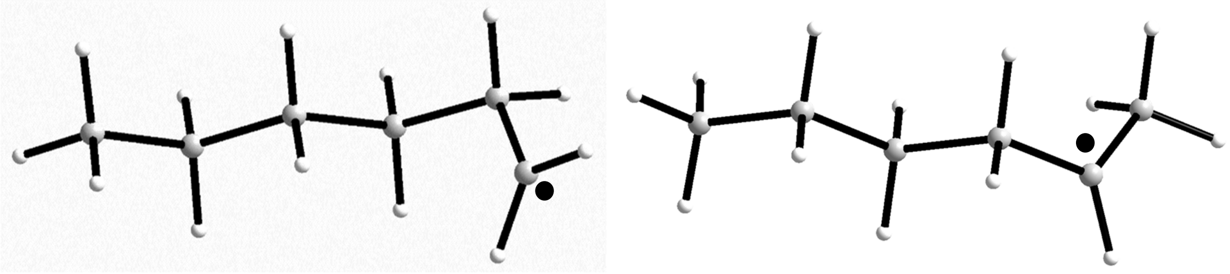

Figure S84. Calculated structures of 1-hexene primary and secondary radicals.

Table S59. Calculated Coordinates of 1-hexene primary radical.

C	2.69238778014071	0.56628617093442	0.99955790520879
C	2.07745587247848	-0.76470728179138	0.87525727261444
C	0.55991494523665	-0.76501028039489	0.91602516125933
C	-0.08342145098594	-0.11414863252920	-0.29004453257215
C	-1.59571216677795	-0.08850230304471	-0.23398804209964
C	-2.22858124319095	0.54861676400588	-1.45119663670799
H	0.22983770725558	-0.24283278344672	1.82909716418801

H	0.20256987759920	-1.80279384692410	0.99736812735158
H	0.23495945867246	-0.65169579846696	-1.19893669877664
H	0.29199378024930	0.91650971255303	-0.39036156774440
H	-1.91127686274483	0.45627323659659	0.67008053941097
H	-1.96860821995723	-1.11932368145762	-0.12329181956660
H	-1.95081766249865	0.00479073187465	-2.36503558557306
H	-1.89360846567275	1.58917598335387	-1.56520023696497
H	-3.32346652457135	0.55007857751220	-1.37606800235735
H	2.42396013974872	-1.25055988020591	-0.05433872871269
H	2.44904277837966	-1.41855377385372	1.69254438447864
H	3.73448486316526	0.73004525743610	0.73656737584950
H	2.15624539347363	1.38165182784848	1.48235392071423

Table S60. Calculated coordinates of 1-hexene secondary radical**.**

C	2.83966229822937	0.49687392702078	1.23436075154405
C	1.98629595704609	-0.69029842451832	1.05161628450824
C	0.51275686270565	-0.59708099483109	1.06978117714571
C	-0.10250978875641	-0.13822153276237	-0.25499276732673
C	-1.61310209177989	-0.04923478716670	-0.21388946683174
C	-2.21946141026825	0.40352271762599	-1.52375323827672
H	0.19842803828894	0.11552750860945	1.85197363136171
H	0.07680856577886	-1.57638123974866	1.32349141461583
H	0.20077159296838	-0.83764832049498	-1.04991341409666
H	0.31341873976150	0.84569877493161	-0.52170756880007
H	-1.90955837621371	0.64559052428324	0.58788422915778
H	-2.02225745022549	-1.03504093841285	0.05864563167691
H	-1.95727121071392	-0.28951700272707	-2.33540094837300
H	-1.85037330490795	1.40086728651254	-1.80095911036488

H	-3.31404728425488	0.45214232423513	-1.45876989898084
H	2.44286920318285	-1.60815948896251	0.68116544256081
H	2.84023415075339	1.15143686604695	0.33893575683215
H	3.88389656251710	0.22194761720560	1.42860491565528
H	2.47864894588836	1.12151518315328	2.06742717799218

Table S61. Compared energies of radical-derived intermediates along catalytic cycle for 1-hexene hydrogenation without accounting for entropic contributions.

	**Primary 1-hexene radical**	**Secondary 1-hexene radical**	**4-HS**	**4^{+}-HS**	**4-LS**	**4^{+}-LS**
HS- S = 1 (Eh)			-3702.65	-2741.719		
S = 1/2 (Eh)	-235.86	-235.86				
LS- S = 0 (Eh)					-3702.62	- 2741.716

Table S62. Compared Gibbs free energies of radical-derived intermediates along catalytic cycle for 1-hexene hydrogenation with accounting for entropic contributions.

	**Primary 1-hexene radical**	**Secondary 1-hexene radical**	**4-HS**	**4^{+}-HS**
HS- S = 1 (Eh)			-3702.11	-2741.21
S = 1/2 (Eh)	-235.72	-235.73		

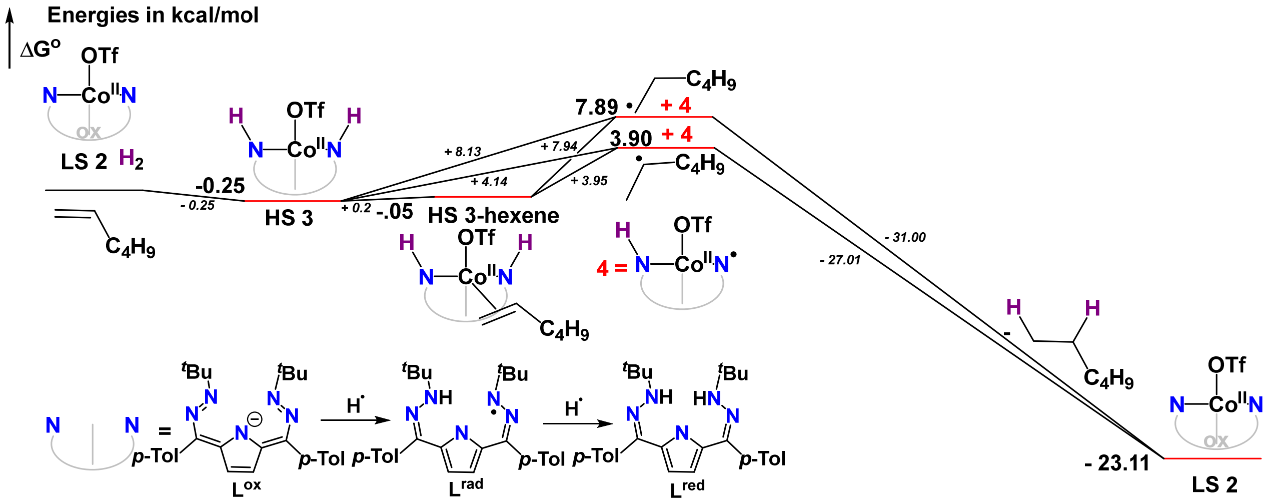

Figure S85. Simplified catalytic steps with radical pathways towards 1-hexene hydrogenation (with accounting for entropic contributions).

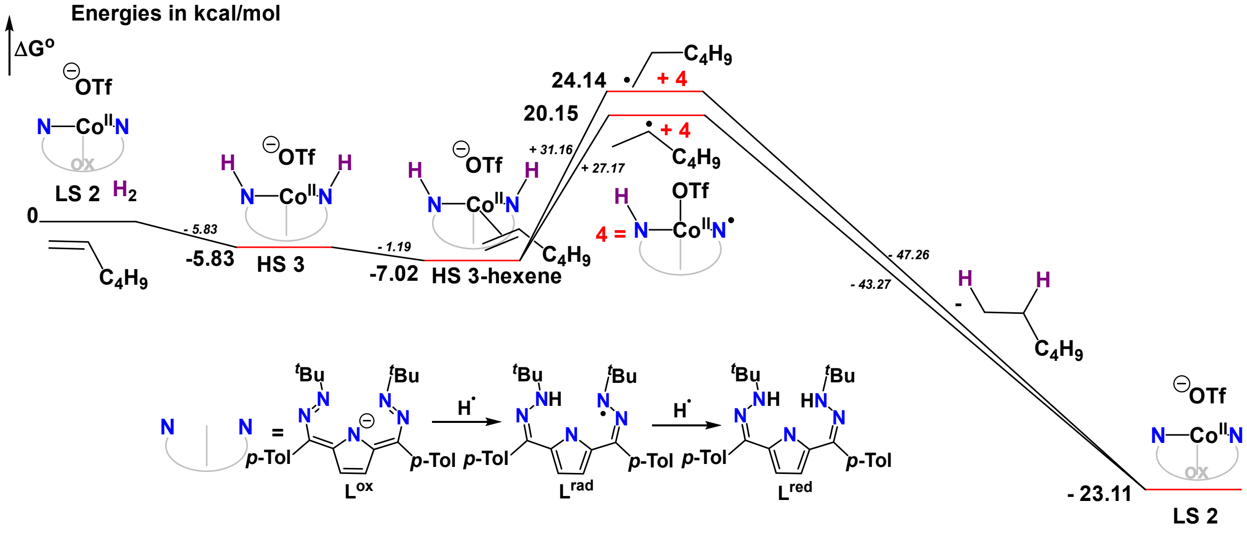

Figure S86. Simplified catalytic steps with radical pathways towards 1-hexene hydrogenation without triflate (accounting for entropic contributions).

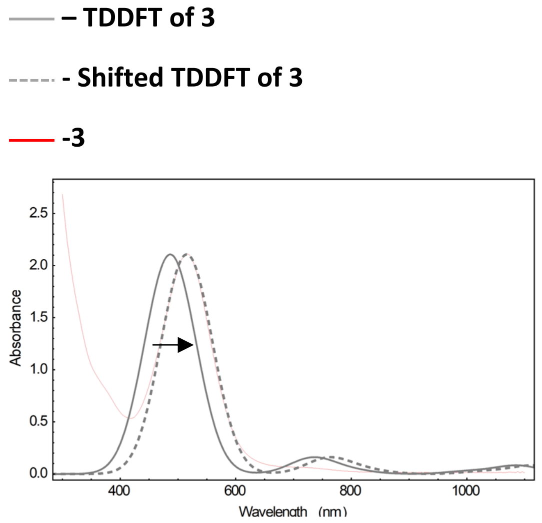

Figure S87. TDDFT of **3**. Note that TD-DFT typically underestimates the energies of transitions and a shift to match experimental data of 50-75 nm is common in the related Ni- and Fe-DHP complexes.

Table S63. Calculated vs. experimental values.

	N–H [a]	N–D [a]
experimental value	2811-2881 (avg. 2846)	~2050, range: 2031-2144 (avg. 2088)
theoretical value	3031	2115 (calc.)

Scaling Factor for B3P/TZVP[8]	0.964	
Scaled theoretical value	2922	2038
Theor./Exp. Ratio	0.99	-

[a] units of cm^{-1}

The theoretical stretching frequencies were determined from the B3P DFT calculation for the structure of **3**. The ratio of the theoretical stretches compared to the observed is 0.99 for the more resolvable N-D stretches, or in other words, shows that the data observed is a very good match for the theoretical values.

Equation S1. IR Verification Calculations (units on all IR frequencies are in cm^{-1}).

$$\mathrm{ve} = \frac{1}{2\pi}\sqrt{\frac{k}{\mu}}$$

$$\mu\mathrm{d} = \frac{14.01 * 2.014}{14.01 + 2.014} = 1.761, \qquad \mu\mathrm{h} = \frac{14.01 * 1.008}{14.01 + 1.008} = 0.9403$$

$$2050 = \frac{1}{2\pi}\sqrt{\frac{k}{1.761}}$$

$$k = 2.921 \times 10^{8}$$

$$\mathrm{veh} = \frac{1}{2\pi}\sqrt{\frac{2.921 \times 10^{8}}{0.9403}}$$

$$\mathrm{veh} = 2805 \rightarrow 0.7\%\ off\ from\ experimental\ average\ (2846)$$

$$2846 = \frac{1}{2\pi}\sqrt{\frac{k}{0.9403}}$$

$$k = 3.007 \times 10^{8}$$

$$\mathrm{veh} = \frac{1}{2\pi}\sqrt{\frac{3.007 \times 10^{8}}{1.761}}$$

$$\mathrm{veh} = 2080 \rightarrow 0.7\%\ off\ from\ experimental\ average\ (2050)$$

Mass Spectrometry

**Data File**	010522-01052022_ldhcootf-a.d	**Sample Name**	01052022_ldhcootf
**Sample Type**	Sample	**Position**	P1-A-07
**Instrument Name**	G6224A TOF LCMS	**User Name**	Anderson-Sophie.Whitmeyer
**Acq Method**	FIA_P_M-Mixed_No-Water_50-3200_130V.m	**Acquired Time**	1/5/2022 11:09:57 AM
**IRM Calibration Status**	Success	**DA Method**	FIA_P_M-Mixed_No-Water_50-3200_130V.m
**Comment**			

Compound Table

Label	Tgt Score	Mass Error (ppm)	Tgt Formula	Obs. RT	Ref. Mass	Obs. Mass
Cpd 1: C29 H35 D Co F3 N5 O3 S	8.25	19.45	C29 H35 D Co F3 N5 O3 S	0.118	651.1886	651.2012

Figure S88. HRMS of **3**. Due to the mixed H_2 and D_2 atmosphere, the mass is not exact, but it shows that there's a relatively clean formation of one hydrogenated species.

General Catalytic Hydrogenation Procedures and Products

Procedure for 1% loading:

In a nitrogen-filled glovebox, a 250 mL Schlenk flask with a magnetic stir bar was charged with unsaturated substrate (0.077 mmol, 100 eq.), **2** (0.0005 g, 0.0008 mmol), mesitylene (0.002 mL, 0.014 mmol) (internal standard), and benzene-d_6 (0.1 mL). On a Schlenk line, the solution was freeze-pump-thaw-degassed, and warmed to room temperature with the contents under static vacuum. At room temperature, this vessel was backfilled with 1 atm H_2 gas. The vessel was then sealed and left to stir for 18 hr. The dark red-purple **2** could be observed to pinken within the hour, turn greenish-red, and then begin to turn orange/yellow after 6 hours. After 18 hr, the vessel was shipped back into the nitrogen-filled glovebox and diluted to 0.7 mL total volume. This was then analyzed via ^{1}H and ^{19}F NMR, and checked by GC-MS as needed.

Procedure for 2.5% loading:

The general method described above was implemented with 0.031 mmol, 40 equiv. of unsaturated solvent used.

Procedure for 2.5% loading with $NaBAr^F{}_4$:

The general method described above was implemented with 0.031 mmol, 40 equiv. of unsaturated solvent used and with the addition of 0.0008 mmol, 1 equiv. of $NaBAr^F{}_4$ pre-added to the reaction vessel with 0.07 mL of THF.

Procedure for 10% loading:

In a nitrogen-filled glovebox, a 250 mL Schlenk flask with a magnetic stir bar was charged with unsaturated substrate (0.0077 mmol, 10 eq.), **2** (0.0005 g, 0.0008 mmol), mesitylene (0.002 mL, 0.014 mmol) (internal standard), and benzene-d_6 (0.1 mL). On a Schlenk line, the solution was

freeze-pump-thaw-degassed, and backfilled at 77 K with 3.8 atm H_2 gas. The vessel was then sealed and left to stir for 18 hr. After 18 hr, the vessel was shipped back into the nitrogen-filled glovebox and diluted to 0.7 mL total volume. This was then analyzed via ^{1}H and ^{19}F NMR as well as checked by GC-MS as needed.

1-Hexene: The title compound was purchased from TCI Chemicals and dried according to the general procedure for olefin preparations. The spectral data collected matched those previously reported.[9] ^{1}H NMR (400 MHz, C_6D_6): δ 0.83 (t, 3H, CH_3), 1.25 (m, 4H, CH_2CH_2CH$_3$), 1.96 (m, 2H, CHCH_2), 4.98 (m, 2H, CH=CH_2), 5.75 (m, 1H, C*H*=CH$_2$).

Hexane: Hydrogenation of 1-hexene by the general procedure for 1% cat. loading yielded hexane as follows: (79.5, 72.0, 86.0) *79(6)*% unisolated yields. The spectral data collected matched those previously reported.[10] ^{1}H NMR (400 MHz, C_6D_6): δ = 1.19~1.32 (m, 8 H), 0.89 (t, 6 H).

1-Hexyne: The title compound was purchased from Sigma Aldrich and dried according to the general procedure for olefin preparations. The spectral data collected matched those previously reported.[9] ^{1}H NMR (400 MHz, C_6D_6): δ 0.71 (t, 3H, CH_3), 1.19–1.33 (m, 4H, CH_2 CH_2), 1.77 (t, 1H, ≡C*H*), 1.93 (dt, 2H, ≡CHCH_2).

Hexane and 1-Hexene: Hydrogenation of 1-hexyne by the general procedure for 1% cat. loading yielded hexane and 1-hexene as follows: (68.3, 67.9, 69.8) *69(1)*% and (5.1, 10.4, 1.9) *6(3)*% respectively unisolated yields. The spectral data collected matched those previously reported (see above for NMR peaks).[9,10]

Styrene: The title compound was purchased from Acros Organics and dried according to the general procedure for olefin preparation. The spectral data collected matched those previously reported.[9] ^{1}H NMR (400 MHz, C_6D_6): δ 5.06 (dd, 1H, *(E)*-C*H*H), 5.59 (dd, 1H, *(Z)*-CH*H*), 6.57 (dd, 1H, C*H*), 7.01–7.05 (m, 1H, *p*-C_6H_5), 7.08–7.12 (m, 2H, *m*-C_6H_5), 7.21–7.24 (m, 2H, *o*-C_6H_5).

Ethyl benzene: Hydrogenation of styrene by the general procedure for 1% cat. loading yielded ethyl benzene as follows: (52.6, 61.2, 55.5) *55(4)%* unisolated yields. The spectral data collected matched those previously reported.[11] ^{1}H NMR (400 MHz, C_6D_6): δ = 7.20-7.06 (m, 5H, ar), 2.45 (sept, 1H, CH), 1.49 (d, 6H, Me).

3,3-Dimethylbutene: The title compound was purchased from Sigma Aldrich and dried according to the general procedure for olefin preparations. The spectral data collected matched those previously reported.[9,11] ^{1}H NMR (400 MHz, C_6D_6): δ = 5.84-5.79 (3 H), 0.96 (s, 9 H, ^{t}Bu).

2,2-Dimethylbutane: Hydrogenation of 3,3-dimethylbutene by the general procedure for 1% cat. loading yielded 2,2-dimethylbutane as follows: (74.2, 70.7, 70.0) *72(2)%* unisolated yields. The spectral data collected matched those previously reported.[10,11] ^{1}H NMR (400 MHz, C_6D_6): δ = 1.19 (q, J = 7.5 Hz,2 H), 0.85 (s, 9 H), 0.81 (t, J = 7.5 Hz, 3 H).

α-Methyl styrene: The title compound was purchased from TCI Chemicals and dried according to the general procedure for olefin preparations. The spectral data collected matched those previously reported.[1] ^{1}H NMR (400 MHz, C_6D_6): δ = 7.36-7.35 (m, 3H), 7.15-7.09 (d, 2 H), 5.35(s, 1H), 5.00 (s, 1H), 1.96 (s, 3H, Me). The hydrogenation of this complex under the NaBAr$_F$[4]

containing conditions lead to hydrogenated products derived from radical coupling reactions, namely III and V as listed in this resource.[12] (III) ^{1}H NMR (400 MHz, C_6D_6): δ = 7.00-7.30 (m, 10 H), 5.10 (d, 1 H), 4.74 (d, 1 H), 2.71 (s, 2 H), 1.15 (s, 6 H). (V) ^{1}H NMR (400 MHz, C_6D_6): δ = 7.00-7.30 (m, 9 H), 2.33 (d, 1 H), 2.05 (d, 1 H), 1.58 (s, 3 H), 1.24 (s, 3 H), 1.00 (s, 3 H).

Isopropyl benzene (cumene): Hydrogenation of α-methyl styrene by the general procedure for 2.5% cat. loading yielded isopropyl benzene as follows: (27.2, 30.0, 26.7) *28(1)%* unisolated yields. Utilizing the procedure for 2.5% catalyst loading with $NaBAr_F{}^4$ co-catalyst yield isopropyl benzene as follows: (45.24, 49.90, 48.27) 48(2)% unisolated yields. The spectral data collected matched those previously reported.[11] ^{1}H NMR (400 MHz, C_6D_6): δ = 7.19-7.06 (m 5 H), 2.74-2.50 (m, 1 H, $CHMe_2$), 1.14-1.13 (d, 6 H, Me).

β-Methyl styrene: The title compound was purchased from Sigma Aldrich and dried according to the general procedure for olefin preparations. The spectral data collected matched those previously reported.[10] ^{1}H NMR (400 MHz, C_6D_6): δ = 7.25-7.00 (m, 5 H), 6.25 (d, 1 H), 6.0 (m, 1 H), 1.60 (d, 3 H).

n-Propyl benzene: Hydrogenation of β-methyl styrene by the general procedure for 2.5% cat. loading with $NaBAr^F{}_4$ co-catalyst yielded n-propyl benzene as follows: (5.60, 6.06, 4.66) *5.4(0.6)%* unisolated yields. The spectral data collected matched those previously reported.[10] ^{1}H NMR (400 MHz, C_6D_6): δ = 7.18 (d, 2 H), 7.03~7.11 (m, 3 H), 2.3 (t, 2 H), 1.52 (m, 2 H), 0.83 (t, 3 H).

Benzoquinone: The title compound was purchased from Sigma Aldrich and dried according to the general procedure for olefin preparations. The spectral data collected matched those previously reported.[13] ^{1}H NMR (400 MHz, C_6D_6): δ = 5.92 (s, 4H)

Hydroquinone: Hydrogenation of benzoquinone by the general procedure for 2.5% cat. loading yielded hydroquinone as follows: (43.0, 43.4, 36.2) *41(3)%* unisolated yields. The spectral data collected matched those previously reported.[11] ^{1}H NMR (400 MHz, C_6D_6): δ = 7.85 (s, 2H), 6.36 (s, 4H).

2-methyl-pent-1,3-ene: The title compound was purchased from Sigma Aldrich and dried according to the general procedure for olefin preparations. The spectral data collected matched those previously reported.[14] ^{1}H NMR (400 MHz, C_6D_6): *δ* = 4.792 (d, 1H), 4.785 (d, 1H), 6.102 (d, 1 H), 5.578 (q, 1H), 1.765 (s, 3H), 1.708 (d, 3 H).

4-methyl-2-pentene (cis- and trans-) and 2-methyl-pent-2-ene: Hydrogenation of 2-methyl-pent-1,3-ene by the general procedure for 2.5% cat. loading yielded products as follows: *trans-4-Methyl-2-pentene (trans-E):* (19.3, 10.7, 9.3) *13(4)% cis-4-Methyl-2-pentene (cis-E):* (9.7, 6.5, 6.5) *8(1)% and 2-methyl-pent-2-ene:* (10.0, 15.6, 15.4) *14(3)%* unisolated yields. The spectral data collected matched those previously reported.[15] ***cis*-4-Methyl-2-pentene (*cis*-E):** ^{1}H NMR (400 MHz, C_6D_6): *δ* = 5.36–5.20 (m, 2 H, =CHMe + =CH-*i*Pr), 2.55 (m, 1 H, -$CHMe_2$), 1.51 (dd, 3 H, CH_3-CH=), 0.91 [d, $^3J_{H,H}$ = 6.4 Hz, 6 H,-$C(CH_3)_2$] ppm. ***trans*-4-Methyl-2-pentene (*trans*-E):** ^{1}H NMR (400 MHz, C_6D_6): *δ* = 5.72 (m, 1 H, =CH-*i*Pr), 5.34 (dq, 1 H, =CH-Me), 2.18 (m, 1 H, -$CHMe_2$), 1.56 (dd, 3 H, CH_3-CH=), 0.93 [d, 6 H, -$C(CH_3)_2$] ppm. **2-Methyl-2-pentene:** ^{1}H NMR

(400 MHz, C_6D_6): δ = 5.17-5.10 (m, 1H), 2.02-1.90 (m, 2H), 1.63 (d, 3H), 1.51 (d, 3H), 0.92 (t, 3H).

α-cyclopropyl styrene: The title compound was synthesized according to literature procedures[16] and dried according to the general procedure for olefin preparations. The spectral data collected matched those previously reported[17]**:** ^{1}H NMR (400 MHz, C_6D_6): δ = 5.25 (1H), 4.87 (1H). ^{1}H NMR (400 MHz, $CDCl_3$): δ 7.60 (d, 2H), 7.34 (t, 2H), 7.29 (d, 1H), 5.28 (s, 1H), 4.94 (s, 1H), 1.69-1.62 (m, 1H), 0.86-0.82 (m, 2H), 0.62-0.58 (m, 2H).

Pentan-2-ylbenzene and (E)-pent-2-en-2-ylbenzene: Hydrogenation of α-cyclopropyl styrene by the general procedure for 10% cat. loading yielded products as follows: *pentan-2-ylbenzene* (31.0, 37.3, 35.4) *35(3)% and (E)-pent-2-en-2-ylbenzene:* (11.0, 11.2, 7.5) *10(2)%* unisolated yields. The spectral data collected matched those previously reported. [18] **Pentan-2-ylbenzene**: H NMR (400 MHz, $CDCl_3$) δ = 7.33 - 7.28 (m, 2 H), 7.23 - 7.15 (m, 3 H), 2.71 (sxt, 1 H), 1.65 - 1.49 (m, 3 H), 1.35 - 1.15 (m, 5 H), 0.88 (t, 3 H). **(E)-pent-2-en-2-ylbenzene**: H NMR (400 MHz, $CDCl_3$) δ: 1.06 (t, 3H), 2.03 (s, 3H), 2.21 (dq, 2H), 5.77 (t, 1H), 7.21 (dd, 1H), 7.30 (dd, 8.0 Hz, 2H), 7.38 (d, 2H).

Controls

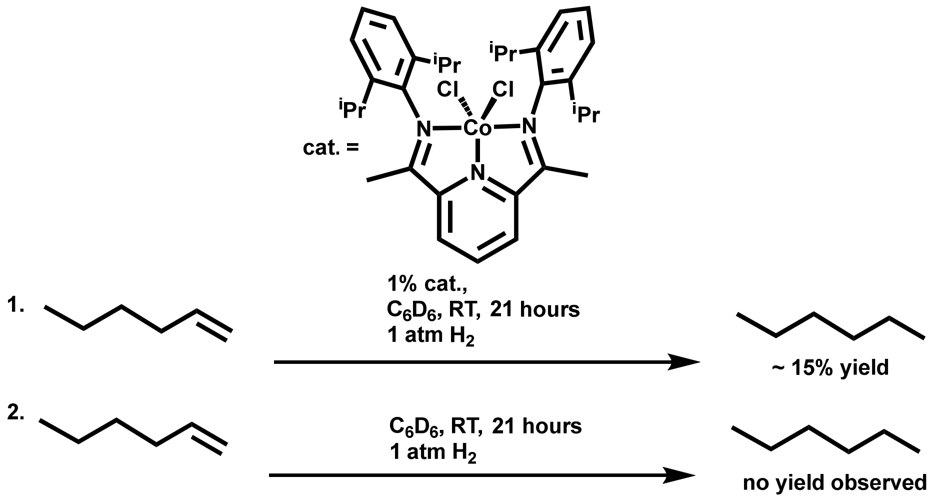

Figure S89. Controls (1.) with alternative cobalt catalyst[19] and (2.) with no cobalt-containing species. Some yield is observed from the alternative catalyst, but it is significantly less than that observed with **2**, and no quantifiable yield was observed in the absence of cobalt catalyst.

NMR data from Hydrogenations: Figures S90-100[20]

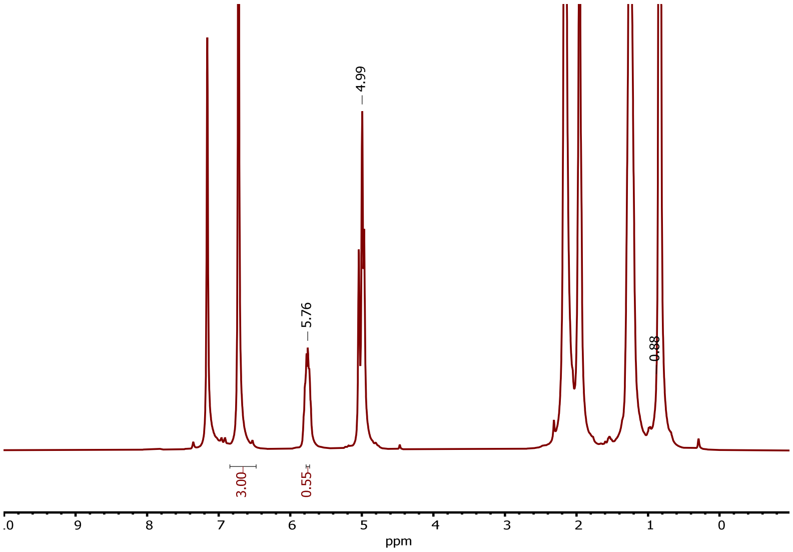

Figure S90.^{1}H NMR of 1 mol%: **2** hydrogenation of 1-hexene with 1 atm H_2 in C_6D_6 with 4 μL mesitylene internal standard. Integration of hexanes product done via subtraction from 1-hexene peaks (labeled) due to overlap in the aliphatic region.

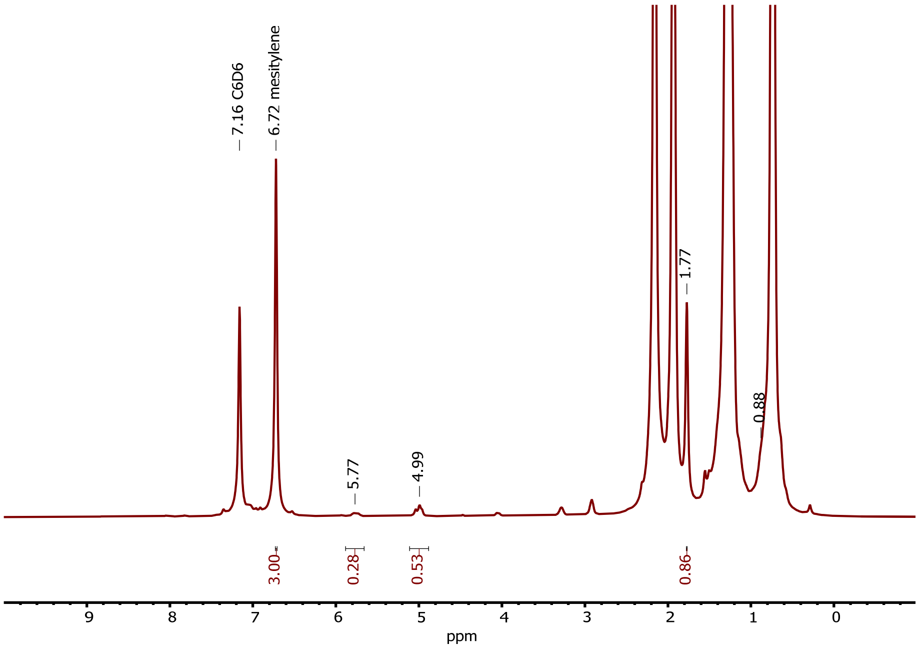

Figure S91. ^{1}H NMR of 1 mol%: **2** hydrogenation of 1-hexyne with 1 atm H_2 in C_6D_6 with 4 μL mesitylene internal standard. Integration of hexanes product done via subtraction from 1-hexyne peaks (labeled) due to overlap in the aliphatic region. 1-hexene product integrated directly.

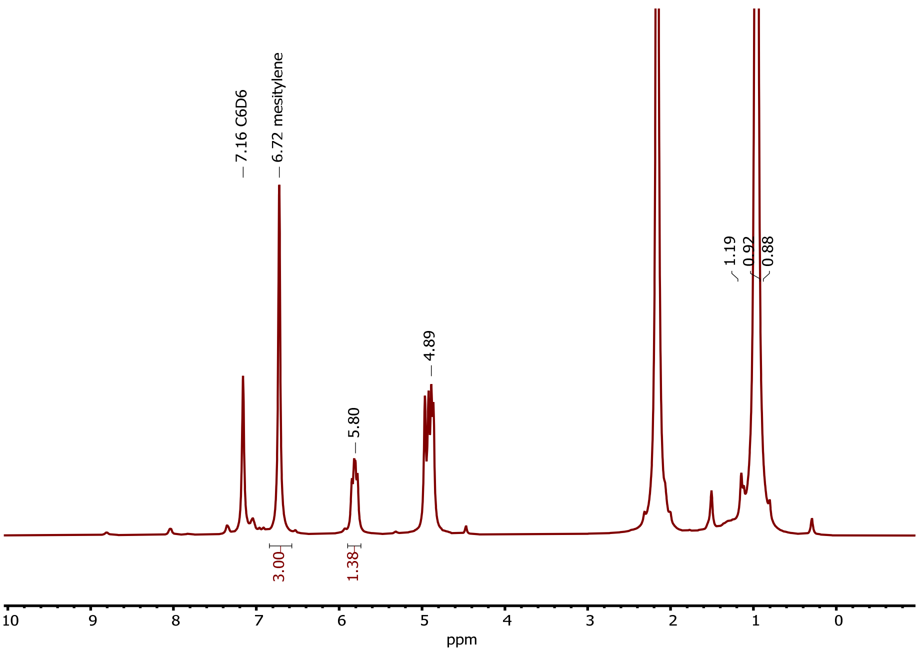

Figure S92. ^{1}H NMR of 1 mol%: **2** hydrogenation of 3,3-dimethylbutene with 1 atm H_2 in C_6D_6 with 2 μL mesitylene internal standard. Integration of 2,2-dimethylbutane product done via subtraction from 3,3-dimethylbutene peaks (labeled) due to overlap in the aliphatic region.

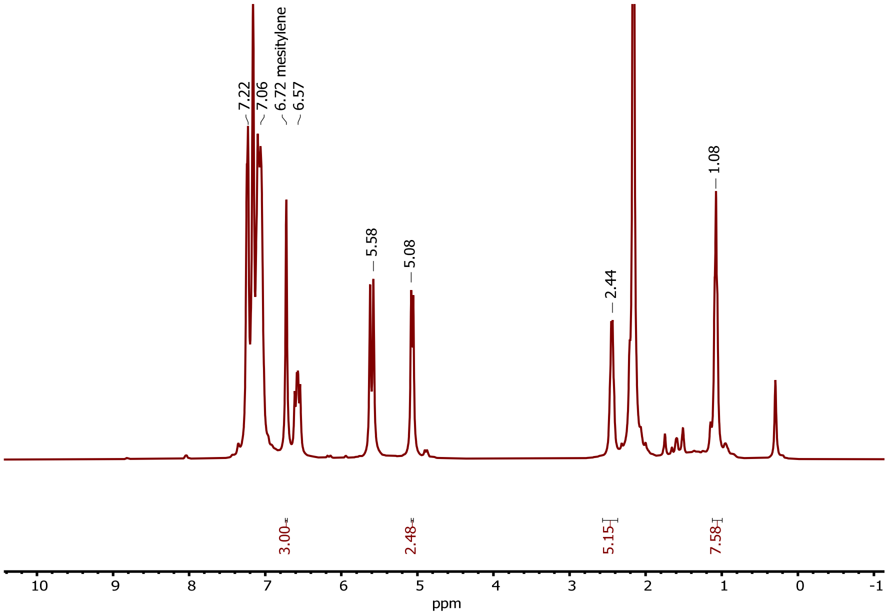

Figure S93. ^{1}H NMR of 1 mol%: **2** hydrogenation of styrene with 1 atm H_2 in C_6D_6 with 2 μL mesitylene internal standard. Integration of ethyl benzene product done via subtraction from styrene peaks (labeled) due to overlap in the aliphatic region.

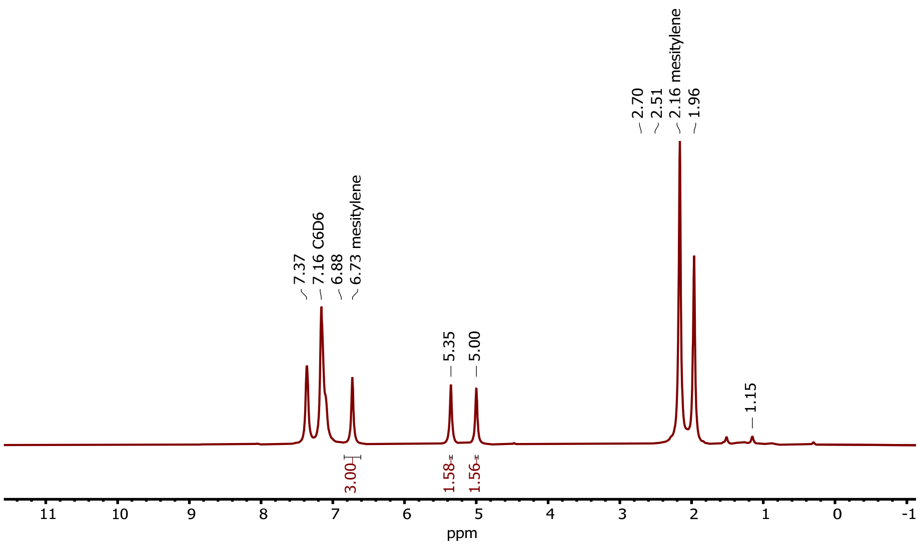

Figure S94.^{1}H NMR of 2.5 mol%: **2** hydrogenation of α-methylstyrene with 1 atm H_2 in C_6D_6 with 1 μL mesitylene internal standard. Integration of cumene product done via subtraction from α-methylstyrene peaks (labeled) due to overlap in the aliphatic region with mesitylene.

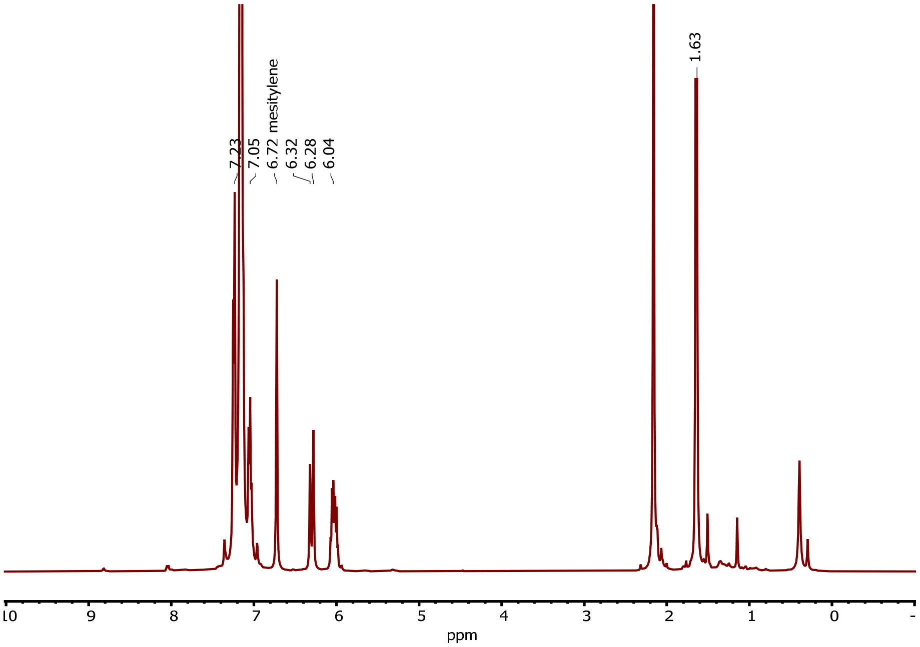

Figure S95. ^{1}H NMR of unsuccessful 2.5 mol%: **2** hydrogenation of β-methylstyrene with 1 atm H_2 in C_6D_6 with 1 μL mesitylene internal standard.

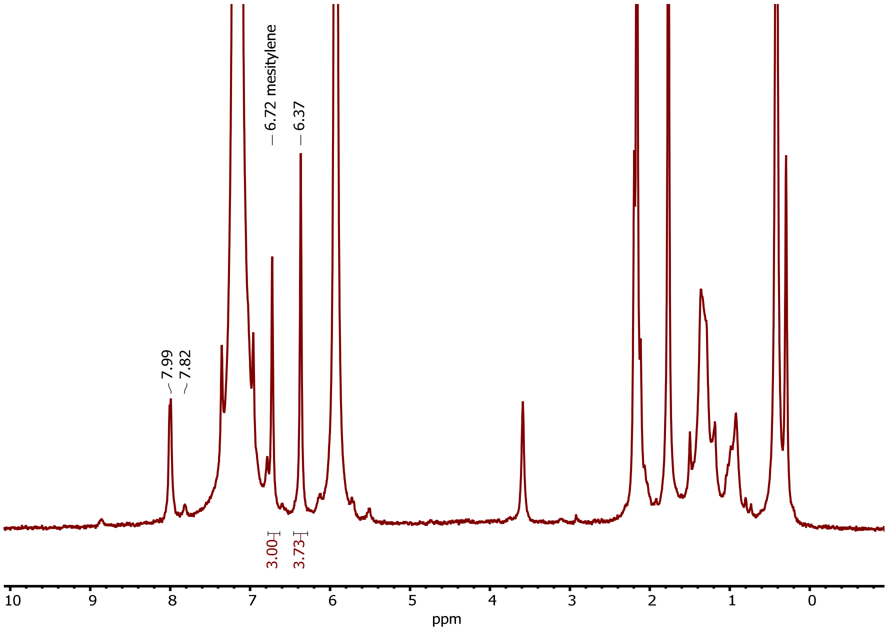

Figure S96. ^{1}H NMR of 2.5 mol%: **2** hydrogenation of benzoquinone with 1 atm H_2 in C_6D_6 with 1 µL mesitylene internal standard. Integration of hydroquinone product done via direct integration relative to internal standard.

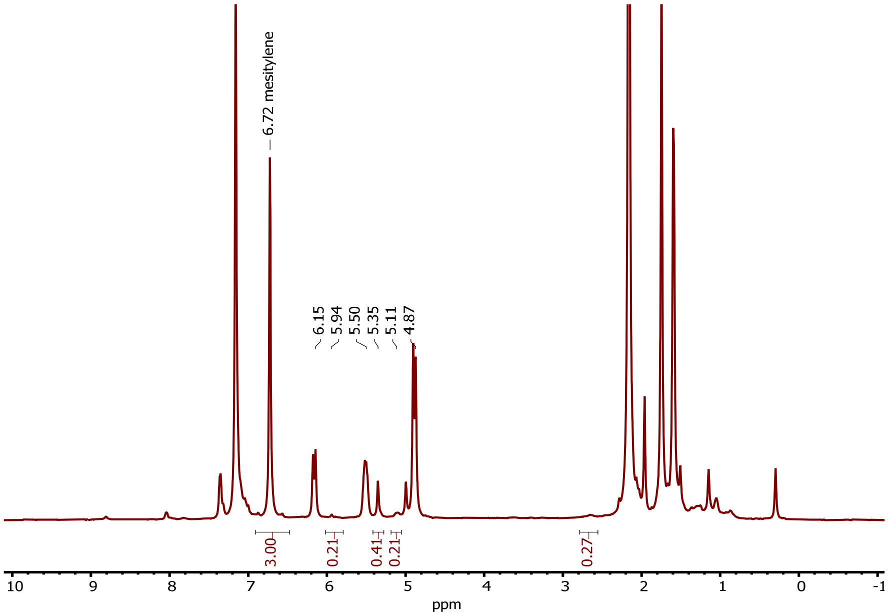

Figure S97. ^{1}H NMR of 2.5 mol%: **2** hydrogenation of 2-methyl-pent-1,3-ene with 1 atm H_2 in C_6D_6 with 1 µL mesitylene internal standard. Integration of 4-methyl-2-pentene (cis- and trans-) and 2-methyl-pent-2-ene products done via direct integration relative to internal standard.

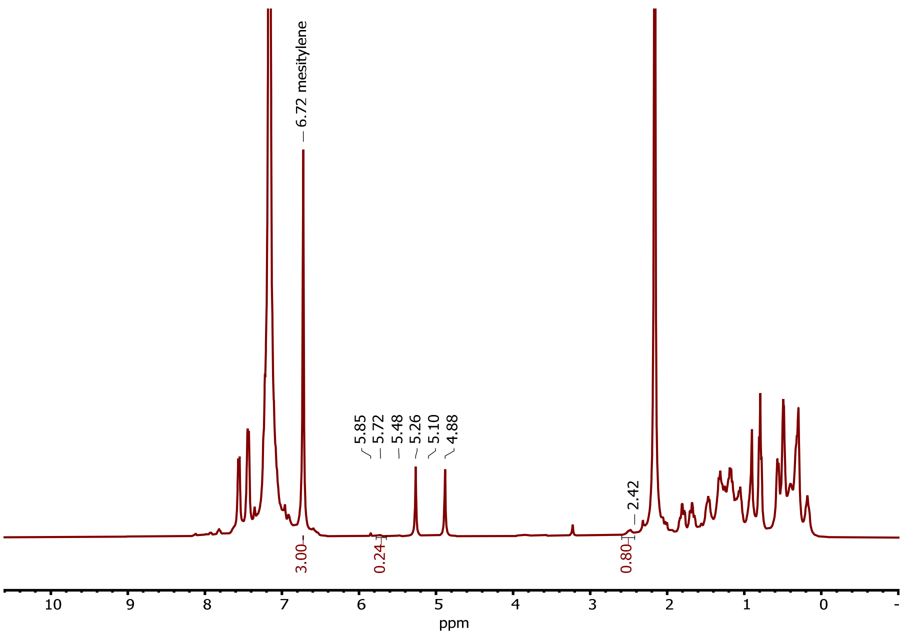

Figure S98. ^{1}H NMR of 10 mol%: **2** hydrogenation of α-cyclopropylstyrene with 1 atm H_2 in THF and C_6D_6 with 1 μL mesitylene internal standard. Integration of various products done directly.

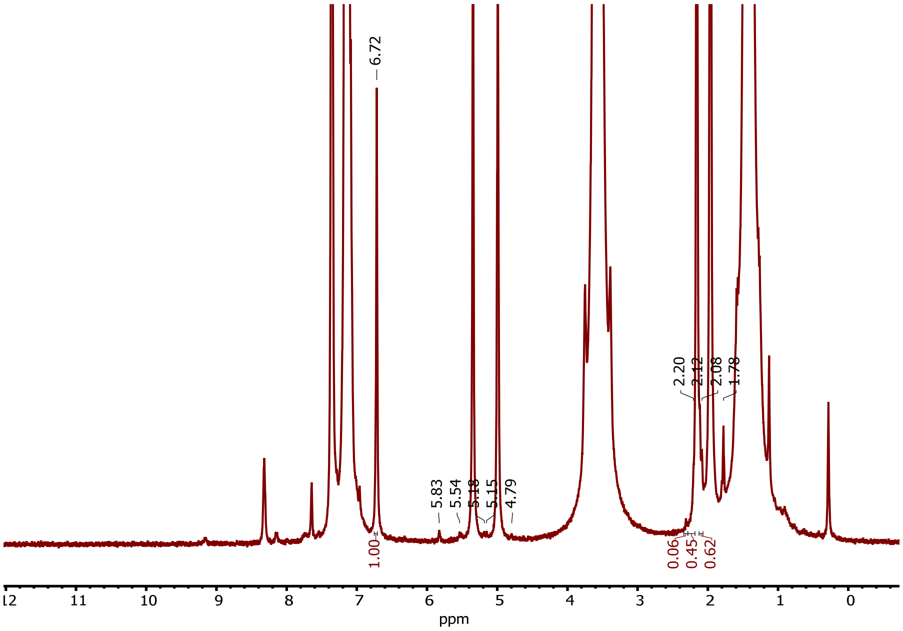

Figure S99. ^{1}H NMR of 2.5 mol%: **2** hydrogenation of α-methylstyrene with 1 atm H_2 in C_6D_6 with 1 μL mesitylene internal standard. Integration of various products done directly.

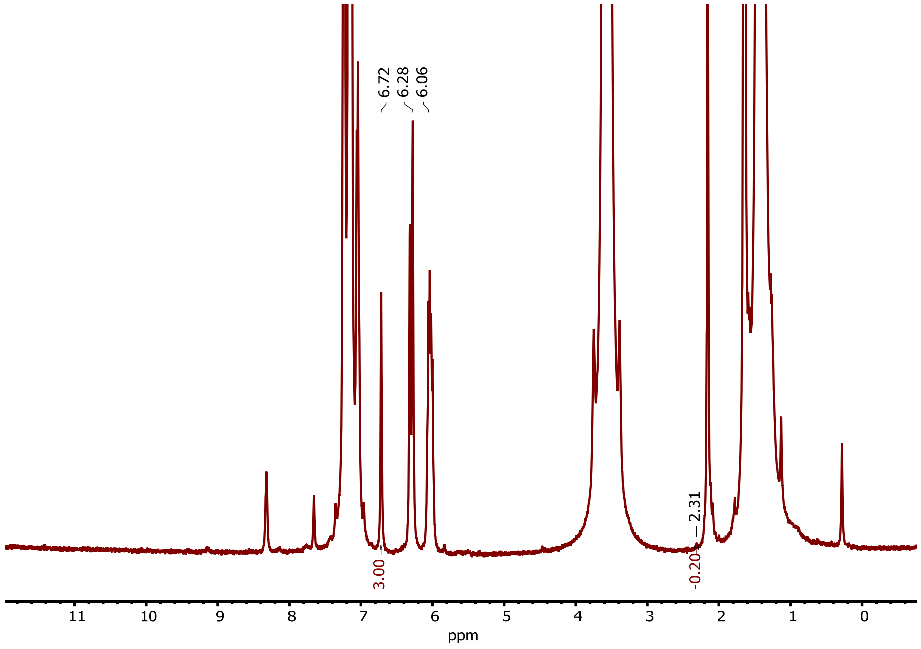

Figure S100. ^{1}H NMR of 2.5 mol%: **2** hydrogenation of β-methylstyrene with 1 atm H_2 in C_6D_6 with 1 μL mesitylene internal standard. Integration of various products done directly.

References

1. Martin, J.; Knüpfer, C.; Eyselein, J.; Färber, C.; Grams, S.; Langer, J.; Thum, K.; Wiesinger, M.; Harder, S. Highly Active Superbulky Alkaline Earth Metal Amide Catalysts for Hydrogenation of Challenging Alkenes and Aromatic Rings. *Angewandte Chem.*, **2020**, *23*, 9102-9112.

2. Stoll, S.; Schweiger, A. EasySpin, a comprehensive software package for spectral simulation and analysis in EPR. *J. Magn. Reson.* **2006**, *178*, 42-55.

3. McNeece, A. J.; Jesse, K. A.; Xie, J.; Filatov, A. S.; Anderson, J. S. Generation and Oxidative Reactivity of a Ni (II) Superoxo Complex via Ligand-Based Redox Non-Innocence. *J. Am. Chem. Soc.*, **2020**, *142*, 10824-10832.

4. G. M. Sheldrick, Crystal structure refinement with SHELXL. *Acta Crystallogr.* **2015,** *C71*, 3-8.

5. O. V. Dolomanov, L. J. Bourhis, R. J. Gildea, A. K. Howard, H. Puschmann, OLEX2: a complete structure solution, refinement and analysis program, *J. Appl. Cryst.* **2009**. *42*, 339.

6. G. M. Sheldrick. *Acta Crystallogr.* **2008***, A64*, 112-122.

7. (a) Neese, F. The Orca Program System. *Wiley Interdiscip. Rev. Comput. Mol. Sci.* **2012**, *2*, 73-78. (b) H - Kr: A. Schaefer, H. Horn and R. Ahlrichs, Fully optimized contracted Gaussian basis sets for atoms Li to Kr. *J. Chem. Phys.* **1992**, *97*, 2571. (c) Rb - Xe: A. Schaefer, C. Huber and R. Ahlrichs, Fully optimized contracted Gaussian basis sets of triple zeta valence quality for atoms Rb to Xe. *J. Chem. Phys.* **1994** *100*, 5829. (d) F. Weigend, R. Ahlrichs, Balanced basis sets of split

valence, triple zeta valence and quadruple zeta valence quality for H to Rn: Design and assessment of accuracy. *Phys. Chem. Chem. Phys.* **2005**, 7, 3297.
8. NIST Computational Chemistry Comparison and Benchmark Database, NIST Standard Reference Database Number 101. Release 21, August 2020, Editor: Russell D. Johnson III
9. Schwieger, S.; Herzog, R.; Wagner, C.; Steinborn, D. Platina-β-diketones as catalysts for hydrosilylation and their reactivity towards hydrosilanes. *J. Organomet. Chem.* **2009**, *694*, 3548-3558.
10. Wang, Y.; Chen, W.; Lu, Z.; Hua Li, Z.; Wang, H. Metal-Free HB(C6F5)2-Catalyzed Hydrogenation of Unfunctionalized Olefins and Mechanism Study of Borane-Mediated σ-Bond Metathesis. *Angew. Chem. Int. Ed.*, **2013**, *29*, 7496-7499.
11. Spielmann, J.; Buch, F.; Harder, S. Early Main-Group Metal Catalysts for the Hydrogenation of Alkenes with H_2. *Angew. Chem. Int. Ed.,* **2008,** *49,* 9434-9438.
12. Henry, A. T.; Cosby, T. P. L.; Boyle, P.D.; Baines, K. M. Selective dimerization of α-methylstyrene by tunable bis (catecholato) germane Lewis acid catalysts. *Dalton Trans.*, **2021**, *50*, 15906–15913.
13. McNeece, A. J.; Jesse, K. A.; Filatov, A. S.; Schneider, J. E.; Anderson, J. S. Catalytic hydrogenation enabled by ligand-based storage of hydrogen. *ChemComm.* **2021**, *57*, 3869-3872.
14. Harris, R. K.; Ng, S.; Connelly, A. Proton-coupled carbon-13 NMR spectra of butadienes. *Magn Reson Chem.* **1991**, *29*, 1152-1157.
15. (a) Chahboun, G.; Petrisor, C. E.; Gómez-Bengoa, E.; Royo, E.; Cuenca, T. Insight into cis-to-trans Olefin Isomerisation Catalysed by Group 4 and 6 Cyclopentadienyl Compounds. *Eur. J. Inorg. Chem.* **2009**, *11*,1514-1520.;(b) Pinkas, J.; Gyepes, R.; Císařová, I.; Kubišta, J.; Horáček, M.; Mach, K. Steric Effects in Reactions of Decamethyltitanocene Hydride with Internal Alkynes, Conjugated Diynes, and Conjugated Dienes. *Organometallics.* **2014**, *13*, 3399–3413.
16. Kristensen, Steffan K.; Laursen, Simon L. R.; Taarning, Esben; Skrydstrup, Troels. Ex Situ Formation of Methanethiol: Application in the Gold (I)-Promoted Anti-Markovnikov Hydrothiolation of Olefins. *Angew. Chem. Int. Ed.*, **2018**, *57*, 42, 13887 – 13891.
17. Masnovi, J.; Samsel, E. G.; Bullock, R. M. Cyclopropylbenzyl Radical Clocks. *J. Chem. Soc., ChemComm.* **1989**, 1044−1045.
18. (a) Harada, S.; Matsuda, D.; Morikawa, T.; Nishida, A. Direct Synthesis of Enones by Visible-Light-Promoted Oxygenation of Trisubstituted Olefins Using Molecular Oxygen. *Synlett*. **2020**, *31*, 1372-1377.; (b) Bedford, R. B.; Brenner, P. B.; Carter, E.; Carvell, T. W.; Cogswell, P. M.; Gallagher, T.; Harvey, J. N.; Murphy, D. M.; Neeve, E. C.; Nunn, J.; Pye, D. R. Expedient Iron-Catalyzed Coupling of Alkyl, Benzyl and Allyl Halides with Arylboronic Esters. *Chem. A. Eur. Journal.* **2014**, *20*, 7935-7938.
19. Britovsek, G. J. P.; Bruce, M.; Gibson, V. C.; Kimberley, B. S.; Maddox, P. J.; Mastroianni, S.; McTavish, S. J.; Redshaw, C.; Solan, G. A.; Stömberg, S.; White, A. J. P.; Williams, D. J. Iron and cobalt ethylene polymerization catalysts bearing 2, 6-bis (imino) pyridyl ligands: synthesis, structures, and polymerization studies. *J. Am. Chem. Soc.* **1999**, 121, 38, 8728-8740.

20. Morris Bullock; Samsel, Edward G. Hydrogen atom transfer reactions of transition-metal hydrides. Kinetics and mechanism of the hydrogenation of. alpha.-cyclopropylstyrene by metal carbonyl hydrides. *J. Am. Chem. Soc.* **1990**, *112*, 19, 6886 – 6898.
